# Gaudichaudione H Enhances the Sensitivity of Hepatocellular Carcinoma Cells to Disulfidptosis via Regulating NRF2‐SLC7A11 Signaling Pathway

**DOI:** 10.1002/advs.202411131

**Published:** 2025-01-22

**Authors:** Mengjiao Shi, Xinyan Li, Ying Guo, Yinggang Zhang, Jiayi Xu, Liangwen Yan, Rongrong Liu, Hong Wang, Shenkang Tang, Yaping Zhao, Zongfang Li, Yetong Feng, Dongmei Ren, Pengfei Liu

**Affiliations:** ^1^ Department of General Surgery National & Local Joint Engineering Research Center of Biodiagnosis and Biotherapy The Second Affiliated Hospital of Xi'an Jiaotong University Xi'an 710004 China; ^2^ International Joint Research Center on Cell Stress and Disease Diagnosis and Therapy National & Local Joint Engineering Research Center of Biodiagnosis and Biotherapy The Second Affiliated Hospital of Xi'an Jiaotong University Xi'an 710004 China; ^3^ Shaanxi Provincial Clinical Research Center for Hepatic & Splenic Diseases The Second Affiliated Hospital of Xi'an Jiaotong University Xi'an 710004 China; ^4^ Department of Oncology Affiliated Hospital of Shaanxi University of Chinese Medicine Xianyang 712000 China; ^5^ Core Research Laboratory The Second Affiliated Hospital of Xi'an Jiaotong University Xi'an 710004 China; ^6^ Key Laboratory of Chemical Biology (Ministry of Education) School of Pharmaceutical Sciences Shandong University Jinan 250012 China; ^7^ Key Laboratory of Environment and Genes Related To Diseases Xi'an Jiaotong University Ministry of Education of China Xi'an 710061 China

**Keywords:** disulfidptosis, Gaudichaudione H, hepatocellular carcinoma, NRF2, SLC7A11

## Abstract

Gaudichaudione H (GH) is a naturally occurring small molecular compound derived from *Garcinia oligantha Merr*. (Clusiaceae), but the full pharmacological functions remain unclear. Herein, the potential of GH in disulfidptosis regulation, a novel form of programmed cell death induced by disulfide stress is explored. The omics results indicated that NRF2 signaling could be significantly activated by GH. The potential targets are associated with hepatocarcinogenesis and cell death. Moreover, both glutathione (GSH) metabolism and NADP^+^‐NADPH metabolism are affected by GH, indicating the potential in disulfidptosis regulation. It is also observed that GH enhanced the sensitivity of hepatocellular carcinoma (HCC) cells to disulfidptosis, which is dependent on the activation of NRF2‐SLC7A11 pathway. GH significantly increased the levels of NRF2 and promoted the transcription of NRF2 target gene, SLC7A11, through autophagy‐mediated non‐canonical mechanism. Under the condition of glucose starvation, GH‐induced upregulation of SLC7A11 aggravated uptake of cysteine, disturbance of GSH synthesis, depletion of NADPH, and accumulation of disulfide molecules, ultimately leading to the formation of disulfide bonds between different cytoskeleton proteins and disulfidptosis eventually. Collectively, the findings underscore the potential role of GH in promoting cancer cell disulfidptosis, thereby offering a promising avenue for the treatment of drug‐resistant HCC in clinical settings.

## Graphical abstract

Gaudichaudione H (GH) effectively activates NRF2 signaling and its target genes, such as SLC7A11, and pharmaceutical activation of NRF2‐SLC7A11 signaling pathway aggravates uptake of cysteine, disturbance of GSH synthesis, depletion of NADPH, and accumulation of disulfide molecules, ultimately leading to the formation of disulfide bonds between different cytoskeleton proteins and disulfidptosis eventually.

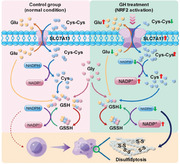

## 1 Introduction

As a key component in system Xc^−^‐ and a canonical ferroptosis suppressor, a novel and unexpected role for solute carrier family 7 member 11 (SLC7A11) has recently been uncovered in promoting disulfidptosis, a novel form of programmed cell death induced by disulfide stress that is distinct from apoptosis, autophagic cell death, necroptosis, or ferroptosis. Glucose‐derived nicotinamide adenine dinucleotide phosphate (NADPH) acts as a key reducing agent during the process of converting SLC7A11‐imported cystine to cysteine, and suppresses the formation of disulfide bond between cytoskeleton proteins. Under conditions of glucose starvation, SLC7A11‐mediated cystine uptake and its subsequent reduction to cysteine exhaust NADPH, leading to aberrant disulfide bonds in actin cytoskeleton proteins, action network collapse and disulfidptosis in an SLC7A11‐dependent manner. Glucose starvation‐induced disulfidptosis can be mitigated through suppressing the WAVE (WASP family Verprolin homolog) regulatory complex, which regulates actin polymerization and lamellipodia formation.^[^ ^1^ ^]^ Thus, disulfidptosis highlights the importance of balancing cystine uptake and glucose metabolism as a novel therapeutic strategy against cancer and other diseases. However, the underlying regulatory mechanisms of disulfidptosis in tumor progression and drug sensitivity are not yet fully understood. Some researchers have elucidated a comprehensive molecular landmarks of disulfidptosis in different cancer types via multi‐omics analysis, offering opportunities to overcome drug resistance for clinical cancer therapy.^[^ ^2^ ^]^ Nevertheless, the accuracy and validity of these molecular profiles, particularly in relation to disulfidptosis‐related alterations and prognostic predictions, require further validation in vitro and in vivo. Moreover, few effective small molecular modulators of disulfidptosis have been currently identified, limiting the application of disulfidptosis model in clinical settings.

Gaudichaudione H (GH) is a naturally occurring small molecular compound isolated from *Garcinia oligantha Merr*. (Clusiaceae). As an unusual and rare caged polyprenylated xanthone, GH has garnered scientists’ attention because of its important bioactive properties. For example, GH has been shown to induce apoptosis in cancer cells and effectively suppress cancer cell growth, suggesting its significant potential in cancer treatment.^[^ ^3^ ^]^ In 2020, some researchers conducted the first analysis of GH‐induced anti‐inflammatory effect in vitro and in vivo, and the study revealed that treatment with GH effectively inhibited the expression of various inflammatory cytokines and improved histological damage. In addition, the macrophage infiltration was also reduced by GH treatment.^[^ ^4^ ^]^ Recently, the GH‐induced toxicity was tested in a zebrafish model. The results indicated that a high dose of GH could lead to cardiotoxicity, cardiovascular defects, and embryonic mortality during the development progress of zebrafish embryo. More importantly, the effect of GH on some metabolic pathways associated with Heme binding and Iron ion binding could be the potential mechanism of GH‐induced toxicity.^[^ ^5^ ^]^ However, the complete pharmacological functions of GH remain to be fully elucidated, and the major biological activities and potential pharmacological targets still require further investigation.

As the most common form of liver cancer, hepatocellular carcinoma (HCC) has currently become one of the most deadly cancers. Patients with hepatitis B/C infection or other chronic liver illnesses have an increased risk of developing HCC. Human HCC is typically detected at an advanced stage and is distinguished by a high degree of drug resistance to chemotherapy, which limits the effectiveness of chemotherapy and increases the risk of recurrence following treatment.^[^ ^6^ ^]^ Therefore, developing strategies to overcome drug resistance is essential in the treatment of HCC. In recent years, the development of novel cell death model provides multiple promising avenues for the treatment of drug‐resistant HCC in pre‐clinical and clinical settings. For instance, some researchers observed that low‐dose Oxaliplatin combined with the dihydroorotate dehydrogenase (DHODH)‐inhibitor Leflunomide suppressed the progression of HCC via inducing ferroptosis, which significantly enhanced chemotherapy sensitivity and relieved chemotherapy toxicity.^[^ ^7^ ^]^ Additionally, some studies revealed the function of proteasome inhibitors in inducing HCC pyroptosis, and identified the inhibition of proteasome activity and WEE family kinases as a promising anti‐cancer strategy targeting solid tumor cells.^[^ ^8^ ^]^ Recently, the relationship between disulfidptosis and HCC treatment was also investigated by several groups, and these studies showed that disulfidptosis‐related genes hold significant associations with tumor stemness, prognosis, genetic variations, as well as drug sensitivity. Moreover, some functional genes (such as RPN1 and SLCO1B1) involved in disulfidptosis regulation have great potential to become novel targets in the treatment of HCC.^[^ ^9^ ^]^

In the present study, our findings primarily suggested that the treatment with GH heightened the susceptibility of HCC to glucose starvation‐induced disulfidptosis, but not ferroptosis. Mechanistically, GH targeted nuclear factor erythroid 2‐related factor 2 (NRF2), a key transcription factor regulating oxidative stress, via regulating autophagy. The treatment with GH significantly increased the protein level of NRF2 and promoted the transcription of NRF2 target gene, SLC7A11, which influenced glutathione (GSH) metabolism and NADP^+^‐NADPH metabolism processes in HCC, and promoted the formation of disulfide bonds between different cytoskeleton proteins as well as disulfidptosis ultimately. Therefore, pharmacological targeting of NRF2‐SLC7A11 axis offer a new perspective on the precise cancer treatment via modulating disulfidptosis.

## 2 Results

### 2.1 Network Pharmacology Analysis of GH Potential Targets and Function

**Figure 1:**
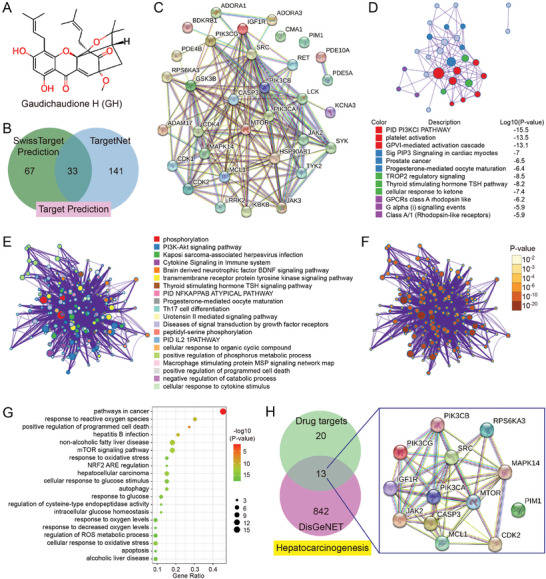
Network pharmacology assay of GH potential bioavailable targets and therapeutic action. The chemical structure of GH was shown in Figure 1A initially. The potential bioavailable targets of GH were predicted using SwissTargetPrediction database and TargetNet database respectively (B). The overlap section between the results from two database were applied to create protein–protein interaction network (C), and the connected network components were identified using the molecular complex detection (MCODE) algorithm (D). In addition, the overlap genes were used for cluster analysis using Metascape, and the top 20 enriched terms in cluster analysis were organized as a network plot (E,F). The items related with oxidative stress and liver diseases are summarized in Figure 1G. At last, the potential bioavailable targets of GH associated with hepatocarcinogenesis were organized in Figure 1H.

In our study, the potential bioavailable targets of GH were predicted using SwissTargetPrediction database and TargetNet database respectively, which was based on the chemical structure of GH (**Figure** **1**A). Herein, a total of 100 potential bioavailable targets were predicted by SwissTargetPrediction database, and 174 potential bioavailable targets were predicted by TargetNet database. There were 35 overlapping targets between the results from the two database (Figure 1B and Table S1, Supporting Information), and these overlapping targets were chosen for cluster analysis using Metascape. A protein‐protein interaction network was established first, and the connected network components were identified using the molecular complex detection (MCODE) algorithm. The results showed that PID PI3KCI PATHWAY, platelet activation, and GPVI‐mediated activation cascade were the most important pathway and process related with GH function (Figure 1C,D). In addition, the top 20 enriched terms in cluster analysis were organized as a network plot. We noticed that phosphorylation and PI3K‐Akt signaling pathway were the most relevant biological process and pathway, and the pathways related with some metabolic process and programmed cell death were also considered as the potential target signals of GH (Figure 1E,F). Additionally, cluster analysis revealed a strong association between cancer‐related pathways, particularly those involved in hepatocellular carcinoma, and the potential targets of GH. This suggests that GH may have therapeutic potential against liver cancer, likely through its effects on reactive oxygen species, autophagy, and oxidative stress (Figure 1G). The genes associated with hepatocarcinogenesis were summarized using DisGeNET database (Table S2, Supporting Information), and 13 potential bioavailable targets (including the key autophagy regulator MTOR) of GH were overlapped with hepatocarcinogenesis genes (Figure 1H), indicating that GH treatment could be considered as a novel approach against HCC, and PI3K/Akt/mTOR and autophagy could be potential mechanism.

### 2.2 GH Modulates Multiple Genes and Metabolites Pivotal for Hepatocarcinogenesis

**Figure 2:**
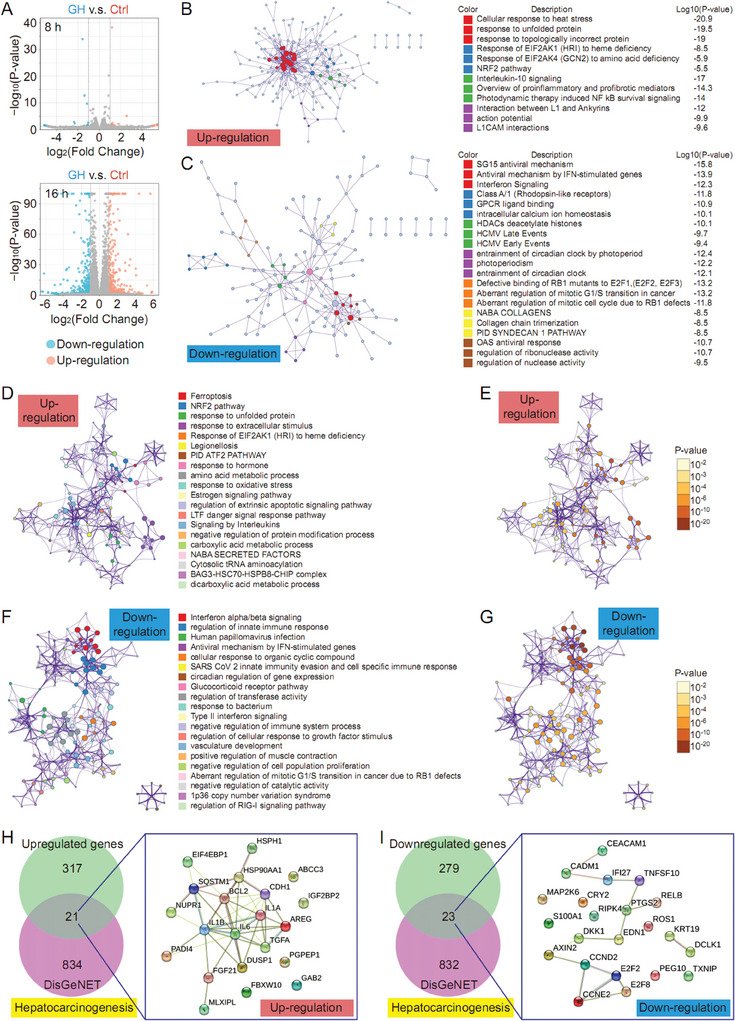
GH modulates multiple genes pivotal for hepatocarcinogenesis. In our work, HCC cell line, MHCC97‐H, was treated with GH (2 µm) for 8 h and 16 h respectively. Then the cell samples were harvested for RNA‐seq assay. First, a volcano plot was generated to evaluate the effect of GH treatment on gene transcription (A). Then, both up‐regulated genes and down‐regulated genes were analyzed using the molecular complex detection (MCODE) algorithm (B,C). In addition, differentially expressed genes were also used for cluster analysis using Metascape. Herein, network of enriched terms for both up‐regulated genes and down‐regulated genes were colored by cluster ID, and the same terms were further colored by *p*‐value (D–G). Finally, the relationship between GH‐mediated genes and hepatocarcinogenesis were analyzed, and the crosstalk among those related genes were shown in Protein‐Protein Interaction Networks created using STING database (H,I).

Network pharmacology analysis has revealed the potential therapeutic action of GH against hepatocarcinogenesis. To validate the hypothesis, an RNA‐seq assay was performed in the present study to compare the difference of genome‐wide mRNA expression profiles. Herein, HCC cell line, MHCC97‐H, was treated with GH for 8 and 16 h respectively. After 8‐h treatment, we noticed that there were only 18 genes upregulated and 16 genes downregulated in GH‐treated MHCC97‐H cells (GH group) compared with untreated cells (Ctrl group). Following a 16‐h treatment, the transcription of 338 genes were upregulated and the transcription of 302 genes were downregulated in GH group relative to Ctrl group (**Figure** **2**A and Table S3, Supporting Information), which was chosen for further cluster analysis. A protein‐protein interaction network was established based on the upregulated and downregulated genes respectively, and the connected network components were identified using the molecular complex detection (MCODE) algorithm. The results showed that cellular response to heat stress, response to unfolded protein, and response to topologically incorrect protein were the most important pathway and process related with GH‐upregulated genes, while SG15 antiviral mechanism, Antiviral mechanism by IFN‐stimulated genes, Interferon Signaling were the most important pathway and process related with GH‐downregulated genes. NRF2 pathway, NFkB survival signaling, and PID SYNDECAN 1 pathway could be the potential mechanism (Figure 2B,C). Moreover, the top 20 enriched terms in cluster analysis of upregulated genes and downregulated genes were organized as network plots. We found that Ferroptosis and NRF2 pathway were the top pathways associated with the upregulated genes (Figure 2D,E), and Interferon alpha/beta signaling and regulation of innate immune response were the top ones related with the downregulated genes (Figure 2F,G). We also analyzed the relevance of GH‐regulated genes with the genes associated with hepatocarcinogenesis in DisGeNET database. We observed that there were 21 GH‐upregulated genes and 23 GH‐downregulated genes overlapped with hepatocarcinogenesis genes. Interestingly, those GH‐upregulated genes showed greater crosstalk in Protein‐Protein Interaction Networks compared with the 23 GH‐downregulated genes (Figure 2H,I), indicating that GH‐upregulated genes could play more important role in hepatocarcinogenesis. Furthermore, both ferroptosis and NRF2 signaling pathway could be associated with the function.

**Figure 3:**
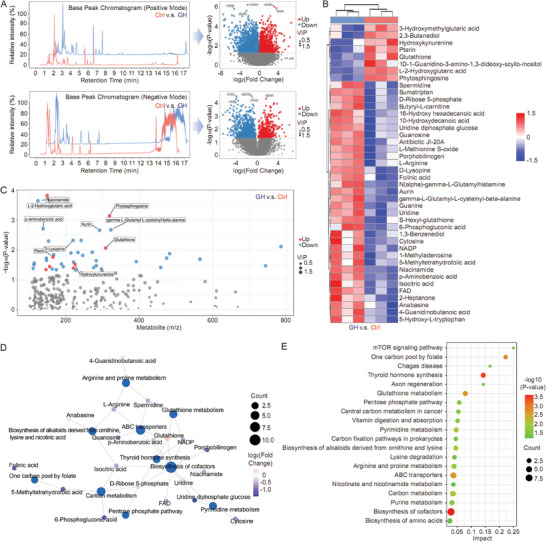
GH modulates metabolites pivotal for ferroptosis. Herein, HCC cell line, MHCC97‐H, was treated with GH (2 µm) for 16 h. Then the cell samples were harvested for metabolomics analysis. The primary metabolic shift was evaluated using both Positive Mode and Negative Mode (A). In secondary metabolites analysis, both upregulated metabolites and downregulated metabolites were summarized in Figure 3B. Besides, the association between mass‐to‐charge ratio and *p*‐value was analyzed in Figure 3C, and the crosstalk among differential metabolites and key metabolisms were summarized in a network plot (D). Finally, the important metabolism pathways in HCC cells influenced by GH are shown in Figure 3E.

Ferroptosis is a regulated cell death driven by lethal lipid peroxidation which is considered as a consequence of imbalanced cellular metabolism and redox homeostasis. Thus, metabolomics analysis was performed to evaluate the metabolic profiles in different groups. In our study, the primary metabolic shift was analyzed using both Positive Mode and Negative Mode. We found that there were 2218 downregulated metabolites and 1278 upregulated metabolites in GH‐treated HCC cells compared with untreated cells, while 961 metabolites were downregulated and 432 metabolites were upregulated in the GH group relative to Ctrl group in Negative Mode (**Figure** **3**A). In the secondary metabolites assay, the results indicated that 8 metabolites were downregulated and 34 metabolites were upregulated in the GH‐treated group compared with the Ctrl group (Figure 3B and Table S4, Supporting Information). The association between mass‐to‐charge ratio and *p*‐value was summarized in Figure 3C, and the interaction or crosstalk among differential metabolites and key metabolism pathways was organized as a network plot (Figure 3D). Our findings suggested that central carbon metabolism in cancer, cysteine and methionine metabolism, oxidative phosphorylation could be affected by GH treatment in HCC cells. In addition, ferroptosis regulation was also found to be relevant with GH treatment in metabolomics analysis (Figure 3E).

### 2.3 GH Enhances the Sensitivity of HCC Cells to Disulfidptosis, but not Ferroptosis

Both RNA‐seq assay and metabolomics analysis highlighted the promising potential of GH in the regulation of HCC ferroptosis. To further evaluate the function, we next examined the effect of GH on HCC ferroptosis. Both MHCC97‐H cells and Hep 3B cells were treated with GH at different concentration 5 and 10 µm). We found GH induced expected cell death in a dose‐dependent manner. However, GH‐induced was difficult to be rescued by ferroptosis inhibitor Ferrostatin‐1 (Fer‐1, 2.5, 5, and 10 µm), suggesting that the cellular toxicity caused by GH treatment was not due to cell ferroptosis (Figure S1A,B, Supporting Information). To confirm the hypothesis, the levels of both MDA and 4‐HNE were determined in our study. Compared with untreated group, GH treatment did not increase the level of either MDT or 4‐HNE in HCC cells, even though expected cell death could be induced by GH at the same concentrations (Figure S1C,D, Supporting Information). In addition, we also evaluated whether GH treatment sensitized HCC cells to Erastin or RSL3‐induced ferroptosis. The results indicated that GH treatment did not affect Erastin or RSL3‐induced ferroptosis in HCC cells (Figure S1E,F, Supporting Information). Consistently, GH treatment could not enhance the production of MDA or 4‐HNE in the Erastin and RSL3‐induced ferroptosis model (Figure S1G,H, Supporting Information). Overall, our findings suggest that GH could not regulate the sensitivity of HCC cells to ferroptosis.

**Figure 4:**
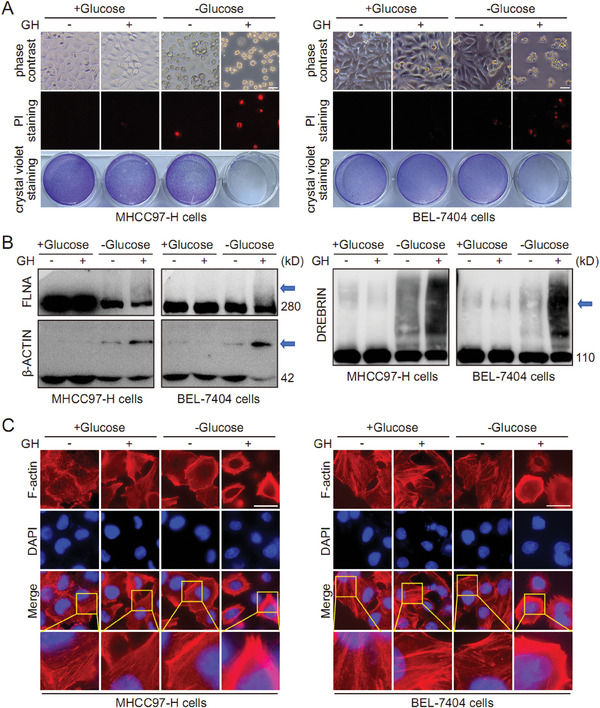
GH enhances the sensitivity of HCC cells to disulfidptosis. Herein, MHCC97‐H and BEL‐7404 cells were treated with GH (2 µm) for 16 h first, followed with glucose starvation (6–8 h) to induce disulfidptosis. Then the morphology change and cell death were evaluated in each group (A, Scale bar = 20 µm). In addition, the glucose starvation‐induced disulfide bond formation in the cytoskeleton proteins (FLNA, β‐ACTIN, and DREBRIN) was evaluated using non‐reducing western blot (B). Finally, phalloidin staining was applied to evaluate the actin filament (F‐actin) in different groups (C, Scale bar = 10 µm).

Although GH did not affect the response of HCC cells to ferroptosis, our metabolomics data showed that both GSH metabolism and NADP metabolism were affected by GH treatment (Figure 3B). Recently, a novel form of programmed cell death induced by disulfide stress, disulfidptosis, was reported by scientists. As a key component in system Xc^−^‐ and a canonical ferroptosis suppressor, SLC7A11, plays a critical role in disulfidptosis regulation. More importantly, both GSH metabolism and NADP metabolism were linked with disulfidptosis. Therefore, we next examined the influence of GH on disulfidptosis in HCC cells. In this study, four different HCC cell lines (MHCC97‐H, Hep 3B, BEL‐7402, and BEL‐7404) were treated with GH (2 µm) for 16 h first, followed with glucose starvation (6‐8 h) to induce disulfidptosis. Our results showed that pre‐treatment with GH significantly increased the sensitivity of HCC cells to glucose starvation‐induced disulfidptosis (**Figures** **4**A and S2A, Supporting Information). Moreover, the glucose starvation‐induced disulfide bond formation in cytoskeleton proteins was evaluated using non‐reducing western blot as the reference.^[^ ^1a^ ^]^ The results showed that both FLNA and DREBRIN exhibited significantly slower migration with smears, which was enhanced by GH pre‐treatment. The results for β‐ACTIN also exhibited a similar tendency, although much weaker compared to the results of FLNA and DREBRIN (Figure 4B). In addition, phalloidin staining was applied to evaluate the actin filament (F‐actin) in different groups. The results indicated that the F‐actin was primarily organized in the cell cortex and stress fibers under normal condition. Differently, morphological changes were induced by glucose starvation, characterized by F‐actin contraction and marginal clustering. GH pre‐treatment enhanced the morphological change of F‐actin, which was consistent with the disulfidptosis observations (Figure 4C). Moreover, the GLUT1 inhibitor BAY‐876 was used to induce disulfidptosis via pharmacological blockade of glucose uptake as the reference.^[^ ^1a^ ^]^ Similar to the glucose starvation‐induced disulfidptosis model, we observed that GH pre‐treatment enhanced the sensitivity of HCC cells to BAY‐876‐induced disulfidptosis (Figure S2B, Supporting Information).

### 2.4 GH Activates NRF2 Signaling Pathway in HCC Cells Through an Autophagy‐Mediated Non‐Canonical Mechanism

**Figure 5:**
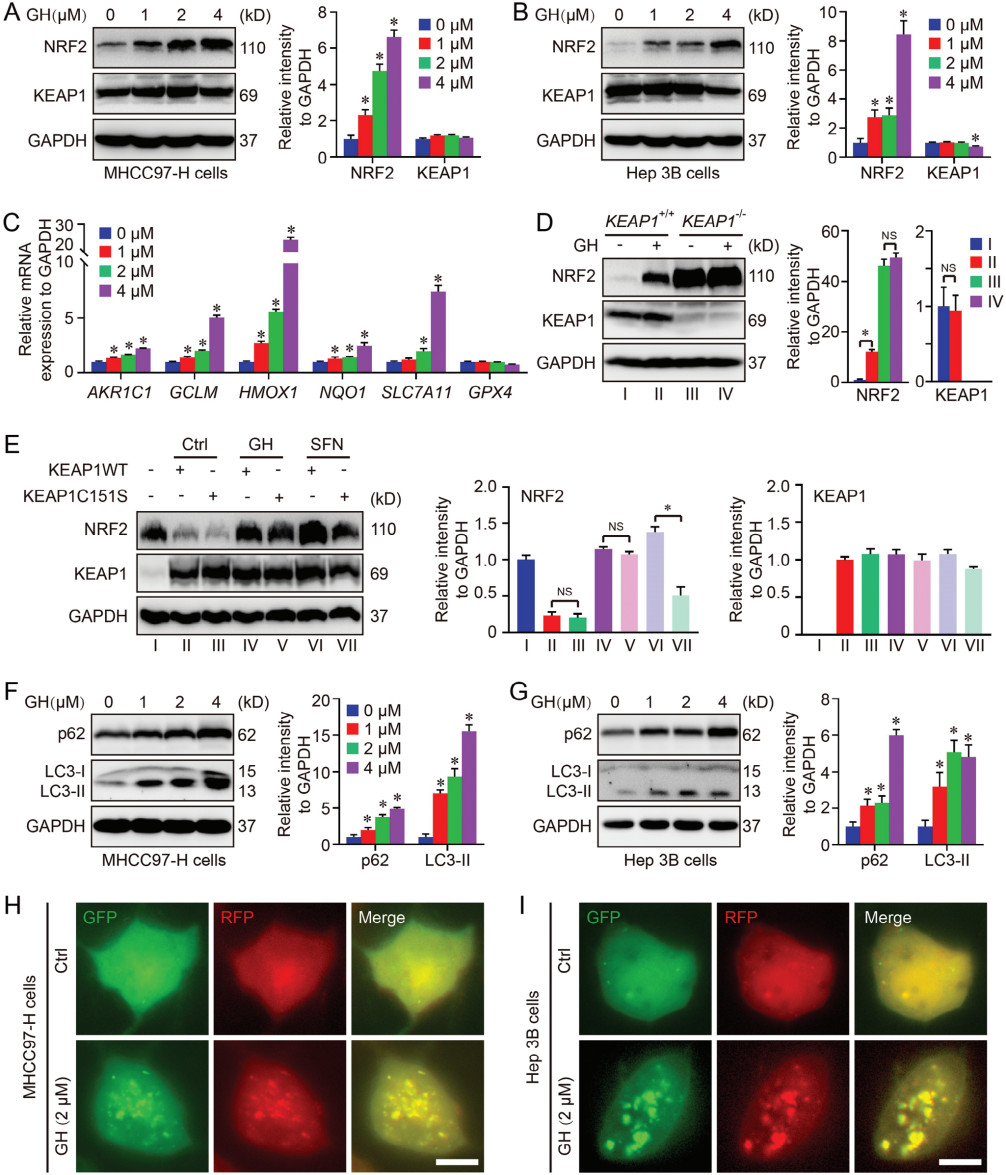
GH treatment leads to NRF2 activation in HCC cells. In the current work, HCC cells (MHCC97‐H and Hep 3B) were treated with GH in different concentrations (0, 1, 2, and 4 µm) for 16 h, and the protein level of NRF2 was determined using western blot (A,B). To confirm the activation of NRF2 signaling pathway, the transcription of NRF2 target genes (*AKR1C1*, *GCLM*, *HMOX1*, *NQO1*, *SLC7A11*, and *GPX4*) was evaluated using qPCR (C). *KEAP1* knockout MHCC97‐H cell line was created using CRISPR/Cas9, and the effect of GH on NRF2 signal was further test in both wild‐type cells and *KEAP1* knockout cells (D). We next tested the position of KEAP1‐Cys151 in GH‐induced NRF2 activation, and *KEAP1* knockout cells were transfected with either KEAP1‐WT (wild‐type) or KEAP1‐C151S (mutation). Then the transfected cells were treated with GH (2 µm) and sulforaphane (SFN, a canonical NRF2 activator, 5 µm) for 16 h. The protein level of NRF2 in each group was determined using western blot (E). Besides, the influence of GH treatment on autophagy in HCC cells was analyzed in our work, and the protein levels of both p62 and LC3 (two key autophagy makers) were measured using western blot (F,G). Moreover, HCC cells were transfected with mRFP‐GFP‐LC3 reporter construct for autophagic flux assay (H,I). (Data were presented as means ± SD. *: *p* < 0.05 compared with Ctrl groups, Scale bar = 10 µm).

Our RNA‐seq assay suggested that GH played an important role in hepatocarcinogenesis, and the function was associated with NRF2 signaling pathway which was considered as the master regulator of cellular redox homeostasis and oxidative stress. Therefore, the effect of GH on NRF2 signal was evaluated in our work. Because disulfidptosis model is closely related with glucose metabolism, in which both MHCC97‐H and Hep 3B cell lines have been used by several groups in recent years.^[^ ^10^ ^]^ Thus, those two cell lines were chosen for further analysis. HCC cells were treated with GH at different concentrations (0, 1, 2, and 4 µm) for 16 h, and the protein level of NRF2 was determined using western blot. The results showed that the protein level of NRF2 in GH‐treated HCC cells increased in a dose‐dependent manner, compared with untreated HCC cells. Besides, we also tested the expression of E3 ligase adaptor Kelch‐like ECH‐associated protein 1 (KEAP1) which was considered as the principal negative regulator of NRF2. We found that GH treatment did not show a significant effect on the expression of KEAP1 except at the high dose of GH in Hep 3B cells (**Figure** **5**A,B). We also tested the effect of GH on NRF2 activation at different time points. The results showed that GH treatment induced expected NRF2 activation even with short‐time treatment (4‐ and 8‐h, Figure S3A,B, Supporting Information). Subsequently, the transcription of NRF2 target genes (*AKR1C1*, *GCLM*, *HMOX1*, *NQO1, SLC7A11, and GPX4*) was evaluated using qPCR. Consistent with the expression of NRF2, the transcription levels of most NRF2 target genes were upregulated by GH treatment, indicating the potential of GH for NRF2 activation (Figure 5C).

As the component of E3 ubiquitin ligase during the process of NRF2 ubiquitylation, KEAP1 is considered as the most important regulator of NRF2 signaling pathway. Therefore, the effect of GH on NRF2 signal was further tested in both wild‐type and *KEAP1* knockout MHCC97‐H cells. The results revealed that the protein level of NRF2 could not be increased by GH in *KEAP1* knockout cells, indicating that the effect of GH on NRF2 activation was in a KEAP1‐dependent manner (Figure 5D). Previous studies have reported that Cys151 in KEAP1 is the major cysteine sensor during the process of canonical NRF2 activation.^[^ ^11^ ^]^ Therefore, we next tested the position of KEAP1‐Cys151 in GH‐induced NRF2 activation. Herein, *KEAP1* knockout liver cells were transfected with either KEAP1‐WT (wild‐type) or KEAP1‐C151S (mutation). Then the transfected cells were treated with GH and sulforaphane (SFN, a canonical NRF2 activator) for 16 h. The protein level of NRF2 in each group was determined using western blot. The results showed that the canonical NRF2 activator SFN enhanced the expression of NRF2 in the presence of KEAP1‐WT, which could be blocked by KEAP1‐C151S. In contrast, GH treatment activated the expression of NRF2 in both KEAP1‐WT group and KEAP1‐C151S group, in which no difference could be observed. These results indicate that NRF2 signaling was activated by GH treatment in a KEAP1‐C151‐independent manner, which had been identified as non‐canonical activation (Figure 5D). The non‐canonical activation of NRF2 is mainly dependent of autophagy regulation, and our Network pharmacology analysis also indicated the important potential of GH in autophagy regulation (Figure 1G,H). Thus, we next analyzed the influence of GH treatment on autophagy in HCC cells. The results showed that GH treatment increased the levels of both p62 and LC3 (two key autophagy makers) in HCC cells (Figure 5E,F). More importantly, autophagic flux assay indicated that the treatment with GH led to an increase in yellow puncta (autophagosomes) compared with untreated HCC cells, suggesting that autophagic flux blockage in HCC cells was induced by GH treatment (Figure 5G,H). Therefore, the upregulation of p62 and LC3 was mainly due to GH‐induced blockage of autophagic flux, contributing to non‐canonical NRF2 activation in HCC cells.

### 2.5 GH Sensitizes HCC cells to Disulfidptosis via NRF2‐SLC7A11 Signaling Pathway

**Figure 6:**
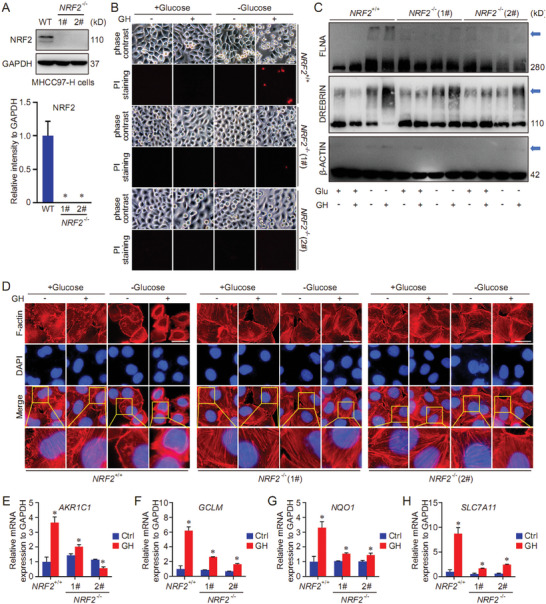
GH sensitizes HCC cells to disulfidptosis in a NRF2‐dependent manner. *NRF2* knockout HCC cell line was established using CRISPR/Cas9 technique, and the knockout effect was confirmed using western blot (A). Both wild‐type and *NRF2* knockout MHCC97‐H cells were treated with GH (2 µm) for 16 h first, followed with glucose starvation (6–8 h) to induce disulfidptosis. Then the morphology change and cell death were evaluated in each group (B, Scale bar = 20 µm). In addition, the glucose starvation‐induced disulfide bond formation in the cytoskeleton proteins (FLNA, β‐ACTIN, and DREBRIN) was evaluated using non‐reducing western blot (C). Next, phalloidin staining was applied to evaluate the actin filament (F‐actin) in different groups (D, Scale bar = 10 µm). Finally, the transcription of NRF2 target genes (AKR1C1, GCLM, NQO1, and SLC7A11) was evaluated using qPCR in different groups (E–H). (Data were presented as means ± SD. *: *p* < 0.05 compared with Ctrl groups).

Our qPCR data indicated that the transcription of NRF2 target genes (*AKR1C1*, *GCLM*, *HMOX1*, *NQO1, and SLC7A11*) was upregulated by GH treatment in HCC cells (Figure 5C). In addition, we also tested the effect of GH treatment on transcription of disulfidptosis‐related genes (*SLC7A11, SLC3A2, RPN1, NCKAP1, NUBPL, NDUFA11, LRPPRC, OXSM, NDUFS1, GYS1, GSR*) as the report.^[^ ^1a^ ^]^ The results indicated that the transcription of most genes could be activated by GH treatment. Among these genes, the transcription of *SLC7A11* showed the most pronounced increase after GH treatment (Figure S4A, Supporting Information), indicating the important position of NRF2‐SLC7A11 signaling pathway in the function of GH on disulfidptosis regulation. To ascertain the hypothesis, *NRF2* knockout HCC cell line was established using CRISPR/Cas9 technique (**Figure** **6**A). The effect of GH on disulfidptosis was evaluated in both wild‐type and *NRF2* knockout cells. The results indicated that GH sensitized wild‐type HCC cells to glucose starvation‐induced disulfidptosis, which could be totally suppressed by *NRF2* knockout (Figure 6B). Furthermore, the non‐reducing western blot also showed that the slower migration of cytoskeleton proteins were rescued in *NRF2* knockout HCC cells (Figure 6C). Moreover, the phalloidin staining results exhibited that glucose starvation‐induced F‐actin contraction and marginal clustering could be enhanced by GH treatment, and NRF2 knockout effectively blocked GH function and inhibited glucose starvation‐induced disulfidptosis (Figure 6D). GH‐induced activation of NRF2 target genes were evaluated in both wild‐type and *NRF2* knockout cells. As expected, the GH‐induced activation of NRF2 target genes (*AKR1C1*, *GCLM*, *NQO1, and SLC7A11*) was significantly suppressed in *NRF2* knockout cells compared with wild type cells (Figure 6E–H). Additionally, the effect of GH treatment on transcription of other disulfidptosis‐related genes was also evaluated in both wild‐type and *NRF2* knockout cells. The results indicated that GH regulated the transcription of some disulfidptosis‐related genes (e.g. *LRPPRC*, *NUBPL*, and *OXSM*) in a NRF2 dependent manner, further supporting the important position of NRF2 in disulfidptosis regulation (Figure S5A–I, Supporting Information).

**Figure 7:**
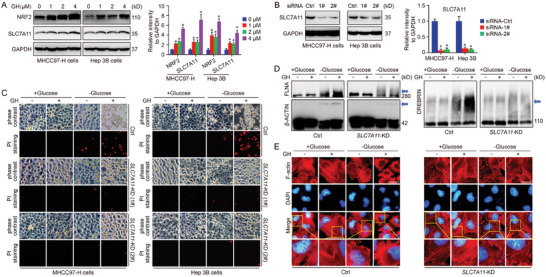
SLC7A11 holds a critical position in function of GH on disulfidptosis. HCC cells (MHCC97‐H and Hep 3B) were treated with GH in different concentrations (0, 1, 2, and 4 µm) for 16 h, and the protein level of NRF2 and SLC7A11 was determined using western blot (A,B). In addition, *SLC7A11* knockdown cells were established using siRNA transfection technique, and the knockdown effect was analyzed using western blot (B). Both wild‐type and *SLC7A11* knockdown HCC cells were treated with GH (2 µm) for 16 h first, followed with glucose starvation (6–8 h) to induce disulfidptosis. Then the morphology change and cell death were evaluated in each group (C, Scale bar = 20 µm). In addition, the glucose starvation‐induced disulfide bond formation in the cytoskeleton proteins (FLNA, β‐ACTIN, and DREBRIN) was evaluated using non‐reducing western blot (D). Finally, phalloidin staining was applied to evaluate the actin filament (F‐actin) in different groups (E, Scale bar = 10 µm). (Data were presented as means ± SD. *: *p* < 0.05 compared with Ctrl groups).

To further confirm the importance of NRF2‐SLC7A11 signaling pathway in GH function, the protein levels of NRF2 and SLC7A11 were measured using western blot in GH‐treated cells. The results indicated that both NRF2 and SLC7A11 were upregulated by GH treatment in a dose‐dependent manner (**Figure** **7**A). In addition, *SLC7A11* knockdown model was established using siRNA transfection technique (Figure 7B). The action of GH on glucose starvation‐induced disulfidptosis was evaluated in both wild‐type and *SLC7A11* knockdown HCC cells. Our results showed that GH sensitized HCC cells to disulfidptosis, which could be blocked by *SLC7A11* knockdown (Figure 7C). Moreover, the non‐reducing western blot results indicated that GH treatment enhanced the slower migration of cytoskeleton proteins, while *SLC7A11* knockdown suppressed GH function effectively (Figure 7D). The organization of F‐action in different groups was further analyzed via phalloidin staining, and the results indicated that GH treatment enhanced glucose starvation‐induced F‐actin contraction and marginal clustering in wild‐type HCC cells, but not in *SLC7A11* knockdown HCC cells (Figure 7E). These findings suggest that the NRF2 target gene, SLC7A11, plays a critical role in the function of GH on disulfidptosis regulation.

### 2.6 GH Disturbs GSH Synthesis and Exhausts NADPH in Disulfidptosis Model

**Figure 8:**
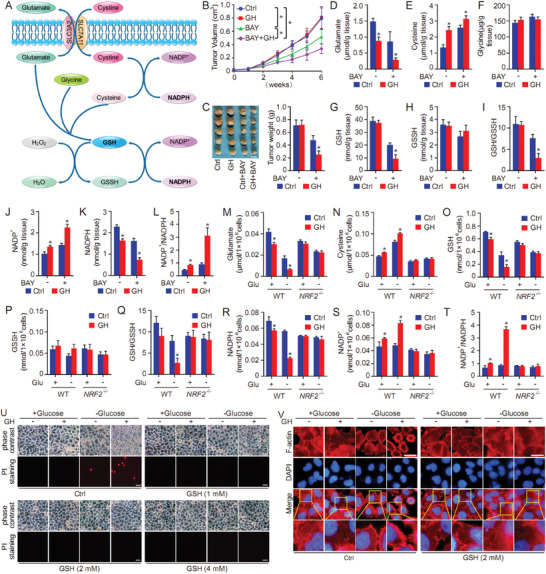
GH disturbs GSH synthesis and exhausts NADPH in disulfidptosis model. An overview of GSH synthesis and NADPH metabolism was shown in (A). Wild‐type MHCC97‐H cells were injected into SCID mice, and the mice were further treated with GH and BAY‐876 (BAY). Both tumor volume (B) and tumor weight (C) were measured in our work. In addition, the levels of glutamate (D), cysteine (E), and glycine (F) were determined in tumor tissues from each group, and both GSH‐GSSH metabolism (G–I) and NADP^+^‐NADPH metabolism (J–L) in tumor tissues form different groups were evaluated in our work. On the other hand, the influence of GH on GSH synthesis and NADP^+^‐NADPH metabolism was evaluated using wild‐type and *NRF2* knockout HCC cells in vitro. Both wild‐type and *NRF2* knockout MHCC97‐H cells were treated with GH (2 µm) for 16 h first, followed with glucose starvation (6–8 h) to induce disulfidptosis. The levels of glutamate (M) and cysteine (N) were measured in each group, and both GSH‐GSSH metabolism (O–Q) and NADP^+^‐NADPH metabolism (R–T) in different groups were evaluated respectively. Then the morphology change and cell death were evaluated in each group (U, Scale bar = 20 µm), and phalloidin staining was applied to evaluate the actin filament (F‐actin) in different groups (V, Scale bar = 10 µm). (Data were presented as means ± SD. *: *p* < 0.05 compared with Ctrl groups).

As a key component in system Xc^−^‐, SLC7A11 primarily regulates the transport of extracellular cystine in exchange for intracellular glutamate release. The processes of GSH synthesis and NADPH generation are closely related to SLC7A11 function. Our metabolomics data showed that the levels of both GSH and NADP^+^ were affected by GH treatment (Figure 3B). Therefore, the influence of GH on GSH synthesis and NADP^+^‐NADPH metabolism was evaluated in vitro and in vivo (**Figure** **8**A). In this context, BAY‐876 was used to induce HCC disulfidptosis model in vivo as the reference.^[^ ^1a^ ^]^ Notably, the combination of GH and BAY‐876 exhibited better therapeutic outcomes compared with either agent alone. Both tumor volume and tumor weight were reduced in the combination group relative to the individual GH or BAY‐876 groups (Figure 8B,C). As key components in GSH synthesis and metabolism, the levels of glutamate, cysteine, and glycine were determined in tumor tissues from each group. Our results revealed that GH treatment decreased the level of glutamate and increased the level of cysteine in tumor tissues, and the effect of GH was further enhanced under BAY‐867 exposure (Figure 8D,E). The level of glycine did not show a significant difference among different groups (Figure 8E). Next, both GSH‐GSSH metabolism and NADP^+^‐NADPH metabolism were evaluated in our work. We observed that GH did not affect GSH‐GSSH metabolism without co‐treatment with BAY‐876. In the BAY‐876‐induced disulfidptosis model, GH treatment decreased the level of GSH as well as the ratio of GSH/GSSH (Figure 8G–I). We then analyzed the NADP^+^‐NADPH metabolism in tumor tissues, and the results indicated that the treatment with GH decreased NADPH amount and increased the level of NADP^+^ as well as the ratio of NADP^+^/NADPH. More pronounced effects of GH were observed in disulfidptosis mode compared with normal condition (Figure 8J–L).

To further confirm the importance of NRF2‐SLC7A11 axis in the metabolism regulation, the effect of GH on GSH synthesis and NADP^+^‐NADPH metabolism was evaluated in vitro using wild‐type and *NRF2 knock*out HCC cells. We found that the effect of GH on glutamate and cysteine could be inhibited by *NRF2* knockout, and GH didn't affect the glutamate and cysteine levels in *NRF2* knockout HCC cells (Figure 8M,N). Similar to the in vivo results, GH treatment decreased the level of GSH as well as the ratio of GSH/GSSH in wild‐type cells, which could be blocked in *NRF2* knockout cells (Figure 8O–Q). Moreover, GH treatment also reduced NADPH amount and increased the level of NADP^+^ as well as the ratio of NADP^+^/NADPH in wild‐type cells, but not in *NRF2* knockout HCC cells (Figure 8R–T). Therefore, GH disturbed GSH synthesis and depleted NADPH in disulfidptosis model in an NRF2‐depedentent manner. To determine the critical position of GSH in disulfidptosis model, the effect of GH on disulfidptosis was analyzed in GSH‐treated HCC cells. Our results indicated that GH sensitized normal HCC cells to glucose starvation‐induced disulfidptosis, which could be effective suppressed by co‐treatment with GSH, supporting the pivotal role of GSH metabolism in disulfidptosis regulation (Figure 8U,V). Taken together, GH sensitizes HCC cells to disulfidptosis via NRF2‐SLC7A11 signaling pathway, and disturbance of GSH synthesis and depletion of NADPH could be considered as the underlying metabolic mechanisms.

Our current work indicated that GH disrupted cellular GSH synthesis and NADPH metabolism, which are central to maintain homeostasis of internal environment in both cancer and non‐cancer cells. Therefore, the influence of GH on non‐cancer cells was tested as well. Herein, mouse liver cell line, BNL CL.2, and primary mouse embryonic fibroblasts were used in our disulfidptosis model. Our results showed that GH activated NRF2‐SLC7A11 signaling effectively in those cells (Figure S6A, Supporting Information). However, it seems that both liver cells and fibroblasts are more resistant to glucose starvation‐induced disulfidptosis compared with liver cancer cells, and no cell death could been observed after 8‐h glucose starvation in both GH‐treated and untreated groups (Figure S6B,C, Supporting Information). GH treatment did not enhanced the sensitivity of those normal cells to disulfidptosis even under glucose starvation condition for 16 h (Figure S6B–G, Supporting Information). We also analyzed the influence of GH treatment on GSH synthesis and NADPH metabolism in normal cells, and the results indicated that GH treatment could affect GSH and NADPH metabolism modestly, which might not be enough to modulate disulfidptosis (Figure S6H–M, Supporting Information). Therefore, it is possible that normal cells are more resistant to disulfidptosis and GH treatment than cancer cells, in which the special metabolic reprogramming of cancer cells could play a significant role, and this hypothesis still needs further investigation in vitro and in vivo.

## 3 Discussion

The transcription factor NRF2 has been identified as one of the most important regulators in oxidative stress and cellular metabolism. A lot of studies demonstrate the key position of NRF2 in maintaining redox homeostasis as well as metabolic balance. Since the level of ROS is always related with disease progression, NRF2 signaling pathway is closely associated with the development and treatment of multiple diseases, such as tumor, neurological disorders and cardiovascular diseases. An extensive panel of antioxidant enzyme genes are considered as NRF2 targeted genes, and the activation of NRF2 is regarded as an effective strategy against oxidative stress.^[^ ^11^, ^12^ ^]^ Consequently, NRF2 activators, such as SFN and Bixin, exhibit great therapeutic action against various oxidative stress‐related diseases.^[^ ^13^ ^]^ In contrast, accumulating evidences indicate that NRF2 signaling pathway acts as a functional driver of cancer progression and metastasis, and the activation of NRF2 and its target genes promote drug resistance to chemotherapy in cancer treatment. Therefore, the suppression of NRF2 signaling pathway holds great value in the treatment of drug‐resistant cancer.^[^ ^14^ ^]^ Our previous results indicated that the combination of NRF2 inhibitor (brusatol) and erastin exhibited more effective therapeutic action against lung cancer compared with single treatment in vitro and in vivo.^[^ ^15^ ^]^ In addition, *NRF2* knockout leads to the blockage of ferritinophagy and apoferritin accumulation in the autophagosome, which enhances the intracellular labile iron pool and promotes ferroptosis, signifying the importance of NRF2 suppression in cancer treatment.^[^ ^16^ ^]^ Herein, our omics assay highlighted the promising potential of GH in modulating ferroptosis. To further confirm the hypothesis, we examined the effect of GH on HCC ferroptosis. However, the results indicated that GH could not regulate the sensitivity of HCC cells to ferroptosis, even though serving as a functional NRF2 activator (Figure S1A–H, Supporting Information). Several NRF2 target genes (such as *AKR1C1*, *HMOX1*, *GPX4*, and *SLC7A11*) related with ferroptosis were upregulated by GH treatment (Figure 5C). Among those target genes, both *GPX4* and *SLC7A11* are considered as ferroptosis suppressors. Differently, some reports suggested that HMOX1 upregulation resulted in an increase in the labile iron pool, which promoted cellular lipid peroxidation, and increased the sensitivity to ferroptosis.^[^ ^17^ ^]^ Therefore, the influence of NRF2 activator on ferroptosis should be determined by the activation manner of different NRF2 target genes. It is also important to investigate the accurate position of various NRF2 target genes in comprehensive network of ferroptosis. In this study, our findings indicated that natural compound‐induced NRF2 activation heightened the susceptibility of HCC to glucose starvation or compound‐induced disulfidptosis via regulating the transcription of SLC7A11. *NRF2* knockout suppressed disulfidptosis of HCC cells effectively, revealing an opposite role of NRF2 in cancer treatment. Therefore, NRF2 activators also hold great potential in cancer chemotherapy via regulating disulfidptosis response. However, the effective disulfidptosis activators are still limited currently. The discovery of novel disulfidptosis inducers via high‐throughput screening is essential in the future.

KEAP1‐CUL3 mediated ubiquitination degradation pathway is regarded as the most important regulatory mechanism of NRF2 protein, in which the E3 ligase adaptor KEAP1 acts as the principal negative regulator. The binding between NRF2 and KEAP1 results in the ubiquitination and degradation of NRF2 protein. However, the binding will be disturbed by some chemical stimuli or high levels of reactive oxygen species exposure, leading to the canonical activation of NRF2 signaling pathway and elimination of oxidative stress.^[^ ^18^ ^]^ Moreover, NRF2 signaling can also be activated through an autophagy‐mediated non‐canonical mechanism. For example, the blockage of autophagy leads to the accumulation of p62, which is a selective autophagy substrate, and p62 is able to compete with NRF2 for the binding to KEAP1.^[^ ^19^ ^]^ Therefore, the upregulation of p62 suppresses the ubiquitination and degradation of NRF2 and promotes the transcription of NRF2 target genes. In our study, our results indicated that GH treatment increased the protein levels of both p62 and LC3 in HCC cells. More importantly, the autophagic flux blockage in HCC cells was induced by GH treatment (Figure 5). Thus, the upregulation of p62 and LC3 was mainly due to GH‐induced blockage of autophagic flux, contributing to non‐canonical NRF2 activation in HCC cells. Besides, some studies indicated that the close relationship between autophagy and glucose starvation. For example, the core autophagy signal, AMPK pathway, can be activated by glucose deprivation via regulating the ratio of AMP or ADP level to ATP level.^[^ ^20^ ^]^ Moreover, the accumulation of DEP domain‐containing MTOR‐interacting protein (DEPTOR) can be induced by glucose starvation, and the upregulated DEPTOR binds and inhibits both MTORC1 and MTORC2, and promote cell autophagy process.^[^ ^21^ ^]^ On the other hand, the glucose metabolism and energy status are also regulated by autophagy. Some researchers have found that autophagy directly regulates the degradation of glycogen, in which the starch‐binding domain‐containing protein 1 (STBD1) links glycogen to autophagosome membrane for lysosomal degradation eventually.^[^ ^22^ ^]^ Therefore, it is also possible that GH affects glucose starvation‐induced disulfidptosis via regulating autophagy directly, although the accurate association between autophagy and disulfidptosis still requires investigation intensively.

In our current model, GH is identified as an activator of NRF2‐SLC7A11 signaling pathway. Moreover, glucose is the major source of NADPH, which is essential during the process of converting SLC7A11‐imported cystine to cysteine. Without glucose starvation, NADPH generation is enough to maintain the conversion from cystine to cysteine. However, our results indicated that GSH synthesis was still suppressed in the in vitro model modestly, which may be due to the reduced intracellular glutamate (Figure 8M–O). In addition, we observed that glucose deprivation resulted in a more pronounced increase in cysteine and reduction in glutamate as compared with the normal glucose condition (Figure 8M,N), and GH disturbed the GSH‐GSSH metabolism under glucose starvation condition more effectively than normal condition in vitro (Figure 8O–Q). It is possible glucose deprivation increased the sensitivity of HCC to some NRF2 activators. In addition, we observed that GH did not affect GSH‐GSSH metabolism without co‐treatment with BAY‐876 in vivo (Figure 8G–I). However, in the in vitro data, the treatment with GH decreased the level of GSH in normal condition (Figure 8O), even though the compound did not show significant influence on GSH/GSSH ratio (Figure 8Q). The inconsistent results could be due to only single dose of GH used in current work, and the effect of GH in gradient concentrations on GSH‐GSSH metabolism should be analyzed using multiple in vitro and in vivo models. Anyway, the detailed pharmacological mechanism and best clinical application manner still need further investigation. Moreover, to further evaluate the toxicity of GH in vivo preliminarily, different organs (liver, lung, kidney, spleen, testis, skeletal muscle and cardiac muscle) from GH‐treated and untreated mice were harvested for HE staining. We find that that no morphology change could be observed in different organs of GH‐treated group compared with normal mice (Figure S7A, Supporting Information). In addition, the activity of ALT and AST, as well as the levels of creatinine and UN in serum were measured to evaluate liver and kidney function respectively. The results further supported that GH did not cause toxicity in liver or kidney (Figure S7B–E, Supporting Information). However, the pharmacokinetics, biodistribution, as well as metabolic process of GH remain unclear, and it is essential to understand how GH is metabolized and localized in vivo, which is critical for assessing therapeutic viability and minimizing off‐target effects of GH in clinical settings.

As reported, disulfidptosis was primarily mediated by the susceptibility of the actin cytoskeleton to disulfide stress. Under the condition of glucose starvation, the glucose‐oxidizing pathway, pentose phosphate pathway (PPP) is influenced, leading to a vital reduction of NADPH during the conversion from SLC7A11‐imported cystine to cysteine. Therefore, the upregulation of SLC7A11 strengthens intracellular NADPH depletion along with excessive accumulation of cysteine or other disulfide molecules, resulting in the formation of abnormal disulfide bond in actin cytoskeleton protein as well as disulfidptosis eventually.^[^ ^1^, ^23^ ^]^ However, as the downstream of System Xc^−^, the position of GSH metabolism is still unclear in disulfidptosis model. Our current work indicated that the GSH synthesis was blocked in disulfidptosis, which could be further suppressed by GH treatment. The administration of GSH, a functional reducing agent, could protect cells from glucose starvation‐induced disulfidptosis (Figure 8), supporting the critical role of GSH in the regulation of disulfidptosis in vitro and in vivo. Some studies have indicated the function of GSH in disulfide bond formation. For example, GSH contributes to the reduction of interchain disulfide bond in recombinant monoclonal antibody during bioprocessing.^[^ ^24^ ^]^ Moreover, the disulfide bond of IgG4 heavy chain can be disrupted by GH treatment, which improve the function of IgG4 in Fc‐Fc reaction to immobilized IgG subtypes.^[^ ^25^ ^]^ Recently, some scientists discovered that GSH controlled the properties of myofibrillar protein gels via modulating the formation of disulfide bonds in a temperature‐dependent manner.^[^ ^26^ ^]^ Our work further confirmed the function of GSH on disulfide bond formation in actin cytoskeleton protein, and the suppression of GSH synthesis could be another important underlying mechanism in disulfidptosis model. Therefore, functional inhibitors of GSH synthesis hold promising potential in the development of novel disulfidptosis inducers.

Some researchers have performed CRISPR screens and the results indicated that the WAVE regulatory complex, which promotes actin polymerization and lamellipodia formation, are closely associated with disulfidptosis regulation. As the report, the effect of GH on transcription of disulfidptosis‐related genes (*SLC7A11*, *SLC3A2*, *RPN1*, *NCKAP1*, *NUBPL*, *NDUFA11*, *LRPPRC*, *OXSM*, *NDUFS1*, *GYS1*, *GSR*) were further tested in our work, and the results indicated that the transcription of most genes could be activated by GH treatment. Among these genes, the transcription of *SLC7A11* showed the most pronounced increase after GH treatment (Figure S4A, Supporting Information), indicating the important position of NRF2‐SLC7A11 signaling pathway in the function of GH on disulfidptosis regulation. However, it is still possible that multiple pathways are involved in the pharmacological function of GH on disulfidptosis regulation based on the compensatory mechanisms in cancer biology. For example, our network pharmacology assay indicated the potential of GH in mTOR signal regulation (Figure 1G), which is closely related with NRF2 activation through regulating autophagy. The mTOR signaling pathway could be another significant pharmacological mechanism for GH activity. In addition, the close association between disulfidptosis and glucose/energy metabolism has been confirmed in vitro and in vivo as reports. Our results also indicated the effect of GH on NADP^+^‐NADPH metabolism (Figures 3 and 8). Accordingly, we further tested the action of GH on transcription of some key enzyme during the process of glycolysis and pentose phosphate pathway (PPP), such as Phosphofructokinase 1 (PFK1), Phosphoglycerate kinase 1 (PGK1), glucose‐6‐phosphate isomerase (GPI) and glucose‐6‐phosphate dehydrogenease (G6PD). We found that GH treatment promoted the transcription of *PFK1* and *G6PD* in wild type cells, and the effect could be blocked in *NRF2 knock*out cells in some degree (Figure S8A–D, Supporting Information). Some studies have revealed that NRF2 acts as the upstream of G6PD and regulate the transcription directly.^[^ ^27^ ^]^ However, the relationship between NRF2 and PFK1 remains unclear for us. Nonetheless, it is possible that chemical compound‐induced NRF2 activation regulates disulfidptosis via modulating glucose/energy metabolism directly. Therefore, the accurate network underlying the influence of GH on cancer cell disulfidptosis still requires intensive investigation via high‐throughput screening and multi‐omic analyses.

In conclusion, our current work primarily exhibited that GH, a naturally occurring small molecular compound from *Garcinia oligantha Merr*., enhances the sensitivity of HCC cells to disulfidptosis, but not ferroptosis, via regulating NRF2‐SLC7A11 signaling pathway. Specifically, GH significantly increased the protein levels of NRF2 and promoted the transcription of NRF2 target gene, SLC7A11, through an autophagy‐mediated non‐canonical mechanism. The activation of NRF2‐SLC7A11 aggravated the uptake of cysteine, disturbance of GSH synthesis, and depletion of NADPH, which promoted the formation of disulfide bonds between different cytoskeleton proteins as well as disulfidptosis in HCC cells. Taken together, our study revealed the novel significance of NRF2 activation and GSH synthesis in cancer treatment, and pharmacological targeting of NRF2‐SLC7A11 signaling pathway could be considered as promising avenue for the treatment of drug‐resistant HCC in clinical settings, offering a novel insight into the precise cancer treatment via regulating disulfidptosis.

## 4 Experimental Section

### Chemicals and Cell Culture

Erastin (HY‐15763), RSL3 (HY‐100218A), Chloroquine (HY‐17589A), BAY‐876 (HY‐100017) were sourced from MCE. For this experiments, Gaudichaudione H (GH) was obtained from Key Laboratory of Chemical Biology (Ministry of Education), School of Pharmaceutical Sciences, Shandong University, The purification of GH has been described in the previous report.^[^ ^28^ ^]^ In addition, human hepatocellular carcinoma cell lines, MHCC97‐H was acquired from the China Center for Type Culture Collection (China), and Hep3B were obtained from the American Type Culture Collection (ATCC, USA), while BEL‐7404 and BEL‐7402 were procured from the Cell Bank of Chinese Academy of Sciences (China). Mouse liver cell line, BNL CL.2, and mouse embryonic fibroblasts were purchased from the National Collection of Authenticated Cell Cultures (China). Every cell line was kept at 37 °C in a humidified environment with 5% CO_2_ in DMEM (High Glucose) supplemented with 10% FBS, 100 U mL^−1^ penicillin, and 0.1g mL^−1^ streptomycin. Cells were passaged approximately every 3 to 4 days, and the culture media were changed every 2 days.

Herein, both *NRF2* and *KEAP1* knockout models were created using CRISPR/Cas9 technology following the procedures described in this previous work,^[^ ^11^, ^15^, ^29^ ^]^ and siRNA targeting SLC7A11 was used to induce gene knockdown as previous study.^[^ ^30^ ^]^

### Cell Viability Assay

In this work, samples were treated with propidium iodide (PI) staining solution (YEASEN) and incubated for 15 min in order to assess cell death across the various experimental groups. They were then washed three times using phosphate‐buffered saline (PBS). Finally, the stained cells from each group were analyzed using a fluorescence microscope (Zeiss). Additionally, Crystal Violet Assay was used to determine cell viability in different groups. In brief, the cell samples were incubated with crystal violet staining solution (Beyotine) for 10 min, and washed three times using PBS. The images of stained cells in different groups were captured using a scanner.

### Real‐Time qRT‐PCR

Real‐time qRT‐PCR was performed according to the previously published protocols.^[^ ^31^ ^]^ Total mRNA in different groups was extracted using TRIzol (Invitrogen), and 1 µg of total RNA was used to synthesize cDNA for real‐time qRT‐PCR. In this work, qPCR normalization was carried out using GAPDH, and each experiment was run in triplicate. The primer sequences (5’‐3’) used in qPCR are as follows:

Human‐*AKR1C1*‐F: GGAGGCCATGGAGAAGTGTA

Human‐*AKR1C1*‐R: GCACACAGGCTTGTACCTGA

Human‐*GCLM* ‐F: GACAAAACACAGTTGGAACAGC

Human‐*GCLM*‐R: CAGTCAAATCTGGTGGCATC

Human‐*NQO1*‐F: ATGTATGACAAAGGACCCTTCC

Human‐*NQO1*‐R: TCCCTTGCAGAGAGTACATGG

Human‐*HMOX1*‐F: AACTTTCAGAAGGGCCAGGT

Human‐*HMOX1*‐R: CTGGGCTCTCCTTGTTGC

Human‐*GPX4*‐F: GAGGCAAGACCGAAGTAAACTAC

Human‐*GPX4*‐R: CCGAACTGGTTACACGGGAA

Human‐*SLC7A11*‐F: TCTCCAAAGGAGGTTACCTGC

Human‐*SLC7A11*‐R: AGACTCCCCTCAGTAAAGTGAC

Human‐*RPN1*‐F: GGCCAAGATTTCAGTCATTGTGG

Human‐*RPN1*‐R: CTTCGTTGGATAGGGAGAGTAGA

Human‐*NCKAP1*‐F: TTGTACCCCATAGCAAGTCTCT

Human‐*NCKAP1*‐R: GGGCATTTCTCCACTGGTCAG

Human‐*NUBPL*‐F: CTGAGATGTTTCGCAGAGTCC

Human‐*NUBPL*‐R: CAAGGGTCTGTGCTAGTTTCC

Human‐*LRPPRC*‐F: GCTCATAGGATATGGGACACACT

Human‐*LRPPRC*‐R: CCAGGAAATCAGTTGGTGAGAAT

Human‐*OXSM*‐F: CAATATCCAGATTGCATAGGCGA

Human‐*OXSM*‐R: CGATCCCAAACCAGGTGAGTT

Human‐*GYS1*‐F: GCGCTCACGTCTTCACTACTG

Human‐*GYS1*‐R: TCCAGATGCCCATAAAAATGGC

Human‐*SLC3A2*‐F: TGAATGAGTTAGAGCCCGAGA

Human‐*SLC3A2*‐R: GTCTTCCGCCACCTTGATCTT

Human‐*NDUFA11*‐F: TTGAAGGAGTGGCTAAGGTTGG

Human‐*NDUFA11*‐R: CACCGAGGAAGTAGTTCAGGG

Human‐*NDUFS*1‐F: TTAGCAAATCACCCATTGGACTG

Human‐*NDUFS1*‐R: CCCCTCTAAAAATCGGCTCCTA

Human‐*GSR*‐F: AGGCTTCCTGCTGCTTCTG

Human‐*GSR*‐R: GTAGTCATAGGAGGCCACGG

Human‐*PGK1*‐F: GCTCATAAGGACTACCGACTTGG

Human‐*PGK1*‐R: TGGACGTTAAAGGGAAGCGG

Human‐*PFK1*‐F: GCTGGGCGGCACTATCATT

Human‐*PFK1*‐R: TCAGGTGCGAGTAGGTCCG

Human‐*GPI*‐R: CCAGGATGGGTGTGTTTGACC

Human*‐GPI*‐F: CAAGGACCGCTTCAACCACTT

Human‐*GAPDH*‐F: CTGACTTCAACAGCGACACC

Human‐*GAPDH*‐R: TGCTGTAGCCAAATTCGTTGT

### Western Blot

Herein, western blot assay was conducted following the previous work.^[^ ^11^, ^13^, ^32^ ^]^ Briefly, protein samples (20 µg per lane) were separated on 8% or 12% SDS‐PAGE gels and subsequently transferred to PVDF membranes. The membranes were blocked using 5% Nonfat‐Dried Milk, and incubated with different primary antibodies at 4 °C overnight. In this study, the primary antibodies employed were as follows: anti‐SLC7A11 (1:1000; Proteintech, 26864‐1‐AP), anti‐p62 (1:1000; Proteintech, 18420‐1‐AP), anti‐LC3 (1:1000; Proteintech, 14600‐1‐AP), anti‐NRF2 (1:1000; Proteintech, 16396‐1‐AP), anti‐KEAP1 (1:1000; Proteintech, 10503‐2‐AP) and anti‐GAPDH (1:3000; Proteintech, 10494‐1‐AP). After being washed with PBS, membranes were further incubated with HRP‐labeled secondary antibodies (anti‐mouse IgG (1:3000; ABclonal, AS003) and anti‐rabbit IgG (1:3000; ABclonal, AS014)) for 1 h at room temperature, and the protein bands were visualized using an enhanced chemiluminescence kit (SuperSignal West Femto Maximum Sensitivity Substrate, Thermo Fisher) and ChemiDoc Imagers (Bio‐Rad Laboratories, USA).

### Evaluation of Malondialdehyde (MDA) and 4‐Hydroxynonenal (4‐HNE)

To evaluate ferroptosis across various groups, the levels of MDA and 4‐HNE were measured in this work. In this context, cell sample was prepared using the Lipid Peroxidation (MDA) Assay Kit (MAK085, Sigma–Aldrich, USA) and the Lipid Peroxidation (4‐HNE) Assay Kit (ab238538, Abcam, USA), and the MDA and 4‐HNE concentrations within cell lysates in each group were analyzed according to the manufacturer's instructions.

### RNA Sequencing

In the current work, total RNA was extracted from both GH‐treated and GH‐untreated cell replicates using TRIzol reagent. Then, mRNA was isolated from total RNA using poly‐T oligo‐attached magnetic beads. Sequencing libraries were generated for each experimental group following the manufacturer's protocol outlined in the NEBNext Ultra RNA Library Prep Kit for Illumina (NEB). These libraries underwent sequencing on an Illumina Novaseq 6000 platform provided by Novogene Beijing, China.

### Network Pharmacology Analysis

The potential bioavailable targets of GH were predicted utilizing data from the SwissTargetPrediction database^[^ ^33^ ^]^ and TargetNet^[^ ^34^ ^]^ based on its chemical structure. The overlap targets from the two databases were selected for cluster analysis using Metascape.^[^ ^35^ ^]^ Furthermore, a summary of genes and variants associated with HCC is available in The Comparative Toxicogenomics Database,^[^ ^36^ ^]^ and the potential bioavailable targets of GH related with HCC progression and treatment were subsequently summarized. Herein, Protein–Protein Interaction Networks were created using STING database.^[^ ^37^ ^]^

### Metabolomics

In this study, both GH‐treated and GH‐untreated HCC cells were prepared for Metabolomics assay. In brief, the sample for metabolomics assay were prepared by PANOMIX Biomedical Tech Co., LTD in Suzhou, China. The final extract from each group was analyzed using Ultra‐high‐performance liquid chromatography‐tandem mass spectrometry (UPLC‐MS/MS). Subsequently, the raw data from UPLC‐MS/MS assay were converted to mzXML format using MSConvert in ProteoWizard software package (v3.0.8789)^[^ ^38^ ^]^ and processed with R XCMS(v3.12.0)^[^ ^39^ ^]^ for feature detection, retention time correction, and alignment. Next, both metabolomics data analysis and pathway analysis were performed using MetaboAnalyst and R ropls (v1.22.0) package respectively.^[^ ^40^ ^]^

### Live Cell Immunofluorescence Microscopy

To evaluate the effect of GH on autophagic flux, the ptf‐LC3 vector (mRFP‐GFP‐LC3 reporter construct) was utilized in live cell Immunofluorescence. Following transfection of MHCC97‐H cells with ptf‐LC3 vectors for 16 h using Lipofectamine 3000 solution (Thermo Fisher Scientific), the cells were treated with GH for a duration of 24 h. Subsequently, both treated and untreated cell samples underwent imaging via a Zeiss fluorescence microscope.

### Cytoskeleton Staining

In this work, Actin‐Tracker Red‐Rhodamine was applied for cytoskeleton staining. First, the cell samples in different groups were fixed with 3.7% paraformaldehyde solution at room temperature for 20 min and washed with PBS containing 0.1% Triton X‐100 for three times. Subsequently, cytoskeleton structure was stained using Actin‐Tracker Red‐Rhodamine at a ratio of 1:200 at room temperature for 30 min. After washed with PBS for three times, the samples were observed directly with a fluorescence microscope (Zeiss).

### Analysis of Glutamate, Cysteine, and Glycine

The levels of glutamate, cysteine, and glycine were tested in this work to evaluate the function of system Xc^−^ as well as metabolism of amino acid. All cell or tissue samples were prepared using Glutamate Assay Kit (Sangon Biotech, D799585‐0050), Cysteine Assay Kit (Solarbio, BC0185), and Glycine Assay Kit (ELK Biotechnology, ELK8276). The levels of glutamate, cysteine, and glycine in various groups were determined according to the manufacturer's instructions finally.

### Evaluation of GSH Metabolism and NADP^+^‐NADPH Metabolism

To evaluate GSH metabolism and NADP^+^‐NADPH metabolism across various groups, the levels of GSH/GSSH and NADP^+^/NADPH were measured in this work. For this purpose, cell samples or tissue samples were prepared using the GSH and GSSG Assay Kit (Beyotime Biotechnology, S0053) and NADP^+^/NADPH Assay Kit (Beyotime Biotechnology, S0180S). The level of GSH/GSSH and NADP^+^/NADPH in different groups were calculated according to the manufacturer's instructions.

### Animal Studies

All animal experiments were approved by The Biomedical Ethics Committee of Health Science Center of Xi'an Jiaotong University (Approval number: 2022‐1371) on June 9th, 2022. First, the toxicity of GH (1 mg kg^−1^, twice weekly for 6 weeks) in different organs (liver, lung, kidney, spleen, testis, skeletal muscle, and cardiac muscle) of C57BL/6J mice was evaluated in vivo preliminarily. The tissues from GH‐treated and untreated mice were harvested for HE staining. In addition, to evaluate the effect of GH on liver and kidney function, the activity of alanine aminotransferase (ALT) and aspartate transferase (AST), as well as the levels of creatinine and urea nitrogen (UN) in serum were measured using ALT Activity Assay Kit (Solarbio, BC1555), AST Activity Assay Kit (Solarbio, BC1565), Creatinine Colorimetric Assay Kit (Elabscience, E‐BC‐K188‐M) and UN Colorimetric Assay Kit (Solarbio, BC1535). Besides, SCID Beige Mice, sourced from Charles River Laboratories, were used for xenograft mouse model in the study. Herein, MHCC97‐H cells were used for the xenograft, and the 8‐week‐old male mice (weight = 22–24g) were inoculated with cancer cell suspension containing 5 × 10^6^ cells per mouse (i.h.). When the tumor volume was ≈50–100 mm^3^, the mice were categorized into four groups: Ctrl, GH, BAY‐876, and BAY‐876+GH. Tumor dimensions were determined using a vernier caliper, and the tumor volume was calculated as: Volume = π/6 × Length × Width^2^. In this work, both GH (1 mg kg^−1^) and BAY‐876 (3 mg kg^−1^) were prepared in a 5% DMSO/corn oil solution and administered intraperitoneally to the mice twice weekly for a duration of 6 weeks. Following this treatment period, the mice were euthanized, and the tumor weight and metabolism were evaluated in different groups (*n* = 5).

### Statistical Analysis

In this current study, data are presented as mean ± SD. Statistical analysis was conducted with SPSS version 17.0 software package. Unpaired Student's *t*‐tests were applied to compare two distinct groups whereas one‐way ANOVA followed by Bonferroni's post‐hoc correction was used to compare three or more groups. The Student's *t*‐test was performed as a one‐tailed test and the significance level set at a *p*‐value of less than 0.05.

## Conflict of Interest

The authors declare no conflict of interest.

## Author Contributions

M.S. performed formal analysis, funding acquisition, investigation, methodology, and writing of the original draft. X.L. performed formal analysis and investigation. Y.G. performed investigation and methodology. Y.Z. performed investigation. J.X. performed formal analysis and investigation. L.Y. performed investigation. R.L. performed investigation and provided resources. H.W. performed investigation. S.T. performed investigation and provided resources. Y.Z. performed investigation. Z.L. supervised and reviewed & edited the writing. Y.F. performed investigation, supervised, acquired funding, and reviewed & edited the writing. D.R. acquired funding, supervised, and reviewed & edited the writing. P.L. performed formal analysis, acquired funding, investigated, supervised, wrote the original draft, and reviewed & edited the writing.

## Supporting information



Supporting Information

## Data Availability

The data that support the findings of this study are available from the corresponding author upon reasonable request
