# Supplementary material for: Gaudichaudione H Enhances the Sensitivity of Hepatocellular Carcinoma Cells to Disulfidptosis via Regulating NRF2‐SLC7A11 Signaling Pathway
Source: Adv Sci (Weinh). 2025 Jan 22;12(11):2411131. doi: 10.1002/advs.202411131 (PMC11923960; doi:10.1002/advs.202411131)
Supplement: Supplementary file 1 — Supporting Information [file ADVS-12-2411131-s001.pdf]

ADVANCED SCIENCE
Open Access

Supporting Information

for *Adv. Sci.*, DOI 10.1002/advs.202411131

Gaudichaudione H Enhances the Sensitivity of Hepatocellular Carcinoma Cells to Disulfidptosis via Regulating NRF2-SLC7A11 Signaling Pathway

Mengjiao Shi, Xinyan Li, Ying Guo, Yinggang Zhang, Jiayi Xu, Liangwen Yan, Rongrong Liu, Hong Wang, Shenkang Tang, Yaping Zhao, Zongfang Li, Yetong Feng, Dongmei Ren* and Pengfei Liu**

Supplementary Materials for

Gaudichaudione H enhances the sensitivity of hepatocellular carcinoma cells to disulfidptosis via regulating NRF2-SLC7A11 signaling pathway

Mengjiao Shi [1,2,3], Xinyan Li [2], Ying Guo [2,3], Yinggang Zhang [2], Jiayi Xu [2], Liangwen Yan [2], Rongrong Liu [2,3], Hong Wang [2,3], Shenkang Tang [2,4], Yaping Zhao [2], Zongfang Li [1,3], Yetong Feng [2,5]*, Dongmei Ren [2,6]*, Pengfei Liu [2,3,7]*

1. Department of General Surgery, National & Local Joint Engineering Research Center of Biodiagnosis and Biotherapy, The Second Affiliated Hospital of Xi’an Jiaotong University, Xi’an, China
2. International Joint Research Center on Cell Stress and Disease Diagnosis and Therapy, National & Local Joint Engineering Research Center of Biodiagnosis and Biotherapy, The Second Affiliated Hospital of Xi’an Jiaotong University, Xi’an, China
3. Shaanxi Provincial Clinical Research Center for Hepatic & Splenic Diseases, The Second Affiliated Hospital of Xi’an Jiaotong University, Xi’an, China
4. Department of Oncology, Affiliated Hospital of Shaanxi University of Chinese Medicine, Xianyang, China
5. Core Research Laboratory, The Second Affiliated Hospital of Xi'an Jiaotong University, Xi'an, China
6. Key Laboratory of Chemical Biology (Ministry of Education), School of Pharmaceutical Sciences, Shandong University, Jinan, China
7. Key Laboratory of Environment and Genes Related To Diseases, Xi'an Jiaotong University, Ministry of Education of China, Xi'an, China.

* Corresponding author:
Pengfei Liu, E-mail address: liupengfei@xjtu.edu.cn
Dongmei Ren, Email: rendom@sdu.edu.cn
Yetong Feng, E-mail address: fengyetong@ xjtu.edu.cn

Table S1. The potential bioavailable targets of GH predicted by SwissTargetPrediction and TargetNet

Table S2. The genes and variants associated to hepatocellular carcinoma in The DisGeNET database

Table S3. The secondary metabolites in metabolomics analysis

Table S4. The differentially expressed gene list in RNA-seq assay

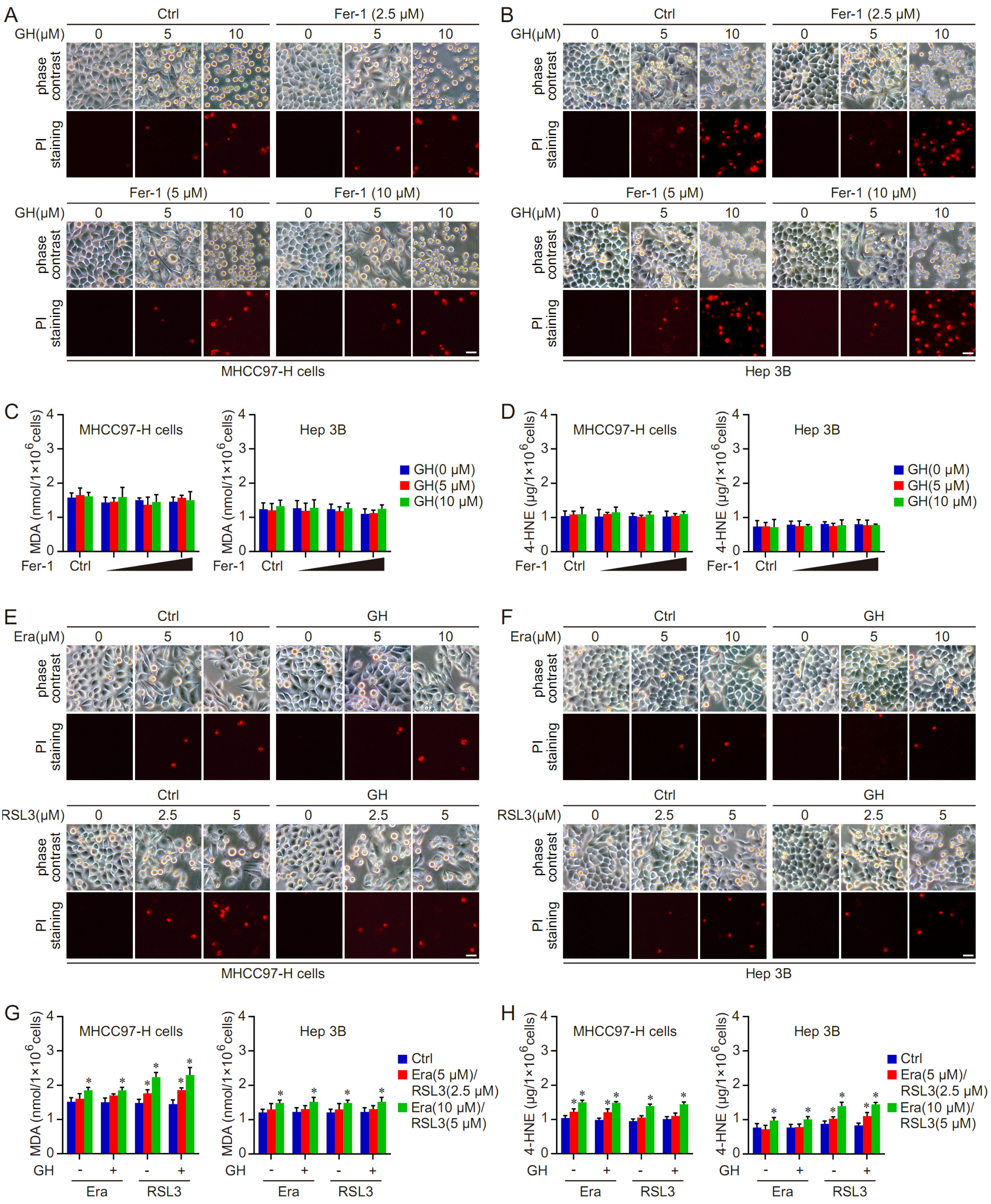

Figure S1. GH dosen't affect the sensitivity of HCC cells to ferroptosis. Herein, both MHCC97-H cells and Hep 3B cells were treated with GH in different concentrations (5 μM and 10 μM) for 16 hours. To evaluate ferroptosis in each group, the cells were co-treated with ferroptosis inhibitor Ferrostatin-1 (Fer-1, 2.5 μM, 5 μM and 10 μM), and the morphology change and cellular toxicity caused by GH treatment were observed first (A-B). To confirm

the position of ferroptosis, the levels of both MDA and 4-HNE were respectively determined in our work (C-D). In addition, the effect of GH on the sensitivity of HCC cells to erastin or RSL3-induced ferroptosis was evaluated in our work (E-F), and the levels of both MDA and 4-HNE were measured in each group (G-H). (Data were presented as means ± SD. *: $P<0.05$ compared with Ctrl group; Scale bar=20 μm)

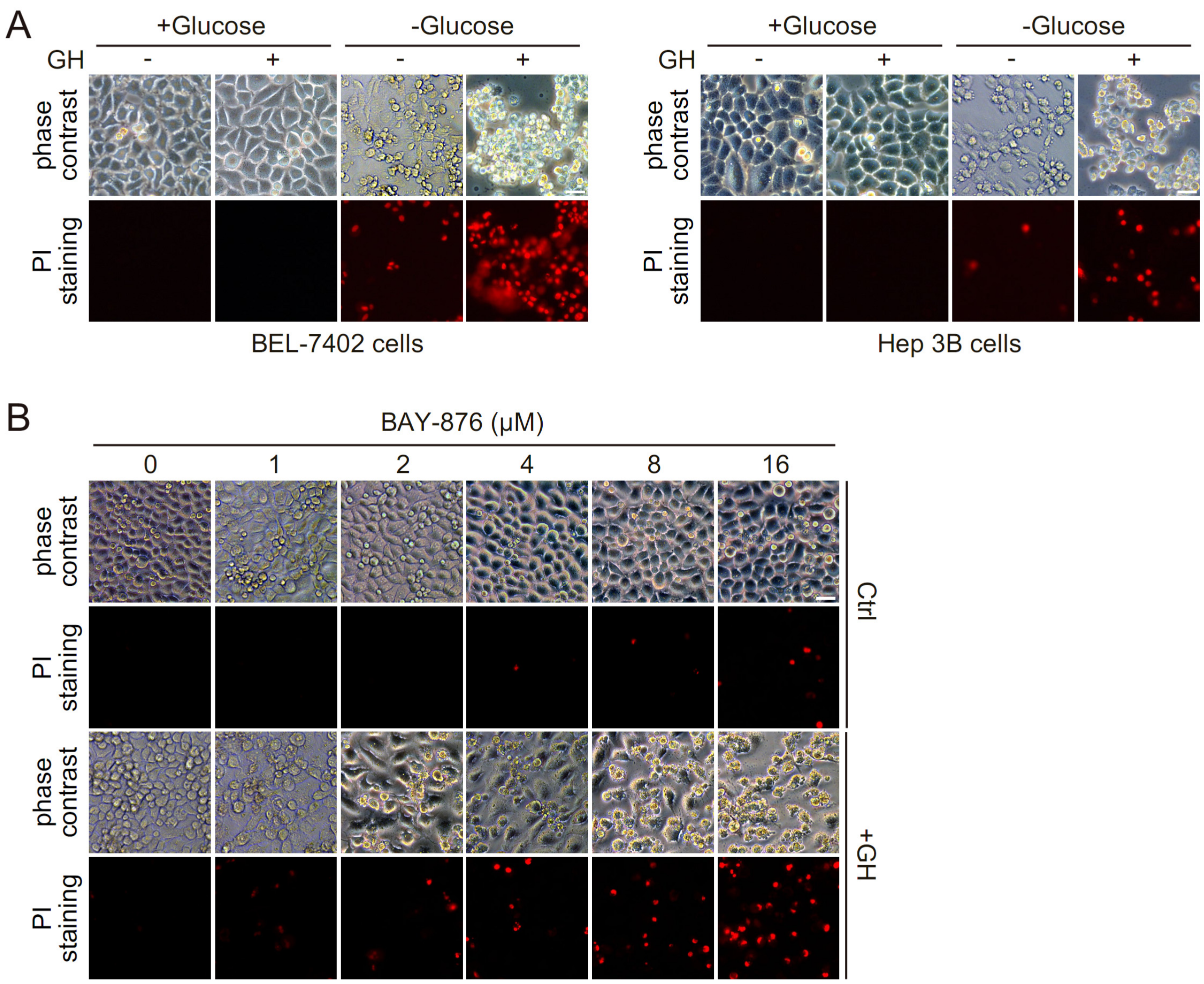

Figure S2. GH enhances the sensitivity of HCC cells to disulfidptosis. Herein, BEL-7402 and Hep 3B cells were treated with GH (2 μM) for 16 hours firstly, followed with glucose starvation (6-8 hours) to induce disulfidptosis. Then the morphology change and cell death were evaluated in each group (A). Besides, the GLUT1 inhibitor BAY-876 in various concentrations were used to induce disulfidptosis in HCC cells (MHCC97-H cells). The effect of GH pre-treatment on the sensitivity of HCC cells to BAY-876-induced disulfidptosis was determined eventually (B). (Scale bar=20 μm)

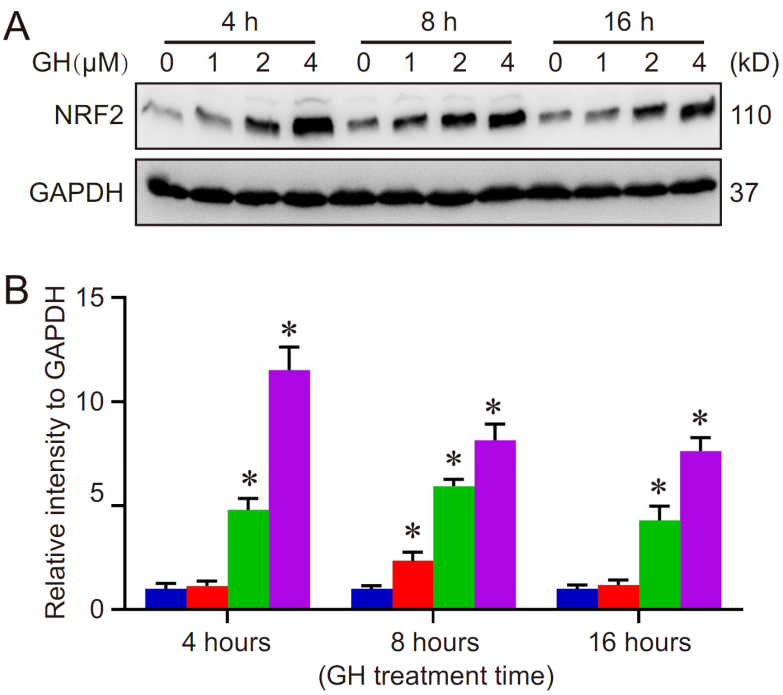

Figure S3. The effect of GH treatment on NRF2 activation. MHCC97-H cells were treated with GH in different concentrations (1 μM, 2 μM and 4 μM), then the cells were harvested for western blot (A) in multiple time points (4h, 8h and 16h). The relative intensity to GAPDH in each group was calculated in our work (B). (Data were presented as means ± SD. *: $P<0.05$ compared with Ctrl group)

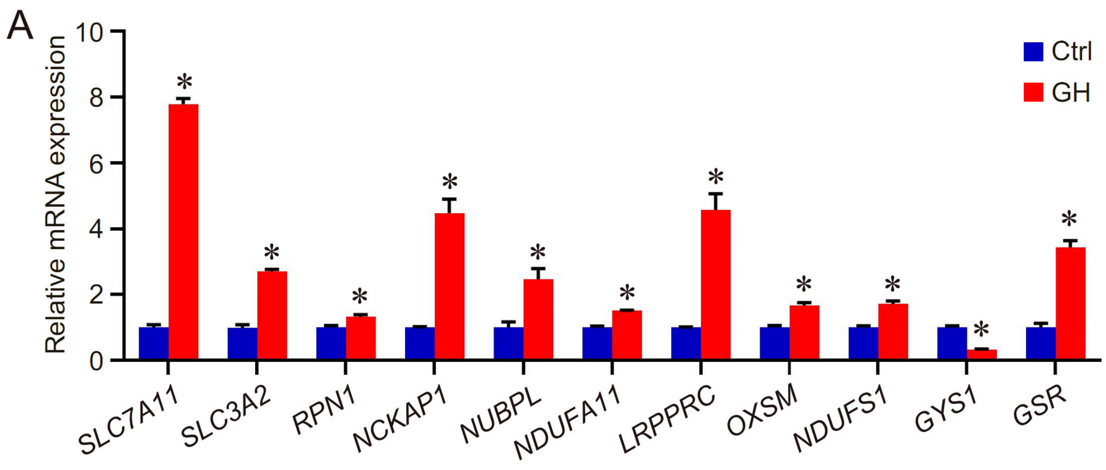

Figure S4. The effect of GH treatment on transcription of disulfidptosis-related genes. MHCC97-H cells were treated with GH (2 μM) for 16 hours, then the cells were harvested for qPCR measurement (A). (Data were presented as means ± SD. *: P<0.05 compared with Ctrl group)

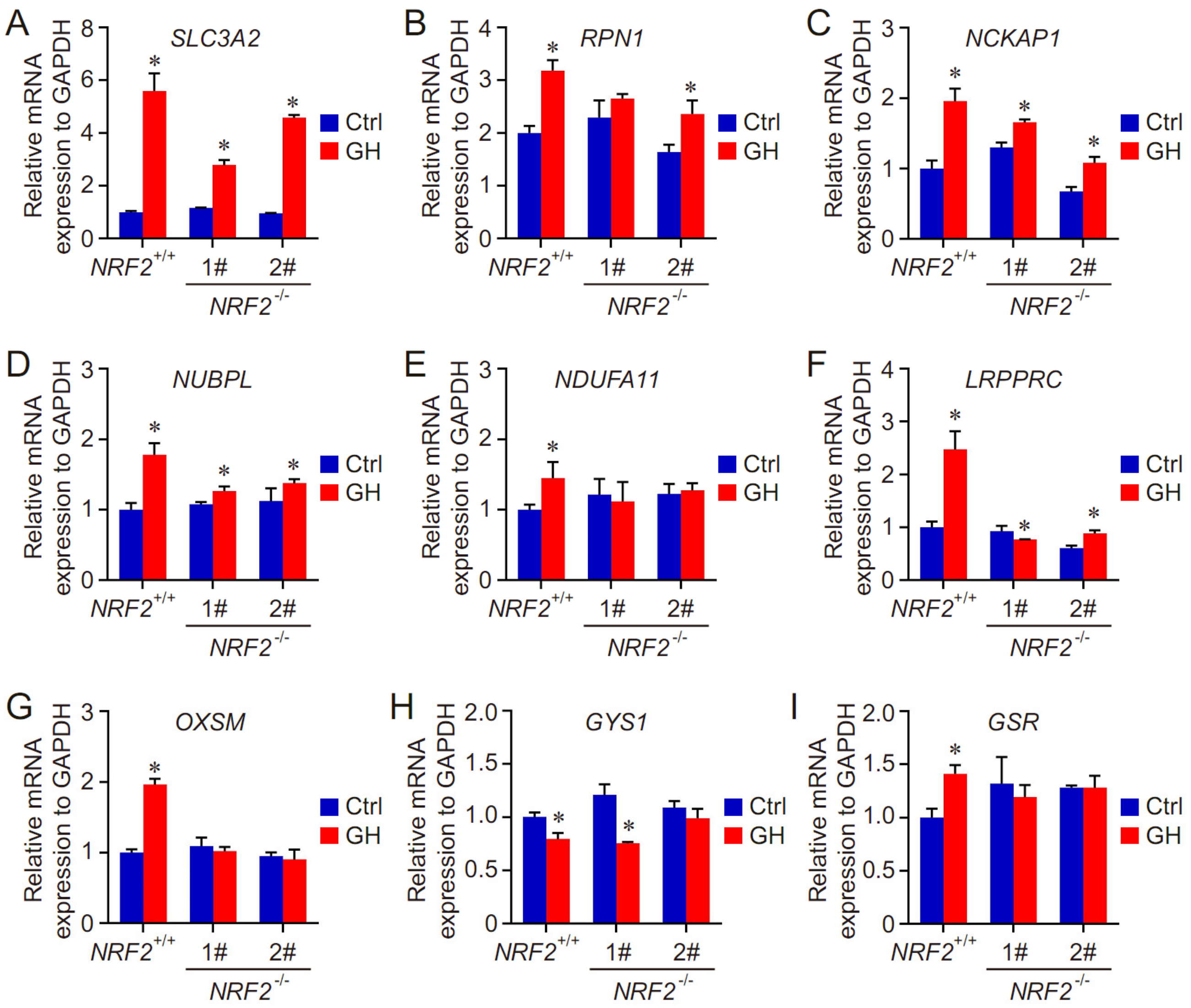

Figure S5. The effect of GH treatment on transcription of disulfidptosis-related genes in wild type and *NRF2* knockout cells. *NRF2* knockout MHCC97-H cell line was established using CRISPR/Cas9 technique. Both wild type and *NRF2* knockout cells were treated with GH (2 μM) for 16 hours, then the cells were harvested for qPCR measurement (A-I). (Data were presented as means ± SD. *: P<0.05 compared with Ctrl group)

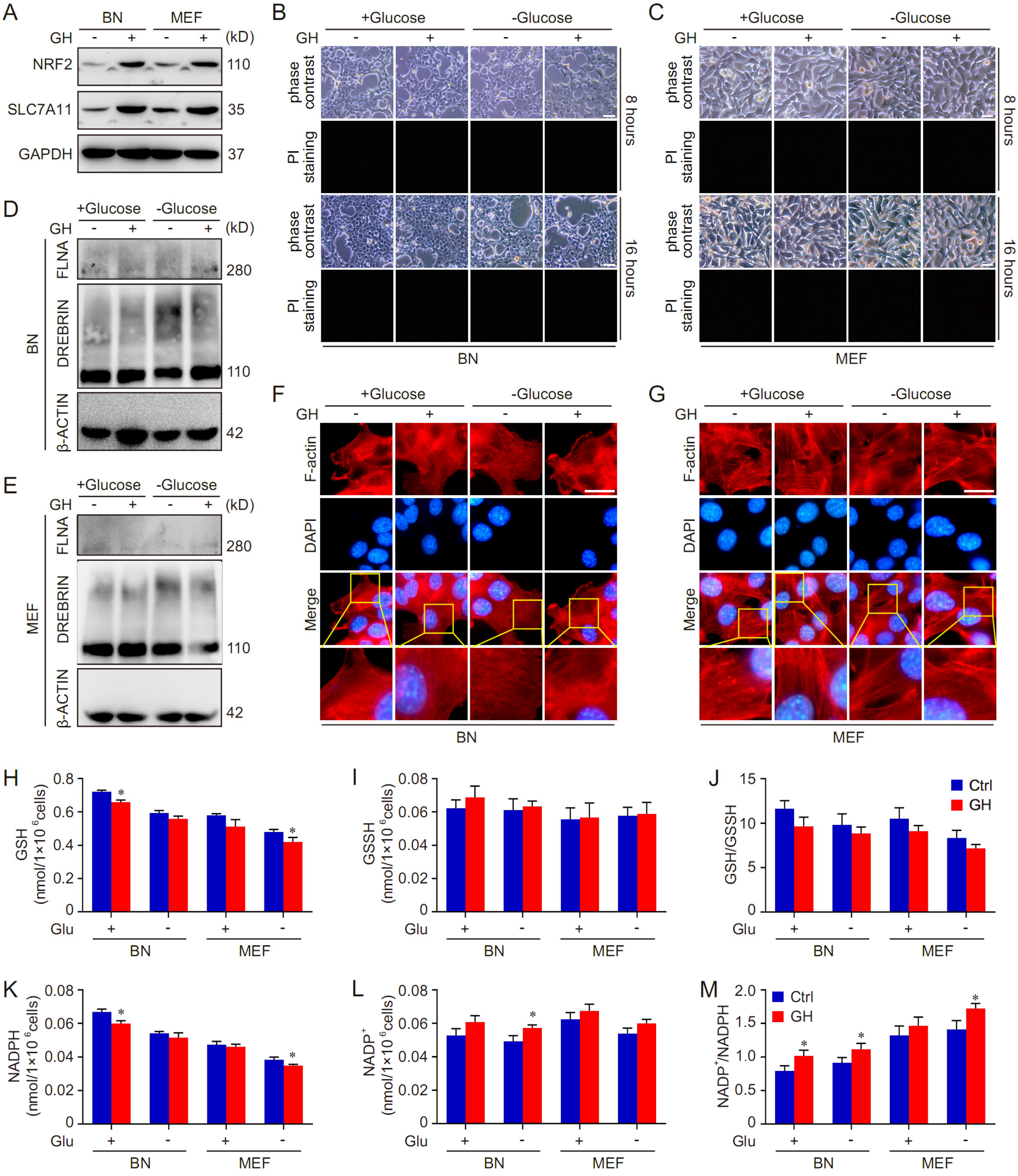

Figure S6. GH didn't affect the response of normal liver cells or fibroblasts to disulfidptosis. Herein, BNL CL.2 cells (BN) and mouse embryonic fibroblasts (MEF) were

treated with GH (2 μM) for 16 hours firstly, followed with glucose starvation (8 hours or 16 hours) to induce disulfidptosis. The effect of GH treatment on NRF2-SLC7A11 signaling pathway was tested using immunoblot (A). The morphology change and cell death were evaluated in each group (B-C, Scale bar=20 μm). In addition, the glucose starvation (16 hours)-induced disulfide bond formation in the cytoskeleton proteins (FLNA, β-ACTIN and DREBRIN) was evaluated using non-reducing western blot (D-E). Finally, phalloidin staining was applied to evaluate the actin filament (F-actin) in different groups (F-G, Scale bar=10 μm). Moreover, both GSH-GSSH metabolism (H-J) and $NADP^+$-NADPH metabolism (K-M) in different groups were evaluated respectively. (Data were presented as means ± SD. *: $P<0.05$ compared with Ctrl groups)

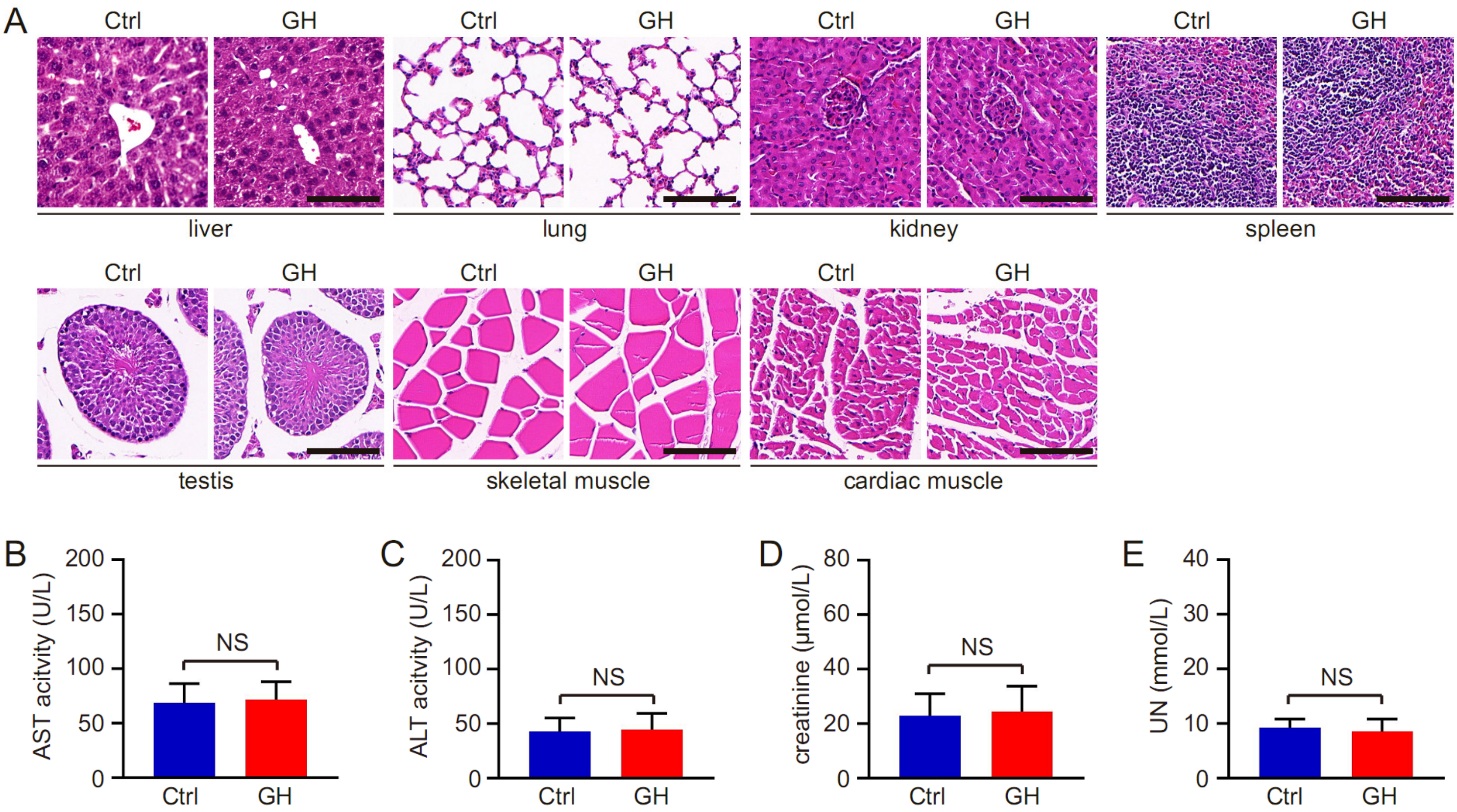

Figure S7. Evaluation of GH toxicity in different organs. Herein, the toxicity of GH (1 mg/kg, twice weekly for six weeks) in different organs (liver, lung, kidney, spleen, testes, skeletal muscle and cardiac muscle) of C57BL/6J mice was evaluated in vivo preliminarily. The tissues from GH-treated and untreated mice were harvested for HE staining (A, Scale bar=100 μm). In addition, to evaluate the effect of GH on liver and kidney function, the activity of alanine aminotransferase (ALT) and aspartate transferase (AST), as well as the levels of creatinine and urea nitrogen (UN) in serum were measured respectively (B-E). (Data were presented as means ± SD. NS: $P > 0.05$ compared with Ctrl group)

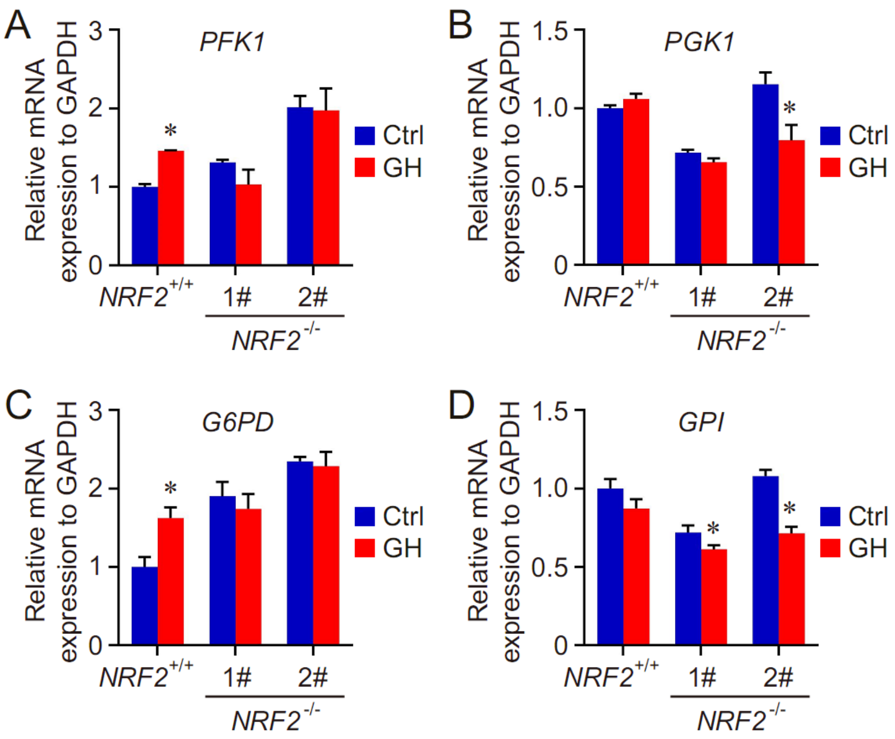

Figure S8. The effect of GH treatment on transcription of key enzymes during the process of glycolysis and pentose phosphate pathway. *NRF2* knockout MHCC97-H cell line was established using CRISPR/Cas9 technique. Both wild type and *NRF2* knockout cells were treated with GH (2 μM) for 16 hours, then the cells were harvested for qPCR measurement (A-D). (Data were presented as means ± SD. *: P<0.05 compared with Ctrl group)

Table S1. The potential bioavailable targets of GH predicted by SwissTargetPrediction and TargetNet

Database	Targets	Number
SwissTargetPrediction	IKBKB TXN TXN2 MCL1 CASP1 RET PIK3CA MAPK14 LRRK2 TNK2 HSP90AB1 MMP1 PDE4B KDM1A PDK1 WNT3A MMP3 PLA2G7 PDE10A TNKS2 CCNB3 CDK1 CCNB1 CCNB2 CDK2 CCNA1 CCNA2 MMP16 RPS6KA3 GSK3B MMP13 MMP15 ADAM17 MMP14 MMP7 MMP8 EIF2AK3 CSF1R NLRP3 FLT3 JAK3 JAK1 JAK2 TYK2 CASP3 CASP7 MAP3K12 HRH3 CAPN1 ADAM9 CXCR2 SYK FCER2 PARP1 CXCR1 P2RX3 IGF1R ADORA3 PIM1 ADAMTS5 ADAMTS4 CHEK1 WEE1 ADAM33 ERN1 CMA1 ABL1 CACNA1C ADORA1 SRC MTOR NQO1 PLEC LCK SCN9A ROS1 BDKRB1 PFKFB3 PIK3CB PIK3CG RORC PDE5A ITK MAPK1 MME CDK1 CCNB1 CCNE1 CDK2 CDK2 CDK1 PRKDC HDAC1 CDK4 NOS2 MYLK HTR7 MMP10 KCNA3 PSEN2 PSENEN NCSTN APH1A PSEN1 APH1B CASP8 BMP1 PIK3C2B TBXAS1 HDAC6 NCOR2 HDAC3 CAMK2D	100
TargetNet	AKT1 CRHR1 Cnr1 PTPN1 HCRTR2 PTPN22 Agtr1b ADORA3 TMIGD3 AGTR1 PTAFR Tacr1 AKR1B1 ALK NPY1R ADRA1B Ednrb PRKCE AGTR2 HMGCR PGR GHSR ADORA1 Adora2a SLC5A2 CTSS LRRK2 CNR2 AVPR1A JAK3 ERBB2 CDK4 NTRK1 PDE7A CDK5 Adora1 Hmgcr FGFR1 OPRK1 Oprm1 MTOR AKT3 Avpr2 IGF1R Crhr1 KCNA5 ampC PIK3CG ZAP70 MET MAPKAPK2 MPL BTK TGFBR1 AVPR2 BACE2 EDNRA PTGDR Ednra HCRTR1 ADORA2A ADORA2B Cnr2 TEK CDK2 FNTB HCK TYK2 CTSD DRD1 F7 Adora3 ache P2RY1 KDR EDNRB JAK2 OXTR BDKRB2 Cckbr PLK1 MAPK9 MAPK8 NR3C2 RPS6KB1 F10 SRC AVPR1B Prcp PLIN5 CXCR3 GSK3B FLT4 PIM3 CALCRL PTGER3 SLC5A1 ADAM17 PDPK1 Agtr2 NR2E3 NR3C1 PAK4 HNF4A NR1H4 CASP9 PDE4B PRKCG LYN RET BCL2A1 TOP1 PDE3A CDK1 HSP90AB1 TBXA2R AXL BDKRB1 PTPN7 Tspo LCK AKT2 EGLN1 OPRM1 BCL2 ADK HSP90AA1 PRKCD RPS6KA3 IKBKB Mapk14 ELANE FLT1 SERPINE1 PIK3CB PIK3CA PDE4D F2 CMA1 Oprk1 PDE10A HTR6 MDM2 PRKCB DNMT1 AURKA FKBP1A PIK3CD PDE4A MAPK14 PDE5A GNRHR Gnrhr CETP BCL2L1 DGAT1 BRAF FYN CASP3 CNR1 SYK PRSS1 ALOX5AP EPHB4 MCL1 PPARG MAPK10 Oprd1 KCNA3 P2RY12 PIM1 PTK2 CDK7 PIM2	174
Overlapping targets	ADAM17 ADORA1 ADORA3 BDKRB1 CASP3 CDK1 CDK2 CDK4 CMA1 GSK3B HSP90AB1 IGF1R IKBKB JAK2 JAK3 KCNA3 LCK LRRK2 MAPK14 MCL1 MTOR PDE10A PDE4B PDE5A PIK3CA PIK3CB PIK3CG PIM1 RET RPS6KA3 SRC SYK TYK2	33

Table S2. The genes and variants associated to hepatocellular carcinoma in The DisGeNET database

Disease	Disease_id	Gene	Gene_id	UniProt	Gene_Full_Name	N_diseases_g	DSI_g	DPI_g	pLI	Score_gda	EI_gda	N_PMIDs
Hepatocarcinogenesis	C1512409	AR	367	P10275	androgen receptor	854	0.351	0.846	0.98837	0.1	0.9	10
Hepatocarcinogenesis	C1512409	MIR122	406906		microRNA 122	250	0.468	0.808		0.1	1	18
Hepatocarcinogenesis	C1512409	CDKN2A	1029	P42771;Q8N726	cyclin dependent kinase inhibitor 2A	1314	0.3	0.885	0.39474	0.1	0.92	25
Hepatocarcinogenesis	C1512409	CTNNB1	1499	P35222	catenin beta 1	1368	0.303	0.885	0.99999	0.1	0.909	33
Hepatocarcinogenesis	C1512409	NFE2L2	4780	Q16236	nuclear factor, erythroid 2 like 2	823	0.357	0.885	0.00357	0.1	1	11
Hepatocarcinogenesis	C1512409	H3P10	115482713		H3 histone pseudogene 10	769	0.35	0.846		0.1	1	10
Hepatocarcinogenesis	C1512409	PPARA	5465	Q07869	peroxisome proliferator activated receptor alpha	408	0.432	0.885	0.03018	0.1	1	12
Hepatocarcinogenesis	C1512409	TGFA	7039	P01135	transforming growth factor alpha	376	0.432	0.885	0.71981	0.1	0.9	10
Hepatocarcinogenesis	C1512409	AKT1	207	P31749	AKT serine/threonine kinase 1	1250	0.311	0.962	0.9759	0.1	1	16
Hepatocarcinogenesis	C1512409	AFP	174	P02771	alpha fetoprotein	392	0.429	0.885	1.1E-11	0.1	1	13
Hepatocarcinogenesis	C1512409	TP53	7157	P04637	tumor protein p53	2494	0.236	0.962	0.53235	0.1	0.935	62
Hepatocarcinogenesis	C1512409	FGF19	9965	O95750	fibroblast growth factor 19	123	0.548	0.615	0.57022	0.08	0.75	8
Hepatocarcinogenesis	C1512409	IGF2	3481	P01344	insulin like growth factor 2	604	0.39	0.885	0.04413	0.08	1	8
Hepatocarcinogenesis	C1512409	GABPA	2551	Q06546	GA binding protein transcription factor subunit alpha	632	0.379	0.885	0.99812	0.08	1	8
Hepatocarcinogenesis	C1512409	SLCO6A1	133482	Q86UG4	solute carrier organic anion transporter family member 6A1	449	0.412	0.885	2.9E-11	0.07	1	7
Hepatocarcinogenesis	C1512409	CCND1	595	P24385	cyclin D1	859	0.344	0.923	0.89057	0.07	0.857	7
Hepatocarcinogenesis	C1512409	GSTK1	373156	Q9Y2Q3	glutathione S-transferase kappa 1	445	0.412	0.885	1.9E-06	0.07	1	7
Hepatocarcinogenesis	C1512409	DLC1	10395	Q96QB1	DLC1 Rho GTPase activating protein	144	0.529	0.731	0.99948	0.07	1	7
Hepatocarcinogenesis	C1512409	IGF1	3479	P05019	insulin like growth factor 1	1206	0.318	0.885	0.2716	0.07	1	7
Hepatocarcinogenesis	C1512409	PSMD10	5716	O75832	proteasome 26S subunit, non-ATPase 10	116	0.552	0.808	0.50136	0.07	1	7
Hepatocarcinogenesis	C1512409	NR1I3	9970	Q14994	nuclear receptor subfamily 1 group I member 3	318	0.448	0.846	3.5E-08	0.07	1	7
Hepatocarcinogenesis	C1512409	PTGS2	5743	P35354	prostaglandin-endoperoxide synthase 2	1234	0.314	0.962	0.99597	0.07	0.857	7
Hepatocarcinogenesis	C1512409	CYP2E1	1571	P05181	cytochrome P450 family 2 subfamily E member 1	306	0.459	0.885	8.1E-09	0.07	1	7
Hepatocarcinogenesis	C1512409	LOC11080626	110806263		TERT 5' regulatory region	245	0.464	0.731		0.07	1	7
Hepatocarcinogenesis	C1512409	MYC	4609	P01106	MYC proto-oncogene, bHLH transcription factor	821	0.344	0.923	0.99801	0.07	0.857	7
Hepatocarcinogenesis	C1512409	HGF	3082	P14210	hepatocyte growth factor	671	0.374	0.885	0.99947	0.07	1	7
Hepatocarcinogenesis	C1512409	IL6	3569	P05231	interleukin 6	2367	0.248	0.962	0.31536	0.06	1	6
Hepatocarcinogenesis	C1512409	RASSF1	11186	Q9NS23	Ras association domain family member 1	400	0.42	0.808	4.9E-06	0.06	1	6
Hepatocarcinogenesis	C1512409	GSTP1	2950	P09211	glutathione S-transferase pi 1	610	0.383	0.923	0.01416	0.06	1	6
Hepatocarcinogenesis	C1512409	PIK3CG	5294	P48736	osphatidylinositol-4,5-bisphosphate 3-kinase catalytic subunit gam	1101	0.32	0.885	1.4E-06	0.06	1	6
Hepatocarcinogenesis	C1512409	MTDH	92140	Q86UE4	metadherin	200	0.491	0.769	0.32924	0.06	1	6
Hepatocarcinogenesis	C1512409	PIK3CB	5291	P42338	hosphatidylinositol-4,5-bisphosphate 3-kinase catalytic subunit be	1083	0.322	0.885	0.99964	0.06	1	6
Hepatocarcinogenesis	C1512409	PIK3CD	5293	O00329	hosphatidylinositol-4,5-bisphosphate 3-kinase catalytic subunit del	1119	0.319	0.885	0.99999	0.06	1	6
Hepatocarcinogenesis	C1512409	AKR1B10	57016	O60218	aldo-keto reductase family 1 member B10	130	0.546	0.769	7.6E-09	0.06	1	6
Hepatocarcinogenesis	C1512409	PIK3CA	5290	P42336	hosphatidylinositol-4,5-bisphosphate 3-kinase catalytic subunit alp	1511	0.292	0.923	1	0.06	1	6
Hepatocarcinogenesis	C1512409	SOCS1	8651	O15524	suppressor of cytokine signaling 1	315	0.445	0.808	0.58544	0.06	1	6
Hepatocarcinogenesis	C1512409	HPGDS	27306	O60760	hematopoietic prostaglandin D synthase	570	0.388	0.923	1.8E-06	0.06	1	6
Hepatocarcinogenesis	C1512409	MET	4233	P08581	MET proto-oncogene, receptor tyrosine kinase	594	0.38	0.846	0.96998	0.05	0.8	5
Hepatocarcinogenesis	C1512409	IGF2R	3482	P11717	insulin like growth factor 2 receptor	166	0.518	0.731	1	0.05	0.6	5
Hepatocarcinogenesis	C1512409	STAT3	6774	P40763	signal transducer and activator of transcription 3	1193	0.32	0.923	1	0.05	1	5
Hepatocarcinogenesis	C1512409	PKM	5315	P14618	pyruvate kinase M1/2	237	0.482	0.808	0.8549	0.05	1	5
Hepatocarcinogenesis	C1512409	ESR1	2099	P03372	estrogen receptor 1	1101	0.324	0.962	0.9992	0.05	1	5
Hepatocarcinogenesis	C1512409	TGFB1	7040	P01137	transforming growth factor beta 1	1558	0.287	0.962	0.03685	0.05	1	5
Hepatocarcinogenesis	C1512409	DYNLL1	8655	P63167	dynein light chain LC8-type 1	103	0.568	0.769	0.6983	0.05	1	5
Hepatocarcinogenesis	C1512409	CDKN1B	1027	P46527	cyclin dependent kinase inhibitor 1B	454	0.409	0.769	0.62376	0.05	1	5
Hepatocarcinogenesis	C1512409	GJB1	2705	P08034	gap junction protein beta 1	224	0.514	0.731	0.84627	0.05	1	5
Hepatocarcinogenesis	C1512409	DLEC1	9940	Q9Y238	DLEC1 cilia and flagella associated protein	94	0.572	0.615	2.1E-35	0.05	1	5
Hepatocarcinogenesis	C1512409	ALB	213	P02768	albumin	1198	0.317	0.962	0.64329	0.05	1	5
Hepatocarcinogenesis	C1512409	YAP1	10413	P46937	Yes associated protein 1	374	0.432	0.808	0.99918	0.05	1	5
Hepatocarcinogenesis	C1512409	IFNL3	282617	Q8IZI9	interferon lambda 3	175	0.506	0.808	2.4E-08	0.05	0.8	5
Hepatocarcinogenesis	C1512409	MIR221	407006		microRNA 221	304	0.449	0.846		0.04	1	4
Hepatocarcinogenesis	C1512409	TMED7-TICAM	100302736	Q86XR7;Q9Y3B3	TMED7-TICAM2 readthrough	329	0.438	0.769	0.00259	0.04	1	4

Hepatocarcinogenesis	C1512409	MAPK1	5594	P28482	mitogen-activated protein kinase 1	1059	0.33	0.923	0.99698	0.04	1	4
Hepatocarcinogenesis	C1512409	NOTCH1	4851	P46531	notch receptor 1	693	0.369	0.885	1	0.04	0.75	4
Hepatocarcinogenesis	C1512409	TMED7	51014	Q86XR7;Q9Y3B3	transmembrane p24 trafficking protein 7	336	0.436	0.808	0.47966	0.04	1	4
Hepatocarcinogenesis	C1512409	PSMD9	5715	O00233	proteasome 26S subunit, non-ATPase 9	344	0.435	0.846	4.9E-06	0.04	1	4
Hepatocarcinogenesis	C1512409	VEGFA	7422	P15692	vascular endothelial growth factor A	1899	0.266	0.923	2.4E-05	0.04	1	4
Hepatocarcinogenesis	C1512409	TICAM2	353376	Q86XR7;Q9Y3B3	toll like receptor adaptor molecule 2	332	0.438	0.769	0.00259	0.04	1	4
Hepatocarcinogenesis	C1512409	FASN	2194	P49327	fatty acid synthase	245	0.474	0.846	1	0.04	1	4
Hepatocarcinogenesis	C1512409	CCL4	6351	P13236;Q8NHW4	C-C motif chemokine ligand 4	276	0.466	0.923	0.24134	0.04	1	4
Hepatocarcinogenesis	C1512409	S100A9	6280	P06702	S100 calcium binding protein A9	363	0.433	0.885	0.4777	0.04	1	4
Hepatocarcinogenesis	C1512409	POU5F1P4	645682		POU class 5 homeobox 1 pseudogene 4	284	0.452	0.808		0.04	1	4
Hepatocarcinogenesis	C1512409	MIR152	406943		microRNA 152	136	0.521	0.769		0.04	0.75	4
Hepatocarcinogenesis	C1512409	PTPN11	5781	Q06124	protein tyrosine phosphatase non-receptor type 11	702	0.385	0.923	0.99998	0.04	1	4
Hepatocarcinogenesis	C1512409	STMN1	3925	P16949	stathmin 1	284	0.458	0.885	0.07524	0.04	1	4
Hepatocarcinogenesis	C1512409	CDKN2B	1030	P42772	cyclin dependent kinase inhibitor 2B	440	0.417	0.769	0.00734	0.04	0.75	4
Hepatocarcinogenesis	C1512409	H3P23	115482696		H3 histone pseudogene 23	310	0.443	0.769		0.04	1	4
Hepatocarcinogenesis	C1512409	TERT	7015	O14746	telomerase reverse transcriptase	703	0.374	0.846	0.99046	0.04	1	4
Hepatocarcinogenesis	C1512409	DCTN6	10671	O00399	dynactin subunit 6	315	0.442	0.769	0.05574	0.04	1	4
Hepatocarcinogenesis	C1512409	CIB1	10519	Q99828	calcium and integrin binding 1	275	0.458	0.769	0.00033	0.04	1	4
Hepatocarcinogenesis	C1512409	ZNRD2	10534	O60232	zinc ribbon domain containing 2	311	0.443	0.769	1.8E-07	0.04	1	4
Hepatocarcinogenesis	C1512409	IFI27	3429	P40305	interferon alpha inducible protein 27	332	0.437	0.769	0.0107	0.04	1	4
Hepatocarcinogenesis	C1512409	CDKN1A	1026	P38936	cyclin dependent kinase inhibitor 1A	490	0.403	0.769	0.00186	0.04	1	4
Hepatocarcinogenesis	C1512409	SQSTM1	8878	Q13501	sequestosome 1	470	0.428	0.885	0.00086	0.04	1	4
Hepatocarcinogenesis	C1512409	MAT1A	4143	Q00266	methionine adenosyltransferase 1A	103	0.582	0.577	0.00081	0.04	1	4
Hepatocarcinogenesis	C1512409	EGFR	1956	P00533	epidermal growth factor receptor	1394	0.295	0.885	0.36837	0.04	1	4
Hepatocarcinogenesis	C1512409	RAF1	5894	P04049	Raf-1 proto-oncogene, serine/threonine kinase	470	0.418	0.885	0.85295	0.04	1	4
Hepatocarcinogenesis	C1512409	ABCB4	5244	P21439	ATP binding cassette subfamily B member 4	157	0.529	0.615	2.2E-07	0.04	1	4
Hepatocarcinogenesis	C1512409	TNF	7124	P01375	tumor necrosis factor	2724	0.231	0.962	0.8033	0.04	1	4
Hepatocarcinogenesis	C1512409	NOTCH2	4853	Q04721	notch receptor 2	384	0.441	0.808	1	0.04	0.75	4
Hepatocarcinogenesis	C1512409	HIF1A	3091	Q16665	hypoxia inducible factor 1 subunit alpha	1044	0.327	0.923	0.9777	0.04	1	4
Hepatocarcinogenesis	C1512409	SUB1	10923	P53999	SUB1 regulator of transcription	260	0.465	0.846	0.89245	0.04	1	4
Hepatocarcinogenesis	C1512409	EZH2	2146	Q15910	enhancer of zeste 2 polycomb repressive complex 2 subunit	653	0.376	0.885	1	0.04	1	4
Hepatocarcinogenesis	C1512409	PDGFRA	5156	P16234	platelet derived growth factor receptor alpha	452	0.415	0.808	1	0.03	1	3
Hepatocarcinogenesis	C1512409	DNMT1	1786	P26358	DNA methyltransferase 1	496	0.406	0.885	1	0.03	1	3
Hepatocarcinogenesis	C1512409	SERPINE1	5054	P05121	serpin family E member 1	770	0.359	0.885	0.04361	0.03	1	3
Hepatocarcinogenesis	C1512409	MDM2	4193	Q00987	MDM2 proto-oncogene	702	0.362	0.846	0.99981	0.03	1	3
Hepatocarcinogenesis	C1512409	PIN1	5300	Q13526	peptidylprolyl cis/trans isomerase, NIMA-interacting 1	212	0.488	0.769	0.74031	0.03	1	3
Hepatocarcinogenesis	C1512409	CCNE1	898	P24864	cyclin E1	133	0.538	0.731	0.97187	0.03	0.667	3
Hepatocarcinogenesis	C1512409	MAPK8	5599	P45983	mitogen-activated protein kinase 8	520	0.397	0.885	0.99823	0.03	1	3
Hepatocarcinogenesis	C1512409	PTEN	5728	P60484	phosphatase and tensin homolog	1349	0.305	0.923	0.25651	0.03	1	3
Hepatocarcinogenesis	C1512409	POU5F1	5460	Q01860	POU class 5 homeobox 1	328	0.441	0.846	0.95371	0.03	1	3
Hepatocarcinogenesis	C1512409	EPCAM	4072	P16422	epithelial cell adhesion molecule	328	0.451	0.885	1.3E-07	0.03	1	3
Hepatocarcinogenesis	C1512409	MTOR	2475	P42345	mechanistic target of rapamycin kinase	960	0.343	0.885	1	0.03	1	3
Hepatocarcinogenesis	C1512409	PCNA	5111	P12004	proliferating cell nuclear antigen	581	0.382	0.846	0.97606	0.03	1	3
Hepatocarcinogenesis	C1512409	KLF6	1316	Q99612	Kruppel like factor 6	204	0.501	0.846	0.97946	0.03	1	3
Hepatocarcinogenesis	C1512409	MIR182	406958		microRNA 182	211	0.486	0.846		0.03	1	3
Hepatocarcinogenesis	C1512409	HDAC1	3065	Q13547	histone deacetylase 1	277	0.46	0.846	0.61427	0.03	1	3
Hepatocarcinogenesis	C1512409	TGFBR2	7048	P37173	transforming growth factor beta receptor 2	502	0.413	0.846	0.12535	0.03	1	3
Hepatocarcinogenesis	C1512409	EGF	1950	P01133	epidermal growth factor	774	0.357	0.923	8.5E-17	0.03	1	3
Hepatocarcinogenesis	C1512409	RIPK1	8737	Q13546	receptor interacting serine/threonine kinase 1	250	0.479	0.769	0.00794	0.03	1	3
Hepatocarcinogenesis	C1512409	APC	324	P25054	APC regulator of WNT signaling pathway	703	0.373	0.962	1	0.03	0.667	3
Hepatocarcinogenesis	C1512409	FOXM1	2305	Q08050	forkhead box M1	323	0.441	0.769	0.00074	0.03	1	3
Hepatocarcinogenesis	C1512409	CASP3	836	P42574	caspase 3	819	0.351	0.923	0.10017	0.03	1	3
Hepatocarcinogenesis	C1512409	BTG2	7832	P78543	BTG anti-proliferation factor 2	77	0.587	0.538	0.01903	0.03	1	3
Hepatocarcinogenesis	C1512409	AHR	196	P35869	aryl hydrocarbon receptor	532	0.41	0.923	0.99999	0.03	1	3
Hepatocarcinogenesis	C1512409	CDK5RAP3	80279	Q96JB5	CDK5 regulatory subunit associated protein 3	29	0.7	0.462	7.6E-15	0.03	1	3

Hepatocarcinogenesis	C1512409	PNPLA3	80339	Q9NST1	patatin like phospholipase domain containing 3	132	0.556	0.692	5.7E-08	0.03	1	3
Hepatocarcinogenesis	C1512409	HMGA2	8091	P52926	high mobility group AT-hook 2	382	0.429	0.808	0.87015	0.03	1	3
Hepatocarcinogenesis	C1512409	AXIN1	8312	O15169	axin 1	118	0.559	0.577	0.40765	0.03	1	3
Hepatocarcinogenesis	C1512409	HTATIP2	10553	Q9BUP3	HIV-1 Tat interactive protein 2	63	0.608	0.577	5.4E-13	0.03	1	3
Hepatocarcinogenesis	C1512409	HNF1A	6927	P20823	HNF1 homeobox A	292	0.478	0.846	0.96115	0.03	1	3
Hepatocarcinogenesis	C1512409	XPO1	7514	O14980	exportin 1	178	0.504	0.692	1	0.03	1	3
Hepatocarcinogenesis	C1512409	MIR155	406947		microRNA 155	584	0.384	0.885		0.03	1	3
Hepatocarcinogenesis	C1512409	POU5F1P3	642559		POU class 5 homeobox 1 pseudogene 3	285	0.452	0.808		0.03	1	3
Hepatocarcinogenesis	C1512409	E2F1	1869	Q01094	E2F transcription factor 1	324	0.441	0.846	0.9877	0.03	1	3
Hepatocarcinogenesis	C1512409	LGALS3BP	3959	Q08380	galectin 3 binding protein	127	0.546	0.769	2.7E-07	0.03	1	3
Hepatocarcinogenesis	C1512409	MALAT1	378938		metastasis associated lung adenocarcinoma transcript 1	336	0.435	0.846		0.03	1	3
Hepatocarcinogenesis	C1512409	AREG	374	P15514	amphiregulin	221	0.487	0.769	0.35407	0.03	1	3
Hepatocarcinogenesis	C1512409	SOAT1	6646	P35610	sterol O-acyltransferase 1	389	0.424	0.846	7.4E-10	0.03	1	3
Hepatocarcinogenesis	C1512409	GTF2H1	2965	P32780	general transcription factor IIH subunit 1	258	0.469	0.846	0.98982	0.03	1	3
Hepatocarcinogenesis	C1512409	SIRT2	22933	Q8IXJ6	sirtuin 2	172	0.51	0.769	2.3E-08	0.03	1	3
Hepatocarcinogenesis	C1512409	CDH1	999	P12830	cadherin 1	508	0.401	0.808	0.14631	0.03	1	3
Hepatocarcinogenesis	C1512409	NRAS	4893	P01111	NRAS proto-oncogene, GTPase	611	0.39	0.808	0.49009	0.03	1	3
Hepatocarcinogenesis	C1512409	STK11	6794	Q15831	serine/threonine kinase 11	372	0.435	0.808	0.9934	0.03	1	3
Hepatocarcinogenesis	C1512409	DAPK2	23604	Q9UIK4	death associated protein kinase 2	130	0.536	0.692	5E-10	0.02	1	2
Hepatocarcinogenesis	C1512409	GFER	2671	P55789	growth factor, augmenter of liver regeneration	120	0.56	0.846	0.00017	0.02	1	2
Hepatocarcinogenesis	C1512409	MSH2	4436	P43246	mutS homolog 2	490	0.406	0.808	0.89539	0.02	1	2
Hepatocarcinogenesis	C1512409	AOC1	26	P19801	amine oxidase copper containing 1	61	0.628	0.769	6.7E-08	0.02	1	2
Hepatocarcinogenesis	C1512409	MRC1	4360	P22897	mannose receptor C-type 1	431	0.413	0.846	0.94335	0.02	1	2
Hepatocarcinogenesis	C1512409	MYD88	4615	Q99836	MYD88 innate immune signal transduction adaptor	480	0.414	0.923	0.12443	0.02	1	2
Hepatocarcinogenesis	C1512409	MYBL2	4605	P10244	MYB proto-oncogene like 2	80	0.59	0.538	0.12226	0.02	1	2
Hepatocarcinogenesis	C1512409	MGMT	4255	P16455	O-6-methylguanine-DNA methyltransferase	444	0.412	0.885	1.2E-07	0.02	1	2
Hepatocarcinogenesis	C1512409	POLDIP2	26073	Q9Y2S7	DNA polymerase delta interacting protein 2	530	0.396	0.923	0.41407	0.02	1	2
Hepatocarcinogenesis	C1512409	LAMTOR2	28956	Q9Y2Q5	late endosomal/lysosomal adaptor, MAPK and MTOR activator 2	129	0.538	0.808	0.19816	0.02	1	2
Hepatocarcinogenesis	C1512409	SETD2	29072	Q9BYW2	SET domain containing 2, histone lysine methyltransferase	447	0.424	0.846	1	0.02	1	2
Hepatocarcinogenesis	C1512409	NXT1	29107	Q9UKK6	nuclear transport factor 2 like export factor 1	178	0.505	0.731	0.64574	0.02	1	2
Hepatocarcinogenesis	C1512409	IL1B	3553	P01584	interleukin 1 beta	1801	0.276	0.962	0.13005	0.02	1	2
Hepatocarcinogenesis	C1512409	IL1A	3552	P01583	interleukin 1 alpha	1002	0.333	0.962	0.00016	0.02	1	2
Hepatocarcinogenesis	C1512409	CCN1	3491	O00622	cellular communication network factor 1	246	0.473	0.731	0.71388	0.02	1	2
Hepatocarcinogenesis	C1512409	HSF1	3297	Q00613	heat shock transcription factor 1	185	0.507	0.769	0.74762	0.02	1	2
Hepatocarcinogenesis	C1512409	SMAD7	4092	O15105	SMAD family member 7	269	0.47	0.769	0.54854	0.02	1	2
Hepatocarcinogenesis	C1512409	IGF1R	3480	P08069	insulin like growth factor 1 receptor	556	0.399	0.885	0.96799	0.02	1	2
Hepatocarcinogenesis	C1512409	GPX2	2877	P18283	glutathione peroxidase 2	60	0.619	0.5	0.00015	0.02	1	2
Hepatocarcinogenesis	C1512409	MIR214	406996		microRNA 214	221	0.48	0.808		0.02	1	2
Hepatocarcinogenesis	C1512409	FGF21	26291	Q9NSA1	fibroblast growth factor 21	236	0.485	0.769	0.02944	0.02	1	2
Hepatocarcinogenesis	C1512409	MAT2A	4144	P31153	methionine adenosyltransferase 2A	81	0.615	0.654	0.99846	0.02	1	2
Hepatocarcinogenesis	C1512409	NUPR1	26471	O60356	nuclear protein 1, transcriptional regulator	68	0.606	0.615	0.00415	0.02	1	2
Hepatocarcinogenesis	C1512409	GJA1	2697	P17302	gap junction protein alpha 1	662	0.393	0.885	0.15523	0.02	1	2
Hepatocarcinogenesis	C1512409	MIR34A	407040		microRNA 34a	428	0.421	0.846		0.02	1	2
Hepatocarcinogenesis	C1512409	GPT	2875	P24298	glutamic--pyruvic transaminase	511	0.403	0.923	3.6E-19	0.02	1	2
Hepatocarcinogenesis	C1512409	MIR30E	407034		microRNA 30e	88	0.576	0.769		0.02	1	2
Hepatocarcinogenesis	C1512409	ARR3	407	P36575	arrestin 3	279	0.46	0.846	0.10401	0.02	1	2
Hepatocarcinogenesis	C1512409	HSPA4	3308	P34932	heat shock protein family A (Hsp70) member 4	550	0.394	0.923	0.99954	0.02	1	2
Hepatocarcinogenesis	C1512409	HSPA5	3309	P11021	heat shock protein family A (Hsp70) member 5	350	0.434	0.885	0.77341	0.02	1	2
Hepatocarcinogenesis	C1512409	NCL	4691	P19338	nucleolin	167	0.509	0.808	0.99992	0.02	1	2
Hepatocarcinogenesis	C1512409	AKAP12	9590	Q02952	A-kinase anchoring protein 12	99	0.566	0.615	0.03567	0.02	0.5	2
Hepatocarcinogenesis	C1512409	H4C3	8364	P62805	H4 clustered histone 3	132	0.534	0.731	0.00036	0.02	1	2
Hepatocarcinogenesis	C1512409	H4C11	8363	P62805	H4 clustered histone 11	121	0.541	0.692	0.38683	0.02	1	2
Hepatocarcinogenesis	C1512409	H4C12	8362	P62805	H4 clustered histone 12	121	0.541	0.692	0.00157	0.02	1	2
Hepatocarcinogenesis	C1512409	H4C6	8361	P62805	H4 clustered histone 6	121	0.541	0.692	0.13072	0.02	1	2
Hepatocarcinogenesis	C1512409	H4C4	8360	P62805	H4 clustered histone 4	125	0.537	0.692	0.00199	0.02	1	2

Hepatocarcinogenesis	C1512409	H4C1	8359	P62805	H4 clustered histone 1	121	0.541	0.692	0.00021	0.02	1	2
Hepatocarcinogenesis	C1512409	AXIN2	8313	Q9Y2T1	axin 2	169	0.507	0.692	0.57015	0.02	1	2
Hepatocarcinogenesis	C1512409	H4C9	8294	P62805	H4 clustered histone 9	121	0.541	0.692	0.01581	0.02	1	2
Hepatocarcinogenesis	C1512409	STAM	8027	Q92783	signal transducing adaptor molecule	20	0.736	0.269	0.85048	0.02	1	2
Hepatocarcinogenesis	C1512409	AIMP2	7965	Q13155	inoacyl tRNA synthetase complex interacting multifunctional prote	555	0.393	0.923	0.00802	0.02	1	2
Hepatocarcinogenesis	C1512409	CAD	790	P27708	yl-phosphate synthetase 2, aspartate transcarbamylase, and dihyd	249	0.482	0.846	0.99993	0.02	1	2
Hepatocarcinogenesis	C1512409	YY1	7528	P25490	YY1 transcription factor	245	0.481	0.808	0.9938	0.02	1	2
Hepatocarcinogenesis	C1512409	HULC	728655		hepatocellular carcinoma up-regulated long non-coding RNA	72	0.606	0.538		0.02	1	2
Hepatocarcinogenesis	C1512409	TPM3	7170	P06753	tropomyosin 3	297	0.49	0.808	0.00665	0.02	1	2
Hepatocarcinogenesis	C1512409	TLR4	7099	O00206	toll like receptor 4	1174	0.321	0.962	4.6E-09	0.02	1	2
Hepatocarcinogenesis	C1512409	H4C8	8365	P62805	H4 clustered histone 8	122	0.541	0.692	7.2E-06	0.02	1	2
Hepatocarcinogenesis	C1512409	H4C2	8366	P62805	H4 clustered histone 2	128	0.537	0.731	0.00292	0.02	1	2
Hepatocarcinogenesis	C1512409	PTGES	9536	O14684	prostaglandin E synthase	150	0.54	0.769	0.71317	0.02	0.5	2
Hepatocarcinogenesis	C1512409	ATG5	9474	Q9H1Y0	autophagy related 5	282	0.462	0.885	0.97758	0.02	1	2
Hepatocarcinogenesis	C1512409	GRAP2	9402	O75791	GRB2 related adaptor protein 2	538	0.394	0.923	0.00015	0.02	1	2
Hepatocarcinogenesis	C1512409	XPR1	9213	Q9UBH6	xenotropic and polytropic retrovirus receptor 1	217	0.488	0.731	0.99999	0.02	1	2
Hepatocarcinogenesis	C1512409	DCLK1	9201	O15075	doublecortin like kinase 1	123	0.545	0.615	0.99964	0.02	1	2
Hepatocarcinogenesis	C1512409	STARD13	90627	Q9Y3M8	StAR related lipid transfer domain containing 13	60	0.641	0.538	2.4E-08	0.02	1	2
Hepatocarcinogenesis	C1512409	SOCS3	9021	O14543	suppressor of cytokine signaling 3	375	0.434	0.808	0.74613	0.02	1	2
Hepatocarcinogenesis	C1512409	SPHK1	8877	Q9NYA1	sphingosine kinase 1	236	0.48	0.808	3E-07	0.02	1	2
Hepatocarcinogenesis	C1512409	DLK1	8788	P80370	delta like non-canonical Notch ligand 1	144	0.527	0.692	0.03458	0.02	1	2
Hepatocarcinogenesis	C1512409	TNFSF10	8743	P50591	TNF superfamily member 10	445	0.413	0.885	0.13495	0.02	1	2
Hepatocarcinogenesis	C1512409	RUNX3	864	Q13761	RUNX family transcription factor 3	281	0.459	0.731	0.84295	0.02	1	2
Hepatocarcinogenesis	C1512409	IKBKG	8517	Q9Y6K9	nhibitor of nuclear factor kappa B kinase regulatory subunit gamm	252	0.493	0.769	0.08485	0.02	1	2
Hepatocarcinogenesis	C1512409	CASR	846	P41180	calcium sensing receptor	517	0.41	0.846	0.0467	0.02	1	2
Hepatocarcinogenesis	C1512409	H4C13	8368	P62805	H4 clustered histone 13	121	0.541	0.692	0.00035	0.02	1	2
Hepatocarcinogenesis	C1512409	H4C5	8367	P62805	H4 clustered histone 5	121	0.541	0.692	0.01345	0.02	1	2
Hepatocarcinogenesis	C1512409	THY1	7070	P04216	Thy-1 cell surface antigen	197	0.496	0.808	0.0499	0.02	1	2
Hepatocarcinogenesis	C1512409	TAZ	6901	Q16635	tafazzin	252	0.477	0.808	0.72591	0.02	1	2
Hepatocarcinogenesis	C1512409	MAP3K7	6885	O43318	mitogen-activated protein kinase kinase kinase 7	273	0.477	0.808	0.99971	0.02	1	2
Hepatocarcinogenesis	C1512409	URGCP	55665	Q8TCY9	upregulator of cell proliferation	37	0.674	0.385	0.57799	0.02	1	2
Hepatocarcinogenesis	C1512409	H4C15	554313	P62805	H4 clustered histone 15	121	0.541	0.692		0.02	1	2
Hepatocarcinogenesis	C1512409	LAPTM4B	55353	Q86VI4	lysosomal protein transmembrane 4 beta	88	0.579	0.692	4.3E-13	0.02	1	2
Hepatocarcinogenesis	C1512409	MSL2	55167	Q9HCI7	MSL complex subunit 2	9	0.821	0.269	0.62406	0.02	1	2
Hepatocarcinogenesis	C1512409	PPARG	5468	P37231	peroxisome proliferator activated receptor gamma	877	0.358	0.885	0.02919	0.02	1	2
Hepatocarcinogenesis	C1512409	PML	5371	P29590	promyelocytic leukemia	274	0.477	0.846	0.21291	0.02	1	2
Hepatocarcinogenesis	C1512409	PIM1	5292	P11309	Pim-1 proto-oncogene, serine/threonine kinase	224	0.482	0.769	0.84846	0.02	1	2
Hepatocarcinogenesis	C1512409	SF3B6	51639	Q9Y3B4	splicing factor 3b subunit 6	116	0.544	0.808	0.58902	0.02	1	2
Hepatocarcinogenesis	C1512409	SIRT6	51548	Q8N6T7	sirtuin 6	195	0.496	0.846	0.03912	0.02	1	2
Hepatocarcinogenesis	C1512409	DCTN4	51164	Q9UJW0	dynactin subunit 4	253	0.472	0.846	0.73221	0.02	1	2
Hepatocarcinogenesis	C1512409	ACOX1	51	Q15067	acyl-CoA oxidase 1	95	0.61	0.731	0.0621	0.02	1	2
Hepatocarcinogenesis	C1512409	REG3A	5068	Q06141	regenerating family member 3 alpha	148	0.522	0.769	0.00063	0.02	1	2
Hepatocarcinogenesis	C1512409	PRDX1	5052	Q06830	peroxiredoxin 1	237	0.485	0.846	7.7E-10	0.02	1	2
Hepatocarcinogenesis	C1512409	NOX4	50507	Q9NPH5	NADPH oxidase 4	260	0.471	0.885	5.8E-20	0.02	1	2
Hepatocarcinogenesis	C1512409	NME2	4831	P22392	NME/NM23 nucleoside diphosphate kinase 2	95	0.573	0.731	8.8E-09	0.02	1	2
Hepatocarcinogenesis	C1512409	PRKAR1A	5573	P10644	protein kinase cAMP-dependent type I regulatory subunit alpha	597	0.401	0.846	0.99985	0.02	1	2
Hepatocarcinogenesis	C1512409	H4C14	8370	P62805	H4 clustered histone 14	121	0.541	0.692		0.02	1	2
Hepatocarcinogenesis	C1512409	AURKA	6790	O14965	aurora kinase A	245	0.475	0.731	0.8676	0.02	1	2
Hepatocarcinogenesis	C1512409	SPP1	6696	P10451	secreted phosphoprotein 1	824	0.353	0.885	2.1E-06	0.02	1	2
Hepatocarcinogenesis	C1512409	SPG7	6687	Q9UQ90	SPG7 matrix AAA peptidase subunit, paraplegin	419	0.436	0.885	2E-36	0.02	1	2
Hepatocarcinogenesis	C1512409	NME1-NME2	654364	P22392	NME1-NME2 readthrough	64	0.623	0.615	8.8E-09	0.02	1	2
Hepatocarcinogenesis	C1512409	CXADRP1	653108		CXADR pseudogene 1	272	0.463	0.846		0.02	1	2
Hepatocarcinogenesis	C1512409	UCA1	652995		urothelial cancer associated 1	152	0.512	0.808		0.02	1	2
Hepatocarcinogenesis	C1512409	SKP2	6502	Q13309	S-phase kinase associated protein 2	204	0.492	0.808	0.96042	0.02	1	2
Hepatocarcinogenesis	C1512409	BMI1	648	P35226	BMI1 proto-oncogene, polycomb ring finger	309	0.448	0.808	0.94286	0.02	1	2

Hepatocarcinogenesis	C1512409	SHBG	6462	P04278	sex hormone binding globulin	368	0.438	0.808	1.3E-06	0.02	1	2
Hepatocarcinogenesis	C1512409	SFRP1	6422	Q8N474	secreted frizzled related protein 1	227	0.479	0.846	0.96457	0.02	1	2
Hepatocarcinogenesis	C1512409	BHMT	635	Q93088	betaine--homocysteine S-methyltransferase	71	0.595	0.731	3.7E-14	0.02	1	2
Hepatocarcinogenesis	C1512409	RING1	6015	Q06587	ring finger protein 1	42	0.653	0.423	0.64854	0.02	0.5	2
Hepatocarcinogenesis	C1512409	AICDA	57379	Q9GZX7	activation induced cytidine deaminase	265	0.463	0.885	9.2E-06	0.02	1	2
Hepatocarcinogenesis	C1512409	SLC12A9	56996	Q9BXP2	solute carrier family 12 member 9	280	0.454	0.769	1.2E-15	0.02	1	2
Hepatocarcinogenesis	C1512409	SULF2	55959	Q8IWU5	sulfatase 2	75	0.603	0.615	0.01059	0.02	1	2
Hepatocarcinogenesis	C1512409	NFKB1	4790	P19838	nuclear factor kappa B subunit 1	551	0.396	0.923	1	0.02	1	2
Hepatocarcinogenesis	C1512409	ETS2	2114	P15036	ETS proto-oncogene 2, transcription factor	115	0.548	0.808	0.99888	0.02	1	2
Hepatocarcinogenesis	C1512409	DNMT3A	1788	Q9Y6K1	DNA methyltransferase 3 alpha	350	0.445	0.846	7.4E-39	0.02	1	2
Hepatocarcinogenesis	C1512409	H3P9	115482684		H3 histone pseudogene 9	177	0.505	0.731		0.02	1	2
Hepatocarcinogenesis	C1512409	H3P8	115482693		H3 histone pseudogene 8	116	0.544	0.808		0.02	1	2
Hepatocarcinogenesis	C1512409	PEG10	23089	Q86TG7	paternally expressed 10	75	0.597	0.615	0.87274	0.02	1	2
Hepatocarcinogenesis	C1512409	AKR1B1	231	P15121	aldo-keto reductase family 1 member B	220	0.491	0.885	2E-05	0.02	1	2
Hepatocarcinogenesis	C1512409	DIO3	1735	P55073	iodothyronine deiodinase 3	80	0.593	0.731	0.62331	0.02	1	2
Hepatocarcinogenesis	C1512409	CDK2AP2	10263	O75956	cyclin dependent kinase 2 associated protein 2	117	0.544	0.808	0.78801	0.02	1	2
Hepatocarcinogenesis	C1512409	CLU	1191	P10909	clusterin	412	0.426	0.885	7.8E-05	0.02	1	2
Hepatocarcinogenesis	C1512409	H4-16	121504	P62805	H4 histone 16	122	0.54	0.692	0.02228	0.02	1	2
Hepatocarcinogenesis	C1512409	CYP1A2	1544	P05177	cytochrome P450 family 1 subfamily A member 2	218	0.494	0.923	2.5E-10	0.02	1	2
Hepatocarcinogenesis	C1512409	NLRP3	114548	Q96P20	NLR family pyrin domain containing 3	805	0.361	0.962	0.00081	0.02	1	2
Hepatocarcinogenesis	C1512409	TRIM13	10206	O60858	tripartite motif containing 13	275	0.462	0.846	0.6366	0.02	1	2
Hepatocarcinogenesis	C1512409	CXADR	1525	P78310	CXADR Ig-like cell adhesion molecule	305	0.454	0.885	0.36867	0.02	1	2
Hepatocarcinogenesis	C1512409	SIRT1	23411	Q96EB6	sirtuin 1	675	0.378	0.885	0.08733	0.02	1	2
Hepatocarcinogenesis	C1512409	CDK2	1017	P24941	cyclin dependent kinase 2	270	0.456	0.692	0.61054	0.02	0.5	2
Hepatocarcinogenesis	C1512409	FOS	2353	P01100	Fos proto-oncogene, AP-1 transcription factor subunit	528	0.411	0.885	0.26176	0.02	1	2
Hepatocarcinogenesis	C1512409	PTGR1	22949	Q14914	prostaglandin reductase 1	32	0.729	0.231	3.5E-08	0.02	1	2
Hepatocarcinogenesis	C1512409	RPIA	22934	P49247	ribose 5-phosphate isomerase A	52	0.691	0.462	0.00014	0.02	1	2
Hepatocarcinogenesis	C1512409	CDKN2C	1031	P42773	cyclin dependent kinase inhibitor 2C	149	0.527	0.654	0.39526	0.02	1	2
Hepatocarcinogenesis	C1512409	RUVBL2	10856	Q9Y230	RuvB like AAA ATPase 2	23	0.743	0.385	0.99992	0.02	1	2
Hepatocarcinogenesis	C1512409	DLL3	10683	Q9NYJ7	delta like canonical Notch ligand 3	134	0.552	0.654	5.4E-05	0.02	1	2
Hepatocarcinogenesis	C1512409	KHDRBS1	10657	Q07666	KH RNA binding domain containing, signal transduction associated	289	0.457	0.846	0.99423	0.02	1	2
Hepatocarcinogenesis	C1512409	ENG	2022	P17813	endoglin	371	0.446	0.846	0.99858	0.02	1	2
Hepatocarcinogenesis	C1512409	EGR1	1958	P18146	early growth response 1	378	0.433	0.923	0.3509	0.02	1	2
Hepatocarcinogenesis	C1512409	FGFR4	2264	P22455	fibroblast growth factor receptor 4	220	0.49	0.769	7.2E-13	0.02	0.5	2
Hepatocarcinogenesis	C1512409	EDN1	1906	P05305	endothelin 1	679	0.38	0.846	0.45504	0.02	1	2
Hepatocarcinogenesis	C1512409	COPS5	10987	Q92905	COP9 signalosome subunit 5	101	0.566	0.769	0.99925	0.02	1	2
Hepatocarcinogenesis	C1512409	AHSA1	10598	O95433	activator of HSP90 ATPase activity 1	526	0.396	0.923	0.99896	0.02	1	2
Hepatocarcinogenesis	C1512409	MRPL28	10573	Q13084	mitochondrial ribosomal protein L28	178	0.505	0.731	2E-07	0.02	1	2
Hepatocarcinogenesis	C1512409	AKT2	208	P31751	AKT serine/threonine kinase 2	264	0.474	0.769	0.64432	0.02	1	2
Hepatocarcinogenesis	C1512409	FHL2	2274	Q14192	four and a half LIM domains 2	121	0.556	0.731	2.9E-05	0.02	1	2
Hepatocarcinogenesis	C1512409	RPP14	11102	O95059	ribonuclease P/MRP subunit p14	117	0.544	0.808	0.00193	0.02	1	2
Hepatocarcinogenesis	C1512409	NDRG1	10397	Q92597	N-myc downstream regulated 1	225	0.488	0.731	0.01132	0.02	1	2
Hepatocarcinogenesis	C1512409	CDKN3	1033	Q16667	cyclin dependent kinase inhibitor 3	150	0.514	0.769	0.00195	0.02	1	2
Hepatocarcinogenesis	C1512409	SAT2	112483	Q96F10	spermidine/spermine N1-acetyltransferase family member 2	37	0.686	0.462	2.8E-06	0.02	1	2
Hepatocarcinogenesis	C1512409	SULF1	23213	Q8IWU6	sulfatase 1	83	0.585	0.538	0.00138	0.02	1	2
Hepatocarcinogenesis	C1512409	NUP62	23636	P37198	nucleoporin 62	273	0.47	0.885	0.95006	0.02	1	2
Hepatocarcinogenesis	C1512409	NAT2	10	P11245	N-acetyltransferase 2	311	0.451	0.885	3.3E-06	0.02	1	2
Hepatocarcinogenesis	C1512409	HOTAIR	100124700		HOX transcript antisense RNA	234	0.475	0.769		0.02	1	2
Hepatocarcinogenesis	C1512409	PRDX5	25824	P30044	peroxiredoxin 5	153	0.522	0.808	0.00018	0.02	1	2
Hepatocarcinogenesis	C1512409	MIR675	100033819		microRNA 675	69	0.595	0.654		0.02	1	2
Hepatocarcinogenesis	C1512409	LILRB1	10859	Q8NHL6	leukocyte immunoglobulin like receptor B1	168	0.51	0.769	5.7E-14	0.02	0.5	2
Hepatocarcinogenesis	C1512409	CRK	1398	P46108	CRK proto-oncogene, adaptor protein	544	0.394	0.923	0.95936	0.02	1	2
Hepatocarcinogenesis	C1512409	DDX11-AS1	100506660		DDX11 antisense RNA 1	11	0.839	0.115		0.02	1	2
Hepatocarcinogenesis	C1512409	MAPK14	1432	Q16539	mitogen-activated protein kinase 14	626	0.379	0.923	0.37466	0.02	1	2
Hepatocarcinogenesis	C1512409	G6PD	2539	P11413	glucose-6-phosphate dehydrogenase	410	0.424	0.846	0.97158	0.02	1	2

Hepatocarcinogenesis	C1512409	G6PC	2538	P35575	glucose-6-phosphatase catalytic subunit	132	0.56	0.692	1.8E-05	0.02	1	2
Hepatocarcinogenesis	C1512409	RNF19A	25897	Q9NV58	ring finger protein 19A, RBR E3 ubiquitin protein ligase	523	0.397	0.923	0.02531	0.02	1	2
Hepatocarcinogenesis	C1512409	PTPRO	5800	Q16827	protein tyrosine phosphatase receptor type O	50	0.656	0.423	1.2E-05	0.01	1	1
Hepatocarcinogenesis	C1512409	ADRM1	11047	Q16186	adhesion regulating molecule 1	51	0.633	0.538	0.99704	0.01	1	1
Hepatocarcinogenesis	C1512409	RIPK3	11035	Q9Y572	receptor interacting serine/threonine kinase 3	196	0.497	0.885	1.2E-14	0.01	1	1
Hepatocarcinogenesis	C1512409	ACOT1	641371	Q86TX2	acyl-CoA thioesterase 1	14	0.78	0.192	6.4E-05	0.01	1	1
Hepatocarcinogenesis	C1512409	MIR602	693187		microRNA 602	11	0.792	0.231		0.01	1	1
Hepatocarcinogenesis	C1512409	MIR615	693200		microRNA 615	41	0.647	0.615		0.01	1	1
Hepatocarcinogenesis	C1512409	DBH-AS1	138948		DBH antisense RNA 1	18	0.78	0.192		0.01	1	1
Hepatocarcinogenesis	C1512409	TERF1	7013	P54274	telomeric repeat binding factor 1	102	0.563	0.692	0.90655	0.01	1	1
Hepatocarcinogenesis	C1512409	IGDCC4	57722	Q8TDY8	immunoglobulin superfamily DCC subclass member 4	5	1	0.077	2.4E-06	0.01	1	1
Hepatocarcinogenesis	C1512409	TERF2	7014	Q15554	telomeric repeat binding factor 2	125	0.541	0.731	0.99237	0.01	1	1
Hepatocarcinogenesis	C1512409	SPARC	6678	P09486	secreted protein acidic and cysteine rich	344	0.445	0.846	0.89148	0.01	1	1
Hepatocarcinogenesis	C1512409	USP28	57646	Q96RU2	ubiquitin specific peptidase 28	35	0.691	0.269	4.5E-13	0.01	1	1
Hepatocarcinogenesis	C1512409	WDR5	11091	P61964	WD repeat domain 5	61	0.619	0.692	0.99978	0.01	1	1
Hepatocarcinogenesis	C1512409	SUV39H1	6839	O43463	suppressor of variegation 3-9 homolog 1	77	0.595	0.577	0.98957	0.01	1	1
Hepatocarcinogenesis	C1512409	SREBF2	6721	Q12772	sterol regulatory element binding transcription factor 2	112	0.566	0.577	0.20734	0.01	1	1
Hepatocarcinogenesis	C1512409	SRF	6722	P11831	serum response factor	147	0.534	0.808	0.97545	0.01	1	1
Hepatocarcinogenesis	C1512409	PTPRD	5789	P23468	protein tyrosine phosphatase receptor type D	93	0.584	0.731	1	0.01	1	1
Hepatocarcinogenesis	C1512409	ST13	6767	P50502	ST13 Hsp70 interacting protein	81	0.584	0.692	0.66074	0.01	1	1
Hepatocarcinogenesis	C1512409	SEC63	11231	Q9UGP8	SEC63 homolog, protein translocation regulator	24	0.736	0.308	2.1E-07	0.01	1	1
Hepatocarcinogenesis	C1512409	SCARNA13	677768		small Cajal body-specific RNA 13	4	0.89	0.154		0.01	1	1
Hepatocarcinogenesis	C1512409	WIF1	11197	Q9Y5W5	WNT inhibitory factor 1	171	0.507	0.731	1.3E-09	0.01	1	1
Hepatocarcinogenesis	C1512409	SPTBN1	6711	Q01082	spectrin beta, non-erythrocytic 1	76	0.619	0.538	1	0.01	1	1
Hepatocarcinogenesis	C1512409	PTPN13	5783	Q12923	protein tyrosine phosphatase non-receptor type 13	77	0.592	0.654	1.4E-25	0.01	1	1
Hepatocarcinogenesis	C1512409	PDAP1	11333	Q13442	PDGFA associated protein 1	95	0.576	0.769	0.04963	0.01	1	1
Hepatocarcinogenesis	C1512409	BSG	682	P35613	basigin (Ok blood group)	287	0.458	0.769	2.6E-05	0.01	1	1
Hepatocarcinogenesis	C1512409	SPINK1	6690	P00995	serine peptidase inhibitor Kazal type 1	216	0.492	0.808	0.31476	0.01	1	1
Hepatocarcinogenesis	C1512409	ZNF398	57541	Q8TD17	zinc finger protein 398	27	0.695	0.308	0.14568	0.01	1	1
Hepatocarcinogenesis	C1512409	TFCP2	7024	Q12800	transcription factor CP2	46	0.65	0.385	3.6E-06	0.01	1	1
Hepatocarcinogenesis	C1512409	TFF1	7031	P04155	trefoil factor 1	147	0.529	0.769	0.00089	0.01	1	1
Hepatocarcinogenesis	C1512409	TLE1	7088	Q04724	TLE family member 1, transcriptional corepressor	106	0.56	0.654	0.99922	0.01	1	1
Hepatocarcinogenesis	C1512409	TLR3	7098	O15455	toll like receptor 3	393	0.427	0.885	1.6E-07	0.01	1	1
Hepatocarcinogenesis	C1512409	PGRMC1	10857	O00264	progesterone receptor membrane component 1	79	0.588	0.654	0.6711	0.01	1	1
Hepatocarcinogenesis	C1512409	MIR506	574511		microRNA 506	76	0.585	0.615		0.01	1	1
Hepatocarcinogenesis	C1512409	TNFRSF1A	7132	P19438	TNF receptor superfamily member 1A	487	0.409	0.923	0.9949	0.01	1	1
Hepatocarcinogenesis	C1512409	HSPH1	10808	Q92598	heat shock protein family H (Hsp110) member 1	48	0.65	0.538	0.99822	0.01	1	1
Hepatocarcinogenesis	C1512409	TP73	7161	O15350	tumor protein p73	300	0.449	0.808	0.99696	0.01	1	1
Hepatocarcinogenesis	C1512409	TPD52	7163	P55327	tumor protein D52	76	0.597	0.692	8.5E-05	0.01	1	1
Hepatocarcinogenesis	C1512409	SEPTIN9	10801	Q9UHD8	septin 9	150	0.548	0.769	0.99579	0.01	1	1
Hepatocarcinogenesis	C1512409	TRAF2	7186	Q12933	TNF receptor associated factor 2	111	0.546	0.769	0.99979	0.01	1	1
Hepatocarcinogenesis	C1512409	TRAF6	7189	Q9Y4K3	TNF receptor associated factor 6	254	0.472	0.808	0.99739	0.01	1	1
Hepatocarcinogenesis	C1512409	MIR657	724027		microRNA 657	11	0.861	0.269		0.01	1	1
Hepatocarcinogenesis	C1512409	TSC2	7249	P49815	TSC complex subunit 2	410	0.439	0.808	1	0.01	1	1
Hepatocarcinogenesis	C1512409	TSG101	7251	Q99816	tumor susceptibility 101	80	0.588	0.615	0.34407	0.01	0	1
Hepatocarcinogenesis	C1512409	TKT	7086	P29401	transketolase	92	0.595	0.769	0.00675	0.01	1	1
Hepatocarcinogenesis	C1512409	BTG1	694	P62324	BTG anti-proliferation factor 1	98	0.564	0.692	0.53641	0.01	1	1
Hepatocarcinogenesis	C1512409	TJP1	7082	Q07157	tight junction protein 1	91	0.576	0.692	1	0.01	1	1
Hepatocarcinogenesis	C1512409	MIB1	57534	Q86YT6	mindbomb E3 ubiquitin protein ligase 1	329	0.438	0.769	3.4E-88	0.01	1	1
Hepatocarcinogenesis	C1512409	TFRC	7037	P02786	transferrin receptor	359	0.437	0.885	7E-05	0.01	1	1
Hepatocarcinogenesis	C1512409	KCNQ1OT1	10984		KCNQ1 opposite strand/antisense transcript 1	159	0.534	0.731		0.01	1	1
Hepatocarcinogenesis	C1512409	APOBEC2	10930	Q9Y235	apolipoprotein B mRNA editing enzyme catalytic subunit 2	19	0.751	0.423	0.01707	0.01	1	1
Hepatocarcinogenesis	C1512409	TGFB2	7042	P61812	transforming growth factor beta 2	389	0.433	0.885	0.99905	0.01	1	1
Hepatocarcinogenesis	C1512409	TGFBI	7045	Q15582	transforming growth factor beta induced	225	0.484	0.769	6E-08	0.01	1	1
Hepatocarcinogenesis	C1512409	BLCAP	10904	P62952	BLCAP apoptosis inducing factor	27	0.705	0.385	0.50953	0.01	1	1

Hepatocarcinogenesis	C1512409	NLN	57486	Q9BYT8	neurolysin	74	0.597	0.731	3.9E-12	0.01	1	1
Hepatocarcinogenesis	C1512409	TGM2	7052	P21980	transglutaminase 2	315	0.445	0.808	6.8E-18	0.01	1	1
Hepatocarcinogenesis	C1512409	TH	7054	P07101	tyrosine hydroxylase	321	0.462	0.885	1.7E-06	0.01	1	1
Hepatocarcinogenesis	C1512409	THPO	7066	P40225	thrombopoietin	185	0.513	0.692	0.19714	0.01	1	1
Hepatocarcinogenesis	C1512409	MOK	5891	Q9UQ07	MOK protein kinase	251	0.476	0.769	8.8E-22	0.01	1	1
Hepatocarcinogenesis	C1512409	TIMP1	7076	P01033	TIMP metallopeptidase inhibitor 1	603	0.38	0.885	0.4993	0.01	1	1
Hepatocarcinogenesis	C1512409	TIMP3	7078	P35625	TIMP metallopeptidase inhibitor 3	325	0.445	0.923	0.63037	0.01	1	1
Hepatocarcinogenesis	C1512409	TTPA	7274	P49638	alpha tocopherol transfer protein	147	0.612	0.577	0.00144	0.01	1	1
Hepatocarcinogenesis	C1512409	RNASE3	6037	P12724	ribonuclease A family member 3	207	0.49	0.808		0.01	1	1
Hepatocarcinogenesis	C1512409	COX8A	1351	P10176	cytochrome c oxidase subunit 8A	526	0.4	0.846	0.11606	0.01	1	1
Hepatocarcinogenesis	C1512409	S100B	6285	P04271	S100 calcium binding protein B	599	0.383	0.923	0.04387	0.01	1	1
Hepatocarcinogenesis	C1512409	SERPINB3	6317	P29508	serpin family B member 3	101	0.565	0.615	8.2E-16	0.01	1	1
Hepatocarcinogenesis	C1512409	CEACAM1	634	P13688	CEA cell adhesion molecule 1	167	0.525	0.731	0.00079	0.01	1	1
Hepatocarcinogenesis	C1512409	ADH5	128	P11766	alcohol dehydrogenase 5 (class III), chi polypeptide	65	0.604	0.731	2.5E-11	0.01	1	1
Hepatocarcinogenesis	C1512409	PLK3	1263	Q9H4B4	polo like kinase 3	52	0.644	0.538	1.9E-06	0.01	1	1
Hepatocarcinogenesis	C1512409	PRDM1	639	O75626	PR/SET domain 1	126	0.538	0.731	0.9565	0.01	1	1
Hepatocarcinogenesis	C1512409	TSPYL2	64061	Q9H2G4	TSPY like 2	63	0.615	0.769	0.87393	0.01	1	1
Hepatocarcinogenesis	C1512409	CENPK	64105	Q9BS16	centromere protein K	19	0.743	0.423	3.1E-11	0.01	1	1
Hepatocarcinogenesis	C1512409	CKS1BP7	137529		CDC28 protein kinase regulatory subunit 1B pseudogene 7	63	0.61	0.577		0.01	1	1
Hepatocarcinogenesis	C1512409	RBP1	5947	P09455	retinol binding protein 1	103	0.565	0.731	5.9E-06	0.01	1	1
Hepatocarcinogenesis	C1512409	RB1	5925	P06400	RB transcriptional corepressor 1	339	0.444	0.885	1	0.01	1	1
Hepatocarcinogenesis	C1512409	SNHG6	641638		small nucleolar RNA host gene 6	51	0.633	0.423		0.01	1	1
Hepatocarcinogenesis	C1512409	SET	6418	Q01105	SET nuclear proto-oncogene	183	0.507	0.808	0.99597	0.01	1	1
Hepatocarcinogenesis	C1512409	CCR7	1236	P32248	C-C motif chemokine receptor 7	320	0.443	0.769	0.05762	0.01	1	1
Hepatocarcinogenesis	C1512409	BCL2	596	P10415	BCL2 apoptosis regulator	1456	0.291	0.885	0.55903	0.01	1	1
Hepatocarcinogenesis	C1512409	RELB	5971	Q01201	RELB proto-oncogene, NF-kB subunit	72	0.597	0.731	0.994	0.01	1	1
Hepatocarcinogenesis	C1512409	RNF2	6045	Q99496	ring finger protein 2	63	0.608	0.615	0.99743	0.01	1	1
Hepatocarcinogenesis	C1512409	BCL3	602	P20749	BCL3 transcription coactivator	136	0.541	0.731	0.98871	0.01	1	1
Hepatocarcinogenesis	C1512409	GAS5	60674		growth arrest specific 5	184	0.497	0.808		0.01	1	1
Hepatocarcinogenesis	C1512409	ROS1	6098	P08922	ROS proto-oncogene 1, receptor tyrosine kinase	356	0.439	0.885	1.6E-72	0.01	1	1
Hepatocarcinogenesis	C1512409	RP9	6100	Q8TA86	RP9 pre-mRNA splicing factor	54	0.67	0.5	0.01896	0.01	1	1
Hepatocarcinogenesis	C1512409	OPN1SW	611	P03999	opsin 1, short wave sensitive	164	0.519	0.769	0.02113	0.01	1	1
Hepatocarcinogenesis	C1512409	CPS1	1373	P31327	carbamoyl-phosphate synthase 1	133	0.59	0.615	1.1E-09	0.01	1	1
Hepatocarcinogenesis	C1512409	RPL29	6159	P47914	ribosomal protein L29	48	0.644	0.462	0.19534	0.01	1	1
Hepatocarcinogenesis	C1512409	RPS3	6188	P23396	ribosomal protein S3	26	0.7	0.346	0.92	0.01	1	1
Hepatocarcinogenesis	C1512409	RPS6KA1	6195	Q15418	ribosomal protein S6 kinase A1	68	0.619	0.654	0.00191	0.01	1	1
Hepatocarcinogenesis	C1512409	RPS6KA3	6197	P51812	ribosomal protein S6 kinase A3	315	0.491	0.846	0.99999	0.01	1	1
Hepatocarcinogenesis	C1512409	RPS27	6232	P42677	ribosomal protein S27	104	0.578	0.769	0.75663	0.01	1	1
Hepatocarcinogenesis	C1512409	RREB1	6239	Q92766	ras responsive element binding protein 1	227	0.5	0.808	1	0.01	1	1
Hepatocarcinogenesis	C1512409	S100A1	6271	P23297	S100 calcium binding protein A1	396	0.421	0.846	0.006	0.01	1	1
Hepatocarcinogenesis	C1512409	S100A8	6279	P05109	S100 calcium binding protein A8	369	0.43	0.962	0.13761	0.01	1	1
Hepatocarcinogenesis	C1512409	SFRP2	6423	Q96HF1	secreted frizzled related protein 2	134	0.544	0.731	0.00167	0.01	1	1
Hepatocarcinogenesis	C1512409	SFRP5	6425	Q5T4F7	secreted frizzled related protein 5	106	0.559	0.692	5.2E-06	0.01	1	1
Hepatocarcinogenesis	C1512409	BCAT1	586	P54687	branched chain amino acid transaminase 1	62	0.606	0.538	3.8E-08	0.01	1	1
Hepatocarcinogenesis	C1512409	SLC10A1	6554	Q14973	solute carrier family 10 member 1	54	0.626	0.462	4.8E-16	0.01	1	1
Hepatocarcinogenesis	C1512409	SLC22A3	6581	O75751	solute carrier family 22 member 3	154	0.531	0.846	7.8E-10	0.01	1	1
Hepatocarcinogenesis	C1512409	SMARCA1	6594	P28370	l, matrix associated, actin dependent regulator of chromatin, subfa	183	0.504	0.808	1	0.01	1	1
Hepatocarcinogenesis	C1512409	SMARCA4	6597	P51532	l, matrix associated, actin dependent regulator of chromatin, subfa	347	0.45	0.885	1	0.01	1	1
Hepatocarcinogenesis	C1512409	PVT1	5820		Pvt1 oncogene	190	0.495	0.731		0.01	1	1
Hepatocarcinogenesis	C1512409	SNAI1	6615	O95863	snail family transcriptional repressor 1	243	0.474	0.808	0.21754	0.01	1	1
Hepatocarcinogenesis	C1512409	SNCG	6623	O76070	synuclein gamma	122	0.55	0.615	0.00085	0.01	1	1
Hepatocarcinogenesis	C1512409	CIRBP	1153	Q14011	cold inducible RNA binding protein	72	0.592	0.731	0.33636	0.01	1	1
Hepatocarcinogenesis	C1512409	SOD1	6647	P00441	superoxide dismutase 1	689	0.379	0.962	0.1773	0.01	1	1
Hepatocarcinogenesis	C1512409	SOD3	6649	P08294	superoxide dismutase 3	138	0.531	0.808	0.00072	0.01	1	1
Hepatocarcinogenesis	C1512409	WFDC1	58189	Q9HC57	WAP four-disulfide core domain 1	22	0.743	0.346	3.5E-11	0.01	1	1

Hepatocarcinogenesis	C1512409	SOS1	6654	Q07889	SOS Ras/Rac guanine nucleotide exchange factor 1	261	0.485	0.808	1	0.01	1	1
Hepatocarcinogenesis	C1512409	SOX4	6659	Q06945	SRY-box transcription factor 4	246	0.489	0.808	0.92656	0.01	1	1
Hepatocarcinogenesis	C1512409	BOK	666	Q9UMX3	BCL2 family apoptosis regulator BOK	27	0.722	0.346	0.16279	0.01	1	1
Hepatocarcinogenesis	C1512409	GGTLC5P	653590		gamma-glutamyltransferase light chain 5 pseudogene	165	0.513	0.846		0.01	1	1
Hepatocarcinogenesis	C1512409	CREBBP	1387	Q92793	CREB binding protein	438	0.428	0.808	1	0.01	1	1
Hepatocarcinogenesis	C1512409	SPPL3	121665	Q8TCT6	signal peptide peptidase like 3	50	0.638	0.615	0.99656	0.01	1	1
Hepatocarcinogenesis	C1512409	HHIP	64399	Q96QV1	hedgehog interacting protein	122	0.55	0.654	0.95436	0.01	1	1
Hepatocarcinogenesis	C1512409	RASSF10	644943	A6NK89	Ras association domain family member 10	53	0.626	0.385		0.01	1	1
Hepatocarcinogenesis	C1512409	RASA1	5921	P20936	RAS p21 protein activator 1	237	0.481	0.885	1	0.01	1	1
Hepatocarcinogenesis	C1512409	CLEC7A	64581	Q9BXN2	C-type lectin domain containing 7A	126	0.553	0.769	5.5E-12	0.01	1	1
Hepatocarcinogenesis	C1512409	PLAAT4	5920	Q9UL19	phospholipase A and acyltransferase 4	190	0.5	0.808	1.2E-07	0.01	1	1
Hepatocarcinogenesis	C1512409	SIAH1	6477	Q8IUQ4	siah E3 ubiquitin protein ligase 1	54	0.631	0.538	0.69866	0.01	1	1
Hepatocarcinogenesis	C1512409	CKS1B	1163	P61024	CDC28 protein kinase regulatory subunit 1B	78	0.587	0.654	0.4314	0.01	1	1
Hepatocarcinogenesis	C1512409	RARRES2	5919	Q99969	retinoic acid receptor responder 2	180	0.519	0.731	2.7E-05	0.01	1	1
Hepatocarcinogenesis	C1512409	CTHRC1	115908	Q96CG8	collagen triple helix repeat containing 1	105	0.556	0.692	2.7E-05	0.01	1	1
Hepatocarcinogenesis	C1512409	SLC5A2	6524	P31639	solute carrier family 5 member 2	214	0.499	0.769	4.9E-14	0.01	1	1
Hepatocarcinogenesis	C1512409	SLC5A5	6528	Q92911	solute carrier family 5 member 5	195	0.499	0.769	0.00295	0.01	1	1
Hepatocarcinogenesis	C1512409	RARA	5914	P10276	retinoic acid receptor alpha	274	0.47	0.846	0.96305	0.01	1	1
Hepatocarcinogenesis	C1512409	RAP1A	5906	P62834	RAP1A, member of RAS oncogene family	215	0.502	0.808	0.62227	0.01	1	1
Hepatocarcinogenesis	C1512409	ZNF689	115509	Q96CS4	zinc finger protein 689	8	0.821	0.154	0.41789	0.01	1	1
Hepatocarcinogenesis	C1512409	SOX9	6662	P48436	SRY-box transcription factor 9	466	0.419	0.846	0.99772	0.01	1	1
Hepatocarcinogenesis	C1512409	RUNX2	860	Q13950	RUNX family transcription factor 2	405	0.437	0.846	0.94884	0.01	1	1
Hepatocarcinogenesis	C1512409	EIF1	10209	P41567	eukaryotic translation initiation factor 1	37	0.653	0.577	0.8307	0.01	1	1
Hepatocarcinogenesis	C1512409	BUD31	8896	P41223	BUD31 homolog	26	0.711	0.5	0.23109	0.01	1	1
Hepatocarcinogenesis	C1512409	PRPF4B	8899	Q13523	pre-mRNA processing factor 4B	15	0.76	0.269	1	0.01	1	1
Hepatocarcinogenesis	C1512409	MBD4	8930	O95243	methyl-CpG binding domain 4, DNA glycosylase	62	0.61	0.615	2E-14	0.01	1	1
Hepatocarcinogenesis	C1512409	MBD2	8932	Q9UBB5	methyl-CpG binding domain protein 2	256	0.466	0.846	0.34957	0.01	1	1
Hepatocarcinogenesis	C1512409	CCND2	894	P30279	cyclin D2	241	0.477	0.808	0.98658	0.01	1	1
Hepatocarcinogenesis	C1512409	WNT3A	89780	P56704	Wnt family member 3A	141	0.532	0.769	0.96491	0.01	1	1
Hepatocarcinogenesis	C1512409	PSME3	10197	P61289	proteasome activator subunit 3	78	0.587	0.654	0.99714	0.01	1	1
Hepatocarcinogenesis	C1512409	PLOD3	8985	O60568	procollagen-lysine,2-oxoglutarate 5-dioxygenase 3	59	0.659	0.385	6.1E-13	0.01	1	1
Hepatocarcinogenesis	C1512409	CDKL2	8999	Q92772	cyclin dependent kinase like 2	20	0.751	0.346	3.1E-17	0.01	1	1
Hepatocarcinogenesis	C1512409	CCNG1	900	P51959	cyclin G1	53	0.628	0.615	0.00073	0.01	1	1
Hepatocarcinogenesis	C1512409	UNC5A	90249	Q6ZN44	unc-5 netrin receptor A	24	0.729	0.423	0.99983	0.01	1	1
Hepatocarcinogenesis	C1512409	MLIP	90523	Q5VWP3	muscular LMNA interacting protein	37	0.682	0.577	3.2E-16	0.01	1	1
Hepatocarcinogenesis	C1512409	PRC1	9055	O43663	protein regulator of cytokinesis 1	73	0.599	0.692	2.7E-07	0.01	1	1
Hepatocarcinogenesis	C1512409	SLC25A13	10165	Q9UJS0	solute carrier family 25 member 13	109	0.617	0.5	1.3E-24	0.01	1	1
Hepatocarcinogenesis	C1512409	RGN	9104	Q15493	regucalcin	96	0.566	0.692	0.02212	0.01	1	1
Hepatocarcinogenesis	C1512409	IER3	8870	P46695	immediate early response 3	78	0.593	0.769	0.63122	0.01	1	1
Hepatocarcinogenesis	C1512409	NR1I2	8856	O75469	nuclear receptor subfamily 1 group I member 2	462	0.418	0.846	7.3E-09	0.01	1	1
Hepatocarcinogenesis	C1512409	PROM1	8842	O43490	prominin 1	477	0.41	0.846	1.9E-22	0.01	1	1
Hepatocarcinogenesis	C1512409	TP63	8626	Q9H3D4	tumor protein p63	816	0.362	0.769	0.99728	0.01	1	1
Hepatocarcinogenesis	C1512409	SIK1B	102724428	0A0B4J2F2;P5705	salt inducible kinase 1B (putative)	48	0.653	0.385		0.01	1	1
Hepatocarcinogenesis	C1512409	NCOA1	8648	Q15788	nuclear receptor coactivator 1	108	0.563	0.731	0.99981	0.01	1	1
Hepatocarcinogenesis	C1512409	NUMB	8650	P49757	NUMB endocytic adaptor protein	55	0.619	0.731	0.004	0.01	1	1
Hepatocarcinogenesis	C1512409	.OC10272419	102724197		inactive glutathione hydrolase 2	105	0.559	0.731		0.01	1	1
Hepatocarcinogenesis	C1512409	ABCC3	8714	O15438	ATP binding cassette subfamily C member 3	160	0.517	0.769	1.2E-26	0.01	1	1
Hepatocarcinogenesis	C1512409	TMX2-CTNND	100528016		TMX2-CTNND1 readthrough (NMD candidate)	138	0.538	0.769		0.01	1	1
Hepatocarcinogenesis	C1512409	EED	8726	O75530	embryonic ectoderm development	123	0.588	0.615	0.99929	0.01	1	1
Hepatocarcinogenesis	C1512409	SF3B4	10262	Q15427	splicing factor 3b subunit 4	110	0.576	0.615	0.99176	0.01	1	1
Hepatocarcinogenesis	C1512409	SPRY2	10253	O43597	sprouty RTK signaling antagonist 2	111	0.554	0.654	0.97126	0.01	1	1
Hepatocarcinogenesis	C1512409	TNFRSF6B	8771	O95407	TNF receptor superfamily member 6b	145	0.528	0.731	3.9E-06	0.01	1	1
Hepatocarcinogenesis	C1512409	AK6	102157402	Q9Y3D8	adenylate kinase 6	38	0.678	0.538		0.01	1	1
Hepatocarcinogenesis	C1512409	TNFRSF10B	8795	O14763	TNF receptor superfamily member 10b	179	0.505	0.769	0.0002	0.01	1	1
Hepatocarcinogenesis	C1512409	PEX11A	8800	O75192	peroxisomal biogenesis factor 11 alpha	13	0.792	0.346	8.5E-07	0.01	1	1

Hepatocarcinogenesis	C1512409	TRIM24	8805	O15164	tripartite motif containing 24	79	0.581	0.615	1	0.01	1	1
Hepatocarcinogenesis	C1512409	BANF1	8815	O75531	BAF nuclear assembly factor 1	86	0.599	0.692	0.6518	0.01	1	1
Hepatocarcinogenesis	C1512409	MTA1	9112	Q13330	metastasis associated 1	118	0.547	0.692	1	0.01	0	1
Hepatocarcinogenesis	C1512409	CCNE2	9134	O96020	cyclin E2	59	0.608	0.538	0.86983	0.01	0	1
Hepatocarcinogenesis	C1512409	BCAR1	9564	P56945	BCAR1 scaffold protein, Cas family member	176	0.518	0.846	0.99808	0.01	1	1
Hepatocarcinogenesis	C1512409	CLOCK	9575	O15516	clock circadian regulator	309	0.463	0.885	0.99957	0.01	1	1
Hepatocarcinogenesis	C1512409	APOBEC3B	9582	Q9UH17	apolipoprotein B mRNA editing enzyme catalytic subunit 3B	118	0.547	0.731	5.2E-16	0.01	1	1
Hepatocarcinogenesis	C1512409	RBM39	9584	Q14498	RNA binding motif protein 39	35	0.686	0.462	0.99981	0.01	1	1
Hepatocarcinogenesis	C1512409	CD24	100133941	P25063	CD24 molecule	244	0.472	0.769		0.01	1	1
Hepatocarcinogenesis	C1512409	CD44	960	P16070	CD44 molecule (Indian blood group)	711	0.363	0.962	2.4E-07	0.01	1	1
Hepatocarcinogenesis	C1512409	TCL1B	9623	O95988	T cell leukemia/lymphoma 1B	100	0.561	0.5	0.01013	0.01	1	1
Hepatocarcinogenesis	C1512409	SETD1A	9739	O15047	SET domain containing 1A, histone lysine methyltransferase	68	0.619	0.615	1	0.01	1	1
Hepatocarcinogenesis	C1512409	CD81	975	P60033	CD81 molecule	122	0.555	0.846	0.91844	0.01	1	1
Hepatocarcinogenesis	C1512409	HDAC4	9759	P56524	histone deacetylase 4	256	0.484	0.885	1	0.01	1	1
Hepatocarcinogenesis	C1512409	DLGAP5	9787	Q15398	DLG associated protein 5	33	0.678	0.423	1.2E-14	0.01	1	1
Hepatocarcinogenesis	C1512409	CUL7	9820	Q14999	cullin 7	90	0.633	0.5	2.6E-21	0.01	1	1
Hepatocarcinogenesis	C1512409	ZEB2	9839	O60315	zinc finger E-box binding homeobox 2	292	0.471	0.808	1	0.01	1	1
Hepatocarcinogenesis	C1512409	GAB2	9846	Q9UQC2	GRB2 associated binding protein 2	110	0.563	0.692	0.98325	0.01	1	1
Hepatocarcinogenesis	C1512409	KIF14	9928	Q15058	kinesin family member 14	103	0.578	0.654	4.1E-05	0.01	1	1
Hepatocarcinogenesis	C1512409	MVP	9961	Q14764	major vault protein	172	0.512	0.769	6.8E-08	0.01	1	1
Hepatocarcinogenesis	C1512409	CHD1L	9557	Q86WJ1	chromodomain helicase DNA binding protein 1 like	64	0.631	0.577	1E-36	0.01	1	1
Hepatocarcinogenesis	C1512409	MACROH2A1	9555	O75367	macroH2A.1 histone	43	0.656	0.5	0.73705	0.01	1	1
Hepatocarcinogenesis	C1512409	TP53I11	9537	O14683	tumor protein p53 inducible protein 11	9	0.861	0.154	0.30718	0.01	1	1
Hepatocarcinogenesis	C1512409	EXO1	9156	Q9UQ84	exonuclease 1	87	0.578	0.692	8.1E-13	0.01	0	1
Hepatocarcinogenesis	C1512409	SCAF11	9169	Q99590	SR-related CTD associated factor 11	77	0.584	0.731	1	0.01	1	1
Hepatocarcinogenesis	C1512409	CDH13	1012	P55290	cadherin 13	205	0.501	0.846	0.00071	0.01	1	1
Hepatocarcinogenesis	C1512409	GGTLC1	92086	Q9BX51	gamma-glutamyltransferase light chain 1	105	0.559	0.731	3.3E-05	0.01	1	1
Hepatocarcinogenesis	C1512409	DNM1L	10059	O00429	dynamin 1 like	273	0.475	0.808	0.00057	0.01	1	1
Hepatocarcinogenesis	C1512409	NOLC1	9221	Q14978	nucleolar and coiled-body phosphoprotein 1	47	0.641	0.615	0.0001	0.01	1	1
Hepatocarcinogenesis	C1512409	PTTG1	9232	O95997	PTTG1 regulator of sister chromatid separation, securin	140	0.526	0.654	0.00042	0.01	1	1
Hepatocarcinogenesis	C1512409	PIWIL1	9271	Q96J94	piwi like RNA-mediated gene silencing 1	99	0.57	0.731	7.3E-14	0.01	1	1
Hepatocarcinogenesis	C1512409	SLC9A3R2	9351	Q15599	SLC9A3 regulator 2	83	0.588	0.654	0.03835	0.01	1	1
Hepatocarcinogenesis	C1512409	PPIG	9360	Q13427	peptidylprolyl isomerase G	203	0.497	0.846	0.96292	0.01	1	1
Hepatocarcinogenesis	C1512409	KL	9365	Q9UEF7	klotho	332	0.448	0.769	0.00022	0.01	1	1
Hepatocarcinogenesis	C1512409	OPN4	94233	Q9UHM6	opsin 4	87	0.579	0.769	9E-13	0.01	1	1
Hepatocarcinogenesis	C1512409	MED23	9439	Q9ULK4	mediator complex subunit 23	90	0.612	0.654	3.1E-08	0.01	1	1
Hepatocarcinogenesis	C1512409	H3P41	100420410		H3 histone pseudogene 41	24	0.705	0.308		0.01	1	1
Hepatocarcinogenesis	C1512409	TBPL1	9519	P62380	TATA-box binding protein like 1	198	0.506	0.846	0.37902	0.01	1	1
Hepatocarcinogenesis	C1512409	HOTTIP	100316868		HOXA distal transcript antisense RNA	90	0.575	0.654		0.01	1	1
Hepatocarcinogenesis	C1512409	SRC	6714	P12931	SRC proto-oncogene, non-receptor tyrosine kinase	265	0.467	0.885	0.99691	0.01	1	1
Hepatocarcinogenesis	C1512409	CAV1	857	Q03135	caveolin 1	633	0.388	0.885	0.00827	0.01	1	1
Hepatocarcinogenesis	C1512409	GGTLC3	728226	B5MD39	gamma-glutamyltransferase light chain family member 3	165	0.513	0.846		0.01	1	1
Hepatocarcinogenesis	C1512409	USP7	7874	Q93009	ubiquitin specific peptidase 7	153	0.538	0.808	1	0.01	1	1
Hepatocarcinogenesis	C1512409	CXCR6	10663	O00574	C-X-C motif chemokine receptor 6	325	0.448	0.885	5.7E-05	0.01	1	1
Hepatocarcinogenesis	C1512409	CENPM	79019	Q9NSP4	centromere protein M	16	0.736	0.269	0.01501	0.01	1	1
Hepatocarcinogenesis	C1512409	FSD1	79187	Q9BTV5	fibronectin type III and SPRY domain containing 1	220	0.486	0.808	0.25199	0.01	1	1
Hepatocarcinogenesis	C1512409	RTL10	79680	Q7L3V2	retrotransposon Gag like 10	37	0.67	0.577		0.01	1	1
Hepatocarcinogenesis	C1512409	LIN28A	79727	Q9H9Z2	lin-28 homolog A	161	0.523	0.731	0.01937	0.01	1	1
Hepatocarcinogenesis	C1512409	E2F8	79733	A0AVK6	E2F transcription factor 8	34	0.674	0.423	0.81778	0.01	1	1
Hepatocarcinogenesis	C1512409	ATAD5	79915	Q96QE3	ATPase family AAA domain containing 5	33	0.711	0.385	1	0.01	1	1
Hepatocarcinogenesis	C1512409	IGF2BP2	10644	Q9Y6M1	insulin like growth factor 2 mRNA binding protein 2	94	0.588	0.615	0.23861	0.01	1	1
Hepatocarcinogenesis	C1512409	IGF2BP1	10642	Q9NZI8	insulin like growth factor 2 mRNA binding protein 1	106	0.56	0.692	0.99933	0.01	1	1
Hepatocarcinogenesis	C1512409	PDPN	10630	Q86YL7	podoplanin	256	0.47	0.769	0.00032	0.01	1	1
Hepatocarcinogenesis	C1512409	USP5	8078	P45974	ubiquitin specific peptidase 5	40	0.67	0.577	0.96396	0.01	1	1
Hepatocarcinogenesis	C1512409	COL18A1	80781	P39060	collagen type XVIII alpha 1 chain	323	0.449	0.808	1.7E-10	0.01	1	1

Hepatocarcinogenesis	C1512409	ZNF436	80818	Q9C0F3	zinc finger protein 436	40	0.67	0.5	5.1E-05	0.01	1	1
Hepatocarcinogenesis	C1512409	TXNIP	10628	Q9H3M7	thioredoxin interacting protein	208	0.49	0.731	0.15918	0.01	1	1
Hepatocarcinogenesis	C1512409	TCL1A	8115	P56279	T cell leukemia/lymphoma 1A	106	0.558	0.5	5.7E-10	0.01	1	1
Hepatocarcinogenesis	C1512409	CTCF	10664	P49711	CCCTC-binding factor	186	0.504	0.769	0.99994	0.01	1	1
Hepatocarcinogenesis	C1512409	ZMYM2	7750	Q9UBW7	zinc finger MYM-type containing 2	77	0.599	0.654	0.66648	0.01	1	1
Hepatocarcinogenesis	C1512409	ZFP36	7538	P26651	ZFP36 ring finger protein	161	0.523	0.692	0.75244	0.01	1	1
Hepatocarcinogenesis	C1512409	GGT2	728441	P36268	gamma-glutamyltransferase 2	165	0.513	0.846	0.00013	0.01	1	1
Hepatocarcinogenesis	C1512409	PLK4	10733	O00444	polo like kinase 4	135	0.551	0.731	7.1E-06	0.01	1	1
Hepatocarcinogenesis	C1512409	GGTLC4P	729838		gamma-glutamyltransferase light chain 4 pseudogene	165	0.513	0.846		0.01	1	1
Hepatocarcinogenesis	C1512409	UBE2E2	7325	Q96LR5	ubiquitin conjugating enzyme E2 E2	30	0.695	0.423	0.96378	0.01	1	1
Hepatocarcinogenesis	C1512409	UBE2N	7334	P61088	ubiquitin conjugating enzyme E2 N	80	0.593	0.654	0.88156	0.01	1	1
Hepatocarcinogenesis	C1512409	UBE2V1	7335	A5PLL7;Q13404	ubiquitin conjugating enzyme E2 V1	46	0.641	0.654	0.00012	0.01	1	1
Hepatocarcinogenesis	C1512409	UCP1	7350	P25874	uncoupling protein 1	124	0.545	0.769	1.7E-11	0.01	1	1
Hepatocarcinogenesis	C1512409	UGT1A	7361		UDP glucuronosyltransferase family 1 member A complex locus	117	0.54	0.654		0.01	1	1
Hepatocarcinogenesis	C1512409	UTRN	7402	P46939	utrophin	152	0.525	0.692	1E-19	0.01	1	1
Hepatocarcinogenesis	C1512409	FAM189B	10712	P81408	family with sequence similarity 189 member B	6	0.89	0.154	8.1E-06	0.01	1	1
Hepatocarcinogenesis	C1512409	TRPV1	7442	Q8NER1	transient receptor potential cation channel subfamily V member 1	404	0.446	0.885	1E-16	0.01	1	1
Hepatocarcinogenesis	C1512409	WNT1	7471	P04628	Wnt family member 1	216	0.495	0.769	0.3263	0.01	1	1
Hepatocarcinogenesis	C1512409	WNT3	7473	P56703	Wnt family member 3	140	0.536	0.808	0.87515	0.01	1	1
Hepatocarcinogenesis	C1512409	WNT11	7481	O96014	Wnt family member 11	69	0.608	0.654	0.00644	0.01	1	1
Hepatocarcinogenesis	C1512409	XBP1	7494	P17861	X-box binding protein 1	234	0.477	0.846	0.03201	0.01	1	1
Hepatocarcinogenesis	C1512409	MTCO2P12	107075310		MT-CO2 pseudogene 12	703	0.368	0.962		0.01	1	1
Hepatocarcinogenesis	C1512409	NCOA3	8202	Q9Y6Q9	nuclear receptor coactivator 3	142	0.522	0.654	0.54065	0.01	1	1
Hepatocarcinogenesis	C1512409	SLU7	10569	O95391	SLU7 homolog, splicing factor	26	0.729	0.346	1.6E-09	0.01	1	1
Hepatocarcinogenesis	C1512409	CASP8	841	Q14790	caspase 8	480	0.404	0.923	3.7E-06	0.01	1	1
Hepatocarcinogenesis	C1512409	NR0B2	8431	Q15466	nuclear receptor subfamily 0 group B member 2	251	0.474	0.808	8.3E-06	0.01	1	1
Hepatocarcinogenesis	C1512409	SARNP	84324	P82979	SAP domain containing ribonucleoprotein	33	0.678	0.5	0.99574	0.01	1	1
Hepatocarcinogenesis	C1512409	GNPAT	8443	O15228	glyceronephosphate O-acyltransferase	44	0.663	0.462	0.30403	0.01	1	1
Hepatocarcinogenesis	C1512409	CUL4A	8451	Q13619	cullin 4A	79	0.597	0.654	0.99999	0.01	1	1
Hepatocarcinogenesis	C1512409	ZGPAT	84619	Q8N5A5	zinc finger CCCH-type and G-patch domain containing	54	0.653	0.654	0.00038	0.01	1	1
Hepatocarcinogenesis	C1512409	CAT	847	P04040	catalase	794	0.359	0.962	1.6E-10	0.01	1	1
Hepatocarcinogenesis	C1512409	USP30	84749	Q70CQ3	ubiquitin specific peptidase 30	9	0.821	0.154	8.3E-05	0.01	1	1
Hepatocarcinogenesis	C1512409	PYROXD2	84795	Q8N2H3	pyridine nucleotide-disulphide oxidoreductase domain 2	12	0.792	0.308	1E-15	0.01	1	1
Hepatocarcinogenesis	C1512409	HAVCR2	84868	Q8TDQ0	hepatitis A virus cellular receptor 2	299	0.45	0.808	0.03866	0.01	1	1
Hepatocarcinogenesis	C1512409	GEMIN2	8487	O14893	gem nuclear organelle associated protein 2	71	0.597	0.654	1.5E-06	0.01	1	1
Hepatocarcinogenesis	C1512409	MASTL	84930	Q96GX5	microtubule associated serine/threonine kinase like	35	0.674	0.385	5.4E-13	0.01	1	1
Hepatocarcinogenesis	C1512409	RITA1	84934	Q96K30	RBPJ interacting and tubulin associated 1	36	0.659	0.5	0.0038	0.01	1	1
Hepatocarcinogenesis	C1512409	FRMD5	84978	Q7Z6J6	FERM domain containing 5	18	0.805	0.192	0.99724	0.01	1	1
Hepatocarcinogenesis	C1512409	PAK4	10298	O96013	p21 (RAC1) activated kinase 4	81	0.584	0.731	4.4E-05	0.01	1	1
Hepatocarcinogenesis	C1512409	BHLHE40	8553	O14503	basic helix-loop-helix family member e40	113	0.544	0.731	0.99444	0.01	1	1
Hepatocarcinogenesis	C1512409	PLA2G6	8398	O60733	phospholipase A2 group VI	350	0.476	0.846	2.5E-10	0.01	1	1
Hepatocarcinogenesis	C1512409	IMMP2L	83943	Q96T52	inner mitochondrial membrane peptidase subunit 2	55	0.628	0.577	0.01117	0.01	1	1
Hepatocarcinogenesis	C1512409	FSD1L	83856	Q9BXM9	fibronectin type III and SPRY domain containing 1 like	216	0.488	0.808	0.23386	0.01	1	1
Hepatocarcinogenesis	C1512409	FZD7	8324	O75084	frizzled class receptor 7	70	0.601	0.538	0.08835	0.01	1	1
Hepatocarcinogenesis	C1512409	ARHGAP24	83478	Q8N264	Rho GTPase activating protein 24	273	0.456	0.808	3.9E-10	0.01	1	1
Hepatocarcinogenesis	C1512409	PRDX4	10549	Q13162	peroxiredoxin 4	59	0.619	0.692	0.02676	0.01	1	1
Hepatocarcinogenesis	C1512409	RASSF5	83593	Q8WWW0	Ras association domain family member 5	45	0.641	0.577	0.04335	0.01	1	1
Hepatocarcinogenesis	C1512409	SOX7	83595	Q9BT81	SRY-box transcription factor 7	75	0.599	0.577	3.5E-07	0.01	1	1
Hepatocarcinogenesis	C1512409	RTP3	83597	Q9BQQ7	receptor transporter protein 3	6	0.861	0.115	0.20085	0.01	1	1
Hepatocarcinogenesis	C1512409	ATG7	10533	O95352	autophagy related 7	179	0.505	0.769	7.4E-12	0.01	1	1
Hepatocarcinogenesis	C1512409	IPO7	10527	O95373	importin 7	24	0.729	0.269	1	0.01	1	1
Hepatocarcinogenesis	C1512409	IPO8	10526	O15397	importin 8	20	0.722	0.346	0.2344	0.01	1	1
Hepatocarcinogenesis	C1512409	FBXW10	10517	Q5XX13	F-box and WD repeat domain containing 10	6	0.931	0.115	1.8E-27	0.01	1	1
Hepatocarcinogenesis	C1512409	CEBPA	1050	P49715	CCAAT enhancer binding protein alpha	201	0.496	0.692	0.55365	0.01	1	1
Hepatocarcinogenesis	C1512409	FST	10468	P19883	follistatin	182	0.513	0.731	0.98014	0.01	1	1

Hepatocarcinogenesis	C1512409	ADARB1	104	P78563	adenosine deaminase RNA specific B1	85	0.588	0.654	0.80347	0.01	1	1
Hepatocarcinogenesis	C1512409	CITED2	10370	Q99967	300 interacting transactivator with Glu/Asp rich carboxy-terminal dc	105	0.576	0.654	0.76271	0.01	1	1
Hepatocarcinogenesis	C1512409	KLF2	10365	Q9Y5W3	Kruppel like factor 2	132	0.542	0.692	0.56044	0.01	1	1
Hepatocarcinogenesis	C1512409	ARMC10	83787	Q8N2F6	armadillo repeat containing 10	3	0.931	0.115	0.00012	0.01	1	1
Hepatocarcinogenesis	C1512409	DENR	8562	O43583	density regulated re-initiation and release factor	121	0.543	0.692	0.87514	0.01	1	1
Hepatocarcinogenesis	C1512409	MIR519D	574480		microRNA 519d	47	0.636	0.423		0.01	1	1
Hepatocarcinogenesis	C1512409	IFNA1	3439	P01562	interferon alpha 1	662	0.371	0.923		0.01	1	1
Hepatocarcinogenesis	C1512409	JUND	3727	P17535	JunD proto-oncogene, AP-1 transcription factor subunit	242	0.475	0.808	0.70054	0.01	1	1
Hepatocarcinogenesis	C1512409	RAB18	22931	Q9NP72	RAB18, member RAS oncogene family	113	0.582	0.692	0.91006	0.01	1	1
Hepatocarcinogenesis	C1512409	ATF6	22926	P18850	activating transcription factor 6	179	0.516	0.846	3.2E-07	0.01	1	1
Hepatocarcinogenesis	C1512409	ZHX2	22882	Q9Y6X8	zinc fingers and homeoboxes 2	172	0.51	0.654	0.03372	0.01	1	1
Hepatocarcinogenesis	C1512409	KDR	3791	P35968	kinase insert domain receptor	623	0.378	0.885	0.99982	0.01	1	1
Hepatocarcinogenesis	C1512409	ACAT1	38	P24752	acetyl-CoA acetyltransferase 1	176	0.538	0.692	9.9E-08	0.01	1	1
Hepatocarcinogenesis	C1512409	ARG1	383	P05089	arginase 1	273	0.476	0.846	0.00036	0.01	1	1
Hepatocarcinogenesis	C1512409	KRAS	3845	P01116	KRAS proto-oncogene, GTPase	1213	0.32	0.923	0.00079	0.01	1	1
Hepatocarcinogenesis	C1512409	KRT7	3855	P08729	keratin 7	287	0.463	0.808	3.1E-12	0.01	1	1
Hepatocarcinogenesis	C1512409	ZKSCAN4	387032	Q969J2	zinc finger with KRAB and SCAN domains 4	8	0.839	0.269	0.00014	0.01	1	1
Hepatocarcinogenesis	C1512409	TMEM189	387521	A5PLL7;Q13404	transmembrane protein 189	39	0.659	0.577	0.00839	0.01	1	1
Hepatocarcinogenesis	C1512409	MEM189-UBE2	387522	A5PLL7;Q13404	TMEM189-UBE2V1 readthrough	39	0.659	0.577	0.00839	0.01	1	1
Hepatocarcinogenesis	C1512409	KRT19	3880	P08727	keratin 19	270	0.46	0.731	0.00434	0.01	1	1
Hepatocarcinogenesis	C1512409	RTL1	388015	A6NKG5	retrotransposon Gag like 1	119	0.554	0.808	0.00561	0.01	1	1
Hepatocarcinogenesis	C1512409	HES5	388585	Q5TA89	hes family bHLH transcription factor 5	39	0.67	0.654	0.24882	0.01	1	1
Hepatocarcinogenesis	C1512409	LIN28B	389421	Q6ZN17	lin-28 homolog B	126	0.556	0.654	0.55513	0.01	1	1
Hepatocarcinogenesis	C1512409	JUNB	3726	P17275	JunB proto-oncogene, AP-1 transcription factor subunit	264	0.467	0.769	0.86299	0.01	1	1
Hepatocarcinogenesis	C1512409	JUN	3725	P05412	Jun proto-oncogene, AP-1 transcription factor subunit	344	0.442	0.885	0.05962	0.01	1	1
Hepatocarcinogenesis	C1512409	JAK2	3717	O60674	Janus kinase 2	644	0.385	0.885	0.65372	0.01	1	1
Hepatocarcinogenesis	C1512409	IFNA13	3447	P01562	interferon alpha 13	646	0.374	0.923		0.01	1	1
Hepatocarcinogenesis	C1512409	IFNB1	3456	P01574	interferon beta 1	426	0.421	0.846		0.01	1	1
Hepatocarcinogenesis	C1512409	FOXO3	2309	O43524	forkhead box O3	381	0.431	0.808	0.98806	0.01	1	1
Hepatocarcinogenesis	C1512409	TRIM35	23087	Q9UPQ4	tripartite motif containing 35	6	0.89	0.115	0.00088	0.01	1	1
Hepatocarcinogenesis	C1512409	IGFBP1	3484	P08833	insulin like growth factor binding protein 1	191	0.502	0.808	0.00095	0.01	1	1
Hepatocarcinogenesis	C1512409	IGFBP3	3486	P17936	insulin like growth factor binding protein 3	350	0.439	0.808	0.91029	0.01	1	1
Hepatocarcinogenesis	C1512409	IGFBP7	3490	Q16270	insulin like growth factor binding protein 7	241	0.476	0.769	0.00129	0.01	1	1
Hepatocarcinogenesis	C1512409	KDM4B	23030	O94953	lysine demethylase 4B	71	0.606	0.462	1	0.01	1	1
Hepatocarcinogenesis	C1512409	APRT	353	P07741	adenine phosphoribosyltransferase	384	0.423	0.846	5.5E-13	0.01	1	1
Hepatocarcinogenesis	C1512409	SPEN	23013	Q96T58	spen family transcriptional repressor	49	0.644	0.654	1	0.01	1	1
Hepatocarcinogenesis	C1512409	AKR7A3	22977	O95154	aldo-keto reductase family 7 member A3	6	0.931	0.115	1E-19	0.01	1	1
Hepatocarcinogenesis	C1512409	IL1RN	3557	P18510	interleukin 1 receptor antagonist	701	0.373	0.923	0.03091	0.01	1	1
Hepatocarcinogenesis	C1512409	DKK1	22943	O94907	dickkopf WNT signaling pathway inhibitor 1	372	0.439	0.885	0.17423	0.01	1	1
Hepatocarcinogenesis	C1512409	PCNAP1	359806		proliferating cell nuclear antigen pseudogene 1	9	0.821	0.154		0.01	1	1
Hepatocarcinogenesis	C1512409	ILK	3611	Q13418	integrin linked kinase	192	0.503	0.769	3.8E-05	0.01	1	1
Hepatocarcinogenesis	C1512409	ING2	3622	Q9H160	inhibitor of growth family member 2	76	0.599	0.654	0.72928	0.01	1	1
Hepatocarcinogenesis	C1512409	LAMA4	3910	Q16363	laminin subunit alpha 4	44	0.682	0.577	1.4E-24	0.01	1	1
Hepatocarcinogenesis	C1512409	ARHGAP1	392	Q07960	Rho GTPase activating protein 1	48	0.638	0.577	0.76774	0.01	1	1
Hepatocarcinogenesis	C1512409	LASP1	3927	Q14847	LIM and SH3 protein 1	85	0.572	0.615	0.9827	0.01	1	1
Hepatocarcinogenesis	C1512409	FGFR3	2261	P22607	fibroblast growth factor receptor 3	654	0.391	0.846	1.6E-05	0.01	1	1
Hepatocarcinogenesis	C1512409	FGFR1	2260	P11362	fibroblast growth factor receptor 1	816	0.362	0.885	0.99984	0.01	1	1
Hepatocarcinogenesis	C1512409	MIR15B	406949		microRNA 15b	134	0.532	0.769		0.01	1	1
Hepatocarcinogenesis	C1512409	MIR18A	406953		microRNA 18a	154	0.52	0.846		0.01	1	1
Hepatocarcinogenesis	C1512409	FGF2	2247	P09038	fibroblast growth factor 2	635	0.383	0.923	0.0184	0.01	1	1
Hepatocarcinogenesis	C1512409	MIR186	406962		microRNA 186	105	0.551	0.808		0.01	1	1
Hepatocarcinogenesis	C1512409	MIR193A	406968		microRNA 193a	116	0.541	0.731		0.01	1	1
Hepatocarcinogenesis	C1512409	MIR195	406971		microRNA 195	180	0.496	0.769		0.01	1	1
Hepatocarcinogenesis	C1512409	MIR200B	406984		microRNA 200b	180	0.5	0.769		0.01	1	1
Hepatocarcinogenesis	C1512409	MIR203A	406986		microRNA 203a	237	0.471	0.808		0.01	1	1

Hepatocarcinogenesis	C1512409	MIR205	406988		microRNA 205	210	0.482	0.846		0.01	1	1
Hepatocarcinogenesis	C1512409	MIR21	406991		microRNA 21	726	0.363	0.846		0.01	1	1
Hepatocarcinogenesis	C1512409	FER	2241	P16591	FER tyrosine kinase	64	0.628	0.615	0.6633	0.01	1	1
Hepatocarcinogenesis	C1512409	MIR216A	406998		microRNA 216a	79	0.582	0.654		0.01	1	1
Hepatocarcinogenesis	C1512409	PTK2B	2185	Q14289	protein tyrosine kinase 2 beta	319	0.448	0.808	0.93536	0.01	1	1
Hepatocarcinogenesis	C1512409	ACSL4	2182	O60488	acyl-CoA synthetase long chain family member 4	136	0.561	0.769	0.98103	0.01	1	1
Hepatocarcinogenesis	C1512409	MIR148A	406940		microRNA 148a	172	0.506	0.846		0.01	1	1
Hepatocarcinogenesis	C1512409	MIR141	406933		microRNA 141	179	0.497	0.808		0.01	0	1
Hepatocarcinogenesis	C1512409	MIR139	406931		microRNA 139	138	0.527	0.808		0.01	1	1
Hepatocarcinogenesis	C1512409	LCN2	3934	P80188	lipocalin 2	497	0.405	0.885	1.1E-09	0.01	1	1
Hepatocarcinogenesis	C1512409	LECT2	3950	O14960	leukocyte cell derived chemotaxin 2	47	0.653	0.5	0.00148	0.01	1	1
Hepatocarcinogenesis	C1512409	FHIT	2272	P49789	fragile histidine triad diadenosine triphosphatase	337	0.444	0.846	0.00571	0.01	1	1
Hepatocarcinogenesis	C1512409	LIG4	3981	P49917	DNA ligase 4	293	0.478	0.846	4.2E-06	0.01	1	1
Hepatocarcinogenesis	C1512409	HAGLR	401022		HOXD antisense growth-associated long non-coding RNA	55	0.619	0.385		0.01	1	1
Hepatocarcinogenesis	C1512409	CASC15	401237		cancer susceptibility 15	90	0.603	0.577		0.01	1	1
Hepatocarcinogenesis	C1512409	LRP6	4040	O75581	LDL receptor related protein 6	134	0.536	0.808	0.69664	0.01	1	1
Hepatocarcinogenesis	C1512409	GTF2H5	404672	Q6ZYL4	general transcription factor IIH subunit 5	152	0.556	0.769	0.04524	0.01	1	1
Hepatocarcinogenesis	C1512409	LTBR	4055	P36941	lymphotoxin beta receptor	78	0.592	0.692	0.3058	0.01	1	1
Hepatocarcinogenesis	C1512409	MIRLET7C	406885		microRNA let-7c	154	0.514	0.808		0.01	1	1
Hepatocarcinogenesis	C1512409	MIRLET7I	406891		microRNA let-7i	86	0.575	0.615		0.01	0	1
Hepatocarcinogenesis	C1512409	MIR100	406892		microRNA 100	146	0.521	0.846		0.01	1	1
Hepatocarcinogenesis	C1512409	MIR107	406901		microRNA 107	123	0.543	0.769		0.01	1	1
Hepatocarcinogenesis	C1512409	MIR10A	406902		microRNA 10a	158	0.513	0.846		0.01	0	1
Hepatocarcinogenesis	C1512409	MIR129-2	406918		microRNA 129-2	43	0.653	0.654		0.01	1	1
Hepatocarcinogenesis	C1512409	MIR132	406921		microRNA 132	222	0.485	0.846		0.01	1	1
Hepatocarcinogenesis	C1512409	MIR224	407009		microRNA 224	128	0.534	0.654		0.01	1	1
Hepatocarcinogenesis	C1512409	LPIN1	23175	Q14693	lipin 1	86	0.601	0.692	6.5E-13	0.01	1	1
Hepatocarcinogenesis	C1512409	RPL36	25873	Q9Y3U8	ribosomal protein L36	8	0.839	0.231	0.67802	0.01	1	1
Hepatocarcinogenesis	C1512409	CADM1	23705	Q9BY67	cell adhesion molecule 1	205	0.491	0.769	0.66216	0.01	1	1
Hepatocarcinogenesis	C1512409	RABGEF1	27342	Q9UJ41	RAB guanine nucleotide exchange factor 1	122	0.55	0.731	0.00063	0.01	1	1
Hepatocarcinogenesis	C1512409	SGSM3	27352	Q96HU1	small G protein signaling modulator 3	259	0.469	0.808	3.4E-15	0.01	1	1
Hepatocarcinogenesis	C1512409	BIN1	274	O00499	bridging integrator 1	163	0.55	0.692	0.77822	0.01	1	1
Hepatocarcinogenesis	C1512409	CCR10	2826	P46092	C-C motif chemokine receptor 10	57	0.626	0.654	0.00051	0.01	1	1
Hepatocarcinogenesis	C1512409	SH3BP4	23677	Q9P0V3	SH3 domain binding protein 4	74	0.593	0.692	0.00253	0.01	1	1
Hepatocarcinogenesis	C1512409	NEAT1	283131		nuclear paraspeckle assembly transcript 1	179	0.502	0.769		0.01	1	1
Hepatocarcinogenesis	C1512409	SNHG10	283596		small nucleolar RNA host gene 10	4	0.89	0.154		0.01	1	1
Hepatocarcinogenesis	C1512409	CISD3	284106	P0C7P0	CDGSH iron sulfur domain 3	24	0.711	0.577	0.01982	0.01	1	1
Hepatocarcinogenesis	C1512409	ZNF763	284390	Q0D2J5	zinc finger protein 763	35	0.691	0.5	0.01827	0.01	1	1
Hepatocarcinogenesis	C1512409	RABL3	285282	Q5HYI8	RAB, member of RAS oncogene family like 3	22	0.751	0.231	0.0008	0.01	1	1
Hepatocarcinogenesis	C1512409	ATG9B	285973	Q674R7	autophagy related 9B	8	0.792	0.269	1.3E-17	0.01	1	1
Hepatocarcinogenesis	C1512409	LIN9	286826	Q5TKA1	lin-9 DREAM MuvB core complex component	20	0.743	0.346	0.99771	0.01	1	1
Hepatocarcinogenesis	C1512409	DYNLL2	140735	Q96FJ2	dynein light chain LC8-type 2	22	0.743	0.269	0.74311	0.01	1	1
Hepatocarcinogenesis	C1512409	CCNDBP1	23582	O95273	cyclin D1 binding protein 1	21	0.729	0.385	0.0003	0.01	1	1
Hepatocarcinogenesis	C1512409	GRN	2896	P28799	granulin precursor	412	0.435	0.846	0.06967	0.01	1	1
Hepatocarcinogenesis	C1512409	GNMT	27232	Q14749	glycine N-methyltransferase	57	0.626	0.462	0.00282	0.01	1	1
Hepatocarcinogenesis	C1512409	GPC3	2719	P51654	glypican 3	311	0.466	0.808	0.9982	0.01	1	1
Hepatocarcinogenesis	C1512409	DKK2	27123	Q9UBU2	dickkopf WNT signaling pathway inhibitor 2	74	0.599	0.692	0.75318	0.01	1	1
Hepatocarcinogenesis	C1512409	ZBTB20	26137	Q9HC78	zinc finger and BTB domain containing 20	139	0.566	0.731	0.97382	0.01	1	1
Hepatocarcinogenesis	C1512409	TIPRL	261726	O75663	TOR signaling pathway regulator	24	0.716	0.423	0.95372	0.01	1	1
Hepatocarcinogenesis	C1512409	GAS2	2620	O43903	growth arrest specific 2	10	0.792	0.269	0.0008	0.01	1	1
Hepatocarcinogenesis	C1512409	GAS8	2622	O95995	growth arrest specific 8	60	0.636	0.654	1.1E-12	0.01	1	1
Hepatocarcinogenesis	C1512409	FBXL5	26234	Q9UKA1	F-box and leucine rich repeat protein 5	27	0.711	0.308	0.99854	0.01	1	1
Hepatocarcinogenesis	C1512409	VPS33B	26276	Q9H267	VPS33B late endosome and lysosome associated	55	0.644	0.654	2.4E-16	0.01	1	1
Hepatocarcinogenesis	C1512409	TINF2	26277	Q9BSI4	TERF1 interacting nuclear factor 2	158	0.575	0.731	0.00127	0.01	1	1
Hepatocarcinogenesis	C1512409	GC	2638	P02774	GC vitamin D binding protein	219	0.489	0.885	2.7E-11	0.01	1	1

Hepatocarcinogenesis	C1512409	LHX6	26468	Q9UPM6	LIM homeobox 6	22	0.736	0.346	0.96572	0.01	1	1
Hepatocarcinogenesis	C1512409	RASGRP3	25780	Q8IV61	RAS guanyl releasing protein 3	34	0.686	0.5	0.00911	0.01	1	1
Hepatocarcinogenesis	C1512409	NAP1L5	266812	Q96NT1	nucleosome assembly protein 1 like 5	5	0.89	0.154	0.6928	0.01	1	1
Hepatocarcinogenesis	C1512409	ADGRF1	266977	Q5T601	adhesion G protein-coupled receptor F1	19	0.736	0.308	2E-22	0.01	1	1
Hepatocarcinogenesis	C1512409	GGT1	2678	P19440	gamma-glutamyltransferase 1	209	0.496	0.885	7.5E-05	0.01	1	1
Hepatocarcinogenesis	C1512409	SND1	27044	Q7KZF4	staphylococcal nuclease and tudor domain containing 1	101	0.576	0.769	0.98213	0.01	1	1
Hepatocarcinogenesis	C1512409	RICTOR	253260	Q6R327	RPTOR independent companion of MTOR complex 2	82	0.592	0.615	1	0.01	1	1
Hepatocarcinogenesis	C1512409	DKK4	27121	Q9UBT3	dickkopf WNT signaling pathway inhibitor 4	51	0.647	0.5	0.00077	0.01	0	1
Hepatocarcinogenesis	C1512409	PADI4	23569	Q9UM07	peptidyl arginine deiminase 4	196	0.494	0.808	4.6E-16	0.01	1	1
Hepatocarcinogenesis	C1512409	FOSB	2354	P53539	FosB proto-oncogene, AP-1 transcription factor subunit	278	0.463	0.846	0.97553	0.01	1	1
Hepatocarcinogenesis	C1512409	CD274	29126	Q9NZQ7	CD274 molecule	1011	0.324	0.923	0.01916	0.01	1	1
Hepatocarcinogenesis	C1512409	HMGB3	3149	O15347	high mobility group box 3	71	0.604	0.615	0.79758	0.01	1	1
Hepatocarcinogenesis	C1512409	HMGA1	3159	P17096	high mobility group AT-hook 1	206	0.494	0.769	0.83209	0.01	1	1
Hepatocarcinogenesis	C1512409	NR4A1	3164	P22736	nuclear receptor subfamily 4 group A member 1	216	0.49	0.731	0.37074	0.01	1	1
Hepatocarcinogenesis	C1512409	HNF4A	3172	P41235	hepatocyte nuclear factor 4 alpha	340	0.461	0.808	0.15285	0.01	1	1
Hepatocarcinogenesis	C1512409	APBA1	320	Q02410	amyloid beta precursor protein binding family A member 1	21	0.743	0.346	0.53701	0.01	1	1
Hepatocarcinogenesis	C1512409	HOXB7	3217	P09629	homeobox B7	93	0.57	0.615	0.00028	0.01	1	1
Hepatocarcinogenesis	C1512409	ZNF629	23361	Q9UEG4	zinc finger protein 629	34	0.691	0.5	0.99978	0.01	1	1
Hepatocarcinogenesis	C1512409	HP	3240	P00738	haptoglobin	464	0.412	0.885	1.7E-05	0.01	1	1
Hepatocarcinogenesis	C1512409	HPGD	3248	P15428	15-hydroxyprostaglandin dehydrogenase	194	0.516	0.731	1.4E-05	0.01	1	1
Hepatocarcinogenesis	C1512409	HPN	3249	P05981	hepsin	57	0.641	0.577	0.99911	0.01	1	1
Hepatocarcinogenesis	C1512409	HRAS	3265	P01112	HRas proto-oncogene, GTPase	698	0.378	0.885	0.07977	0.01	1	1
Hepatocarcinogenesis	C1512409	APEX1	328	P27695	apurinic/apyrimidinic endodeoxyribonuclease 1	305	0.458	0.846	1.9E-07	0.01	1	1
Hepatocarcinogenesis	C1512409	HES1	3280	Q14469	hes family bHLH transcription factor 1	243	0.468	0.885	0.7072	0.01	1	1
Hepatocarcinogenesis	C1512409	FN1	2335	P02751	fibronectin 1	724	0.365	0.962	0.0014	0.01	1	1
Hepatocarcinogenesis	C1512409	FLT4	2324	P35916	fms related receptor tyrosine kinase 4	306	0.457	0.808	1	0.01	1	1
Hepatocarcinogenesis	C1512409	HSP90AA1	3320	P07900	heat shock protein 90 alpha family class A member 1	455	0.411	0.923	0.86025	0.01	1	1
Hepatocarcinogenesis	C1512409	HMGB1	3146	P09429	high mobility group box 1	724	0.368	0.923	0.82035	0.01	1	1
Hepatocarcinogenesis	C1512409	HINT1	3094	P49773	histidine triad nucleotide binding protein 1	105	0.619	0.577	0.00182	0.01	1	1
Hepatocarcinogenesis	C1512409	DICER1	23405	Q9UPY3	dicer 1, ribonuclease III	302	0.446	0.846	1	0.01	1	1
Hepatocarcinogenesis	C1512409	GSTM1	2944	P09488	glutathione S-transferase mu 1	627	0.38	0.923	0.00206	0.01	1	1
Hepatocarcinogenesis	C1512409	GSTZ1	2954	O43708	glutathione S-transferase zeta 1	38	0.659	0.538	5.5E-11	0.01	1	1
Hepatocarcinogenesis	C1512409	CBX5	23468	P45973	chromobox 5	59	0.621	0.731	0.93174	0.01	1	1
Hepatocarcinogenesis	C1512409	GTF2H2	2966	Q13888	general transcription factor IIH subunit 2	46	0.65	0.577	0.01552	0.01	1	1
Hepatocarcinogenesis	C1512409	GTF2H3	2967	Q13889	general transcription factor IIH subunit 3	41	0.656	0.615	3E-08	0.01	1	1
Hepatocarcinogenesis	C1512409	GTF2H4	2968	Q92759	general transcription factor IIH subunit 4	87	0.573	0.846	4.2E-07	0.01	1	1
Hepatocarcinogenesis	C1512409	SENP1	29843	Q9P0U3	SUMO specific peptidase 1	79	0.593	0.654	0.99358	0.01	1	1
Hepatocarcinogenesis	C1512409	HCFC2	29915	Q9Y5Z7	host cell factor C2	3	1	0.077	0.99989	0.01	1	1
Hepatocarcinogenesis	C1512409	SERPIND1	3053	P05546	serpin family D member 1	60	0.619	0.654	2.5E-10	0.01	1	1
Hepatocarcinogenesis	C1512409	HCFC1	3054	P51610	host cell factor C1	100	0.601	0.692	1	0.01	1	1
Hepatocarcinogenesis	C1512409	HDGF	3068	P51858	heparin binding growth factor	89	0.575	0.654	0.27272	0.01	1	1
Hepatocarcinogenesis	C1512409	ANXA5	308	P08758	annexin A5	283	0.458	0.769	3.8E-15	0.01	1	1
Hepatocarcinogenesis	C1512409	SIRT5	23408	Q9NXA8	sirtuin 5	63	0.61	0.615	8.7E-10	0.01	1	1
Hepatocarcinogenesis	C1512409	ZNRD1	30834	Q9P1U0	zinc ribbon domain containing 1	50	0.653	0.654	0.2067	0.01	1	1
Hepatocarcinogenesis	C1512409	ANXA6	309	P08133	annexin A6	156	0.514	0.846	1.1E-08	0.01	1	1
Hepatocarcinogenesis	C1512409	HIC1	3090	Q14526	HIC ZBTB transcriptional repressor 1	163	0.522	0.615	0.13002	0.01	1	1
Hepatocarcinogenesis	C1512409	ZNF774	342132	Q6NX45	zinc finger protein 774	3	0.931	0.115	2.3E-15	0.01	1	1
Hepatocarcinogenesis	C1512409	TRPV2	51393	Q9Y5S1	transient receptor potential cation channel subfamily V member 2	90	0.581	0.615	7.9E-13	0.01	1	1
Hepatocarcinogenesis	C1512409	ACP5	54	P13686	acid phosphatase 5, tartrate resistant	130	0.561	0.731	0.07865	0.01	1	1
Hepatocarcinogenesis	C1512409	RIPK4	54101	P57078	receptor interacting serine/threonine kinase 4	119	0.565	0.654	0.00396	0.01	1	1
Hepatocarcinogenesis	C1512409	TERF2IP	54386	Q9NYB0	TERF2 interacting protein	126	0.548	0.731	2.7E-05	0.01	1	1
Hepatocarcinogenesis	C1512409	UGT1A7	54577	Q9HAW7	UDP glucuronosyltransferase family 1 member A7	125	0.55	0.692	8.7E-14	0.01	1	1
Hepatocarcinogenesis	C1512409	CYP1A1	1543	P04798	cytochrome P450 family 1 subfamily A member 1	379	0.436	0.846	1.1E-17	0.01	1	1
Hepatocarcinogenesis	C1512409	KLB	152831	Q86Z14	klotho beta	53	0.647	0.5	0.03643	0.01	1	1
Hepatocarcinogenesis	C1512409	PGPEP1	54858	Q9NXJ5	pyroglutamyl-peptidase I	116	0.581	0.769	0.0085	0.01	1	1

Hepatocarcinogenesis	C1512409	TMEM70	54968	Q9BUB7	transmembrane protein 70	83	0.636	0.654	0.02929	0.01	1	1
Hepatocarcinogenesis	C1512409	TMEM132A	54972	Q24JP5	transmembrane protein 132A	15	0.792	0.308	2E-09	0.01	1	1
Hepatocarcinogenesis	C1512409	PPOX	5498	P50336	protoporphyrinogen oxidase	53	0.678	0.5	0.02829	0.01	1	1
Hepatocarcinogenesis	C1512409	PINX1	54984	Q96BK5	PIN2 (TERF1) interacting telomerase inhibitor 1	88	0.587	0.692	4.6E-09	0.01	1	1
Hepatocarcinogenesis	C1512409	PPP2CA	5515	P67775	protein phosphatase 2 catalytic subunit alpha	87	0.608	0.769	0.9893	0.01	1	1
Hepatocarcinogenesis	C1512409	DARS2	55157	Q6PI48	aspartyl-tRNA synthetase 2, mitochondrial	92	0.638	0.577	9.8E-13	0.01	1	1
Hepatocarcinogenesis	C1512409	PRMT6	55170	Q96LA8	protein arginine methyltransferase 6	46	0.67	0.462	0.00334	0.01	1	1
Hepatocarcinogenesis	C1512409	PPP2R1A	5518	P30153	protein phosphatase 2 scaffold subunit Aalpha	104	0.588	0.577	0.98389	0.01	1	1
Hepatocarcinogenesis	C1512409	MAP1S	55201	Q66K74	microtubule associated protein 1S	28	0.686	0.5	0.4197	0.01	1	1
Hepatocarcinogenesis	C1512409	DUOX1	53905	Q9NRD9	dual oxidase 1	97	0.57	0.692	6.2E-33	0.01	1	1
Hepatocarcinogenesis	C1512409	CYP3A4	1576	P08684	cytochrome P450 family 3 subfamily A member 4	291	0.462	0.885	5.8E-11	0.01	1	1
Hepatocarcinogenesis	C1512409	PLK1	5347	P53350	polo like kinase 1	253	0.467	0.808	0.97851	0.01	1	1
Hepatocarcinogenesis	C1512409	SIRT7	51547	Q9NRC8	sirtuin 7	80	0.582	0.577	0.00056	0.01	1	1
Hepatocarcinogenesis	C1512409	DNMT3B	1789	Q9UBC3	DNA methyltransferase 3 beta	315	0.453	0.846	0.19964	0.01	1	1
Hepatocarcinogenesis	C1512409	EEF1AKNMT	51603	Q8N6R0	eEF1A lysine and N-terminal methyltransferase	26	0.729	0.269	1.6E-09	0.01	1	1
Hepatocarcinogenesis	C1512409	AGER	177	Q15109	advanced glycosylation end-product specific receptor	450	0.42	0.885	6.4E-16	0.01	1	1
Hepatocarcinogenesis	C1512409	RSF1	51773	Q96T23	remodeling and spacing factor 1	46	0.647	0.423	1	0.01	1	1
Hepatocarcinogenesis	C1512409	PGAM1	5223	P18669	phosphoglycerate mutase 1	62	0.621	0.615	2.6E-07	0.01	1	1
Hepatocarcinogenesis	C1512409	DLAT	1737	P10515	dihydrolipoamide S-acetyltransferase	93	0.576	0.731	2.3E-08	0.01	1	1
Hepatocarcinogenesis	C1512409	OIT3	170392	Q8WWZ8	oncoprotein induced transcript 3	8	0.821	0.154	3.4E-11	0.01	1	1
Hepatocarcinogenesis	C1512409	DECR1	1666	Q16698	2,4-dienoyl-CoA reductase 1	399	0.426	0.885	4.5E-14	0.01	1	1
Hepatocarcinogenesis	C1512409	DDX3X	1654	O00571	DEAD-box helicase 3 X-linked	180	0.537	0.769	0.99986	0.01	1	1
Hepatocarcinogenesis	C1512409	DCN	1634	P07585	decorin	297	0.457	0.808	0.31231	0.01	1	1
Hepatocarcinogenesis	C1512409	DAPK3	1613	O43293	death associated protein kinase 3	72	0.612	0.654	0.00655	0.01	1	1
Hepatocarcinogenesis	C1512409	PITX1	5307	P78337	paired like homeodomain 1	109	0.561	0.654	0.88244	0.01	1	1
Hepatocarcinogenesis	C1512409	DAPK1	1612	P53355	death associated protein kinase 1	249	0.472	0.808	0.99767	0.01	1	1
Hepatocarcinogenesis	C1512409	PLAGL1	5325	Q9UM63	PLAG1 like zinc finger 1	130	0.566	0.769	0.9764	0.01	1	1
Hepatocarcinogenesis	C1512409	PLAU	5328	P00749	plasminogen activator, urokinase	439	0.425	0.923	2.3E-06	0.01	1	1
Hepatocarcinogenesis	C1512409	MOB1A	55233	Q9H8S9	MOB kinase activator 1A	21	0.736	0.269	0.95091	0.01	1	1
Hepatocarcinogenesis	C1512409	PTPA	5524	Q15257	protein phosphatase 2 phosphatase activator	245	0.48	0.846	0.99057	0.01	1	1
Hepatocarcinogenesis	C1512409	CX3CR1	1524	P49238	C-X3-C motif chemokine receptor 1	310	0.457	0.885	0.06137	0.01	1	1
Hepatocarcinogenesis	C1512409	ZNF253	56242	O75346	zinc finger protein 253	21	0.736	0.346	0.00321	0.01	1	1
Hepatocarcinogenesis	C1512409	PROX1	5629	Q92786	prospero homeobox 1	114	0.565	0.538	0.99884	0.01	1	1
Hepatocarcinogenesis	C1512409	LGMN	5641	Q99538	legumain	95	0.579	0.731	0.00041	0.01	1	1
Hepatocarcinogenesis	C1512409	GPR137	56834	Q96N19	G protein-coupled receptor 137	32	0.686	0.231	0.66798	0.01	1	1
Hepatocarcinogenesis	C1512409	PRDM8	56978	Q9NQV8	PR/SET domain 8	39	0.678	0.462	0.77626	0.01	1	1
Hepatocarcinogenesis	C1512409	PARP1	142	P09874	poly(ADP-ribose) polymerase 1	565	0.389	0.923	0.00033	0.01	1	1
Hepatocarcinogenesis	C1512409	CRY2	1408	Q49AN0	cryptochrome circadian regulator 2	76	0.599	0.692	0.00032	0.01	1	1
Hepatocarcinogenesis	C1512409	CRY1	1407	Q16526	cryptochrome circadian regulator 1	91	0.585	0.769	1.7E-09	0.01	1	1
Hepatocarcinogenesis	C1512409	SALL4	57167	Q9UJQ4	spalt like transcription factor 4	245	0.489	0.769	0.99997	0.01	1	1
Hepatocarcinogenesis	C1512409	CRMP1	1400	Q14194	collapsin response mediator protein 1	148	0.525	0.808	0.99173	0.01	1	1
Hepatocarcinogenesis	C1512409	MIR363	574031		microRNA 363	83	0.578	0.692		0.01	1	1
Hepatocarcinogenesis	C1512409	FUNDC1	139341	Q8IVP5	FUN14 domain containing 1	29	0.686	0.385	0.04205	0.01	1	1
Hepatocarcinogenesis	C1512409	MIR511	574445		microRNA 511	45	0.647	0.538		0.01	1	1
Hepatocarcinogenesis	C1512409	MIR493	574450		microRNA 493	52	0.626	0.538		0.01	1	1
Hepatocarcinogenesis	C1512409	MIR497	574456		microRNA 497	116	0.539	0.808		0.01	1	1
Hepatocarcinogenesis	C1512409	MIR181D	574457		microRNA 181d	44	0.641	0.654		0.01	1	1
Hepatocarcinogenesis	C1512409	PRL	5617	P01236	prolactin	506	0.406	0.885	2.9E-07	0.01	1	1
Hepatocarcinogenesis	C1512409	EIF2AK2	5610	P19525	eukaryotic translation initiation factor 2 alpha kinase 2	142	0.538	0.769	0.01764	0.01	1	1
Hepatocarcinogenesis	C1512409	MAP2K6	5608	P52564	mitogen-activated protein kinase kinase 6	59	0.631	0.615	0.91334	0.01	1	1
Hepatocarcinogenesis	C1512409	MEG3	55384		maternally expressed 3	239	0.471	0.846		0.01	1	1
Hepatocarcinogenesis	C1512409	CTSE	1510	P14091	cathepsin E	56	0.631	0.615	1.4E-14	0.01	1	1
Hepatocarcinogenesis	C1512409	UBE2Q1	55585	Q7Z7E8	ubiquitin conjugating enzyme E2 Q1	19	0.76	0.308	0.99919	0.01	1	1
Hepatocarcinogenesis	C1512409	PRKAA1	5562	Q13131	protein kinase AMP-activated catalytic subunit alpha 1	325	0.453	0.846	0.13725	0.01	1	1
Hepatocarcinogenesis	C1512409	PRKAA2	5563	P54646	protein kinase AMP-activated catalytic subunit alpha 2	317	0.456	0.846	3.5E-08	0.01	1	1

Hepatocarcinogenesis	C1512409	PRKAB1	5564	Q9Y478	protein kinase AMP-activated non-catalytic subunit beta 1	296	0.46	0.846	0.00526	0.01	1	1
Hepatocarcinogenesis	C1512409	SIK1	150094	0A0B4J2F2;P5705	salt inducible kinase 1	157	0.544	0.731	0.93716	0.01	1	1
Hepatocarcinogenesis	C1512409	CTNND1	1500	O60716	catenin delta 1	196	0.5	0.808	0.99999	0.01	1	1
Hepatocarcinogenesis	C1512409	PRKCA	5578	P17252	protein kinase C alpha	368	0.44	0.846	0.3677	0.01	1	1
Hepatocarcinogenesis	C1512409	CAND1	55832	Q86VP6	cullin associated and neddylation dissociated 1	22	0.729	0.346	1	0.01	1	1
Hepatocarcinogenesis	C1512409	PRKDC	5591	P78527	protein kinase, DNA-activated, catalytic subunit	131	0.54	0.808	1	0.01	1	1
Hepatocarcinogenesis	C1512409	ZNF569	148266	Q5MCW4	zinc finger protein 569	42	0.666	0.538	6.6E-08	0.01	1	1
Hepatocarcinogenesis	C1512409	MAPK3	5595	P27361	mitogen-activated protein kinase 3	647	0.379	0.885	0.03688	0.01	1	1
Hepatocarcinogenesis	C1512409	PIWIL4	143689	Q7Z3Z4	piwi like RNA-mediated gene silencing 4	83	0.579	0.692	1.8E-06	0.01	1	1
Hepatocarcinogenesis	C1512409	CSE1L	1434	P55060	chromosome segregation 1 like	163	0.521	0.808	1	0.01	1	1
Hepatocarcinogenesis	C1512409	MAP2K1	5604	Q02750	mitogen-activated protein kinase kinase 1	389	0.439	0.846	0.89669	0.01	1	1
Hepatocarcinogenesis	C1512409	NDRG3	57446	Q9UGV2	NDRG family member 3	24	0.722	0.308	0.36564	0.01	1	1
Hepatocarcinogenesis	C1512409	ZMYND10	51364	O75800	zinc finger MYND-type containing 10	126	0.553	0.731	2.3E-07	0.01	1	1
Hepatocarcinogenesis	C1512409	MIR302A	407028		microRNA 302a	65	0.601	0.5		0.01	0	1
Hepatocarcinogenesis	C1512409	ERBB2	2064	P04626	erb-b2 receptor tyrosine kinase 2	995	0.328	0.923	0.00599	0.01	1	1
Hepatocarcinogenesis	C1512409	MLH1	4292	P40692	mutL homolog 1	526	0.399	0.808	3.4E-05	0.01	1	1
Hepatocarcinogenesis	C1512409	NR3C2	4306	P08235	nuclear receptor subfamily 3 group C member 2	287	0.469	0.846	0.83739	0.01	1	1
Hepatocarcinogenesis	C1512409	MMP7	4316	P09237	matrix metallopeptidase 7	320	0.446	0.885	1E-09	0.01	1	1
Hepatocarcinogenesis	C1512409	MPG	4350	P29372	N-methylpurine DNA glycosylase	74	0.597	0.731	1.9E-12	0.01	1	1
Hepatocarcinogenesis	C1512409	EPHX1	2052	P07099	epoxide hydrolase 1	196	0.5	0.846	3.2E-09	0.01	0	1
Hepatocarcinogenesis	C1512409	MIR330	442902		microRNA 330	81	0.584	0.692		0.01	1	1
Hepatocarcinogenesis	C1512409	MIR338	442906		microRNA 338	128	0.535	0.808		0.01	1	1
Hepatocarcinogenesis	C1512409	MIR371A	442916		microRNA 371a	49	0.647	0.5		0.01	1	1
Hepatocarcinogenesis	C1512409	EPHB2	2048	P29323	EPH receptor B2	649	0.374	0.846	0.99997	0.01	1	1
Hepatocarcinogenesis	C1512409	COX2	4513	P00403	cytochrome c oxidase subunit II	875	0.352	0.962		0.01	1	1
Hepatocarcinogenesis	C1512409	NUDT1	4521	P36639	nudix hydrolase 1	73	0.597	0.769	7.4E-12	0.01	1	1
Hepatocarcinogenesis	C1512409	MUC1	4582	P15941	mucin 1, cell surface associated	594	0.384	0.885	0.01877	0.01	1	1
Hepatocarcinogenesis	C1512409	MUTYH	4595	Q9UIF7	mutY DNA glycosylase	156	0.521	0.769	1.3E-18	0.01	1	1
Hepatocarcinogenesis	C1512409	ENO1	2023	P06733	enolase 1	231	0.481	0.885	1.9E-05	0.01	1	1
Hepatocarcinogenesis	C1512409	ELANE	1991	P08246	elastase, neutrophil expressed	346	0.447	0.846	0.00123	0.01	1	1
Hepatocarcinogenesis	C1512409	MFAP1	4236	P55081	microfibril associated protein 1	357	0.431	0.846	0.79602	0.01	1	1
Hepatocarcinogenesis	C1512409	ERCC2	2068	P18074	ERCC excision repair 2, TFIIH core complex helicase subunit	499	0.42	0.846	7.1E-20	0.01	1	1
Hepatocarcinogenesis	C1512409	MEN1	4221	O00255	menin 1	364	0.444	0.808	0.99971	0.01	1	1
Hepatocarcinogenesis	C1512409	MIR30B	407030		microRNA 30b	109	0.555	0.808		0.01	1	1
Hepatocarcinogenesis	C1512409	ALDH2	217	P05091	aldehyde dehydrogenase 2 family member	337	0.457	0.885	3.4E-10	0.01	1	1
Hepatocarcinogenesis	C1512409	MIR31	407035		microRNA 31	235	0.471	0.769		0.01	1	1
Hepatocarcinogenesis	C1512409	FABP4	2167	P15090	fatty acid binding protein 4	194	0.5	0.808	5.2E-05	0.01	1	1
Hepatocarcinogenesis	C1512409	MIR95	407052		microRNA 95	38	0.663	0.577		0.01	1	1
Hepatocarcinogenesis	C1512409	MECOM	2122	Q03112	MDS1 and EVI1 complex locus	191	0.513	0.731	1	0.01	1	1
Hepatocarcinogenesis	C1512409	MIR17HG	407975	Q75NE6	miR-17-92a-1 cluster host gene	224	0.483	0.808		0.01	1	1
Hepatocarcinogenesis	C1512409	SMAD2	4087	Q15796	SMAD family member 2	289	0.456	0.808	0.99661	0.01	1	1
Hepatocarcinogenesis	C1512409	SMAD3	4088	P84022	SMAD family member 3	470	0.415	0.923	0.79796	0.01	1	1
Hepatocarcinogenesis	C1512409	MAN2A1	4124	Q16706	mannosidase alpha class 2A member 1	28	0.705	0.538	1.6E-10	0.01	1	1
Hepatocarcinogenesis	C1512409	ETS1	2113	P14921	ETS proto-oncogene 1, transcription factor	327	0.444	0.731	0.7816	0.01	1	1
Hepatocarcinogenesis	C1512409	MAZ	4150	P56270	MYC associated zinc finger protein	60	0.617	0.654	0.94363	0.01	1	1
Hepatocarcinogenesis	C1512409	PIM3	415116	Q86V86	Pim-3 proto-oncogene, serine/threonine kinase	62	0.621	0.654	0.98192	0.01	1	1
Hepatocarcinogenesis	C1512409	MCL1	4170	Q07820	MCL1 apoptosis regulator, BCL2 family member	375	0.43	0.808	0.96349	0.01	1	1
Hepatocarcinogenesis	C1512409	ERCC3	2071	P19447	ERCC excision repair 3, TFIIH core complex helicase subunit	253	0.504	0.846	3.4E-11	0.01	1	1
Hepatocarcinogenesis	C1512409	MAP3K5	4217	Q99683	mitogen-activated protein kinase kinase kinase 5	170	0.513	0.808	0.51964	0.01	1	1
Hepatocarcinogenesis	C1512409	EIF4EBP1	1978	Q13541	eukaryotic translation initiation factor 4E binding protein 1	201	0.494	0.885	0.20762	0.01	1	1
Hepatocarcinogenesis	C1512409	NFATC1	4772	O95644	nuclear factor of activated T cells 1	161	0.517	0.731	0.15652	0.01	1	1
Hepatocarcinogenesis	C1512409	EIF4E	1977	P06730	eukaryotic translation initiation factor 4E	317	0.448	0.846	0.94133	0.01	1	1
Hepatocarcinogenesis	C1512409	PAX5	5079	Q02548	paired box 5	199	0.487	0.654	0.99798	0.01	1	1
Hepatocarcinogenesis	C1512409	DUSP1	1843	P28562	dual specificity phosphatase 1	214	0.491	0.808	0.01015	0.01	1	1
Hepatocarcinogenesis	C1512409	HBEGF	1839	Q99075	heparin binding EGF like growth factor	155	0.52	0.769	0.07563	0.01	1	1

Hepatocarcinogenesis	C1512409	PCK1	5105	P35558	phosphoenolpyruvate carboxykinase 1	73	0.608	0.538	8.7E-15	0.01	1	1
Hepatocarcinogenesis	C1512409	NDUFA13	51079	Q9P0J0	NADH:ubiquinone oxidoreductase subunit A13	131	0.556	0.692	3.8E-06	0.01	1	1
Hepatocarcinogenesis	C1512409	MLXIPL	51085	Q9NP71	MLX interacting protein like	165	0.554	0.731	0.04777	0.01	1	1
Hepatocarcinogenesis	C1512409	METTL9	51108	Q9H1A3	methyltransferase like 9	24	0.736	0.5	0.80352	0.01	1	1
Hepatocarcinogenesis	C1512409	AGT	183	P01019	angiotensinogen	765	0.367	0.923	5E-08	0.01	1	1
Hepatocarcinogenesis	C1512409	CHCHD2	51142	Q9Y6H1	coiled-coil-helix-coiled-coil-helix domain containing 2	57	0.638	0.538	1.7E-07	0.01	1	1
Hepatocarcinogenesis	C1512409	DOK1	1796	Q99704	docking protein 1	45	0.65	0.538	0.029	0.01	1	1
Hepatocarcinogenesis	C1512409	HAO2	51179	Q9NYQ3	hydroxyacid oxidase 2	22	0.736	0.308	6E-10	0.01	1	1
Hepatocarcinogenesis	C1512409	NIN	51199	Q8N4C6	ninein	52	0.659	0.731	9.6E-09	0.01	1	1
Hepatocarcinogenesis	C1512409	CPA4	51200	Q9UI42	carboxypeptidase A4	49	0.633	0.5	9.4E-18	0.01	1	1
Hepatocarcinogenesis	C1512409	NUSAP1	51203	Q9BXS6	nucleolar and spindle associated protein 1	55	0.623	0.346	0.04567	0.01	1	1
Hepatocarcinogenesis	C1512409	GOLM1	51280	Q8NBJ4	golgi membrane protein 1	100	0.56	0.692	9.6E-09	0.01	1	1
Hepatocarcinogenesis	C1512409	TNFRSF12A	51330	Q9NP84	TNF receptor superfamily member 12A	173	0.509	0.692	0.018	0.01	1	1
Hepatocarcinogenesis	C1512409	DUSP9	1852	Q99956	dual specificity phosphatase 9	26	0.705	0.308	0.81981	0.01	1	1
Hepatocarcinogenesis	C1512409	E2F2	1870	Q14209	E2F transcription factor 2	75	0.599	0.692	0.25505	0.01	1	1
Hepatocarcinogenesis	C1512409	E2F5	1875	Q15329	E2F transcription factor 5	31	0.686	0.308	0.935	0.01	1	1
Hepatocarcinogenesis	C1512409	ARID2	196528	Q68CP9	AT-rich interaction domain 2	167	0.543	0.808	1	0.01	1	1
Hepatocarcinogenesis	C1512409	NFKBIA	4792	P25963	NFKB inhibitor alpha	226	0.487	0.885	0.99452	0.01	1	1
Hepatocarcinogenesis	C1512409	EEF1A2	1917	Q05639	eukaryotic translation elongation factor 1 alpha 2	166	0.554	0.808	0.99558	0.01	1	1
Hepatocarcinogenesis	C1512409	AHCY	191	P23526	adenosylhomocysteinase	107	0.582	0.692	0.03592	0.01	1	1
Hepatocarcinogenesis	C1512409	NRDC	4898	O43847	nardilysin convertase	42	0.647	0.5	0.04407	0.01	1	1
Hepatocarcinogenesis	C1512409	NTRK1	4914	P04629	neurotrophic receptor tyrosine kinase 1	443	0.422	0.808	2E-06	0.01	1	1
Hepatocarcinogenesis	C1512409	ROR1	4919	Q01973	receptor tyrosine kinase like orphan receptor 1	109	0.552	0.731	0.99645	0.01	1	1
Hepatocarcinogenesis	C1512409	NUP98	4928	P52948	nucleoporin 98 and 96 precursor	129	0.532	0.577	1	0.01	1	1
Hepatocarcinogenesis	C1512409	OGG1	4968	O15527	8-oxoguanine DNA glycosylase	313	0.453	0.808	3.9E-12	0.01	0	1
Hepatocarcinogenesis	C1512409	OPRM1	4988	P35372	opioid receptor mu 1	370	0.457	0.808	7.9E-11	0.01	1	1
Hepatocarcinogenesis	C1512409	P2RY2	5029	P41231	purinergic receptor P2Y2	136	0.543	0.808	0.08425	0.01	1	1
Hepatocarcinogenesis	C1512409	PEBP1	5037	P30086	phosphatidylethanolamine binding protein 1	181	0.5	0.808	0.09167	0.01	1	1
Hepatocarcinogenesis	C1512409	PAEP	5047	P09466	progestagen associated endometrial protein	397	0.43	0.846	3.1E-08	0.01	1	1
Hepatocarcinogenesis	C1512409	DUOX2	50506	Q9NRD8	dual oxidase 2	150	0.533	0.769	4.5E-56	0.01	1	1
Hepatocarcinogenesis	C1512409	LPAR1	1902	Q92633	lysophosphatidic acid receptor 1	145	0.534	0.808	0.18603	0.01	1	1
Hepatocarcinogenesis	C1512409	DELEC1	50514	Q9P2X7	deleted in esophageal cancer 1	92	0.573	0.692	2.7E-05	0.01	1	1
Hepatocarcinogenesis	C1512409	DACT1	51339	Q9NYF0	dishevelled binding antagonist of beta catenin 1	134	0.547	0.692	0.40017	0.01	1	1

Table S3. The differentially expressed gene list in RNA-seq assay

gene_id	GH-1	GH-2	CTRL-1	CTRL-2	GH	CTRL	log2FoldChange	pvalue	padj	gene_name	gene_description
ENSG00000132837	17.1066	39.7117	0.97179	0	28.4092	0.48589	5.856782593	0.00027191	0.00131254	DMGDH	dimethylglycine dehydrogenase [Source:HGNC Symbol;Acc:HGNC:24475]
ENSG00000100292	42306.62	42590.3	842.542	826.168	42448.5	834.355	5.668865955	0	0	HMOX1	heme oxygenase 1 [Source:HGNC Symbol;Acc:HGNC:5013]
ENSG00000105550	79.49535	75.3505	3.88716	0	77.4229	1.94358	5.304797581	1.93E-10	2.37E-09	FGF21	fibroblast growth factor 21 [Source:HGNC Symbol;Acc:HGNC:3678]
ENSG00000130513	5507.317	5222.6	275.988	230.536	5364.96	253.262	4.404044853	0	0	GDF15	growth differentiation factor 15 [Source:HGNC Symbol;Acc:HGNC:30142]
ENSG00000130487	436.7213	459.231	35.9562	16.8202	447.976	26.3882	4.08387569	2.12E-20	5.68E-19	KLHDC7B	kelch domain containing 7B [Source:HGNC Symbol;Acc:HGNC:25145]
ENSG00000173110	197.229	221.978	15.5486	9.89423	209.604	12.7214	4.040120264	5.89E-27	2.10E-25	HSPA6	heat shock protein family A (Hsp70) member 6 [Source:HGNC Symbol;Acc:HGNC:5239]
ENSG00000159339	48.30097	35.6387	3.88716	2.96827	41.9699	3.42771	3.61303467	2.44E-06	1.75E-05	PADI4	peptidyl arginine deiminase 4 [Source:HGNC Symbol;Acc:HGNC:18368]
ENSG00000116183	80.50162	69.241	3.88716	9.89423	74.8713	6.8907	3.445501233	1.02E-09	1.16E-08	PAPPA2	pappalysin 2 [Source:HGNC Symbol;Acc:HGNC:14615]
ENSG00000106823	321.0002	295.292	27.2101	29.6827	308.146	28.4464	3.437730691	3.88E-36	2.04E-34	ECM2	extracellular matrix protein 2 [Source:HGNC Symbol;Acc:HGNC:3154]
ENSG00000007314	45.28216	45.8212	6.80253	1.97885	45.5517	4.39069	3.370364886	2.17E-06	1.57E-05	SCN4A	sodium voltage-gated channel alpha subunit 4 [Source:HGNC Symbol;Acc:HGNC:10591]
ENSG00000139269	266.6616	254.562	26.2383	25.725	260.612	25.9817	3.326286025	2.49E-30	1.02E-28	INHBE	inhibin subunit beta E [Source:HGNC Symbol;Acc:HGNC:24029]
ENSG00000227764	24.15049	31.5657	3.88716	1.97885	27.8581	2.933	3.24475225	0.00022291	0.00109313	LINC01693	long intergenic non-protein coding RNA 1693 [Source:HGNC Symbol;Acc:HGNC:52478]
ENSG00000128165	552.4424	611.968	68.9971	59.3654	582.205	64.1812	3.180634823	7.08E-61	7.06E-59	ADM2	adrenomedullin 2 [Source:HGNC Symbol;Acc:HGNC:28898]
ENSG00000229953	78.48908	89.606	9.7179	10.8837	84.0475	10.3008	3.028807467	4.58E-10	5.43E-09	AL590666.2	novel transcript
ENSG00000074211	225.4045	281.037	34.9844	33.6404	253.221	34.3124	2.883300433	8.05E-25	2.61E-23	PPP2R2C	protein phosphatase 2 regulatory subunit Bgamma [Source:HGNC Symbol;Acc:HGNC:9306]
ENSG00000204388	5560.65	5645.18	815.332	719.311	5602.91	767.321	2.867860423	0	0	HSPA1B	heat shock protein family A (Hsp70) member 1B [Source:HGNC Symbol;Acc:HGNC:5233]
ENSG00000009950	329.0504	320.749	44.7023	44.524	324.9	44.6132	2.864454499	9.89E-33	4.56E-31	MLXIPL	MLX interacting protein like [Source:HGNC Symbol;Acc:HGNC:12744]
ENSG00000107731	1847.512	2015.12	272.101	267.144	1931.31	269.623	2.840449036	1.99E-167	7.34E-165	UNC5B	unc-5 netrin receptor B [Source:HGNC Symbol;Acc:HGNC:12568]
ENSG00000272405	338.1068	374.716	68.0253	35.6192	356.411	51.8223	2.781094126	1.08E-10	1.37E-09	AL365181.3	novel transcript, antisense to BCAN
ENSG00000183054	77.48281	201.613	27.2101	13.8519	139.548	20.531	2.764195712	0.00069299	0.00304259	RGPD6	RANBP2-like and GRIP domain containing 6 [Source:HGNC Symbol;Acc:HGNC:32419]
ENSG00000132692	56.35114	53.9672	9.7179	6.92596	55.1592	8.32193	2.727398704	1.75E-06	1.27E-05	BCAN	brevican [Source:HGNC Symbol;Acc:HGNC:23059]
ENSG00000180316	54.3386	58.0402	6.80253	10.8837	56.1894	8.84309	2.669300541	2.09E-06	1.51E-05	PNPLA1	patatin like phospholipase domain containing 1 [Source:HGNC Symbol;Acc:HGNC:21246]
ENSG00000182459	27.1693	22.4015	2.91537	4.94712	24.7854	3.93124	2.658453688	0.00159489	0.00642175	TEX19	testis expressed 19 [Source:HGNC Symbol;Acc:HGNC:33802]
ENSG00000101255	7312.566	7181.72	1217.65	1092.32	7247.14	1154.99	2.64922777	0	0	TRIB3	tribbles pseudokinase 3 [Source:HGNC Symbol;Acc:HGNC:16228]
ENSG00000151632	4706.326	4684.97	751.194	764.824	4695.65	758.009	2.631100829	0	0	AKR1C2	aldo-keto reductase family 1 member C2 [Source:HGNC Symbol;Acc:HGNC:385]
ENSG00000135842	2427.124	2596.54	437.305	407.642	2511.83	422.474	2.571534629	9.72E-190	4.78E-187	FAM129A	family with sequence similarity 129 member A [Source:HGNC Symbol;Acc:HGNC:16784]
ENSG00000134548	67.42011	53.9672	9.7179	10.8837	60.6937	10.3008	2.559354761	1.47E-06	1.09E-05	SPX	spexin hormone [Source:HGNC Symbol;Acc:HGNC:28139]
ENSG00000133134	50.31352	67.2045	9.7179	10.8837	58.759	10.3008	2.512236714	3.12E-06	2.20E-05	BEX2	brain expressed X-linked 2 [Source:HGNC Symbol;Acc:HGNC:30933]
ENSG00000148680	38.23827	38.6935	9.7179	3.95769	38.4659	6.8378	2.489116789	0.00020394	0.00100781	HTR7	5-hydroxytryptamine receptor 7 [Source:HGNC Symbol;Acc:HGNC:5302]
ENSG00000070669	114.7148	150.701	24.2947	23.7462	132.708	24.0204	2.465649604	8.12E-12	1.15E-10	ASNS	asparagine synthetase (glutamine-hydrolyzing) [Source:HGNC Symbol;Acc:HGNC:753]
ENSG00000272068	46.28843	77.387	10.6897	11.8731	61.8377	11.2814	2.45453991	4.42E-06	3.05E-05	AL365181.2	novel transcript
ENSG00000185633	45.28216	34.6205	7.77432	6.92596	39.9513	7.35014	2.442202367	0.0001441	0.00073508	NDUFA4L2	NDUFA4, mitochondrial complex associated like 2 [Source:HGNC Symbol;Acc:HGNC:29836]
ENSG00000238120	21.13168	25.4562	1.94358	6.92596	23.294	4.43477	2.396568128	0.00502157	0.01746959	LINC01589	long intergenic non-protein coding RNA 1589 [Source:HGNC Symbol;Acc:HGNC:51520]
ENSG00000125538	76.47654	77.387	17.4922	12.8625	76.9318	15.1774	2.340632366	2.86E-07	2.37E-06	IL1B	interleukin 1 beta [Source:HGNC Symbol;Acc:HGNC:5992]
ENSG00000145362	109.6835	117.099	22.3512	22.7567	113.391	22.5539	2.329864756	4.34E-10	5.16E-09	ANK2	ankyrin 2 [Source:HGNC Symbol;Acc:HGNC:493]
ENSG00000186529	54.3386	62.1132	12.6333	10.8837	58.2259	11.7585	2.307364669	9.06E-06	5.92E-05	CYP4F3	cytochrome P450 family 4 subfamily F member 3 [Source:HGNC Symbol;Acc:HGNC:2646]
ENSG00000128965	312.9501	357.406	68.9971	69.2596	335.178	69.1283	2.27749177	2.73E-25	9.13E-24	CHAC1	ChaC glutathione specific gamma-glutamylcyclotransferase 1 [Source:HGNC Symbol;Acc:HGNC:28680]
ENSG00000140297	25.15676	21.3832	4.85895	4.94712	23.27	4.90303	2.246908494	0.00557373	0.01908644	GCNT3	glucosaminyl (N-acetyl) transferase 3, mucin type [Source:HGNC Symbol;Acc:HGNC:4205]
ENSG00000225614	1774.055	1707.6	372.195	364.108	1740.83	368.152	2.24136194	2.63E-119	6.37E-117	ZNF469	zinc finger protein 469 [Source:HGNC Symbol;Acc:HGNC:23216]
ENSG00000204389	4401.426	5100.41	1043.7	968.645	4750.92	1006.17	2.239246149	4.17E-74	5.45E-72	HSPA1A	heat shock protein family A (Hsp70) member 1A [Source:HGNC Symbol;Acc:HGNC:5232]
ENSG00000283378	6.037622	48.876	5.83074	5.93654	27.4568	5.88364	2.221660789	0.01334851	0.0400401	CNTNAP3C	contactin associated protein like 3C [Source:HGNC Symbol;Acc:HGNC:53878]
ENSG00000149150	34.21319	38.6935	7.77432	7.91538	36.4533	7.84485	2.216193718	0.00059725	0.00265769	SLC43A1	solute carrier family 43 member 1 [Source:HGNC Symbol;Acc:HGNC:9225]
ENSG00000171658	358.2322	356.387	75.7996	79.1538	357.31	77.4767	2.205483832	1.65E-26	5.73E-25	NMRAL2P	NmrA like redox sensor 2, pseudogene [Source:HGNC Symbol;Acc:HGNC:52332]
ENSG00000115008	70.43892	77.387	19.4358	12.8625	73.913	16.1491	2.19301234	1.74E-06	1.27E-05	IL1A	interleukin 1 alpha [Source:HGNC Symbol;Acc:HGNC:5991]
ENSG00000049249	239.4923	217.905	58.3074	42.5452	228.699	50.4263	2.180312869	1.80E-16	3.79E-15	TNFRSF9	TNF receptor superfamily member 9 [Source:HGNC Symbol;Acc:HGNC:11924]
ENSG00000254634	32.20065	33.6022	9.7179	4.94712	32.9014	7.33251	2.163698762	0.00155696	0.00628962	SMG1P6	SMG1 pseudogene 6 [Source:HGNC Symbol;Acc:HGNC:49863]
ENSG00000249839	54.3386	67.2045	22.3512	4.94712	60.7715	13.6491	2.151165603	0.00013124	0.00067737	AC011330.1	histidine acid phosphatase domain containing 2A (HISPPD2A) pseudogene
ENSG00000080031	41.25708	39.7117	12.6333	5.93654	40.4844	9.2849	2.122234619	0.00060155	0.00267455	PTPRH	protein tyrosine phosphatase, receptor type H [Source:HGNC Symbol;Acc:HGNC:9672]
ENSG00000161381	59.36995	37.6752	9.7179	12.8625	48.5226	11.2902	2.104748807	0.00020674	0.0010203	PLXDC1	plexin domain containing 1 [Source:HGNC Symbol;Acc:HGNC:20945]
ENSG00000126500	80.50162	75.3505	18.464	17.8096	77.9261	18.1368	2.103122621	1.31E-06	9.84E-06	FLRT1	fibronectin leucine rich transmembrane protein 1 [Source:HGNC Symbol;Acc:HGNC:3760]
ENSG00000268754	48.30097	61.095	11.6615	13.8519	54.698	12.7567	2.100583591	5.92E-05	0.00033058	LINC01081	long intergenic non-protein coding RNA 1081 [Source:HGNC Symbol;Acc:HGNC:49124]
ENSG00000108448	3122.457	3218.69	732.73	748.993	3170.57	740.861	2.097510202	1.04E-189	4.97E-187	TRIM16L	tripartite motif containing 16 like [Source:HGNC Symbol;Acc:HGNC:32670]
ENSG00000187134	6609.183	6419.05	1503.36	1547.46	6514.11	1525.41	2.094469293	0	0	AKR1C1	aldo-keto reductase family 1 member C1 [Source:HGNC Symbol;Acc:HGNC:384]
ENSG00000060566	639.9879	617.059	149.656	147.424	628.524	148.54	2.081098844	1.34E-41	8.15E-40	CREB3L3	cAMP responsive element binding protein 3 like 3 [Source:HGNC Symbol;Acc:HGNC:18855]
ENSG00000167772	1005.264	965.301	290.565	182.054	985.282	236.309	2.05961317	6.99E-11	9.01E-10	ANGPTL4	angiopoietin like 4 [Source:HGNC Symbol;Acc:HGNC:16039]
ENSG00000151012	12906.42	14415.4	3128.19	3452.1	13660.9	3290.14	2.0538574	1.95E-85	3.17E-83	SLC7A11	solute carrier family 7 member 11 [Source:HGNC Symbol;Acc:HGNC:11059]
ENSG00000154263	24.15049	32.584	7.77432	5.93654	28.3672	6.85543	2.04784959	0.00439536	0.01558115	ABCA10	ATP binding cassette subfamily A member 10 [Source:HGNC Symbol;Acc:HGNC:30]
ENSG00000179111	246.5362	202.632	55.392	53.4288	224.584	54.4104	2.04533772	4.09E-15	7.67E-14	HES7	hes family bHLH transcription factor 7 [Source:HGNC Symbol;Acc:HGNC:15977]
ENSG00000163219	45.28216	59.0585	10.6897	14.8413	52.1703	12.7655	2.031753411	0.00014074	0.00071986	ARHGAP25	Rho GTPase activating protein 25 [Source:HGNC Symbol;Acc:HGNC:28951]
ENSG00000138378	27.1693	35.6387	9.7179	5.93654	31.404	7.82722	2.002719778	0.00337895	0.01243497	STAT4	signal transducer and activator of transcription 4 [Source:HGNC Symbol;Acc:HGNC:11365]
ENSG00000103154	34.21319	60.0767	12.6333	10.8837	47.145	11.7585	2.002578815	0.00046973	0.00215065	NECAB2	N-terminal EF-hand calcium binding protein 2 [Source:HGNC Symbol;Acc:HGNC:23746]
ENSG00000196358	18.11287	32.584	6.80253	5.93654	25.3484	6.36953	1.991799441	0.00924411	0.02943954	NTNG2	netrin G2 [Source:HGNC Symbol;Acc:HGNC:14288]
ENSG00000162444	21.13168	21.3832	4.85895	5.93654	21.2575	5.39774	1.978139587	0.01508196	0.0444716	RBP7	retinol binding protein 7 [Source:HGNC Symbol;Acc:HGNC:30316]
ENSG00000107187	22.13795	20.365	4.85895	5.93654	21.2515	5.39774	1.977809512	0.01515046	0.04462899	LHX3	LIM homeobox 3 [Source:HGNC Symbol;Acc:HGNC:6595]
ENSG00000251003	41.25708	42.7665	10.6897	10.8837	42.0118	10.7867	1.961568664	0.00069558	0.00305216	ZFPM2-AS1	ZFPM2 antisense RNA 1 [Source:HGNC Symbol;Acc:HGNC:50698]
ENSG00000081181	72.45146	77.387	24.2947	14.8413	74.9192	19.568	1.935389984	1.22E-05	7.81E-05	ARG2	arginase 2 [Source:HGNC Symbol;Acc:HGNC:664]
ENSG00000244578	26.16303	33.6022	8.74611	6.92596	29.8826	7.83603	1.930219638	0.00496618	0.01730133	LINC01391	long intergenic non-protein coding RNA 1391 [Source:HGNC Symbol;Acc:HGNC:50666]
ENSG00000123342	43.26962	60.0767	12.6333	14.8413	51.6732	13.7373	1.911529487	0.00028797	0.00138053	MMP19	matrix metallopeptidase 19 [Source:HGNC Symbol;Acc:HGNC:7165]

ENSG00000176046	1166.267	1194.41	329.437	299.795	1180.34	314.616	1.907250589	3.47E-65	3.71E-63	NUPR1	nuclear protein 1, transcriptional regulator [Source:HGNC Symbol;Acc:HGNC:29990]
ENSG00000023909	5831.336	5940.47	1602.48	1550.43	5885.9	1576.45	1.90048394	1.99E-282	1.73E-279	GCLM	glutamate-cysteine ligase modifier subunit [Source:HGNC Symbol;Acc:HGNC:4312]
ENSG00000259863	56.35114	60.0767	19.4358	11.8731	58.2139	15.6544	1.893351799	0.00013625	0.00070078	SH3RF3-AS1	SH3RF3 antisense RNA 1 [Source:HGNC Symbol;Acc:HGNC:44168]
ENSG00000175445	384.3953	398.136	109.812	100.921	391.265	105.367	1.892454375	2.06E-23	6.28E-22	LPL	lipoprotein lipase [Source:HGNC Symbol;Acc:HGNC:6677]
ENSG00000249348	19.11914	31.5657	6.80253	6.92596	25.3424	6.86425	1.884047424	0.01182399	0.03611294	UGDH-AS1	UGDH antisense RNA 1 [Source:HGNC Symbol;Acc:HGNC:40601]
ENSG00000171931	36.22573	31.5657	7.77432	10.8837	33.8957	9.32899	1.862394201	0.00354482	0.01295495	FBXW10	F-box and WD repeat domain containing 10 [Source:HGNC Symbol;Acc:HGNC:1211]
ENSG00000223559	79.49535	83.4965	18.464	26.7144	81.4959	22.5892	1.85209883	8.39E-06	5.52E-05	AC073136.1	exportin, tRNA (nuclear export receptor for tRNAs) (XPOT) pseudogene
ENSG00000229512	66.41384	53.9672	11.6615	21.7673	60.1905	16.7144	1.850263004	0.00018285	0.00091215	AC068580.1	novel transcript
ENSG00000198729	23.14422	29.5292	10.6897	3.95769	26.3367	7.32369	1.843714919	0.01329775	0.03992843	PPP1R14C	protein phosphatase 1 regulatory inhibitor subunit 14C [Source:HGNC Symbol;Acc:HGNC:14952]
ENSG00000130433	28.17557	24.438	5.83074	8.90481	26.3068	7.36777	1.837456531	0.01097617	0.03395919	CACNG6	calcium voltage-gated channel auxiliary subunit gamma 6 [Source:HGNC Symbol;Acc:HGNC:13625]
ENSG00000185904	30.18811	22.4015	5.83074	8.90481	26.2948	7.36777	1.836909516	0.01144608	0.03514052	LINC00839	long intergenic non-protein coding RNA 839 [Source:HGNC Symbol;Acc:HGNC:28269]
ENSG00000175197	402.5081	430.72	123.417	111.805	416.614	117.611	1.824354371	2.49E-23	7.55E-22	DDIT3	DNA damage inducible transcript 3 [Source:HGNC Symbol;Acc:HGNC:2726]
ENSG00000113389	111.696	130.336	34.0126	34.6298	121.016	34.3212	1.817952195	7.10E-08	6.39E-07	NPR3	natriuretic peptide receptor 3 [Source:HGNC Symbol;Acc:HGNC:7945]
ENSG00000116717	839.2294	748.414	237.117	214.705	793.822	225.911	1.812880417	7.28E-40	4.28E-38	GADD45A	growth arrest and DNA damage inducible alpha [Source:HGNC Symbol;Acc:HGNC:4095]
ENSG00000197653	26.16303	25.4562	8.74611	5.93654	25.8096	7.34132	1.812712601	0.01238965	0.03759941	DNAH10	dynein axonemal heavy chain 10 [Source:HGNC Symbol;Acc:HGNC:2941]
ENSG00000170801	3990.868	3743.09	1088.4	1149.71	3866.98	1119.06	1.789093896	2.28E-163	7.82E-161	HTRA3	HtrA serine peptidase 3 [Source:HGNC Symbol;Acc:HGNC:30406]
ENSG00000138669	33.20692	40.73	15.5486	5.93654	36.9685	10.7426	1.780436249	0.0047933	0.01680617	PRKG2	protein kinase cGMP-dependent 2 [Source:HGNC Symbol;Acc:HGNC:9416]
ENSG00000125772	1274.944	1324.74	378.998	377.96	1299.84	378.479	1.780016205	6.25E-66	6.74E-64	GPCPD1	glycerophosphocholine phosphodiesterase 1 [Source:HGNC Symbol;Acc:HGNC:26957]
ENSG00000153132	660.1133	679.173	193.386	196.895	669.643	195.141	1.778905253	1.30E-35	6.55E-34	CLGN	calmegin [Source:HGNC Symbol;Acc:HGNC:2060]
ENSG00000174564	1004.258	1036.58	299.311	315.626	1020.42	307.469	1.730769133	2.85E-50	2.17E-48	IL20RB	interleukin 20 receptor subunit beta [Source:HGNC Symbol;Acc:HGNC:6004]
ENSG00000156510	445.7777	440.902	141.881	126.646	443.34	134.264	1.723039605	6.21E-23	1.86E-21	HKDC1	hexokinase domain containing 1 [Source:HGNC Symbol;Acc:HGNC:23302]
ENSG00000006459	422.6335	488.76	138.966	138.519	455.697	138.743	1.715543476	1.52E-22	4.49E-21	KDM7A	lysine demethylase 7A [Source:HGNC Symbol;Acc:HGNC:22224]
ENSG00000111981	299.8685	256.599	82.6021	87.0692	278.234	84.8357	1.713818106	1.34E-14	2.41E-13	ULBP1	UL16 binding protein 1 [Source:HGNC Symbol;Acc:HGNC:14893]
ENSG00000257732	69.43265	56.0037	14.5768	23.7462	62.7182	19.1615	1.712118757	0.0002789	0.00134185	AC089983.1	novel transcript, antisense to TXNRD1
ENSG00000180747	284.7745	453.121	120.502	104.879	368.948	112.69	1.710534827	8.52E-14	1.44E-12	SMG1P3	SMG1 pseudogene 3 [Source:HGNC Symbol;Acc:HGNC:49860]
ENSG00000078018	1129.035	1135.35	336.239	356.192	1132.19	346.216	1.709526982	1.38E-54	1.22E-52	MAP2	microtubule associated protein 2 [Source:HGNC Symbol;Acc:HGNC:6839]
ENSG00000171119	50.31352	58.0402	19.4358	13.8519	54.1769	16.6439	1.701647745	0.00061681	0.00273582	NRTN	neurturin [Source:HGNC Symbol;Acc:HGNC:8007]
ENSG00000154493	72.45146	61.095	21.3794	19.7885	66.7732	20.5839	1.697676266	0.00013489	0.00069462	C10orf90	chromosome 10 open reading frame 90 [Source:HGNC Symbol;Acc:HGNC:26563]
ENSG00000178752	169.0534	151.719	58.3074	40.5663	160.386	49.4369	1.697032529	1.06E-08	1.07E-07	ERFE	erythroferrone [Source:HGNC Symbol;Acc:HGNC:26727]
ENSG00000113739	4582.555	4747.08	1544.17	1344.63	4664.82	1444.4	1.691063196	3.23E-149	1.06E-146	STC2	stanniocalcin 2 [Source:HGNC Symbol;Acc:HGNC:11374]
ENSG00000249628	56.35114	70.2592	20.4076	18.799	63.3052	19.6033	1.690820123	0.0002217	0.0010882	LINC00942	long intergenic non-protein coding RNA 942 [Source:HGNC Symbol;Acc:HGNC:48636]
ENSG00000120129	3709.112	3873.42	1240.98	1129.92	3791.27	1185.45	1.677008741	4.02E-142	1.26E-139	DUSP1	dual specificity phosphatase 1 [Source:HGNC Symbol;Acc:HGNC:3064]
ENSG00000211584	1168.28	1158.77	388.716	343.33	1163.52	366.023	1.668182063	1.03E-51	8.11E-50	SLC48A1	solute carrier family 48 member 1 [Source:HGNC Symbol;Acc:HGNC:26035]
ENSG00000204936	61.38249	75.3505	23.323	19.7885	68.3665	21.5557	1.664605175	0.00015332	0.00077853	CD177	CD177 molecule [Source:HGNC Symbol;Acc:HGNC:30072]
ENSG00000163053	65.40757	80.4417	24.2947	21.7673	72.9247	23.031	1.662359606	9.30E-05	0.00049455	SLC16A14	solute carrier family 16 member 14 [Source:HGNC Symbol;Acc:HGNC:26417]
ENSG00000132429	63.39503	85.533	24.2947	22.7567	74.464	23.5257	1.661890036	9.22E-05	0.00049135	POPDC3	popeye domain containing 3 [Source:HGNC Symbol;Acc:HGNC:17649]
ENSG00000168672	282.762	289.183	92.32	90.0375	285.972	91.1788	1.649018758	9.89E-15	1.79E-13	FAM84B	family with sequence similarity 84 member B [Source:HGNC Symbol;Acc:HGNC:24166]
ENSG00000116690	162.0095	187.358	61.2228	50.4606	174.684	55.8417	1.644704657	3.15E-09	3.40E-08	PRG4	proteoglycan 4 [Source:HGNC Symbol;Acc:HGNC:9364]
ENSG00000100302	129.8089	144.591	52.4766	35.6192	137.2	44.0479	1.638065879	2.53E-07	2.11E-06	RASD2	RASD family member 2 [Source:HGNC Symbol;Acc:HGNC:18229]
ENSG00000274615	51.31979	42.7665	19.4358	10.8837	47.0431	15.1597	1.632405062	0.00235374	0.0090904	AC233968.1	aminopeptidase puromycin sensitive pseudogene [Source:NCBI gene;Acc:440434]
ENSG00000087842	1768.07	1688.26	542.259	578.813	1728.14	560.536	1.624539819	1.22E-72	1.53E-70	PIR	pirin [Source:HGNC Symbol;Acc:HGNC:30048]
ENSG00000116761	524.2668	512.18	186.584	151.382	518.223	168.983	1.616187782	6.41E-23	1.91E-21	CTH	cystathionine gamma-lyase [Source:HGNC Symbol;Acc:HGNC:2501]
ENSG00000196139	4958.9	4666.64	1583.05	1568.24	4812.77	1575.64	1.610946912	8.78E-172	3.41E-169	AKR1C3	aldo-keto reductase family 1 member C3 [Source:HGNC Symbol;Acc:HGNC:386]
ENSG00000285106	31.19438	28.511	8.74611	10.8837	29.8527	9.81488	1.605486725	0.01387128	0.04138689	AC016831.6	novel transcript
ENSG00000167103	90.56433	105.898	42.7588	21.7673	98.2312	32.263	1.604635425	3.59E-05	0.00020832	PIP5KL1	phosphatidylinositol-4-phosphate 5-kinase like 1 [Source:HGNC Symbol;Acc:HGNC:28711]
ENSG00000174951	111.696	101.825	34.9844	35.6192	106.76	35.3018	1.596688528	3.86E-06	2.68E-05	FUT1	fucosyltransferase 1 (H blood group) [Source:HGNC Symbol;Acc:HGNC:4012]
ENSG00000274213	26.16303	35.6387	9.7179	10.8837	30.9009	10.3008	1.584941595	0.0138336	0.04130215	AC015912.3	novel transcript
ENSG00000128203	38.23827	40.73	13.6051	12.8625	39.4841	13.2338	1.576840778	0.00518713	0.01796087	ASPHD2	aspartate beta-hydroxylase domain containing 2 [Source:HGNC Symbol;Acc:HGNC:30437]
ENSG00000081041	225.4045	297.329	97.179	78.1644	261.367	87.6717	1.57518881	3.54E-11	4.68E-10	CXCL2	C-X-C motif chemokine ligand 2 [Source:HGNC Symbol;Acc:HGNC:4603]
ENSG00000074935	715.4582	764.706	249.75	252.303	740.082	251.026	1.559818061	8.09E-32	3.55E-30	TUBE1	tubulin epsilon 1 [Source:HGNC Symbol;Acc:HGNC:20775]
ENSG00000262001	338.1068	360.46	115.643	122.688	349.284	119.166	1.551532191	5.14E-16	1.04E-14	DLGAP1-AS2	DLGAP1 antisense RNA 2 [Source:HGNC Symbol;Acc:HGNC:28146]
ENSG00000185561	588.6681	620.114	218.653	193.927	604.391	206.29	1.550461937	1.08E-25	3.69E-24	TLCD2	TLC domain containing 2 [Source:HGNC Symbol;Acc:HGNC:33522]
ENSG00000221926	1264.882	1208.66	445.08	403.685	1236.77	424.382	1.542937106	8.93E-49	6.52E-47	TRIM16	tripartite motif containing 16 [Source:HGNC Symbol;Acc:HGNC:17241]
ENSG00000139289	2287.252	2407.14	852.26	758.888	2347.2	805.574	1.542557914	4.22E-81	6.23E-79	PHLDA1	pleckstrin homology like domain family A member 1 [Source:HGNC Symbol;Acc:HGNC:8933]
ENSG00000279491	57.35741	79.4235	27.2101	19.7885	68.3904	23.4993	1.540125046	0.00048513	0.00221017	AP003733.4	uncharacterized LOC399900 [Source:NCBI gene;Acc:399900]
ENSG00000135116	87.54552	83.4965	28.1819	30.6721	85.521	29.427	1.539403223	5.66E-05	0.00031715	HRK	harakiri, BCL2 interacting protein [Source:HGNC Symbol;Acc:HGNC:5185]
ENSG00000198223	520.2417	467.377	182.696	158.308	493.809	170.502	1.533891454	1.83E-20	4.96E-19	CSF2RA	colony stimulating factor 2 receptor alpha subunit [Source:HGNC Symbol;Acc:HGNC:2435]
ENSG00000133794	1085.766	1106.84	395.518	362.129	1096.3	378.824	1.532808311	3.55E-44	2.35E-42	ARNTL	aryl hydrocarbon receptor nuclear translocator like [Source:HGNC Symbol;Acc:HGNC:701]
ENSG00000116852	414.5834	426.647	146.74	146.435	420.615	146.587	1.520709071	1.39E-18	3.37E-17	KIF21B	kinesin family member 21B [Source:HGNC Symbol;Acc:HGNC:29442]
ENSG00000147872	2859.82	2771.68	1087.43	875.639	2815.75	981.536	1.520287779	2.24E-23	6.83E-22	PLIN2	perilipin 2 [Source:HGNC Symbol;Acc:HGNC:248]
ENSG00000101695	68.42638	80.4417	21.3794	30.6721	74.4341	26.0257	1.51680473	0.00027648	0.00133196	RNF125	ring finger protein 125 [Source:HGNC Symbol;Acc:HGNC:21150]
ENSG00000120694	11408.09	11792.4	4047.5	4071.48	11600.2	4059.49	1.514774124	0	0	HSPH1	heat shock protein family H (Hsp110) member 1 [Source:HGNC Symbol;Acc:HGNC:16969]
ENSG00000197822	47.2947	52.949	25.2665	9.89423	50.1218	17.5804	1.509344473	0.00463831	0.01632477	OCLN	occludin [Source:HGNC Symbol;Acc:HGNC:8104]
ENSG00000115963	3116.419	3107.7	1027.18	1159.6	3112.06	1093.39	1.509317878	1.26E-99	2.47E-97	RND3	Rho family GTPase 3 [Source:HGNC Symbol;Acc:HGNC:671]
ENSG00000250033	163.0158	199.577	60.251	67.2808	181.296	63.7659	1.507609898	1.99E-08	1.93E-07	SLC7A11-AS1	SLC7A11 antisense RNA 1 [Source:HGNC Symbol;Acc:HGNC:44064]
ENSG00000153790	61.38249	72.2957	26.2383	20.7779	66.8391	23.5081	1.506793936	0.00052808	0.00237883	C7orf31	chromosome 7 open reading frame 31 [Source:HGNC Symbol;Acc:HGNC:21722]
ENSG00000170458	417.6022	330.931	134.107	129.614	374.267	131.861	1.505141635	8.04E-15	1.47E-13	CD14	CD14 molecule [Source:HGNC Symbol;Acc:HGNC:1628]
ENSG00000073792	786.9034	782.016	253.637	301.774	784.46	277.706	1.49856542	7.85E-30	3.15E-28	IGF2BP2	insulin like growth factor 2 mRNA binding protein 2 [Source:HGNC Symbol;Acc:HGNC:28867]
ENSG00000004799	117.7336	108.953	32.0691	48.4817	113.343	40.2754	1.493797273	1.23E-05	7.82E-05	PDK4	pyruvate dehydrogenase kinase 4 [Source:HGNC Symbol;Acc:HGNC:8812]
ENSG00000174945	833.1918	909.297	293.481	325.52	871.244	309.5	1.493320235	5.29E-33	2.49E-31	AMZ1	archaelysin family metallopeptidase 1 [Source:HGNC Symbol;Acc:HGNC:22231]
ENSG00000165626	141.8841	127.281	52.4766	43.5346	134.583	48.0056	1.486828227	1.22E-06	9.17E-06	BEND7	BEN domain containing 7 [Source:HGNC Symbol;Acc:HGNC:23514]

ENSG00000173868	42.26335	42.7665	14.5768	15.8308	42.5149	15.2038	1.483742889	0.00566427	0.01933634	PHOSPHO1	phosphoethanolamine/phosphocholine phosphatase [Source:HGNC Symbol;Acc:HGNC:16815]
ENSG00000223396	110.6897	105.898	39.8434	37.5981	108.294	38.7207	1.483658535	1.08E-05	6.94E-05	RPS10P7	ribosomal protein S10 pseudogene 7 [Source:HGNC Symbol;Acc:HGNC:36423]
ENSG00000221883	35.21946	35.6387	10.6897	14.8413	35.4291	12.7655	1.473518653	0.0125186	0.03794395	ARIH2OS	ariadne RBR E3 ubiquitin protein ligase 2 opposite strand [Source:HGNC Symbol;Acc:HGNC:34425]
ENSG00000154319	451.8154	462.285	169.091	162.265	457.05	165.678	1.463841882	6.28E-19	1.57E-17	FAM167A	family with sequence similarity 167 member A [Source:HGNC Symbol;Acc:HGNC:15549]
ENSG00000150527	1528.525	1650.58	560.723	598.601	1589.55	579.662	1.455433554	5.91E-55	5.29E-53	MIA2	MIA SH3 domain ER export factor 2 [Source:HGNC Symbol;Acc:HGNC:18432]
ENSG00000111087	37.232	42.7665	15.5486	13.8519	39.9992	14.7003	1.443709254	0.00881956	0.02830736	GLI1	GLI family zinc finger 1 [Source:HGNC Symbol;Acc:HGNC:4317]
ENSG00000189410	56.35114	44.803	20.4076	16.8202	50.5771	18.6139	1.441803824	0.0035578	0.01298973	SH2D5	SH2 domain containing 5 [Source:HGNC Symbol;Acc:HGNC:28819]
ENSG00000181773	154.9656	172.084	57.3356	63.3231	163.525	60.3293	1.438743201	1.66E-07	1.42E-06	GPR3	G protein-coupled receptor 3 [Source:HGNC Symbol;Acc:HGNC:4484]
ENSG00000204390	76.47654	77.387	27.2101	29.6827	76.9318	28.4464	1.435547508	0.00029615	0.00141656	HSPA1L	heat shock protein family A (Hsp70) member 1 like [Source:HGNC Symbol;Acc:HGNC:5234]
ENSG00000149489	109.6835	112.007	42.7588	39.5769	110.845	41.1678	1.428742158	1.55E-05	9.65E-05	ROM1	retinal outer segment membrane protein 1 [Source:HGNC Symbol;Acc:HGNC:10254]
ENSG00000182118	43.26962	40.73	12.6333	18.799	41.9998	15.7162	1.419178721	0.00872739	0.02805718	FAM89A	family with sequence similarity 89 member A [Source:HGNC Symbol;Acc:HGNC:25057]
ENSG00000232187	268.6742	247.435	98.1508	94.9846	258.054	96.5677	1.418049329	7.69E-11	9.86E-10	FTH1P7	ferritin heavy chain 1 pseudogene 7 [Source:HGNC Symbol;Acc:HGNC:3994]
ENSG00000100889	1441.985	1411.29	567.525	502.627	1426.64	535.076	1.414541041	1.50E-47	1.08E-45	PCK2	phosphoenolpyruvate carboxykinase 2, mitochondrial [Source:HGNC Symbol;Acc:HGNC:8725]
ENSG00000214185	69.43265	66.1862	24.2947	26.7144	67.8094	25.5046	1.41101443	0.00080665	0.00349383	XPOTP1	exportin for tRNA pseudogene 1 [Source:HGNC Symbol;Acc:HGNC:16586]
ENSG00000156453	638.9816	573.275	255.581	200.853	606.128	228.217	1.40877104	7.54E-20	1.95E-18	PCDH1	protocadherin 1 [Source:HGNC Symbol;Acc:HGNC:8655]
ENSG00000138166	266.6616	293.256	110.784	101.911	279.959	106.347	1.396142602	2.87E-11	3.84E-10	DUSP5	dual specificity phosphatase 5 [Source:HGNC Symbol;Acc:HGNC:3071]
ENSG00000153982	144.9029	161.902	63.1663	53.4288	153.402	58.2976	1.395302008	8.58E-07	6.62E-06	GDPD1	glycerophosphodiester phosphodiesterase domain containing 1 [Source:HGNC Symbol;Acc:HGNC:20883]
ENSG00000176678	125.7838	137.464	48.5895	52.4394	131.624	50.5145	1.381767392	4.91E-06	3.36E-05	FOXL1	forkhead box L1 [Source:HGNC Symbol;Acc:HGNC:3817]
ENSG00000224259	316.9751	323.803	117.587	128.625	320.389	123.106	1.380133961	1.31E-12	2.00E-11	LINC01133	long intergenic non-protein coding RNA 1133 [Source:HGNC Symbol;Acc:HGNC:49447]
ENSG00000169429	397.4768	428.683	167.148	151.382	413.08	159.265	1.374687669	2.07E-15	4.02E-14	CXCL8	C-X-C motif chemokine ligand 8 [Source:HGNC Symbol;Acc:HGNC:6025]
ENSG00000178381	415.5896	389.99	136.051	175.128	402.79	155.589	1.372908641	4.81E-14	8.37E-13	ZFAND2A	zinc finger AN1-type containing 2A [Source:HGNC Symbol;Acc:HGNC:28073]
ENSG00000100439	906.6495	903.188	363.449	339.372	904.919	351.411	1.364465196	1.78E-31	7.64E-30	ABHD4	abhydrolase domain containing 4 [Source:HGNC Symbol;Acc:HGNC:20154]
ENSG00000227467	105.6584	114.044	39.8434	45.5135	109.851	42.6784	1.364239015	3.64E-05	0.00021095	LINC01537	long intergenic non-protein coding RNA 1537 [Source:HGNC Symbol;Acc:HGNC:51301]
ENSG00000110628	273.7055	240.307	93.2918	106.858	257.006	100.075	1.361160555	6.43E-10	7.52E-09	SLC22A18	solute carrier family 22 member 18 [Source:HGNC Symbol;Acc:HGNC:10964]
ENSG00000091129	122.765	109.971	52.4766	38.5875	116.368	45.5321	1.353092614	3.51E-05	0.00020384	NRCAM	neuronal cell adhesion molecule [Source:HGNC Symbol;Acc:HGNC:7994]
ENSG00000229644	363.2636	357.406	139.938	142.477	360.335	141.207	1.351581305	1.09E-13	1.83E-12	NAMPTP1	nicotinamide phosphoribosyltransferase pseudogene 1 [Source:HGNC Symbol;Acc:HGNC:17633]
ENSG00000182165	45.28216	34.6205	17.4922	13.8519	39.9513	15.6721	1.349699332	0.01406282	0.04184263	TP53TG1	TP53 target 1 [Source:HGNC Symbol;Acc:HGNC:17026]
ENSG00000219507	334.0817	276.964	129.248	111.805	305.523	120.526	1.341743042	7.77E-11	9.95E-10	FTH1P8	ferritin heavy chain 1 pseudogene 8 [Source:HGNC Symbol;Acc:HGNC:3995]
ENSG00000130766	1223.625	1231.06	482.008	486.796	1227.34	484.402	1.341282421	3.20E-41	1.94E-39	SESN2	sestrin 2 [Source:HGNC Symbol;Acc:HGNC:20746]
ENSG00000092068	172.0722	178.194	61.2228	77.175	175.133	69.1989	1.340160866	4.07E-07	3.28E-06	SLC7A8	solute carrier family 7 member 8 [Source:HGNC Symbol;Acc:HGNC:11066]
ENSG00000184489	97.60822	93.679	28.1819	47.4923	95.6436	37.8371	1.339075441	0.00028053	0.00134838	PTP4A3	protein tyrosine phosphatase type IVA, member 3 [Source:HGNC Symbol;Acc:HGNC:9636]
ENSG00000285517	389.4266	416.464	157.43	161.276	402.945	159.353	1.338360498	9.71E-15	1.76E-13	LINC00941	long intergenic non-protein coding RNA 941 [Source:NCBI gene;Acc:100287314]
ENSG00000109971	78524.3	79562	31452	31234.1	79043.1	31343	1.334486691	0	0	HSPA8	heat shock protein family A (Hsp70) member 8 [Source:HGNC Symbol;Acc:HGNC:5241]
ENSG00000124882	2533.789	2683.09	956.241	1124.97	2608.44	1040.61	1.326017424	1.52E-60	1.50E-58	EREG	epiregulin [Source:HGNC Symbol;Acc:HGNC:3443]
ENSG00000235027	56.35114	46.8395	20.4076	20.7779	51.5953	20.5927	1.325309267	0.00560546	0.01917991	AC068580.3	novel transcript
ENSG00000243244	70.43892	69.241	25.2665	30.6721	69.84	27.9693	1.320702145	0.00131301	0.00541227	STON1	stonin 1 [Source:HGNC Symbol;Acc:HGNC:17003]
ENSG00000138413	5893.725	6077.93	2409.07	2408.26	5985.83	2408.66	1.313297232	1.22E-158	4.10E-156	IDH1	isocitrate dehydrogenase (NADP(+)) 1, cytosolic [Source:HGNC Symbol;Acc:HGNC:5382]
ENSG00000099957	75.47027	56.0037	27.2101	25.725	65.737	26.4676	1.312594735	0.00222368	0.00864691	P2RX6	purinergic receptor P2X 6 [Source:HGNC Symbol;Acc:HGNC:8538]
ENSG00000039068	154.9656	163.938	64.1381	64.3125	159.452	64.2253	1.311864583	1.33E-06	9.92E-06	CDH1	cadherin 1 [Source:HGNC Symbol;Acc:HGNC:1748]
ENSG00000224536	39.24454	52.949	20.4076	16.8202	46.0968	18.6139	1.30756294	0.01042105	0.03251431	AC096677.1	novel transcript
ENSG00000127948	5685.427	5480.22	2241.92	2272.7	5582.82	2257.31	1.306442956	3.67E-146	1.18E-143	POR	cytochrome p450 oxidoreductase [Source:HGNC Symbol;Acc:HGNC:9208]
ENSG00000142611	76.47654	78.4052	36.928	25.725	77.4409	31.3265	1.304829012	0.00096927	0.00412437	PRDM16	PR/SET domain 16 [Source:HGNC Symbol;Acc:HGNC:14000]
ENSG00000205464	71.44519	73.314	43.7305	14.8413	72.3796	29.2859	1.303458874	0.00485501	0.01697814	ATP6AP1L	ATPase H+ transporting accessory protein 1 like [Source:HGNC Symbol;Acc:HGNC:28091]
ENSG00000248323	1861.6	2004.93	880.442	690.617	1933.27	785.529	1.299165341	2.20E-13	3.58E-12	LUCAT1	lung cancer associated transcript 1 [Source:HGNC Symbol;Acc:HGNC:48498]
ENSG00000137875	55.34487	65.168	23.323	25.725	60.2564	24.524	1.297020286	0.00329681	0.01217824	BCL2L10	BCL2 like 10 [Source:HGNC Symbol;Acc:HGNC:993]
ENSG00000147509	94.58941	91.6425	34.9844	41.5558	93.1159	38.2701	1.283259001	0.0002946	0.0014105	RGS20	regulator of G protein signaling 20 [Source:HGNC Symbol;Acc:HGNC:14600]
ENSG00000182870	724.5146	650.662	302.227	263.187	687.588	282.707	1.282015402	1.42E-20	3.87E-19	GALNT9	polypeptide N-acetylgalactosaminyltransferase 9 [Source:HGNC Symbol;Acc:HGNC:4131]
ENSG00000172985	381.3764	393.044	170.063	148.413	387.21	159.238	1.28158004	3.81E-13	6.02E-12	SH3RF3	SH3 domain containing ring finger 3 [Source:HGNC Symbol;Acc:HGNC:24699]
ENSG00000106105	10292.13	10012.4	4160.23	4192.19	10152.3	4176.21	1.281570725	7.87E-229	4.84E-226	GARS	glycyl-tRNA synthetase [Source:HGNC Symbol;Acc:HGNC:4162]
ENSG00000224411	527.2856	536.618	233.23	204.811	531.952	219.02	1.279910784	2.74E-17	6.11E-16	HSP90AA2P	heat shock protein 90 alpha family class A member 2, pseudogene [Source:HGNC Symbol;Acc:HGNC:5256]
ENSG00000210082	110838.7	119015	46899.5	47939.5	114927	47419.5	1.277162721	2.29E-126	6.03E-124	MT-RNR2	mitochondrially encoded 16S RNA [Source:HGNC Symbol;Acc:HGNC:7471]
ENSG00000121281	377.3514	357.406	137.022	167.213	367.379	152.117	1.272584064	4.67E-12	6.72E-11	ADCY7	adenylate cyclase 7 [Source:HGNC Symbol;Acc:HGNC:238]
ENSG00000125266	294.8372	346.205	131.192	134.562	320.521	132.877	1.270258597	8.47E-11	1.08E-09	EFNB2	ephrin B2 [Source:HGNC Symbol;Acc:HGNC:3227]
ENSG00000285184	36.22573	48.876	16.5204	18.799	42.5509	17.6597	1.268778211	0.01597711	0.04663598	AC244033.2	novel transcript
ENSG00000229261	45.28216	39.7117	16.5204	18.799	42.497	17.6597	1.267328329	0.01507295	0.0444628	AL596223.1	uncharacterized LOC101928994 [Source:NCBI gene;Acc:101928994]
ENSG00000146592	100.627	114.044	42.7588	46.5029	107.336	44.6308	1.266109535	0.00012398	0.00064283	CREB5	cAMP responsive element binding protein 5 [Source:HGNC Symbol;Acc:HGNC:16844]
ENSG00000074527	2471.4	2520.17	1016.49	1060.66	2495.78	1038.58	1.264965399	2.92E-70	3.51E-68	NTN4	netrin 4 [Source:HGNC Symbol;Acc:HGNC:13658]
ENSG00000130517	670.176	611.968	300.283	233.504	641.072	266.893	1.263776296	2.00E-17	4.50E-16	PGPEP1	pyroglutamyl-peptidase I [Source:HGNC Symbol;Acc:HGNC:13568]
ENSG00000087074	1015.327	1039.63	437.305	418.526	1027.48	427.916	1.26358745	1.43E-31	6.20E-30	PPP1R15A	protein phosphatase 1 regulatory subunit 15A [Source:HGNC Symbol;Acc:HGNC:14375]
ENSG00000178093	78.48908	81.46	31.0973	35.6192	79.9745	33.3583	1.261795695	0.00090408	0.0038749	TSSK6	testis specific serine kinase 6 [Source:HGNC Symbol;Acc:HGNC:30410]
ENSG00000151929	3305.598	3221.74	1344.96	1377.28	3263.67	1361.12	1.261776631	3.04E-89	5.22E-87	BAG3	BCL2 associated athanogene 3 [Source:HGNC Symbol;Acc:HGNC:939]
ENSG00000185760	115.7211	124.226	45.6741	54.4183	119.974	50.0462	1.261747391	5.45E-05	0.00030668	KCNQ5	potassium voltage-gated channel subfamily Q member 5 [Source:HGNC Symbol;Acc:HGNC:6299]
ENSG00000115425	191.1914	182.267	82.6021	73.2173	186.729	77.9097	1.260820538	4.72E-07	3.77E-06	PECR	peroxisomal trans-2-enoyl-CoA reductase [Source:HGNC Symbol;Acc:HGNC:18281]
ENSG00000087086	63421.19	63579.5	26912.7	26163.3	63500.4	26538	1.258680648	0	0	FTL	ferritin light chain [Source:HGNC Symbol;Acc:HGNC:3999]
ENSG00000166123	1564.75	1540.61	646.24	652.03	1552.68	649.135	1.258205078	3.41E-46	2.42E-44	GPT2	glutamic--pyruvic transaminase 2 [Source:HGNC Symbol;Acc:HGNC:18062]
ENSG00000167995	69.43265	94.6972	35.9562	32.651	82.0649	34.3036	1.257921749	0.0010617	0.00447765	BEST1	bestrophin 1 [Source:HGNC Symbol;Acc:HGNC:12703]
ENSG00000182885	224.3983	217.905	82.6021	102.9	221.152	92.7511	1.254140248	7.85E-08	7.02E-07	ADGRG3	adhesion G protein-coupled receptor G3 [Source:HGNC Symbol;Acc:HGNC:13728]
ENSG00000258102	120.7524	115.062	58.3074	40.5663	117.907	49.4369	1.253203152	0.00010116	0.00053389	MAP1LC3B2	microtubule associated protein 1 light chain 3 beta 2 [Source:HGNC Symbol;Acc:HGNC:34390]
ENSG00000111252	1267.901	1315.58	554.892	529.341	1291.74	542.117	1.252499897	5.45E-38	2.99E-36	SH2B3	SH2B adaptor protein 3 [Source:HGNC Symbol;Acc:HGNC:29605]
ENSG00000158691	191.1914	183.285	75.7996	82.1221	187.238	78.9609	1.245896177	5.36E-07	4.26E-06	ZSCAN12	zinc finger and SCAN domain containing 12 [Source:HGNC Symbol;Acc:HGNC:13172]
ENSG00000253981	65.40757	60.0767	29.1537	23.7462	62.7422	26.4499	1.245749863	0.00373143	0.01352313	ALG1L13P	asparagine-linked glycosylation 1-like 13, pseudogene [Source:HGNC Symbol;Acc:HGNC:44382]

ENSG00000183889	84.52671	40.73	31.0973	21.7673	62.6283	26.4323	1.244292577	0.00904831	0.02892834	PKD1P1	polycystin 1, transient receptor potential channel interacting pseudogene 1 [Source:NCBI gene;Acc:339044]
ENSG00000234975	291.8184	260.672	118.558	114.773	276.245	116.666	1.243588203	1.77E-09	1.98E-08	FTH1P2	ferritin heavy chain 1 pseudogene 2 [Source:HGNC Symbol;Acc:HGNC:3989]
ENSG00000261780	261.6303	230.124	105.925	101.911	245.877	103.918	1.242513773	1.47E-08	1.46E-07	LINC02582	long intergenic non-protein coding RNA 2582 [Source:HGNC Symbol;Acc:HGNC:53792]
ENSG00000105357	196.2227	180.23	76.7714	83.1115	188.226	79.9415	1.235718408	6.51E-07	5.11E-06	MYH14	myosin heavy chain 14 [Source:HGNC Symbol;Acc:HGNC:23212]
ENSG00000161653	141.8841	104.88	53.4484	51.45	123.382	52.4492	1.234296823	9.17E-05	0.00048851	NAGS	N-acetylglutamate synthase [Source:HGNC Symbol;Acc:HGNC:17996]
ENSG00000239467	79.49535	63.1315	32.0691	28.6933	71.3134	30.3812	1.230930188	0.00238092	0.00918818	AC007405.3	glutamate rich 2 [Source:NCBI gene;Acc:285141]
ENSG00000210135	875.4552	978.538	413.011	377.96	926.997	395.485	1.228662306	2.83E-25	9.43E-24	MT-TN	mitochondrially encoded tRNA asparagine [Source:HGNC Symbol;Acc:HGNC:7493]
ENSG00000140403	1894.807	1899.04	809.501	810.338	1896.92	809.919	1.227810465	1.68E-53	1.45E-51	DNAJA4	DnaJ heat shock protein family (Hsp40) member A4 [Source:HGNC Symbol;Acc:HGNC:14885]
ENSG00000175592	1696.572	1680.11	767.714	677.755	1688.34	722.734	1.223814961	4.57E-43	2.92E-41	FOSL1	FOS like 1, AP-1 transcription factor subunit [Source:HGNC Symbol;Acc:HGNC:13718]
ENSG00000183604	109.6835	109.971	41.787	52.4394	109.827	47.1132	1.221568272	0.00017526	0.00087846	SMG1P5	SMG1 pseudogene 5 [Source:HGNC Symbol;Acc:HGNC:49862]
ENSG00000023330	3058.055	2885.72	1251.67	1300.1	2971.89	1275.88	1.220004935	1.99E-73	2.53E-71	ALAS1	5'-aminolevulinate synthase 1 [Source:HGNC Symbol;Acc:HGNC:396]
ENSG00000164620	111.696	102.843	42.7588	49.4712	107.27	46.115	1.21835644	0.00020085	0.00099356	RELL2	RELT like 2 [Source:HGNC Symbol;Acc:HGNC:26902]
ENSG00000162772	644.013	684.264	287.65	283.964	664.138	285.807	1.216359571	6.26E-20	1.63E-18	ATF3	activating transcription factor 3 [Source:HGNC Symbol;Acc:HGNC:785]
ENSG00000185710	96.60195	109.971	60.251	28.6933	103.286	44.4721	1.214189641	0.00108745	0.00457446	SMG1P4	SMG1 pseudogene 4 [Source:HGNC Symbol;Acc:HGNC:49861]
ENSG00000168702	277.7306	299.365	115.643	133.572	288.548	124.608	1.211691346	2.18E-09	2.39E-08	LRP1B	LDL receptor related protein 1B [Source:HGNC Symbol;Acc:HGNC:6693]
ENSG00000154277	207.2917	214.851	87.4611	94.9846	211.071	91.2228	1.210428387	2.06E-07	1.74E-06	UCHL1	ubiquitin C-terminal hydrolase L1 [Source:HGNC Symbol;Acc:HGNC:12513]
ENSG00000187735	5080.659	5079.03	2196.24	2196.52	5079.84	2196.38	1.209655694	8.46E-125	2.15E-122	TCEA1	transcription elongation factor A1 [Source:HGNC Symbol;Acc:HGNC:11612]
ENSG00000165030	553.4487	623.169	241.004	268.134	588.309	254.569	1.208659073	1.09E-16	2.31E-15	NFIL3	nuclear factor, interleukin 3 regulated [Source:HGNC Symbol;Acc:HGNC:7787]
ENSG00000159753	60.37622	52.949	22.3512	26.7144	56.6626	24.5328	1.208227448	0.00718	0.02378456	CARMIL2	capping protein regulator and myosin 1 linker 2 [Source:HGNC Symbol;Acc:HGNC:27089]
ENSG00000213190	555.4612	615.023	275.017	231.525	585.242	253.271	1.207902906	3.70E-16	7.58E-15	MLLT11	MLLT11, transcription factor 7 cofactor [Source:HGNC Symbol;Acc:HGNC:16997]
ENSG00000103723	70.43892	63.1315	27.2101	30.6721	66.7852	28.9411	1.20679269	0.00348179	0.01277206	AP3B2	adaptor related protein complex 3 subunit beta 2 [Source:HGNC Symbol;Acc:HGNC:567]
ENSG00000140044	653.0694	657.789	266.27	301.774	655.429	284.022	1.206716472	5.60E-19	1.41E-17	JDP2	Jun dimerization protein 2 [Source:HGNC Symbol;Acc:HGNC:17546]
ENSG00000140941	4305.831	4366.25	1796.84	1964.99	4336.04	1880.92	1.205101942	8.95E-94	1.61E-91	MAP1LC3B	microtubule associated protein 1 light chain 3 beta [Source:HGNC Symbol;Acc:HGNC:13352]
ENSG00000010810	718.477	712.775	315.832	305.732	715.626	310.782	1.203233993	3.14E-21	8.77E-20	FYN	FYN proto-oncogene, Src family tyrosine kinase [Source:HGNC Symbol;Acc:HGNC:4037]
ENSG00000171791	370.3075	400.172	155.486	180.075	385.24	167.781	1.199450169	1.08E-11	1.50E-10	BCL2	BCL2, apoptosis regulator [Source:HGNC Symbol;Acc:HGNC:990]
ENSG00000101493	136.8528	135.427	66.0817	52.4394	136.14	59.2606	1.199415236	4.18E-05	0.00023944	ZNF516	zinc finger protein 516 [Source:HGNC Symbol;Acc:HGNC:28990]
ENSG00000102452	67.42011	53.9672	26.2383	26.7144	60.6937	26.4764	1.197078092	0.00591569	0.02007988	NALCN	sodium leak channel, non-selective [Source:HGNC Symbol;Acc:HGNC:19082]
ENSG00000073150	524.2668	479.596	227.399	212.726	501.931	220.062	1.189499243	9.50E-15	1.72E-13	PANX2	pannexin 2 [Source:HGNC Symbol;Acc:HGNC:8600]
ENSG00000121743	69.43265	82.4782	38.8716	27.7038	75.9554	33.2877	1.189244636	0.00259786	0.00994476	GJA3	gap junction protein alpha 3 [Source:HGNC Symbol;Acc:HGNC:4277]
ENSG00000134668	262.6365	237.252	120.502	98.9423	249.944	109.722	1.187391187	5.98E-08	5.43E-07	SPOCD1	SPOC domain containing 1 [Source:HGNC Symbol;Acc:HGNC:26338]
ENSG00000171160	155.9719	151.719	73.856	61.3442	153.846	67.6001	1.185973587	1.49E-05	9.33E-05	MORN4	MORN repeat containing 4 [Source:HGNC Symbol;Acc:HGNC:24001]
ENSG00000139974	371.3137	420.537	171.035	178.096	395.925	174.566	1.181446559	8.65E-12	1.22E-10	SLC38A6	solute carrier family 38 member 6 [Source:HGNC Symbol;Acc:HGNC:19863]
ENSG00000167676	1535.568	1377.69	644.297	641.146	1456.63	642.721	1.180441421	1.24E-35	6.30E-34	PLIN4	perilipin 4 [Source:HGNC Symbol;Acc:HGNC:29393]
ENSG00000080824	103864.2	109718	46537.1	47802	106791	47169.5	1.178870577	0	0	HSP90AA1	heat shock protein 90 alpha family class A member 1 [Source:HGNC Symbol;Acc:HGNC:5253]
ENSG00000261824	319.994	329.913	147.712	139.509	324.953	143.61	1.177910661	3.12E-10	3.76E-09	LINC00662	long intergenic non-protein coding RNA 662 [Source:HGNC Symbol;Acc:HGNC:27122]
ENSG00000104413	150.9405	147.646	59.2792	73.2173	149.293	66.2482	1.172705407	2.50E-05	0.00014964	ESRP1	epithelial splicing regulatory protein 1 [Source:HGNC Symbol;Acc:HGNC:25966]
ENSG00000244405	2229.895	2122.03	934.862	997.338	2175.96	966.1	1.171584598	2.44E-52	2.00E-50	ETV5	ETS variant 5 [Source:HGNC Symbol;Acc:HGNC:3494]
ENSG00000135069	8766.627	8633.74	3937.69	3814.23	8700.18	3875.96	1.166444314	8.22E-173	3.37E-170	PSAT1	phosphoserine aminotransferase 1 [Source:HGNC Symbol;Acc:HGNC:19129]
ENSG00000163235	472.947	419.519	182.696	215.694	446.233	199.195	1.164068701	3.07E-12	4.49E-11	TGFA	transforming growth factor alpha [Source:HGNC Symbol;Acc:HGNC:11765]
ENSG00000198431	25198.01	25841.1	11283.5	11499.1	25519.6	11391.3	1.163689634	0	0	TXNRD1	thioredoxin reductase 1 [Source:HGNC Symbol;Acc:HGNC:12437]
ENSG00000273812	52.32606	52.949	27.2101	19.7885	52.6375	23.4993	1.162722137	0.01245298	0.03776833	BX640514.2	novel transcript
ENSG00000165521	106.6647	88.5877	44.7023	42.5452	97.6262	43.6238	1.162201107	0.0006821	0.00299923	EML5	echinoderm microtubule associated protein like 5 [Source:HGNC Symbol;Acc:HGNC:18197]
ENSG00000204934	90.56433	86.5512	47.6177	31.6615	88.5578	39.6396	1.158807183	0.00156857	0.00633131	ATP6V0E2-AS1	ATP6V0E2 antisense RNA 1 [Source:HGNC Symbol;Acc:HGNC:44180]
ENSG00000152409	1282.995	1356.31	568.497	616.411	1319.65	592.454	1.155513121	8.20E-33	3.79E-31	JMY	junction mediating and regulatory protein, p53 cofactor [Source:HGNC Symbol;Acc:HGNC:28916]
ENSG00000168389	319.994	283.073	121.474	149.403	301.534	135.438	1.155235247	9.68E-09	9.83E-08	MFSD2A	major facilitator superfamily domain containing 2A [Source:HGNC Symbol;Acc:HGNC:25897]
ENSG00000134830	123.7712	125.245	48.5895	63.3231	124.508	55.9563	1.154461554	0.00015408	0.0007821	C5AR2	complement component 5a receptor 2 [Source:HGNC Symbol;Acc:HGNC:4527]
ENSG00000105835	3922.442	4002.74	1778.38	1785.91	3962.59	1782.14	1.152824715	3.80E-92	6.75E-90	NAMPT	nicotinamide phosphoribosyltransferase [Source:HGNC Symbol;Acc:HGNC:30092]
ENSG00000114841	63.39503	67.2045	33.0409	25.725	65.2998	29.3829	1.151463116	0.00569206	0.01942669	DNAH1	dynein axonemal heavy chain 1 [Source:HGNC Symbol;Acc:HGNC:2940]
ENSG00000183010	1135.073	1128.22	499.5	520.437	1131.65	509.968	1.150043597	7.45E-30	3.00E-28	PYCR1	pyrroline-5-carboxylate reductase 1 [Source:HGNC Symbol;Acc:HGNC:9721]
ENSG00000033327	172.0722	202.632	95.2354	74.2067	187.352	84.7211	1.14427871	6.83E-06	4.56E-05	GAB2	GRB2 associated binding protein 2 [Source:HGNC Symbol;Acc:HGNC:14458]
ENSG00000109654	665.1447	686.3	299.311	313.647	675.723	306.479	1.14072222	1.87E-18	4.49E-17	TRIM2	tripartite motif containing 2 [Source:HGNC Symbol;Acc:HGNC:15974]
ENSG00000091490	4468.846	4556.67	1960.1	2152	4512.76	2056.05	1.134288889	2.41E-85	3.87E-83	SEL1L3	SEL1L family member 3 [Source:HGNC Symbol;Acc:HGNC:29108]
ENSG00000088340	78.48908	78.4052	42.7588	28.6933	78.4472	35.726	1.133858346	0.00330346	0.01219364	FER1L4	fer-1 like family member 4, pseudogene [Source:HGNC Symbol;Acc:HGNC:15801]
ENSG00000110619	2859.82	2898.96	1343.99	1282.29	2879.39	1313.14	1.13263258	2.98E-66	3.26E-64	CARS	cysteinyl-tRNA synthetase [Source:HGNC Symbol;Acc:HGNC:1493]
ENSG00000130940	110.6897	121.172	65.1099	40.5663	115.931	52.8381	1.132551837	0.00055297	0.00248326	CASZ1	castor zinc finger 1 [Source:HGNC Symbol;Acc:HGNC:26002]
ENSG00000197971	282.762	275.946	136.051	118.731	279.354	127.391	1.132542066	2.12E-08	2.05E-07	MBP	myelin basic protein [Source:HGNC Symbol;Acc:HGNC:6925]
ENSG00000128342	361.251	356.387	165.204	162.265	358.819	163.735	1.131865903	1.43E-10	1.79E-09	LIF	LIF, interleukin 6 family cytokine [Source:HGNC Symbol;Acc:HGNC:6596]
ENSG00000026559	402.5081	333.986	159.374	177.107	368.247	168.24	1.130527186	6.75E-10	7.86E-09	KCNG1	potassium voltage-gated channel modifier subfamily G member 1 [Source:HGNC Symbol;Acc:HGNC:6248]
ENSG00000119771	272.6993	323.803	127.304	145.445	298.251	136.375	1.129100176	2.02E-08	1.96E-07	KLHL29	kelch like family member 29 [Source:HGNC Symbol;Acc:HGNC:29404]
ENSG00000198380	3717.162	3929.43	1734.64	1762.16	3823.29	1748.4	1.128768328	9.08E-81	1.33E-78	GFPT1	glutamine--fructose-6-phosphate transaminase 1 [Source:HGNC Symbol;Acc:HGNC:4241]
ENSG00000274265	62.38876	46.8395	28.1819	21.7673	54.6141	24.9746	1.128474234	0.0141096	0.04195644	AC245297.3	novel transcript
ENSG00000161011	16375.04	16716.6	7566.36	7597.78	16545.8	7582.07	1.125798797	4.91E-259	3.81E-256	SQSTM1	sequestosome 1 [Source:HGNC Symbol;Acc:HGNC:11280]
ENSG00000100814	1065.64	1020.29	467.431	489.764	1042.96	478.598	1.123945084	2.28E-26	7.91E-25	CCNB1IP1	cyclin B1 interacting protein 1 [Source:HGNC Symbol;Acc:HGNC:19437]
ENSG00000233024	77.48281	59.0585	32.0691	30.6721	68.2706	31.3706	1.121987279	0.00603188	0.02043529	AC126755.2	nuclear pore complex interacting protein family member A5 pseudogene [Source:NCBI gene;Acc:105376752]
ENSG00000263155	56.35114	47.8577	28.1819	19.7885	52.1044	23.9852	1.11860577	0.01687335	0.04880805	MYZAP	myocardial zonula adherens protein [Source:HGNC Symbol;Acc:HGNC:43444]
ENSG00000108846	4788.84	5005.72	2356.59	2170.79	4897.28	2263.69	1.11313351	4.62E-87	7.58E-85	ABCC3	ATP binding cassette subfamily C member 3 [Source:HGNC Symbol;Acc:HGNC:54]
ENSG00000175832	1264.882	1225.97	590.848	564.961	1245.43	577.904	1.107664812	2.24E-30	9.22E-29	ETV4	ETS variant 4 [Source:HGNC Symbol;Acc:HGNC:3493]
ENSG00000188372	116.7274	108.953	59.2792	45.5135	112.84	52.3963	1.106206067	0.00050855	0.00230406	ZP3	zona pellucida glycoprotein 3 [Source:HGNC Symbol;Acc:HGNC:13189]
ENSG00000141622	858.3486	913.37	432.446	390.822	885.859	411.634	1.105444249	2.34E-21	6.61E-20	RNF165	ring finger protein 165 [Source:HGNC Symbol;Acc:HGNC:31696]
ENSG00000151773	76.47654	79.4235	33.0409	39.5769	77.95	36.3089	1.102610381	0.00345992	0.01270444	CCDC122	coiled-coil domain containing 122 [Source:HGNC Symbol;Acc:HGNC:26478]
ENSG00000132002	4556.392	4541.39	2195.27	2045.14	4548.89	2120.21	1.101180503	5.74E-88	9.52E-86	DNAJB1	DnaJ heat shock protein family (Hsp40) member B1 [Source:HGNC Symbol;Acc:HGNC:5270]

ENSG00000260916	635.9628	638.443	282.791	313.647	637.203	298.219	1.095608485	3.60E-16	7.39E-15	CCPG1	cell cycle progression 1 [Source:HGNC Symbol;Acc:HGNC:24227]
ENSG00000065833	1675.44	1616.98	741.476	800.443	1646.21	770.959	1.094612715	5.16E-37	2.77E-35	ME1	malic enzyme 1 [Source:HGNC Symbol;Acc:HGNC:6983]
ENSG00000185022	420.621	440.902	217.681	186.012	430.762	201.846	1.09324717	3.27E-11	4.34E-10	MAFF	MAF bZIP transcription factor F [Source:HGNC Symbol;Acc:HGNC:6780]
ENSG00000140961	325.0253	353.333	165.204	153.361	339.179	159.282	1.09021719	2.35E-09	2.57E-08	OSGIN1	oxidative stress induced growth inhibitor 1 [Source:HGNC Symbol;Acc:HGNC:30093]
ENSG00000187840	1735.816	1684.19	818.247	798.464	1710	808.356	1.080906734	4.39E-39	2.48E-37	EIF4EBP1	eukaryotic translation initiation factor 4E binding protein 1 [Source:HGNC Symbol;Acc:HGNC:3288]
ENSG00000186665	322.0065	319.73	158.402	145.445	320.868	151.923	1.07844813	6.72E-09	6.94E-08	C17orf58	chromosome 17 open reading frame 58 [Source:HGNC Symbol;Acc:HGNC:27568]
ENSG00000110172	3869.109	4074.02	1829.88	1957.08	3971.56	1893.48	1.068753821	1.73E-71	2.11E-69	CHORDC1	cysteine and histidine rich domain containing 1 [Source:HGNC Symbol;Acc:HGNC:14525]
ENSG00000250305	189.1788	221.978	99.1226	96.9635	205.579	98.043	1.068013641	5.26E-06	3.58E-05	TRMT9B	tRNA methyltransferase 9B (putative) [Source:HGNC Symbol;Acc:HGNC:26725]
ENSG00000169594	534.3295	526.435	234.201	272.091	530.382	253.146	1.067399403	6.06E-13	9.47E-12	BNC1	basonuclin 1 [Source:HGNC Symbol;Acc:HGNC:1081]
ENSG00000165983	717.4707	694.446	339.155	336.404	705.959	337.779	1.063513383	2.50E-17	5.60E-16	PTER	phosphotriesterase related [Source:HGNC Symbol;Acc:HGNC:9590]
ENSG00000106733	69.43265	106.916	35.9562	48.4817	88.1744	42.219	1.062738504	0.00469409	0.01650396	NMRK1	nicotinamide riboside kinase 1 [Source:HGNC Symbol;Acc:HGNC:26057]
ENSG00000198363	17161.94	17544.4	8235.92	8391.3	17353.2	8313.61	1.061669504	1.42E-233	9.13E-231	ASPH	aspartate beta-hydroxylase [Source:HGNC Symbol;Acc:HGNC:757]
ENSG00000187678	229.4296	213.832	114.671	97.9529	221.631	106.312	1.059564861	2.56E-06	1.83E-05	SPRY4	sprouty RTK signaling antagonist 4 [Source:HGNC Symbol;Acc:HGNC:15533]
ENSG00000197405	53.33233	61.095	26.2383	28.6933	57.2137	27.4658	1.058804411	0.01525165	0.04482867	C5AR1	complement C5a receptor 1 [Source:HGNC Symbol;Acc:HGNC:1338]
ENSG00000136244	515.2104	520.326	278.904	218.663	517.768	248.783	1.056922823	1.31E-11	1.81E-10	IL6	interleukin 6 [Source:HGNC Symbol;Acc:HGNC:6018]
ENSG00000187260	79.49535	61.095	35.9562	31.6615	70.2952	33.8089	1.05597007	0.00821976	0.02676635	WDR86	WD repeat domain 86 [Source:HGNC Symbol;Acc:HGNC:28020]
ENSG00000233621	162.0095	135.427	66.0817	77.175	148.718	71.6284	1.054477731	0.00013793	0.00070816	LINC01137	long intergenic non-protein coding RNA 1137 [Source:HGNC Symbol;Acc:HGNC:49453]
ENSG00000107738	386.4078	445.993	200.189	200.853	416.201	200.521	1.053411881	2.74E-10	3.33E-09	VSIR	V-set immunoregulatory receptor [Source:HGNC Symbol;Acc:HGNC:30085]
ENSG00000204767	78.48908	88.5877	32.0691	48.4817	83.5384	40.2754	1.053319812	0.00474889	0.01667024	INSYN2B	inhibitory synaptic factor family member 2B [Source:HGNC Symbol;Acc:HGNC:37271]
ENSG00000153879	1803.236	1787.03	864.893	868.713	1795.13	866.803	1.05033127	1.64E-39	9.51E-38	CEBPG	CCAAT enhancer binding protein gamma [Source:HGNC Symbol;Acc:HGNC:1837]
ENSG00000144815	2142.349	2079.27	1016.49	1023.06	2110.81	1019.78	1.049579386	4.00E-45	2.76E-43	NXPE3	neurexophilin and PC-esterase domain family member 3 [Source:HGNC Symbol;Acc:HGNC:28238]
ENSG00000141458	3495.783	3601.55	1723.96	1704.78	3548.67	1714.37	1.049555519	9.00E-71	1.09E-68	NPC1	NPC intracellular cholesterol transporter 1 [Source:HGNC Symbol;Acc:HGNC:7897]
ENSG00000166173	1318.214	1320.67	648.184	627.294	1319.44	637.739	1.048815809	1.07E-29	4.29E-28	LARP6	La ribonucleoprotein domain family member 6 [Source:HGNC Symbol;Acc:HGNC:24012]
ENSG00000090861	14422.87	14137.4	7100.87	6718.18	14280.1	6909.53	1.047286387	3.84E-176	1.67E-173	AARS	alanyl-tRNA synthetase [Source:HGNC Symbol;Acc:HGNC:20]
ENSG00000025039	71.44519	64.1497	37.8998	27.7038	67.7975	32.8018	1.046855044	0.00990029	0.03115288	RRAGD	Ras related GTP binding D [Source:HGNC Symbol;Acc:HGNC:19903]
ENSG00000031698	4654	4458.92	2191.39	2226.2	4556.46	2208.79	1.044715851	4.77E-84	7.41E-82	SARS	seryl-tRNA synthetase [Source:HGNC Symbol;Acc:HGNC:10537]
ENSG00000197063	2411.024	2403.07	1197.25	1137.84	2407.05	1167.54	1.043687268	1.73E-49	1.30E-47	MAFG	MAF bZIP transcription factor G [Source:HGNC Symbol;Acc:HGNC:6781]
ENSG00000198142	2806.488	2868.41	1349.82	1408.94	2837.45	1379.38	1.040647783	9.07E-57	8.42E-55	SOWAHC	sosondowah ankyrin repeat domain family member C [Source:HGNC Symbol;Acc:HGNC:26149]
ENSG00000001084	1929.02	2176	1009.69	987.444	2052.51	998.567	1.039332849	1.02E-36	5.45E-35	GCLC	glutamate-cysteine ligase catalytic subunit [Source:HGNC Symbol;Acc:HGNC:4311]
ENSG00000165929	154.9656	154.774	88.4329	62.3337	154.87	75.3833	1.038006314	0.00016693	0.00084155	TC2N	tandem C2 domains, nuclear [Source:HGNC Symbol;Acc:HGNC:19859]
ENSG00000112276	89.55806	75.3505	41.787	38.5875	82.4543	40.1872	1.036832535	0.00444552	0.01573251	BVES	blood vessel epicardial substance [Source:HGNC Symbol;Acc:HGNC:1152]
ENSG00000164220	70.43892	64.1497	27.2101	38.5875	67.2943	32.8988	1.033298333	0.01127481	0.03470855	F2RL2	coagulation factor II thrombin receptor like 2 [Source:HGNC Symbol;Acc:HGNC:3539]
ENSG00000101574	552.4424	588.548	276.96	280.996	570.495	278.978	1.032038839	1.39E-13	2.30E-12	METTL4	methyltransferase like 4 [Source:HGNC Symbol;Acc:HGNC:24726]
ENSG00000183688	203.2666	199.577	87.4611	109.826	201.422	98.6435	1.030435204	1.39E-05	8.78E-05	RFLNB	refilin B [Source:HGNC Symbol;Acc:HGNC:28705]
ENSG00000109321	209.3042	247.435	93.2918	130.604	228.369	111.948	1.029072194	1.22E-05	7.81E-05	AREG	amphiregulin [Source:HGNC Symbol;Acc:HGNC:651]
ENSG00000095752	499.1101	537.636	245.863	262.197	518.373	254.03	1.029067859	2.70E-12	3.97E-11	IL11	interleukin 11 [Source:HGNC Symbol;Acc:HGNC:5966]
ENSG00000104231	1232.681	1245.32	612.228	604.538	1239	608.383	1.026088831	3.23E-27	1.17E-25	ZFAND1	zinc finger AN1-type containing 1 [Source:HGNC Symbol;Acc:HGNC:25858]
ENSG00000065325	1110.922	1045.74	545.174	514.5	1078.33	529.837	1.025109163	4.70E-23	1.42E-21	GLP2R	glucagon like peptide 2 receptor [Source:HGNC Symbol;Acc:HGNC:4325]
ENSG00000080298	132.8277	147.646	82.6021	55.4077	140.237	69.0049	1.022196944	0.00048243	0.00219923	RFX3	regulatory factor X3 [Source:HGNC Symbol;Acc:HGNC:9984]
ENSG00000095637	144.9029	153.756	68.9971	78.1644	149.329	73.5808	1.021324063	0.00015511	0.00078652	SORBS1	sorbin and SH3 domain containing 1 [Source:HGNC Symbol;Acc:HGNC:14565]
ENSG00000073331	295.8435	340.095	151.599	162.265	317.969	156.932	1.01877201	6.80E-08	6.14E-07	ALPK1	alpha kinase 1 [Source:HGNC Symbol;Acc:HGNC:20917]
ENSG00000123689	369.3012	359.442	189.499	170.181	364.372	179.84	1.018483034	4.92E-09	5.18E-08	G0S2	G0/G1 switch 2 [Source:HGNC Symbol;Acc:HGNC:30229]
ENSG00000167785	459.8655	432.756	229.342	211.737	446.311	220.539	1.016889502	1.14E-10	1.45E-09	ZNF558	zinc finger protein 558 [Source:HGNC Symbol;Acc:HGNC:26422]
ENSG00000136010	81.50789	65.168	35.9562	36.6087	73.3379	36.2824	1.015529646	0.00856474	0.02760956	ALDH1L2	aldehyde dehydrogenase 1 family member L2 [Source:HGNC Symbol;Acc:HGNC:26777]
ENSG00000146733	1162.242	1179.13	550.033	608.495	1170.69	579.264	1.015262392	4.13E-24	1.31E-22	PSPH	phosphoserine phosphatase [Source:HGNC Symbol;Acc:HGNC:9577]
ENSG00000236432	83.52043	100.807	43.7305	47.4923	92.1636	45.6114	1.014815867	0.00324592	0.01202635	AC097662.1	uncharacterized LOC654841 [Source:NCBI gene;Acc:654841]
ENSG00000167996	101487.4	108095	53768.2	49981.7	104791	51874.9	1.014405617	1.95E-48	1.41E-46	FTH1	ferritin heavy chain 1 [Source:HGNC Symbol;Acc:HGNC:3976]
ENSG00000152137	1611.039	1647.53	782.291	833.094	1629.28	807.693	1.012473104	5.58E-33	2.60E-31	HSPB8	heat shock protein family B (small) member 8 [Source:HGNC Symbol;Acc:HGNC:30171]
ENSG00000163393	461.8781	471.45	220.596	242.409	466.664	231.502	1.011546215	4.94E-11	6.43E-10	SLC22A15	solute carrier family 22 member 15 [Source:HGNC Symbol;Acc:HGNC:20301]
ENSG00000184156	123.7712	123.208	65.1099	57.3865	123.49	61.2482	1.011369507	0.00062066	0.00275122	KCNQ3	potassium voltage-gated channel subfamily Q member 3 [Source:HGNC Symbol;Acc:HGNC:6297]
ENSG00000184575	12531.08	12488.8	6097.01	6317.47	12510	6207.24	1.011110334	2.40E-172	9.56E-170	XPOT	exportin for tRNA [Source:HGNC Symbol;Acc:HGNC:12826]
ENSG00000160191	275.7181	324.822	143.825	154.35	300.27	149.087	1.010111127	2.28E-07	1.91E-06	PDE9A	phosphodiesterase 9A [Source:HGNC Symbol;Acc:HGNC:8795]
ENSG00000128591	230.4359	227.07	124.389	102.9	228.753	113.645	1.00885949	4.92E-06	3.37E-05	FLNC	filamin C [Source:HGNC Symbol;Acc:HGNC:3756]
ENSG00000166986	8289.655	8439.25	4165.09	4154.59	8364.45	4159.84	1.007726678	1.34E-134	3.67E-132	MARS	methionyl-tRNA synthetase [Source:HGNC Symbol;Acc:HGNC:6898]
ENSG00000090013	1312.176	1396.02	664.704	684.681	1354.1	674.693	1.005040495	1.76E-27	6.47E-26	BLVRB	biliverdin reductase B [Source:HGNC Symbol;Acc:HGNC:1063]
ENSG00000163814	4513.122	4389.67	2245.81	2192.56	4451.4	2219.18	1.004204184	6.71E-79	9.35E-77	CDCP1	CUB domain containing protein 1 [Source:HGNC Symbol;Acc:HGNC:24357]
ENSG00000124588	1511.418	1586.43	800.755	745.036	1548.93	772.895	1.002735124	2.96E-30	1.21E-28	NQO2	N-ribosyldihydronicotinamide:quinone reductase 2 [Source:HGNC Symbol;Acc:HGNC:7856]
ENSG00000128408	90.56433	109.971	202.132	198.874	100.268	200.503	-1.000075863	2.15E-05	0.00013048	RIBC2	RIB43A domain with coiled-coils 2 [Source:HGNC Symbol;Acc:HGNC:13241]
ENSG00000204335	72.45146	98.7702	177.838	165.234	85.6108	171.536	-1.003170286	0.00011743	0.00061206	SP5	Sp5 transcription factor [Source:HGNC Symbol;Acc:HGNC:14529]
ENSG00000115596	39.24454	46.8395	91.3482	82.1221	43.042	86.7352	-1.011281709	0.00435192	0.01546782	WNT6	Wnt family member 6 [Source:HGNC Symbol;Acc:HGNC:12785]
ENSG00000184545	128.8026	88.5877	210.878	227.567	108.695	219.223	-1.011527598	2.22E-05	0.00013419	DUSP8	dual specificity phosphatase 8 [Source:HGNC Symbol;Acc:HGNC:3074]
ENSG00000166897	737.5961	782.016	1515.02	1555.37	759.806	1535.2	-1.014770937	2.02E-31	8.66E-30	ELFN2	extracellular leucine rich repeat and fibronectin type III domain containing 2 [Source:HGNC Symbol;Acc:HGNC:29396]
ENSG00000143507	111.696	143.573	247.806	269.123	127.635	258.465	-1.018198578	1.85E-06	1.34E-05	DUSP10	dual specificity phosphatase 10 [Source:HGNC Symbol;Acc:HGNC:3065]
ENSG00000164465	785.8971	820.709	1622.89	1634.53	803.303	1628.71	-1.019765041	6.02E-34	2.95E-32	DCBLD1	discoidin, CUB and LCCL domain containing 1 [Source:HGNC Symbol;Acc:HGNC:21479]
ENSG00000126464	842.2482	760.633	1590.82	1660.25	801.44	1625.54	-1.020061736	2.32E-31	9.91E-30	PRR12	proline rich 12 [Source:HGNC Symbol;Acc:HGNC:29217]
ENSG00000260597	102.6396	94.6972	186.584	213.715	98.6684	200.15	-1.020119154	1.52E-05	9.51E-05	AC012531.1	novel transcript
ENSG00000213071	30.18811	32.584	58.3074	69.2596	31.3861	63.7835	-1.022943456	0.01326506	0.03984647	LPAL2	lipoprotein(a) like 2, pseudogene [Source:HGNC Symbol;Acc:HGNC:21210]
ENSG00000260097	39.24454	33.6022	69.9689	78.1644	36.4234	74.0666	-1.023573205	0.00759605	0.02501314	SPDYE6	speedy/RINGO cell cycle regulator family member E6 [Source:HGNC Symbol;Acc:HGNC:35465]
ENSG00000237499	36.22573	43.7847	86.4893	76.1856	40.0052	81.3374	-1.024186442	0.00531778	0.01833634	AL357060.1	uncharacterized LOC100130476 [Source:NCBI gene;Acc:100130476]
ENSG00000146376	216.3481	249.471	471.318	476.902	232.91	474.11	-1.025644068	3.79E-11	5.00E-10	ARHGAP18	Rho GTPase activating protein 18 [Source:HGNC Symbol;Acc:HGNC:21035]
ENSG00000169851	1296.076	1258.56	2622.86	2583.38	1277.32	2603.12	-1.027104346	2.86E-52	2.33E-50	PCDH7	protocadherin 7 [Source:HGNC Symbol;Acc:HGNC:8659]

ENSG00000076248	1342.365	1331.87	2688.94	2762.47	1337.12	2725.71	-1.027459339	1.73E-54	1.52E-52	UNG	uracil DNA glycosylase [Source:HGNC Symbol;Acc:HGNC:12572]
ENSG00000244586	62.38876	52.949	128.276	106.858	57.6689	117.567	-1.02761416	0.00088433	0.0038024	WNT5A-AS1	WNT5A antisense RNA 1 [Source:HGNC Symbol;Acc:HGNC:40616]
ENSG00000180447	151.9468	126.263	298.339	270.113	139.105	284.226	-1.030724282	3.23E-07	2.65E-06	GAS1	growth arrest specific 1 [Source:HGNC Symbol;Acc:HGNC:4165]
ENSG00000253669	92.57687	103.861	221.568	180.075	98.2192	200.822	-1.032264156	1.77E-05	0.00010936	GASAL1	growth arrest associated lncRNA 1 [Source:HGNC Symbol;Acc:HGNC:53461]
ENSG00000104894	50.31352	47.8577	111.756	89.0481	49.0856	100.402	-1.032638147	0.00197375	0.00776091	CD37	CD37 molecule [Source:HGNC Symbol;Acc:HGNC:1666]
ENSG00000104856	103.6458	103.861	206.991	217.673	103.754	212.332	-1.033095549	4.96E-06	3.39E-05	RELB	RELB proto-oncogene, NF-kB subunit [Source:HGNC Symbol;Acc:HGNC:9956]
ENSG00000138496	560.4926	602.804	1199.19	1182.36	581.648	1190.77	-1.033805218	8.81E-26	3.02E-24	PARP9	poly(ADP-ribose) polymerase family member 9 [Source:HGNC Symbol;Acc:HGNC:24118]
ENSG00000182179	73.45773	67.2045	150.627	137.53	70.3311	144.079	-1.034608665	0.00017495	0.00087719	UBA7	ubiquitin like modifier activating enzyme 7 [Source:HGNC Symbol;Acc:HGNC:12471]
ENSG00000165434	237.4798	260.672	497.556	527.363	249.076	512.459	-1.040915154	2.56E-12	3.77E-11	PGM2L1	phosphoglucomutase 2 like 1 [Source:HGNC Symbol;Acc:HGNC:20898]
ENSG00000261505	104.6521	75.3505	193.386	177.107	90.0013	185.246	-1.041076946	4.25E-05	0.00024301	AL031714.1	novel transcript, antisense to UBE2I
ENSG00000084636	174.0848	172.084	376.083	336.404	173.084	356.243	-1.041514974	4.64E-09	4.89E-08	COL16A1	collagen type XVI alpha 1 chain [Source:HGNC Symbol;Acc:HGNC:2193]
ENSG00000241852	73.45773	91.6425	175.894	164.244	82.5501	170.069	-1.043195471	5.30E-05	0.00029831	C8orf58	chromosome 8 open reading frame 58 [Source:HGNC Symbol;Acc:HGNC:32233]
ENSG00000127124	167.0409	177.175	353.731	357.182	172.108	355.457	-1.046435021	2.67E-09	2.90E-08	HIVEP3	human immunodeficiency virus type I enhancer binding protein 3 [Source:HGNC Symbol;Acc:HGNC:13561]
ENSG00000198840	1319.22	1307.43	2695.74	2733.78	1313.33	2714.76	-1.047569118	1.36E-56	1.25E-54	MT-ND3	mitochondrially encoded NADH:ubiquinone oxidoreductase core subunit 3 [Source:HGNC Symbol;Acc:HGNC:7458]
ENSG00000273760	316.9751	384.898	689.971	763.835	350.937	726.903	-1.05069122	5.19E-15	9.66E-14	AC245041.1	novel transcript
ENSG00000203667	28.17557	35.6387	68.0253	64.3125	31.9072	66.1689	-1.052707088	0.0098813	0.03112617	COX20	cytochrome c oxidase assembly factor COX20 [Source:HGNC Symbol;Acc:HGNC:26970]
ENSG00000257167	95.59568	119.135	205.048	240.43	107.365	222.739	-1.052940108	5.15E-06	3.51E-05	TMPO-AS1	TMPO antisense RNA 1 [Source:HGNC Symbol;Acc:HGNC:44158]
ENSG00000256667	62.38876	50.9125	126.333	108.837	56.6506	117.585	-1.053426759	0.00067828	0.00298512	KLRA1P	killer cell lectin like receptor A1, pseudogene [Source:HGNC Symbol;Acc:HGNC:6372]
ENSG00000247199	138.8653	140.518	294.452	285.943	139.692	290.198	-1.054846372	5.62E-08	5.11E-07	AC091948.1	uncharacterized LOC102546294 [Source:NCBI gene;Acc:102546294]
ENSG00000182108	50.31352	52.949	121.474	93.0058	51.6313	107.24	-1.054940703	0.001244	0.0051653	DEXI	Dexi homolog [Source:HGNC Symbol;Acc:HGNC:13267]
ENSG00000225630	992.1825	983.629	1980.51	2134.19	987.906	2057.35	-1.05824036	6.25E-42	3.86E-40	MTND2P28	MT-ND2 pseudogene 28 [Source:HGNC Symbol;Acc:HGNC:42129]
ENSG00000250133	58.36368	48.876	108.84	114.773	53.6198	111.807	-1.059827277	0.00075723	0.00330111	HOXC-AS2	HOXC cluster antisense RNA 2 [Source:HGNC Symbol;Acc:HGNC:43750]
ENSG00000175305	217.3544	206.705	450.91	435.346	212.03	443.128	-1.063429333	1.88E-11	2.57E-10	CCNE2	cyclin E2 [Source:HGNC Symbol;Acc:HGNC:1590]
ENSG00000121716	23.14422	48.876	92.32	58.376	36.0101	75.348	-1.066502998	0.01393002	0.04150605	PILRB	paired immunoglobin-like type 2 receptor beta [Source:HGNC Symbol;Acc:HGNC:18297]
ENSG00000198848	31.19438	31.5657	59.2792	72.2279	31.3801	65.7535	-1.066974699	0.00928758	0.02952067	CES1	carboxylesterase 1 [Source:HGNC Symbol;Acc:HGNC:1863]
ENSG00000183421	313.9563	251.508	611.256	573.865	282.732	592.561	-1.06730177	3.75E-13	5.93E-12	RIPK4	receptor interacting serine/threonine kinase 4 [Source:HGNC Symbol;Acc:HGNC:496]
ENSG00000240184	37.232	34.6205	72.8842	78.1644	35.9262	75.5243	-1.071695216	0.00485481	0.01697814	PCDHGC3	protocadherin gamma subfamily C, 3 [Source:HGNC Symbol;Acc:HGNC:8716]
ENSG00000142733	318.9877	322.785	692.886	656.977	320.886	674.932	-1.072766152	1.08E-16	2.31E-15	MAP3K6	mitogen-activated protein kinase kinase kinase 6 [Source:HGNC Symbol;Acc:HGNC:6858]
ENSG00000239264	44.27589	41.7482	92.32	89.0481	43.0121	90.6841	-1.076064935	0.00194264	0.00765491	TXNDC5	thioredoxin domain containing 5 [Source:HGNC Symbol;Acc:HGNC:21073]
ENSG00000146374	169.0534	210.778	366.365	435.346	189.916	400.855	-1.077824806	2.09E-09	2.30E-08	RSPO3	R-spondin 3 [Source:HGNC Symbol;Acc:HGNC:20866]
ENSG00000171408	61.38249	50.9125	110.784	126.646	56.1475	118.715	-1.079752066	0.00046921	0.00214892	PDE7B	phosphodiesterase 7B [Source:HGNC Symbol;Acc:HGNC:8792]
ENSG00000139278	95.59568	87.5695	177.838	209.758	91.5826	193.798	-1.081061557	8.77E-06	5.75E-05	GLIPR1	GLI pathogenesis related 1 [Source:HGNC Symbol;Acc:HGNC:17001]
ENSG00000261324	47.2947	45.8212	103.982	93.9952	46.558	98.9884	-1.088317399	0.00111765	0.00468285	AC010168.2	novel transcript, overlapping HIST4H4
ENSG00000164308	67.42011	67.2045	137.022	149.403	67.3123	143.213	-1.0890956	8.92E-05	0.00047657	ERAP2	endoplasmic reticulum aminopeptidase 2 [Source:HGNC Symbol;Acc:HGNC:29499]
ENSG00000189423	51.31979	30.5475	91.3482	83.1115	40.9336	87.2299	-1.090925597	0.003526	0.01289575	USP32P3	ubiquitin specific peptidase 32 pseudogene 3 [Source:HGNC Symbol;Acc:HGNC:43576]
ENSG00000255150	45.28216	40.73	92.32	91.0269	43.0061	91.6735	-1.091817434	0.00163439	0.00655932	EID3	EP300 interacting inhibitor of differentiation 3 [Source:HGNC Symbol;Acc:HGNC:32961]
ENSG00000144355	196.2227	206.705	437.305	423.473	201.464	430.389	-1.095245164	1.15E-11	1.59E-10	DLX1	distal-less homeobox 1 [Source:HGNC Symbol;Acc:HGNC:2914]
ENSG00000183779	344.1444	340.095	748.278	714.363	342.12	731.321	-1.096044854	1.44E-18	3.48E-17	ZNF703	zinc finger protein 703 [Source:HGNC Symbol;Acc:HGNC:25883]
ENSG00000198763	11900.15	12544.8	25726.2	26583.8	12222.5	26155	-1.097566496	3.50E-242	2.35E-239	MT-ND2	mitochondrially encoded NADH:ubiquinone oxidoreductase core subunit 2 [Source:HGNC Symbol;Acc:HGNC:7456]
ENSG00000274471	32.20065	20.365	59.2792	53.4288	26.2828	56.354	-1.099855116	0.0153426	0.04506909	AC242376.2	hect domain and RLD 2 (HERC2) pseudogene
ENSG00000224543	49.30724	62.1132	142.853	95.974	55.7102	119.414	-1.100720298	0.00075763	0.00330185	SNRPGP15	small nuclear ribonucleoprotein polypeptide G pseudogene 15 [Source:HGNC Symbol;Acc:HGNC:49371]
ENSG00000265190	294.8372	285.11	630.692	616.411	289.974	623.551	-1.104564852	2.17E-16	4.54E-15	ANXA8	annexin A8 [Source:HGNC Symbol;Acc:HGNC:546]
ENSG00000166106	140.8778	131.354	278.904	306.721	136.116	292.812	-1.104908435	2.07E-08	2.00E-07	ADAMTS15	ADAM metallopeptidase with thrombospondin type 1 motif 15 [Source:HGNC Symbol;Acc:HGNC:16305]
ENSG00000112029	506.154	489.778	980.536	1171.48	497.966	1076.01	-1.111342485	2.54E-22	7.40E-21	FBXO5	F-box protein 5 [Source:HGNC Symbol;Acc:HGNC:13584]
ENSG00000248527	2496.557	2541.55	5259.33	5654.55	2519.05	5456.94	-1.11516341	2.38E-97	4.51E-95	MTATP6P1	MT-ATP6 pseudogene 1 [Source:HGNC Symbol;Acc:HGNC:44575]
ENSG00000030419	24.15049	28.511	60.251	54.4183	26.3307	57.3346	-1.123055638	0.01092742	0.03385092	IKZF2	IKAROS family zinc finger 2 [Source:HGNC Symbol;Acc:HGNC:13177]
ENSG00000242265	1927.008	2062.97	4204.93	4486.04	1994.99	4345.49	-1.123156082	5.20E-80	7.45E-78	PEG10	paternally expressed 10 [Source:HGNC Symbol;Acc:HGNC:14005]
ENSG00000178172	2553.914	2419.36	5566.41	5267.69	2486.64	5417.05	-1.123299247	5.57E-99	1.07E-96	SPINK6	serine peptidase inhibitor, Kazal type 6 [Source:HGNC Symbol;Acc:HGNC:29486]
ENSG00000185507	107.6709	101.825	254.609	201.842	104.748	228.226	-1.123739519	1.07E-06	8.15E-06	IRF7	interferon regulatory factor 7 [Source:HGNC Symbol;Acc:HGNC:6122]
ENSG00000196159	116.7274	103.861	212.822	268.134	110.294	240.478	-1.124099142	6.62E-07	5.18E-06	FAT4	FAT atypical cadherin 4 [Source:HGNC Symbol;Acc:HGNC:23109]
ENSG00000140015	99.62076	110.989	218.653	240.43	105.305	229.541	-1.124222687	4.70E-07	3.76E-06	KCNH5	potassium voltage-gated channel subfamily H member 5 [Source:HGNC Symbol;Acc:HGNC:6254]
ENSG00000258947	39.24454	44.803	94.2636	89.0481	42.0238	91.6558	-1.125306124	0.00127029	0.00526261	TUBB3	tubulin beta 3 class III [Source:HGNC Symbol;Acc:HGNC:20772]
ENSG00000198899	20608.42	21890.3	46072.6	46755.2	21249.4	46413.9	-1.127157967	4.76E-303	4.69E-300	MT-ATP6	mitochondrially encoded ATP synthase membrane subunit 6 [Source:HGNC Symbol;Acc:HGNC:7414]
ENSG00000109072	170.0597	170.048	375.111	369.055	170.054	372.083	-1.129655169	7.69E-11	9.86E-10	VTN	vitronectin [Source:HGNC Symbol;Acc:HGNC:12724]
ENSG00000197989	221.3795	174.121	423.7	442.272	197.75	432.986	-1.130253138	3.28E-11	4.36E-10	SNHG12	small nucleolar RNA host gene 12 [Source:HGNC Symbol;Acc:HGNC:30062]
ENSG00000099994	29.18184	28.511	66.0817	60.3548	28.8464	63.2183	-1.132034502	0.00694597	0.02314433	SUSD2	sushi domain containing 2 [Source:HGNC Symbol;Acc:HGNC:30667]
ENSG00000137877	21.13168	24.438	48.5895	51.45	22.7848	50.0197	-1.134577733	0.01619108	0.04713936	SPTBN5	spectrin beta, non-erythrocytic 5 [Source:HGNC Symbol;Acc:HGNC:15680]
ENSG00000180596	23.14422	34.6205	71.9124	55.4077	28.8824	63.6601	-1.141117565	0.00844328	0.02731607	HIST1H2BC	histone cluster 1 H2B family member c [Source:HGNC Symbol;Acc:HGNC:4757]
ENSG00000213347	119.7462	121.172	249.75	281.986	120.459	265.868	-1.142028698	4.09E-08	3.78E-07	MXD3	MAX dimerization protein 3 [Source:HGNC Symbol;Acc:HGNC:14008]
ENSG00000167565	153.9594	155.792	345.957	338.383	154.876	342.17	-1.143649656	2.89E-10	3.50E-09	SERTAD3	SERTA domain containing 3 [Source:HGNC Symbol;Acc:HGNC:17931]
ENSG00000170684	30.18811	40.73	78.715	78.1644	35.459	78.4397	-1.145902126	0.00274136	0.01041842	ZNF296	zinc finger protein 296 [Source:HGNC Symbol;Acc:HGNC:15981]
ENSG00000119938	106.6647	103.861	249.75	216.684	105.263	233.217	-1.147814157	2.62E-07	2.18E-06	PPP1R3C	protein phosphatase 1 regulatory subunit 3C [Source:HGNC Symbol;Acc:HGNC:9293]
ENSG00000157601	46.28843	51.9307	102.038	115.763	49.1096	108.9	-1.148945147	0.00037679	0.00176406	MX1	MX dynamin like GTPase 1 [Source:HGNC Symbol;Acc:HGNC:7532]
ENSG00000205220	61.38249	62.1132	144.797	129.614	61.7479	137.206	-1.152043135	6.05E-05	0.00033686	PSMB10	proteasome subunit beta 10 [Source:HGNC Symbol;Acc:HGNC:9538]
ENSG00000137203	704.3892	694.446	1580.13	1530.64	699.418	1555.38	-1.153062738	8.78E-40	5.14E-38	TFAP2A	transcription factor AP-2 alpha [Source:HGNC Symbol;Acc:HGNC:11742]
ENSG00000047936	182.1349	156.81	355.675	398.738	169.473	377.206	-1.153937902	1.17E-10	1.48E-09	ROS1	ROS proto-oncogene 1, receptor tyrosine kinase [Source:HGNC Symbol;Acc:HGNC:10261]
ENSG00000273899	45.28216	35.6387	87.4611	93.0058	40.4605	90.2334	-1.156695799	0.00117128	0.00488948	NOL12	nucleolar protein 12 [Source:HGNC Symbol;Acc:HGNC:28585]
ENSG00000251348	35.21946	38.6935	68.9971	95.974	36.9565	82.4856	-1.158047745	0.00230562	0.00892559	HSPD1P11	heat shock protein family D (Hsp60) member 1 pseudogene 11 [Source:HGNC Symbol;Acc:HGNC:35142]
ENSG00000019144	749.6714	852.275	1786.15	1791.85	800.973	1789	-1.159496705	6.03E-41	3.60E-39	PHLDB1	pleckstrin homology like domain family B member 1 [Source:HGNC Symbol;Acc:HGNC:23697]
ENSG00000139155	17.1066	28.511	56.3638	45.5135	22.8088	50.9386	-1.160189587	0.01676796	0.04853172	SLCO1C1	solute carrier organic anion transporter family member 1C1 [Source:HGNC Symbol;Acc:HGNC:13819]
ENSG00000137440	34.21319	31.5657	74.8278	72.2279	32.8895	73.5279	-1.160583865	0.00295598	0.01108848	FGFBP1	fibroblast growth factor binding protein 1 [Source:HGNC Symbol;Acc:HGNC:19695]

ENSG00000105516	107.6709	88.5877	242.947	195.906	98.1293	219.427	-1.160954025	1.10E-06	8.32E-06	DBP	D-box binding PAR bZIP transcription factor [Source:HGNC Symbol;Acc:HGNC:2697]
ENSG00000281207	22.13795	26.4745	60.251	48.4817	24.3062	54.3663	-1.161952576	0.011353	0.03489104	SLFNL1-AS1	SLFNL1 antisense RNA 1 [Source:HGNC Symbol;Acc:HGNC:44126]
ENSG00000179841	45.28216	48.876	93.2918	117.741	47.0791	105.517	-1.164133677	0.00046916	0.00214892	AKAP5	A-kinase anchoring protein 5 [Source:HGNC Symbol;Acc:HGNC:375]
ENSG00000081189	31.19438	28.511	68.0253	66.2913	29.8527	67.1583	-1.169595664	0.00424301	0.01515233	MEF2C	myocyte enhancer factor 2C [Source:HGNC Symbol;Acc:HGNC:6996]
ENSG00000239900	116.7274	94.6972	237.117	240.43	105.712	238.773	-1.175159796	1.29E-07	1.12E-06	ADSL	adenylosuccinate lyase [Source:HGNC Symbol;Acc:HGNC:291]
ENSG00000180592	26.16303	22.4015	55.392	54.4183	24.2823	54.9051	-1.176813471	0.00943672	0.02993027	SKIDA1	SKI/DACH domain containing 1 [Source:HGNC Symbol;Acc:HGNC:32697]
ENSG00000137801	1389.659	1512.1	3260.35	3310.61	1450.88	3285.48	-1.179273989	4.88E-74	6.31E-72	THBS1	thrombospondin 1 [Source:HGNC Symbol;Acc:HGNC:11785]
ENSG00000142227	934.8251	859.403	2124.33	1945.21	897.114	2034.77	-1.181482684	5.25E-46	3.69E-44	EMP3	epithelial membrane protein 3 [Source:HGNC Symbol;Acc:HGNC:3335]
ENSG00000130589	1050.546	953.082	2357.56	2187.61	1001.81	2272.59	-1.181671482	2.29E-50	1.75E-48	HELZ2	helicase with zinc finger 2 [Source:HGNC Symbol;Acc:HGNC:30021]
ENSG00000189060	4126.715	3940.63	9061.94	9282.77	4033.67	9172.35	-1.185123993	5.86E-174	2.47E-171	H1F0	H1 histone family member 0 [Source:HGNC Symbol;Acc:HGNC:4714]
ENSG00000163491	40.25081	18.3285	58.3074	75.1962	29.2897	66.7518	-1.187116412	0.00849236	0.02744216	NEK10	NIMA related kinase 10 [Source:HGNC Symbol;Acc:HGNC:18592]
ENSG00000183486	41.25708	47.8577	96.2072	106.858	44.5574	101.532	-1.188302262	0.00041134	0.00190939	MX2	MX dynamin like GTPase 2 [Source:HGNC Symbol;Acc:HGNC:7533]
ENSG00000206337	33.20692	35.6387	74.8278	82.1221	34.4228	78.475	-1.188857336	0.00177295	0.00705403	HCP5	HLA complex P5 [Source:HGNC Symbol;Acc:HGNC:21659]
ENSG00000123496	84.52671	100.807	196.302	227.567	92.6667	211.934	-1.193580693	5.54E-07	4.39E-06	IL13RA2	interleukin 13 receptor subunit alpha 2 [Source:HGNC Symbol;Acc:HGNC:5975]
ENSG00000153294	24.15049	31.5657	59.2792	68.2702	27.8581	63.7747	-1.195125505	0.0050942	0.01768472	ADGRF4	adhesion G protein-coupled receptor F4 [Source:HGNC Symbol;Acc:HGNC:19011]
ENSG00000158050	368.2949	298.347	753.137	774.718	333.321	763.928	-1.196199063	1.43E-19	3.68E-18	DUSP2	dual specificity phosphatase 2 [Source:HGNC Symbol;Acc:HGNC:3068]
ENSG00000197565	350.1821	306.493	746.335	759.877	328.338	753.106	-1.197449692	6.41E-21	1.78E-19	COL4A6	collagen type IV alpha 6 chain [Source:HGNC Symbol;Acc:HGNC:2208]
ENSG00000205413	861.3674	905.224	1984.39	2071.85	883.296	2028.12	-1.199199304	3.41E-52	2.76E-50	SAMD9	sterile alpha motif domain containing 9 [Source:HGNC Symbol;Acc:HGNC:1348]
ENSG00000053918	27.1693	31.5657	73.856	61.3442	29.3675	67.6001	-1.203284452	0.0036382	0.01323616	KCNQ1	potassium voltage-gated channel subfamily Q member 1 [Source:HGNC Symbol;Acc:HGNC:6294]
ENSG00000164850	62.38876	83.4965	168.202	168.12	72.9426	168.161	-1.205452018	6.85E-06	4.57E-05	GPER1	G protein-coupled estrogen receptor 1 [Source:HGNC Symbol;Acc:HGNC:4485]
ENSG00000156398	36.22573	52.949	99.1226	106.858	44.5874	102.99	-1.20828271	0.00042825	0.00197854	SFXN2	sideroflexin 2 [Source:HGNC Symbol;Acc:HGNC:16086]
ENSG00000163485	102.6396	75.3505	211.85	199.863	88.995	205.857	-1.209454192	8.54E-07	6.59E-06	ADORA1	adenosine A1 receptor [Source:HGNC Symbol;Acc:HGNC:262]
ENSG00000181143	284.7745	341.114	663.732	783.623	312.944	723.678	-1.209510562	8.72E-18	2.01E-16	MUC16	mucin 16, cell surface associated [Source:HGNC Symbol;Acc:HGNC:15582]
ENSG00000272468	18.11287	23.4197	52.4766	43.5346	20.7663	48.0056	-1.209609577	0.01388648	0.0414014	AL021807.1	novel transcript
ENSG00000138642	276.7243	248.453	585.989	630.263	262.589	608.126	-1.211311072	1.12E-17	2.57E-16	HERC6	HECT and RLD domain containing E3 ubiquitin protein ligase family member 6 [Source:HGNC Symbol;Acc:HGNC:26072]
ENSG00000073756	229.4296	251.508	525.738	589.696	240.469	557.717	-1.213685194	3.68E-16	7.55E-15	PTGS2	prostaglandin-endoperoxide synthase 2 [Source:HGNC Symbol;Acc:HGNC:9605]
ENSG00000129757	669.1698	670.008	1589.85	1534.6	669.589	1562.22	-1.22229713	8.86E-44	5.76E-42	CDKN1C	cyclin dependent kinase inhibitor 1C [Source:HGNC Symbol;Acc:HGNC:1786]
ENSG00000126705	486.0286	515.234	1242.92	1097.27	500.631	1170.09	-1.225023885	3.19E-30	1.29E-28	AHDC1	AT-hook DNA binding motif containing 1 [Source:HGNC Symbol;Acc:HGNC:25230]
ENSG00000069702	263.6428	234.197	593.766	578.813	248.92	586.288	-1.235773544	7.34E-18	1.70E-16	TGFBR3	transforming growth factor beta receptor 3 [Source:HGNC Symbol;Acc:HGNC:11774]
ENSG00000100342	188.1725	174.121	417.87	440.293	181.147	429.081	-1.243902748	6.90E-14	1.18E-12	APOL1	apolipoprotein L1 [Source:HGNC Symbol;Acc:HGNC:618]
ENSG00000260804	44.27589	37.6752	83.5739	110.815	40.9756	97.1947	-1.245516569	0.00044161	0.00203516	LINC01963	long intergenic non-protein coding RNA 1963 [Source:HGNC Symbol;Acc:HGNC:25283]
ENSG00000128284	64.4013	78.4052	182.696	156.329	71.4033	169.513	-1.247830898	3.19E-06	2.25E-05	APOL3	apolipoprotein L3 [Source:HGNC Symbol;Acc:HGNC:14868]
ENSG00000169962	22.13795	16.292	37.8998	53.4288	19.215	45.6643	-1.247932782	0.01524818	0.04482867	TAS1R3	taste 1 receptor member 3 [Source:HGNC Symbol;Acc:HGNC:15661]
ENSG00000285294	30.18811	44.803	101.066	77.175	37.4955	89.1206	-1.249945985	0.00091294	0.00390944	LINC00842	long intergenic non-protein coding RNA 842 [Source:HGNC Symbol;Acc:HGNC:44989]
ENSG00000185567	3953.636	3819.45	9496.33	9093.79	3886.55	9295.06	-1.257972443	4.77E-189	2.20E-186	AHNAK2	AHNAK nucleoprotein 2 [Source:HGNC Symbol;Acc:HGNC:20125]
ENSG00000279233	34.21319	40.73	85.5175	93.9952	37.4716	89.7563	-1.260377964	0.00048763	0.00221951	AC122688.3	novel transcript
ENSG00000273983	24.15049	16.292	53.4484	43.5346	20.2212	48.4915	-1.261499398	0.01127254	0.03470855	HIST1H3G	histone cluster 1 H3 family member g [Source:HGNC Symbol;Acc:HGNC:4772]
ENSG00000100297	1834.431	1731.02	4255.47	4294.1	1782.73	4274.78	-1.261673672	1.89E-108	3.88E-106	MCM5	minichromosome maintenance complex component 5 [Source:HGNC Symbol;Acc:HGNC:6948]
ENSG00000231131	166.0346	183.285	430.503	407.642	174.66	419.073	-1.262879964	7.94E-14	1.35E-12	LNCAROD	lncRNA activating regulator of DKK1 [Source:HGNC Symbol;Acc:HGNC:50913]
ENSG00000171345	273.7055	225.033	611.256	589.696	249.369	600.476	-1.26757183	6.03E-18	1.40E-16	KRT19	keratin 19 [Source:HGNC Symbol;Acc:HGNC:6436]
ENSG00000163362	78.48908	75.3505	184.64	186.012	76.9198	185.326	-1.26856033	4.07E-07	3.28E-06	INAVA	innate immunity activator [Source:HGNC Symbol;Acc:HGNC:25599]
ENSG00000173702	22.13795	25.4562	51.5049	63.3231	23.7971	57.414	-1.270578466	0.00504571	0.01754116	MUC13	mucin 13, cell surface associated [Source:HGNC Symbol;Acc:HGNC:7511]
ENSG00000115844	95.59568	85.533	221.568	215.694	90.5643	218.631	-1.271337567	4.27E-08	3.94E-07	DLX2	distal-less homeobox 2 [Source:HGNC Symbol;Acc:HGNC:2915]
ENSG00000243988	152.9531	117.099	338.183	315.626	135.026	326.904	-1.275313265	1.44E-10	1.79E-09	RPS24P17	ribosomal protein S24 pseudogene 17 [Source:HGNC Symbol;Acc:HGNC:36348]
ENSG00000233101	50.31352	48.876	118.558	121.699	49.5948	120.129	-1.276239317	4.05E-05	0.00023285	HOXB-AS3	HOXB cluster antisense RNA 3 [Source:HGNC Symbol;Acc:HGNC:40283]
ENSG00000280798	85.53298	108.953	236.145	237.462	97.2428	236.803	-1.284397981	1.84E-08	1.80E-07	LINC00294	long intergenic non-protein coding RNA 294 [Source:HGNC Symbol;Acc:HGNC:27456]
ENSG00000204642	45.28216	35.6387	93.2918	103.889	40.4605	98.5906	-1.284415665	0.0002285	0.00111908	HLA-F	major histocompatibility complex, class I, F [Source:HGNC Symbol;Acc:HGNC:4963]
ENSG00000130749	655.082	681.209	1614.14	1646.4	668.146	1630.27	-1.286915357	2.50E-49	1.87E-47	ZC3H4	zinc finger CCCH-type containing 4 [Source:HGNC Symbol;Acc:HGNC:17808]
ENSG00000189057	711.4331	699.538	1686.06	1760.18	705.485	1723.12	-1.288256623	1.52E-51	1.19E-49	FAM111B	family with sequence similarity 111 member B [Source:HGNC Symbol;Acc:HGNC:24200]
ENSG00000106003	236.4735	249.471	642.353	546.162	242.972	594.257	-1.290560488	4.07E-18	9.61E-17	LFNG	LFNG O-fucosylpeptide 3-beta-N-acetylglucosaminyltransferase [Source:HGNC Symbol;Acc:HGNC:6560]
ENSG00000089127	301.8811	298.347	746.335	724.258	300.114	735.296	-1.292833217	3.79E-24	1.20E-22	OAS1	2'-5'-oligoadenylate synthetase 1 [Source:HGNC Symbol;Acc:HGNC:8086]
ENSG00000182985	77.48281	93.679	217.681	202.832	85.5809	210.256	-1.297181752	6.84E-08	6.17E-07	CADM1	cell adhesion molecule 1 [Source:HGNC Symbol;Acc:HGNC:5951]
ENSG00000186487	21.13168	29.5292	64.1381	60.3548	25.3305	62.2465	-1.297737174	0.00309722	0.01154204	MYT1L	myelin transcription factor 1 like [Source:HGNC Symbol;Acc:HGNC:7623]
ENSG00000280152	20.12541	17.3102	51.5049	40.5663	18.7178	46.0356	-1.298395177	0.01059015	0.03295135	AC009078.3	TEC
ENSG00000111331	1616.07	1599.67	3958.1	3955.71	1607.87	3956.91	-1.299207424	1.28E-112	2.78E-110	OAS3	2'-5'-oligoadenylate synthetase 3 [Source:HGNC Symbol;Acc:HGNC:8088]
ENSG00000267519	554.4549	620.114	1500.44	1390.14	587.285	1445.29	-1.2994732	1.86E-40	1.10E-38	AC020916.1	novel transcript
ENSG00000007968	306.9124	319.73	784.234	768.782	313.321	776.508	-1.309449433	8.06E-26	2.77E-24	E2F2	E2F transcription factor 2 [Source:HGNC Symbol;Acc:HGNC:3114]
ENSG00000124249	105.6584	97.752	263.355	241.419	101.705	252.387	-1.311228276	1.83E-09	2.03E-08	KCNK15	potassium two pore domain channel subfamily K member 15 [Source:HGNC Symbol;Acc:HGNC:13814]
ENSG00000127329	125.7838	93.679	264.327	280.996	109.731	272.661	-1.312616176	1.83E-09	2.04E-08	PTPRB	protein tyrosine phosphatase, receptor type B [Source:HGNC Symbol;Acc:HGNC:9665]
ENSG00000213713	34.21319	37.6752	91.3482	88.0587	35.9442	89.7034	-1.319613168	0.00027175	0.00131217	PIGCP1	phosphatidylinositol glycan anchor biosynthesis class C pseudogene 1 [Source:HGNC Symbol;Acc:HGNC:8961]
ENSG00000152402	147.9217	150.701	351.788	395.769	149.311	373.779	-1.323744341	2.89E-13	4.63E-12	GUCY1A2	guanylate cyclase 1 soluble subunit alpha 2 [Source:HGNC Symbol;Acc:HGNC:4684]
ENSG00000023445	129.8089	122.19	304.17	328.488	125.999	316.329	-1.327813254	1.06E-11	1.48E-10	BIRC3	baculoviral IAP repeat containing 3 [Source:HGNC Symbol;Acc:HGNC:591]
ENSG00000146094	60.37622	49.8942	155.486	121.699	55.1352	138.593	-1.329796729	1.11E-05	7.12E-05	DOK3	docking protein 3 [Source:HGNC Symbol;Acc:HGNC:24583]
ENSG00000184678	98.61449	93.679	238.089	245.377	96.1467	241.733	-1.329978369	2.02E-09	2.22E-08	HIST2H2BE	histone cluster 2 H2B family member e [Source:HGNC Symbol;Acc:HGNC:4760]
ENSG00000171522	55.34487	53.9672	140.91	134.562	54.6561	137.736	-1.333465172	5.41E-06	3.67E-05	PTGER4	prostaglandin E receptor 4 [Source:HGNC Symbol;Acc:HGNC:9596]
ENSG00000171246	2643.472	2496.75	6487.67	6474.78	2570.11	6481.23	-1.334362329	1.26E-165	4.55E-163	NPTX1	neuronal pentraxin 1 [Source:HGNC Symbol;Acc:HGNC:7952]
ENSG00000198938	34610.67	34636.8	85814.9	89116.3	34623.7	87465.6	-1.336946078	0	0	MT-CO3	mitochondrially encoded cytochrome c oxidase III [Source:HGNC Symbol;Acc:HGNC:7422]
ENSG00000279806	62.38876	86.5512	183.668	192.938	74.47	188.303	-1.338775945	2.90E-07	2.41E-06	AC018629.1	TEC
ENSG00000279348	26.16303	20.365	51.5049	66.2913	23.264	58.8981	-1.33939999	0.00331694	0.01223738	AC012513.3	TEC
ENSG00000150347	746.6526	730.085	1792.95	1949.16	738.369	1871.06	-1.341314566	8.45E-56	7.65E-54	ARID5B	AT-rich interaction domain 5B [Source:HGNC Symbol;Acc:HGNC:17362]
ENSG00000137959	404.5207	430.72	1016.49	1101.23	417.62	1058.86	-1.342252881	6.30E-34	3.08E-32	IFI44L	interferon induced protein 44 like [Source:HGNC Symbol;Acc:HGNC:17817]

ENSG00000090539	74.464	64.1497	181.725	170.181	69.3069	175.953	-1.343954035	3.00E-07	2.47E-06	CHRD	chordin [Source:HGNC Symbol;Acc:HGNC:1949]
ENSG00000117228	59.36995	54.9855	146.74	144.456	57.1777	145.598	-1.348356824	2.42E-06	1.74E-05	GBP1	guanylate binding protein 1 [Source:HGNC Symbol;Acc:HGNC:4182]
ENSG00000128422	2627.372	2702.43	7109.61	6480.72	2664.9	6795.17	-1.350525929	3.17E-141	9.55E-139	KRT17	keratin 17 [Source:HGNC Symbol;Acc:HGNC:6427]
ENSG00000041982	2607.246	2628.1	6588.73	6799.32	2617.67	6694.03	-1.354567869	3.54E-181	1.59E-178	TNC	tenascin C [Source:HGNC Symbol;Acc:HGNC:5318]
ENSG00000273802	23.14422	14.2555	58.3074	37.5981	18.6999	47.9527	-1.35834515	0.00927716	0.02950025	HIST1H2BG	histone cluster 1 H2B family member g [Source:HGNC Symbol;Acc:HGNC:4746]
ENSG00000121858	42.26335	42.7665	106.897	112.794	42.5149	109.846	-1.369386309	3.30E-05	0.00019287	TNFSF10	TNF superfamily member 10 [Source:HGNC Symbol;Acc:HGNC:11925]
ENSG00000163131	29.18184	36.657	83.5739	87.0692	32.9194	85.3216	-1.374302477	0.00026882	0.00129886	CTSS	cathepsin S [Source:HGNC Symbol;Acc:HGNC:2545]
ENSG00000121671	141.8841	135.427	387.744	338.383	138.656	363.063	-1.388803568	1.05E-13	1.76E-12	CRY2	cryptochrome circadian regulator 2 [Source:HGNC Symbol;Acc:HGNC:2385]
ENSG00000149573	240.4986	202.632	593.764	567.929	221.565	580.846	-1.390205302	1.90E-20	5.12E-19	MPZL2	myelin protein zero like 2 [Source:HGNC Symbol;Acc:HGNC:3496]
ENSG00000169242	459.8655	442.939	1283.73	1088.37	451.402	1186.05	-1.393791028	3.32E-35	1.67E-33	EFNA1	ephrin A1 [Source:HGNC Symbol;Acc:HGNC:3221]
ENSG00000269821	168.0471	191.431	503.387	441.283	179.739	472.335	-1.394264628	7.01E-17	1.51E-15	KCNQ1OT1	KCNQ1 opposite strand/antisense transcript 1 [Source:HGNC Symbol;Acc:HGNC:6295]
ENSG00000183018	15.09405	20.365	42.7588	50.4606	17.7295	46.6097	-1.394769402	0.00652458	0.0219084	SPNS2	sphingolipid transporter 2 [Source:HGNC Symbol;Acc:HGNC:26992]
ENSG00000111859	64.4013	89.606	197.273	207.779	77.0036	202.526	-1.39557008	4.14E-08	3.83E-07	NEDD9	neural precursor cell expressed, developmentally down-regulated 9 [Source:HGNC Symbol;Acc:HGNC:7733]
ENSG00000279092	17.1066	20.365	53.4484	46.5029	18.7358	49.9757	-1.41590418	0.0041414	0.01484487	AC025678.3	TEC
ENSG00000179094	279.7431	322.785	855.175	762.845	301.264	809.01	-1.425470719	2.42E-27	8.84E-26	PER1	period circadian regulator 1 [Source:HGNC Symbol;Acc:HGNC:8845]
ENSG00000230615	34.21319	39.7117	106.897	92.0163	36.9625	99.4566	-1.428446711	5.24E-05	0.00029533	AL139220.2	uncharacterized LOC107984948 [Source:NCBI gene;Acc:107984948]
ENSG00000260260	55.34487	41.7482	135.079	128.625	48.5466	131.852	-1.441066587	3.37E-06	2.37E-05	SNHG19	small nucleolar RNA host gene 19 [Source:HGNC Symbol;Acc:HGNC:49574]
ENSG00000258839	43.26962	26.4745	99.1226	91.0269	34.8721	95.0747	-1.446308721	0.0001076	0.00056483	MC1R	melanocortin 1 receptor [Source:HGNC Symbol;Acc:HGNC:6929]
ENSG00000113369	893.568	984.648	2684.08	2448.82	939.108	2566.45	-1.450631434	4.65E-75	6.25E-73	ARRDC3	arrestin domain containing 3 [Source:HGNC Symbol;Acc:HGNC:29263]
ENSG00000179954	18.11287	16.292	41.787	52.4394	17.2024	47.1132	-1.453051712	0.00460851	0.01623538	SSC5D	scavenger receptor cysteine rich family member with 5 domains [Source:HGNC Symbol;Acc:HGNC:26641]
ENSG00000102048	83.52043	108.953	261.411	266.155	96.2366	263.783	-1.455108337	7.03E-11	9.04E-10	ASB9	ankyrin repeat and SOCS box containing 9 [Source:HGNC Symbol;Acc:HGNC:17184]
ENSG00000108984	132.8277	133.391	373.167	362.129	133.109	367.648	-1.46576035	1.47E-15	2.88E-14	MAP2K6	mitogen-activated protein kinase kinase 6 [Source:HGNC Symbol;Acc:HGNC:6846]
ENSG00000100867	33.20692	27.4927	76.7714	92.0163	30.3498	84.3939	-1.474903634	0.00013856	0.00071066	DHRS2	dehydrogenase/reductase 2 [Source:HGNC Symbol;Acc:HGNC:18349]
ENSG00000213928	22.13795	17.3102	61.2228	48.4817	19.7241	54.8522	-1.475457566	0.00212187	0.00829693	IRF9	interferon regulatory factor 9 [Source:HGNC Symbol;Acc:HGNC:6131]
ENSG00000166444	246.5362	271.873	702.604	745.036	259.204	723.82	-1.481634234	3.03E-28	1.16E-26	ST5	suppression of tumorigenicity 5 [Source:HGNC Symbol;Acc:HGNC:11350]
ENSG00000118971	17.1066	17.3102	39.8434	56.3971	17.2084	48.1202	-1.483129039	0.00395727	0.0142506	CCND2	cyclin D2 [Source:HGNC Symbol;Acc:HGNC:1583]
ENSG00000100918	24.15049	26.4745	69.9689	73.2173	25.3125	71.5931	-1.500076128	0.00031414	0.0014924	REC8	REC8 meiotic recombination protein [Source:HGNC Symbol;Acc:HGNC:16879]
ENSG00000269858	74.464	86.5512	213.794	242.409	80.5076	228.101	-1.502582326	2.96E-10	3.58E-09	EGLN2	egl-9 family hypoxia inducible factor 2 [Source:HGNC Symbol;Acc:HGNC:14660]
ENSG00000179133	22.13795	23.4197	58.3074	71.2385	22.7788	64.7729	-1.507546605	0.0006337	0.00280147	C10orf67	chromosome 10 open reading frame 67 [Source:HGNC Symbol;Acc:HGNC:28716]
ENSG00000105939	1469.155	1504.97	4113.59	4360.39	1487.06	4236.99	-1.510549876	2.02E-139	5.72E-137	ZC3HAV1	zinc finger CCCH-type containing, antiviral 1 [Source:HGNC Symbol;Acc:HGNC:23721]
ENSG00000168675	20.12541	11.2007	37.8998	51.45	15.6631	44.6749	-1.510793611	0.00575463	0.01960609	LDLRAD4	low density lipoprotein receptor class A domain containing 4 [Source:HGNC Symbol;Acc:HGNC:1224]
ENSG00000133083	30.18811	34.6205	80.6586	104.879	32.4043	92.7687	-1.517374243	5.17E-05	0.00029173	DCLK1	doublecortin like kinase 1 [Source:HGNC Symbol;Acc:HGNC:2700]
ENSG00000049246	229.4296	248.453	656.93	713.374	238.941	685.152	-1.519805638	9.00E-28	3.36E-26	PER3	period circadian regulator 3 [Source:HGNC Symbol;Acc:HGNC:8847]
ENSG00000260774	17.1066	19.3467	57.3356	47.4923	18.2267	52.414	-1.524351559	0.00189497	0.00748907	AC021087.2	novel transcript
ENSG00000172183	70.43892	53.9672	186.012	172.007	62.2031	179.009	-1.524431081	1.87E-08	1.82E-07	ISG20	interferon stimulated exonuclease gene 20 [Source:HGNC Symbol;Acc:HGNC:6130]
ENSG00000237330	26.16303	19.3467	78.715	52.4394	22.7549	65.5772	-1.526978975	0.00078975	0.0034297	RNF223	ring finger protein 223 [Source:HGNC Symbol;Acc:HGNC:40020]
ENSG00000053438	13.08151	10.1825	34.9844	32.651	11.632	33.8177	-1.539319694	0.01156969	0.03540952	NNAT	neuronatin [Source:HGNC Symbol;Acc:HGNC:7860]
ENSG00000115267	143.8967	132.372	380.942	424.463	138.135	402.702	-1.543366463	1.45E-17	3.29E-16	IFIH1	interferon induced with helicase C domain 1 [Source:HGNC Symbol;Acc:HGNC:18873]
ENSG00000132326	82.51416	86.5512	242.947	250.324	84.5327	246.636	-1.544848476	9.27E-12	1.30E-10	PER2	period circadian regulator 2 [Source:HGNC Symbol;Acc:HGNC:8846]
ENSG00000177409	102.6396	126.263	334.296	333.436	114.451	333.866	-1.544890701	9.32E-15	1.69E-13	SAMD9L	sterile alpha motif domain containing 9 like [Source:HGNC Symbol;Acc:HGNC:1349]
ENSG00000117461	329.0504	307.511	929.031	940.941	318.281	934.986	-1.554511576	2.40E-39	1.38E-37	PIK3R3	phosphoinositide-3-kinase regulatory subunit 3 [Source:HGNC Symbol;Acc:HGNC:8981]
ENSG00000172575	38.23827	46.8395	110.784	140.498	42.5389	125.641	-1.562524152	1.75E-06	1.28E-05	RASGRP1	RAS guanyl releasing protein 1 [Source:HGNC Symbol;Acc:HGNC:9878]
ENSG00000065618	11.06897	10.1825	29.1537	33.6404	10.6257	31.397	-1.56273529	0.01365652	0.04082299	COL17A1	collagen type XVII alpha 1 chain [Source:HGNC Symbol;Acc:HGNC:2194]
ENSG00000139874	752.6902	693.428	2167.09	2134.19	723.059	2150.64	-1.572461399	8.09E-84	1.24E-81	SSTR1	somatostatin receptor 1 [Source:HGNC Symbol;Acc:HGNC:11330]
ENSG00000129173	122.765	103.861	317.775	358.171	113.313	337.973	-1.576169428	3.66E-15	6.93E-14	E2F8	E2F transcription factor 8 [Source:HGNC Symbol;Acc:HGNC:24727]
ENSG00000074181	14.08778	22.4015	54.4202	54.4183	18.2446	54.4192	-1.577446874	0.00131852	0.00543044	NOTCH3	notch 3 [Source:HGNC Symbol;Acc:HGNC:7883]
ENSG00000258725	13.08151	8.146	24.2947	39.5769	10.6138	31.9358	-1.587884464	0.01478603	0.04374755	PRC1-AS1	PRC1 antisense RNA 1 [Source:HGNC Symbol;Acc:HGNC:48587]
ENSG00000111291	9.056433	10.1825	30.1255	27.7038	9.61947	28.9147	-1.588092037	0.01641175	0.04768777	GPRC5D	G protein-coupled receptor class C group 5 member D [Source:HGNC Symbol;Acc:HGNC:13310]
ENSG00000182600	10.0627	15.2737	40.8152	35.6192	12.6682	38.2172	-1.593909999	0.00629811	0.02121552	SNORC	secondary ossification center associated regulator of chondrocyte maturation [Source:HGNC Symbol;Acc:HGNC:33763]
ENSG00000205085	8.050162	16.292	34.9844	38.5875	12.1711	36.786	-1.596763181	0.00829406	0.02696919	FAM71F2	family with sequence similarity 71 member F2 [Source:HGNC Symbol;Acc:HGNC:27998]
ENSG00000134986	266.6616	243.362	723.983	820.232	255.012	772.108	-1.597951004	6.71E-32	2.97E-30	NREP	neuronal regeneration related protein [Source:HGNC Symbol;Acc:HGNC:16834]
ENSG00000268089	246.5362	211.796	659.845	736.131	229.166	697.988	-1.606439938	4.77E-29	1.88E-27	GABRQ	gamma-aminobutyric acid type A receptor theta subunit [Source:HGNC Symbol;Acc:HGNC:14454]
ENSG00000240522	10.0627	10.1825	28.1819	33.6404	10.1226	30.9111	-1.610348493	0.01233692	0.03746251	RPL7AP10	ribosomal protein L7a pseudogene 10 [Source:HGNC Symbol;Acc:HGNC:32433]
ENSG00000165124	54.3386	49.8942	138.966	181.064	52.1164	160.015	-1.617951702	3.63E-08	3.38E-07	SVEP1	sushi, von Willebrand factor type A, EGF and pentraxin domain containing 1 [Source:HGNC Symbol;Acc:HGNC:15985]
ENSG00000104081	53.33233	50.9125	168.12	152.371	52.1224	160.245	-1.620343686	1.29E-08	1.29E-07	BMF	Bcl2 modifying factor [Source:HGNC Symbol;Acc:HGNC:24132]
ENSG00000124225	1424.879	1384.82	4331.27	4315.86	1404.85	4323.57	-1.621763103	1.79E-171	6.78E-169	PMEPA1	prostate transmembrane protein, androgen induced 1 [Source:HGNC Symbol;Acc:HGNC:14107]
ENSG00000139438	50.31352	48.876	164.232	142.477	49.5948	153.355	-1.628732785	2.68E-08	2.55E-07	FAM222A	family with sequence similarity 222 member A [Source:HGNC Symbol;Acc:HGNC:25915]
ENSG00000210176	7.043892	13.2372	32.0691	30.6721	10.1406	31.3706	-1.630421361	0.012306	0.0373763	MT-TH	mitochondrially encoded tRNA histidine [Source:HGNC Symbol;Acc:HGNC:7487]
ENSG00000104140	75.47027	77.387	226.427	247.356	76.4286	236.891	-1.631978752	3.96E-12	5.74E-11	RHOV	ras homolog family member V [Source:HGNC Symbol;Acc:HGNC:18313]
ENSG00000203814	16.10032	17.3102	41.787	63.3231	16.7053	52.555	-1.65317503	0.00118285	0.00492943	HIST2H2BF	histone cluster 2 H2B family member f [Source:HGNC Symbol;Acc:HGNC:24700]
ENSG00000126368	366.2824	371.661	1183.64	1137.84	368.972	1160.74	-1.653532867	6.53E-53	5.47E-51	NR1D1	nuclear receptor subfamily 1 group D member 1 [Source:HGNC Symbol;Acc:HGNC:7962]
ENSG00000121690	186.16	182.267	524.766	641.146	184.213	582.956	-1.661768356	4.81E-25	1.57E-23	DEPDC7	DEP domain containing 7 [Source:HGNC Symbol;Acc:HGNC:29899]
ENSG00000179772	22.13795	11.2007	57.3356	48.4817	16.6693	52.9087	-1.665353821	0.00124019	0.00515096	FOXS1	forkhead box S1 [Source:HGNC Symbol;Acc:HGNC:3735]
ENSG00000019186	119.7462	110.989	351.788	388.843	115.368	370.316	-1.682260643	1.58E-18	3.82E-17	CYP24A1	cytochrome P450 family 24 subfamily A member 1 [Source:HGNC Symbol;Acc:HGNC:2602]
ENSG00000246859	65.40757	46.8395	186.584	175.128	56.1235	180.856	-1.687649289	1.33E-09	1.51E-08	STARD4-AS1	STARD4 antisense RNA 1 [Source:HGNC Symbol;Acc:HGNC:44117]
ENSG00000137501	51.31979	65.168	193.386	184.033	58.2439	188.709	-1.696478713	2.80E-10	3.39E-09	SYTL2	synaptotagmin like 2 [Source:HGNC Symbol;Acc:HGNC:15585]
ENSG00000116701	62.38876	78.4052	245.863	216.684	70.397	231.273	-1.716564191	3.32E-12	4.83E-11	NCF2	neutrophil cytosolic factor 2 [Source:HGNC Symbol;Acc:HGNC:7661]
ENSG00000204103	15.09405	14.2555	50.5331	46.5029	14.6748	48.518	-1.725174976	0.00095448	0.00406612	MAFB	MAF bZIP transcription factor B [Source:HGNC Symbol;Acc:HGNC:6408]
ENSG00000212123	16.10032	23.4197	47.6177	84.101	19.76	65.8593	-1.736854177	0.00031423	0.0014924	PRR22	proline rich 22 [Source:HGNC Symbol;Acc:HGNC:28354]
ENSG00000141052	8.050162	8.146	22.3512	31.6615	8.09808	27.0064	-1.73725318	0.01388359	0.04140115	MYOCD	myocardin [Source:HGNC Symbol;Acc:HGNC:16067]
ENSG00000135477	69.43265	67.2045	241.004	219.652	68.3186	230.328	-1.753389277	5.24E-13	8.22E-12	KRT87P	keratin 87 pseudogene [Source:HGNC Symbol;Acc:HGNC:30198]

ENSG00000064205	273.7055	290.201	1030.1	877.618	281.953	953.858	-1.758574112	1.78E-42	1.12E-40	WISP2	WNT1 inducible signaling pathway protein 2 [Source:HGNC Symbol;Acc:HGNC:12770]
ENSG00000235363	36.22573	47.8577	203.104	83.1115	42.0417	143.108	-1.767519441	0.00792668	0.02592638	SNRPGP10	small nuclear ribonucleoprotein polypeptide G pseudogene 10 [Source:HGNC Symbol;Acc:HGNC:39329]
ENSG00000144802	119.7462	126.263	435.362	410.611	123.005	422.986	-1.782068218	5.28E-23	1.59E-21	NFKBIZ	NFKB inhibitor zeta [Source:HGNC Symbol;Acc:HGNC:29805]
ENSG00000223813	15.09405	10.1825	46.6459	41.5558	12.6383	44.1008	-1.802403193	0.00127507	0.005278	AC007255.1	novel transcript, antisense to CHN2
ENSG00000134516	25.15676	18.3285	67.0535	85.0904	21.7426	76.0719	-1.805980165	2.90E-05	0.00017127	DOCK2	dedicator of cytokinesis 2 [Source:HGNC Symbol;Acc:HGNC:2988]
ENSG00000138646	288.7996	306.493	1016.49	1072.53	297.646	1044.51	-1.811209127	7.37E-54	6.43E-52	HERC5	HECT and RLD domain containing E3 ubiquitin protein ligase 5 [Source:HGNC Symbol;Acc:HGNC:24368]
ENSG00000183508	7.043892	12.219	35.9562	31.6615	9.63144	33.8089	-1.81274869	0.00476587	0.01672585	TENT5C	terminal nucleotidyltransferase 5C [Source:HGNC Symbol;Acc:HGNC:24712]
ENSG00000189120	14.08778	18.3285	59.2792	55.4077	16.2081	57.3434	-1.823496209	0.00019823	0.00098188	SP6	Sp6 transcription factor [Source:HGNC Symbol;Acc:HGNC:14530]
ENSG00000183876	54.3386	39.7117	172.007	163.255	47.0252	167.631	-1.833259046	3.80E-10	4.54E-09	ARSI	arylsulfatase family member I [Source:HGNC Symbol;Acc:HGNC:32521]
ENSG00000081277	42.26335	45.8212	166.176	148.413	44.0423	157.295	-1.836798559	6.66E-10	7.77E-09	PKP1	plakophilin 1 [Source:HGNC Symbol;Acc:HGNC:9023]
ENSG00000171346	15.09405	13.2372	51.5049	51.45	14.1657	51.4774	-1.861286379	0.00032125	0.00152378	KRT15	keratin 15 [Source:HGNC Symbol;Acc:HGNC:6421]
ENSG00000147041	26.16303	21.3832	92.32	82.1221	23.7731	87.2211	-1.875090384	3.02E-06	2.14E-05	SYTL5	synaptotagmin like 5 [Source:HGNC Symbol;Acc:HGNC:15589]
ENSG00000170500	13.08151	18.3285	59.2792	56.3971	15.705	57.8381	-1.881509109	0.00013765	0.00070698	LONRF2	LON peptidase N-terminal domain and ring finger 2 [Source:HGNC Symbol;Acc:HGNC:24788]
ENSG00000115738	175.091	147.646	618.058	594.643	161.369	606.351	-1.909513658	4.43E-34	2.19E-32	ID2	inhibitor of DNA binding 2 [Source:HGNC Symbol;Acc:HGNC:5361]
ENSG00000282988	5.031352	7.12775	19.4358	26.7144	6.07955	23.0751	-1.924613984	0.01426685	0.04238136	AL031777.3	novel protein
ENSG00000168646	28.17557	20.365	101.066	84.101	24.2703	92.5836	-1.931148611	1.24E-06	9.30E-06	AXIN2	axin 2 [Source:HGNC Symbol;Acc:HGNC:904]
ENSG00000166407	56.35114	52.949	216.709	203.821	54.6501	210.265	-1.943862489	8.74E-14	1.48E-12	LMO1	LIM domain only 1 [Source:HGNC Symbol;Acc:HGNC:6641]
ENSG00000272734	67.42011	57.022	255.581	224.599	62.221	240.09	-1.947920409	4.20E-15	7.87E-14	ADIRF-AS1	ADIRF antisense RNA 1 [Source:HGNC Symbol;Acc:HGNC:45127]
ENSG00000266897	3.018811	9.16425	23.323	23.7462	6.09153	23.5346	-1.951828205	0.0145245	0.04303414	AC005546.1	novel transcript
ENSG00000007944	59.36995	57.022	236.145	218.663	58.196	227.404	-1.966273765	5.72E-15	1.06E-13	MYLIP	myosin regulatory light chain interacting protein [Source:HGNC Symbol;Acc:HGNC:21155]
ENSG00000285416	14.08778	12.219	44.7023	59.3654	13.1534	52.0339	-1.983407424	0.00018484	0.00092147	AC137630.5	novel pseudogene, ortholog of avian secreted frizzled-related protein 5
ENSG00000107984	979.101	956.137	3844.4	3858.75	967.619	3851.58	-1.992889428	6.32E-212	3.45E-209	DKK1	dickkopf WNT signaling pathway inhibitor 1 [Source:HGNC Symbol;Acc:HGNC:2891]
ENSG00000255176	9.056433	5.09125	30.1255	26.7144	7.07384	28.42	-2.005345064	0.00523645	0.01809769	AP000941.1	novel transcript
ENSG00000132854	133.834	122.19	505.331	525.384	128.012	515.357	-2.009066928	2.02E-32	9.21E-31	KANK4	KN motif and ankyrin repeat domains 4 [Source:HGNC Symbol;Acc:HGNC:27263]
ENSG00000078401	19.11914	8.146	62.1945	48.4817	13.6326	55.3381	-2.019953093	0.00017607	0.00088191	EDN1	endothelin 1 [Source:HGNC Symbol;Acc:HGNC:3176]
ENSG00000167074	229.4296	215.869	909.595	920.163	222.649	914.879	-2.038672524	4.27E-57	3.99E-55	TEF	TEF, PAR bZIP transcription factor [Source:HGNC Symbol;Acc:HGNC:11722]
ENSG00000184588	7.043892	3.05475	25.2665	16.8202	5.04932	21.0434	-2.058063586	0.01569863	0.04599593	PDE4B	phosphodiesterase 4B [Source:HGNC Symbol;Acc:HGNC:8781]
ENSG00000116883	11.06897	9.16425	50.5331	34.6298	10.1166	42.5814	-2.07353534	0.00051645	0.00233487	AL591845.1	novel transcript, antisense to EVA1B
ENSG00000171872	12.07524	8.146	44.7023	41.5558	10.1106	43.1291	-2.09206413	0.00038161	0.00178425	KLF17	Kruppel like factor 17 [Source:HGNC Symbol;Acc:HGNC:18830]
ENSG00000150551	9.056433	5.09125	35.9562	25.725	7.07384	30.8406	-2.123480836	0.00269832	0.01028133	LYPD1	LY6/PLAUR domain containing 1 [Source:HGNC Symbol;Acc:HGNC:28431]
ENSG00000198626	20.12541	24.438	96.2072	99.9317	22.2817	98.0695	-2.138310213	5.11E-08	4.68E-07	RYR2	ryanodine receptor 2 [Source:HGNC Symbol;Acc:HGNC:10484]
ENSG00000205755	8.050162	8.146	41.787	29.6827	8.09808	35.7348	-2.142088548	0.00105094	0.00443605	CRLF2	cytokine receptor like factor 2 [Source:HGNC Symbol;Acc:HGNC:14281]
ENSG00000276603	3.018811	7.12775	24.2947	20.7779	5.07328	22.5363	-2.153127312	0.00940179	0.02984511	AL109614.1	novel transcript, sense intronic to BLCAP
ENSG00000140519	7.043892	3.05475	25.2665	19.7885	5.04932	22.5275	-2.156150152	0.00944553	0.02995176	RHCG	Rh family C glycoprotein [Source:HGNC Symbol;Acc:HGNC:18140]
ENSG00000179674	12.07524	9.16425	37.8998	57.3865	10.6197	47.6432	-2.164508955	0.00017487	0.00087706	ARL14	ADP ribosylation factor like GTPase 14 [Source:HGNC Symbol;Acc:HGNC:22974]
ENSG00000265972	326.0316	379.807	1530.57	1646.4	352.919	1588.48	-2.170459356	6.95E-94	1.28E-91	TXNIP	thioredoxin interacting protein [Source:HGNC Symbol;Acc:HGNC:16952]
ENSG00000162006	17.1066	20.365	99.1226	73.2173	18.7358	86.1699	-2.202077045	3.22E-07	2.65E-06	MSLNL	mesothelin-like [Source:HGNC Symbol;Acc:HGNC:14170]
ENSG00000205583	20.12541	20.365	106.897	80.1433	20.2452	93.5201	-2.208034	8.90E-08	7.91E-07	STAG3L1	stromal antigen 3-like 1 (pseudogene) [Source:HGNC Symbol;Acc:HGNC:33852]
ENSG00000185885	15.09405	16.292	75.7996	70.249	15.693	73.0243	-2.218506962	1.34E-06	9.99E-06	IFITM1	interferon induced transmembrane protein 1 [Source:HGNC Symbol;Acc:HGNC:5412]
ENSG00000159208	9.056433	21.3832	77.7432	65.3019	15.2198	71.5226	-2.234243473	5.12E-06	3.49E-05	CIART	circadian associated repressor of transcription [Source:HGNC Symbol;Acc:HGNC:25200]
ENSG00000160678	5.031352	6.1095	26.2383	26.7144	5.57043	26.4764	-2.249251357	0.00321001	0.01190527	S100A1	S100 calcium binding protein A1 [Source:HGNC Symbol;Acc:HGNC:10486]
ENSG00000134215	60.37622	76.3687	341.098	310.679	68.3725	325.889	-2.253482058	9.98E-24	3.12E-22	VAV3	vav guanine nucleotide exchange factor 3 [Source:HGNC Symbol;Acc:HGNC:12659]
ENSG00000107201	266.6616	243.362	1162.26	1275.37	255.012	1218.81	-2.256563835	1.63E-80	2.36E-78	DDX58	DExD/H-box helicase 58 [Source:HGNC Symbol;Acc:HGNC:19102]
ENSG00000123838	3.018811	6.1095	18.464	25.725	4.56415	22.0945	-2.276370589	0.00753792	0.02485307	C4BPA	complement component 4 binding protein alpha [Source:HGNC Symbol;Acc:HGNC:1325]
ENSG00000135373	18.11287	28.511	122.446	108.837	23.3119	115.641	-2.311593889	1.10E-09	1.25E-08	EHF	ETS homologous factor [Source:HGNC Symbol;Acc:HGNC:3246]
ENSG00000158246	68.42638	66.1862	363.449	326.51	67.3063	344.98	-2.35774066	3.99E-27	1.44E-25	TENT5B	terminal nucleotidyltransferase 5B [Source:HGNC Symbol;Acc:HGNC:28273]
ENSG00000108771	11.06897	13.2372	66.0817	64.3125	12.1531	65.1971	-2.423922432	1.34E-06	9.98E-06	DHX58	DExH-box helicase 58 [Source:HGNC Symbol;Acc:HGNC:29517]
ENSG00000185614	7.043892	1.01825	29.1537	16.8202	4.03107	22.9869	-2.508894313	0.0057415	0.01957508	INKA1	inka box actin regulator 1 [Source:HGNC Symbol;Acc:HGNC:32480]
ENSG00000207547	5.031352	3.05475	24.2947	22.7567	4.04305	23.5257	-2.539640013	0.00281557	0.01064292	MIR25	microRNA 25 [Source:HGNC Symbol;Acc:HGNC:31609]
ENSG00000005102	28.17557	24.438	157.43	155.339	26.3068	156.385	-2.571269815	9.81E-15	1.77E-13	MEOX1	mesenchyme homeobox 1 [Source:HGNC Symbol;Acc:HGNC:7013]
ENSG00000137628	169.0534	184.303	1082.57	1052.75	176.678	1067.66	-2.5954864	3.29E-89	5.58E-87	DDX60	DExD/H-box helicase 60 [Source:HGNC Symbol;Acc:HGNC:25942]
ENSG00000134326	11.06897	13.2372	79.6868	79.1538	12.1531	79.4203	-2.708629938	1.22E-08	1.23E-07	CMPK2	cytidine/uridine monophosphate kinase 2 [Source:HGNC Symbol;Acc:HGNC:27015]
ENSG00000038295	5.031352	9.16425	45.6741	51.45	7.0978	48.5621	-2.775701067	7.70E-06	5.10E-05	TLL1	tolloid like 1 [Source:HGNC Symbol;Acc:HGNC:11843]
ENSG00000272512	3.018811	4.073	32.0691	16.8202	3.5459	24.4446	-2.786656267	0.00164128	0.00658341	AL645608.7	novel transcript
ENSG00000079385	4.025081	7.12775	44.7023	32.651	5.57641	38.6766	-2.795753802	6.53E-05	0.00036089	CEACAM1	carcinoembryonic antigen related cell adhesion molecule 1 [Source:HGNC Symbol;Acc:HGNC:1814]
ENSG00000137965	96.60195	90.6242	650.127	677.755	93.6131	663.941	-2.826069443	1.38E-62	1.42E-60	IFI44	interferon induced protein 44 [Source:HGNC Symbol;Acc:HGNC:16938]
ENSG00000187608	436.7213	399.154	3266.19	2973.22	417.938	3119.7	-2.899923533	1.68E-247	1.18E-244	ISG15	ISG15 ubiquitin-like modifier [Source:HGNC Symbol;Acc:HGNC:4053]
ENSG00000110446	4.025081	3.05475	27.2101	25.725	3.53992	26.4676	-2.901786775	0.000623	0.0027583	SLC15A3	solute carrier family 15 member 3 [Source:HGNC Symbol;Acc:HGNC:18068]
ENSG00000132274	4.025081	7.12775	38.8716	44.524	5.57641	41.6978	-2.903845519	2.06E-05	0.00012519	TRIM22	tripartite motif containing 22 [Source:HGNC Symbol;Acc:HGNC:16379]
ENSG00000124102	20.12541	6.1095	94.2636	104.879	13.1175	99.5712	-2.921636316	5.14E-10	6.07E-09	PI3	peptidase inhibitor 3 [Source:HGNC Symbol;Acc:HGNC:8947]
ENSG00000179776	2.012541	6.1095	33.0409	34.6298	4.06102	33.8353	-3.061223062	9.35E-05	0.00049696	CDH5	cadherin 5 [Source:HGNC Symbol;Acc:HGNC:1764]
ENSG00000126709	137.859	146.628	1246.81	1171.48	142.243	1209.14	-3.087771494	1.13E-119	2.78E-117	IFI6	interferon alpha inducible protein 6 [Source:HGNC Symbol;Acc:HGNC:4054]
ENSG00000197249	10.0627	4.073	59.2792	66.2913	7.06785	62.7853	-3.148717709	8.23E-08	7.34E-07	SERPINA1	serpin family A member 1 [Source:HGNC Symbol;Acc:HGNC:8941]
ENSG00000134827	30.18811	18.3285	267.242	238.451	24.2583	252.847	-3.380467681	3.96E-28	1.51E-26	TCN1	transcobalamin 1 [Source:HGNC Symbol;Acc:HGNC:11652]
ENSG00000119917	182.1349	170.048	1886.24	1978.85	176.091	1932.55	-3.455868011	1.43E-206	7.53E-204	IFIT3	interferon induced protein with tetratricopeptide repeats 3 [Source:HGNC Symbol;Acc:HGNC:5411]
ENSG00000127129	13.08151	8.146	145.768	105.868	10.6138	125.818	-3.566298322	3.22E-15	6.11E-14	EDN2	endothelin 2 [Source:HGNC Symbol;Acc:HGNC:3177]
ENSG00000165949	24.15049	13.2372	252.665	209.758	18.6939	231.212	-3.627065318	1.12E-26	3.96E-25	IFI27	interferon alpha inducible protein 27 [Source:HGNC Symbol;Acc:HGNC:5397]
ENSG00000111335	19.11914	14.2555	191.443	237.462	16.6873	214.452	-3.682767595	1.25E-25	4.24E-24	OAS2	2'-5'-oligoadenylate synthetase 2 [Source:HGNC Symbol;Acc:HGNC:8087]
ENSG00000133321	7.043892	8.146	123.417	119.72	7.59495	121.569	-4.001085098	2.60E-16	5.41E-15	RARRES3	retinoic acid receptor responder 3 [Source:HGNC Symbol;Acc:HGNC:9869]
ENSG00000185745	137.859	142.555	2560.67	2686.28	140.207	2623.47	-4.225913949	7.10E-306	7.49E-303	IFIT1	interferon induced protein with tetratricopeptide repeats 1 [Source:HGNC Symbol;Acc:HGNC:5407]
ENSG00000162494	5.031352	3.05475	92.32	72.2279	4.04305	82.274	-4.345517326	1.64E-11	2.25E-10	LRRC38	leucine rich repeat containing 38 [Source:HGNC Symbol;Acc:HGNC:27005]

ENSG00000135114	231.4422	206.705	5314.72	5438.86	219.073	5376.79	-4.616861069	0	0	OASL	2'-5'-oligoadenylate synthetase like [Source:HGNC Symbol;Acc:HGNC:8090]
ENSG00000134321	1.00627	4.073	70.9407	89.0481	2.53963	79.9944	-4.981352386	6.64E-11	8.57E-10	RSAD2	radical S-adenosyl methionine domain containing 2 [Source:HGNC Symbol;Acc:HGNC:30908]
ENSG00000119922	76.47654	68.2227	2501.39	2721.9	72.3496	2611.64	-5.173352083	2.87E-286	2.65E-283	IFIT2	interferon induced protein with tetratricopeptide repeats 2 [Source:HGNC Symbol;Acc:HGNC:5409]
ENSG00000271503	5.031352	1.01825	213.794	228.557	3.0248	221.175	-6.186908738	3.14E-22	9.10E-21	CCL5	C-C motif chemokine ligand 5 [Source:HGNC Symbol;Acc:HGNC:10632]

Table S4. The secondary metabolites in metabolomics analysis

ID	name	mz	rt	exact_mass	ppm	formula	precursor_type	Ctrl_1	Ctrl_2	Ctrl_3	GH_1	GH_2	GH_3
M165T342	Phenylpyruvic acid	165.058	341.8	164.0473	20.7447	C9H8O3	[M+H]+	5901170.871	5705267.086	4542721.04	6060252.34	6723381.821	7017977.369
M384T98	N-Acetyllactosamine	384.149	98.4	383.1428	3.32162	C14H25NO11	[M+H]+	6898267.027	7988368.199	6401782.439	10070497.2	7525303.265	11118730.1
M350T325	S-(Formylmethyl)glutathione	350.101	325	349.0944	2.50213	C12H19N3O7S	[M+H]+	761131.5814	2376723.395	2206506.889	2157651.85	661372.2302	2314752.843
M427T97	S-Glutathionyl-L-cysteine	427.094	97	426.0879	3.92418	C13H22N4O8S2	[M+H]+	4158795.605	3016876.176	3960299.443	4814551.42	3257028.477	5508661.318
M320T169	S-(Hydroxymethyl)glutathione	320.091	169.4	337.0944	12.9152	C11H19N3O7S	[M+H-H2O]+	19091743.81	85890318.93	16974424.37	87677406.3	90949219.75	76133373.68
M362T336_1	S-Lactoylglutathione	362.101	336.4	379.1049	8.9313	C13H21N3O8S	[M+H-H2O]+	4788187.698	5518306.757	5868812.984	6705631.34	5004847.504	7850489.474
M444T157	GDP	444.029	157.5	443.0243	5.57618	C10H15N5O11P2	[M+H]+	9685933.065	19263968.52	17213064.18	16716695.5	10007146.99	3919432.653
M365T133	Xanthylic acid	365.049	133.5	364.042	0.48213	C10H13N4O9P	[M+H]+	9861552.805	1509354.345	272401.393	7293465.8	5458088.338	6321205.686
M369T68	5a-Cholest-8-en-3b-ol	369.349	68.4	386.3549	3.34103	C27H46O	[M+H-H2O]+	58383702.28	51214923.06	10477342.98	11853725.3	49457956.3	45371684.94
M112T208	Cytosine	112.051	208.4	111.0433	0.21419	C4H5N3O	[M+H]+	1662631.25	1790322.278	1935988.073	3864686.33	2298730.145	2975572.431
M173T299	2-Oxoarginine	173.079	299.5	173.08	6.93321	C6H11N3O3	[M]+	4237094.038	3508746.627	5609597.578	3281196.72	2947892.092	3150662.394
M219T352	1D-1-Guanidino-1-deoxy-3-dehydro-scyllo-inositol	219.08	352.4	219.0855	25.5608	C7H13N3O5	[M]+	6579062.247	3713137.564	3597306.084	4905753.19	5367124.023	4085244.308
M110T160	1,3-Benzenediol	110.035	159.5	110.0368	16.3582	C6H6O2	[M]+	5342800.161	5829564.783	6420156.971	8366882.62	6694712.059	7500062.403
M283T68	Vaccenic acid	283.262	68.1	282.2559	2.7395	C18H34O2	[M+H]+	11856793.61	11346262.41	10633734.18	3122510.4	38417279.56	38368925.64
M369T729	Tetracosanoic acid	369.362	728.5	368.3654	27.8201	C24H48O2	[M+H]+	1498322.085	1767990.04	3174883.89	1168695.02	1416830.746	1429889.213
M279T93	13S-hydroxyoctadecadienoic acid	279.231	92.9	296.2351	11.9401	C18H32O3	[M+H-H2O]+	22190245.14	23906434.75	5128146.696	19079932.8	15884158.29	15064000.22
M101T110	Triethylamine	101.096	109.8	101.1204	4.16541	C6H15N	[M]+	1844266.329	19020988.62	5765730.497	1781465.75	9969531.307	5032256.262
M101T967	Methylmalonic acid	101.071	967	118.0266	3.10666	C4H6O4	[M+H-H2O]+	19211009.55	13281245.95	5662555.093	21581196	14847107.17	7336629.954
M104T97	gamma-Aminobutyric acid	104.071	97.1	103.0633	0.06581	C4H9NO2	[M+H]+	16618988.37	13968277.36	13000188.43	11630742.3	26246525.29	11976975.77
M109T549	m-Cresol	109.101	548.6	108.0575	6.07986	C7H8O	[M+H]+	2641887.151	2399385.299	2114524.055	2059456.95	2195459.013	2671324.017
M110T403	Hydroquinone	110.02	402.6	110.0368	3.20712	C6H6O2	[M]+	105756459	8972522.174	70786114.17	9042221.6	8200153.097	8202320.779
M110T63	Catechol	110.019	62.7	110.0368	8.84574	C6H6O2	[M]+	86145205.69	29734502.4	32994641.62	28179221.8	90003501.23	36092384.66
M111T990	Pyrrole-2-carboxylic acid	111.019	989.7	111.032	9.37481	C5H5NO2	[M]+	821104191.6	895152661.9	742286081.5	893530284	860467377.2	225985671.9
M113T153	Heptanoic acid	113.002	153.4	130.0994	11.1209	C7H14O2	[M+H-H2O]+	596788.3458	626571.7328	519478.986	757782.282	650412.2687	636945.2768
M114T465	Creatinine	114.066	464.8	113.0589	4.17303	C4H7N3O	[M+H]+	12402129.92	18054745.71	26332583.1	1581138.06	18976347.29	19003239.79
M131T548	Ketoleucine	131.086	547.7	130.063	27.7678	C6H10O3	[M+H]+	654640.96	681111.4911	646919.069	578307.2	705951.6549	383475.0623
M114T307_2	3-Methylindole	114.067	306.7	131.0735	9.94151	C9H9N	[M+H-H2O]+	30999111.52	26481062.49	50900908.72	32868576.3	31044421.86	55649241.68
M115T127	Dihydrouracil	115.05	127.4	114.0429	0.57405	C4H6N2O2	[M+H]+	24843584.39	15855745.18	15100573.58	21989250.3	20615793.54	21752998.54
M116T76	L-Proline	116.07	76.3	115.0633	3.23942	C5H9NO2	[M+H]+	24266443.21	20314697.75	17969378.58	17446081.8	17210877.29	17363483.83
M124T921	3-Hydroxybenzyl alcohol glucoside	124.086	921.1	124.0524	8.2516	C7H8O2	[M]+	81284702.55	12732911.18	57038882.43	58584135.3	52693912.01	19267718.7
M124T967	Picolinic acid	124.087	967.3	123.032	3.9339	C6H5NO2	[M+H]+	136561172.2	200892618	171084306	156246272	181299267.5	211181734.4
M126T541	Ciliatine	125.985	540.7	125.0242	10.5289	C2H8NO3P	[M+H]+	8517090.785	7015583.69	12784808.79	6347127.42	12150715.9	7574266.048
M127T242	Thymine	127.05	242.4	126.0429	0.97599	C5H6N2O2	[M+H]+	33379957.92	18303110.83	17261458.22	21190644.2	18535873.57	20312120.58
M130T153	Pipecolic acid	130.05	153.4	129.079	20.2747	C6H11NO2	[M+H]+	296146468	271257633.1	239940773	361768707	286825489.5	334110245
M132T767	(R)-Pantolactone	131.533	767.5	130.063	6.08102	C6H10O3	[M+H]+	15667184.37	51163590.27	1652138.722	5965330.27	6110234.795	85041476.74
M132T61_2	cis-4-Hydroxy-D-proline	131.973	60.8	131.0582	12.5024	C5H9NO3	[M+H]+	20528802.05	11861077.36	14383257.59	9511211.17	13780409.88	12788317.84
M132T323	L-Leucine	132.1	323.3	131.0946	14.9584	C6H13NO2	[M+H]+	25991247.81	29004406.63	21740948.96	46635105	11472996.14	26453689.7
M132T517	cis-4-Hydroxy-L-proline	132.1	517.2	131.0582	18.3855	C5H9NO3	[M+H]+	43974916.3	1981067.424	30107101.46	29313812.8	8140087.161	42108154.49
M133T85	L-Asparagine	133.061	85.4	132.0535	0.9319	C4H8N2O3	[M+H]+	30147081.01	20085390.93	22349386.86	19437800.6	17472238.74	17277082.94
M142T370	O-Phosphoethanolamine	141.957	370.2	141.0191	11.1245	C2H8NO4P	[M+H]+	23840521.17	19606108.78	32700754.38	18622834.1	17000733.38	57788853.49
M145T968	3-Hydroxymethylglutaric acid	144.982	967.5	162.0528	0.4779	C6H10O5	[M+H-H2O]+	479402309.3	540354430	493214012.1	456397815	366498085.2	346328096.4
M162T91_1	Anabasine	162.112	91.2	162.1157	20.3562	C10H14N2	[M]+	1114117615	1073233419	993975979	1195313461	1144955383	1197451209
M146T68	Spermidine	146.165	68.5	145.1579	0.13037	C7H19N3	[M+H]+	38095194.8	47907733.3	37922415.57	52163973.8	65269642.95	63628014.63
M147T928	4-Hydroxycinnamic acid	146.981	928.5	164.0473	8.76992	C9H8O3	[M+H-H2O]+	60400373.97	14689367.07	27948383.23	42500091.4	77880980.82	16146741.48
M147T77_2	L-Lysine	147.113	77	146.1055	0.00149	C6H14N2O2	[M+H]+	258655958.6	176479585.9	190343999.1	254026092	249094400	264357175.6
M148T128	L-2-Hydroxyglutaric acid	148.043	128.1	148.0372	0.60679	C5H8O5	[M]+	20026332.77	21915273.95	19087446.04	6641883.32	6331613.647	7859208.22
M149T443	3-Methyladenine	149.023	443.4	149.0701	0.00431	C6H7N5	[M]+	16078169.14	2926334.812	41182375.22	12679875.3	10601137.19	20600620.97
M167T666	Quinolinic acid	167.015	665.9	167.0219	9.65529	C7H5NO4	[M]+	6199579.241	20834331.45	27347996.41	10089305.7	5640160.422	420696.0262
M155T64_2	2,3-Butanediol	154.988	64.1	154.0122	14.056	C4H10O2S2	[M+H]+	35401689.21	30578338.82	29658161.69	24065808.3	11615892.14	13503497.19
M155T666	Gentisic acid	154.992	665.9	154.0266	14.5087	C7H6O4	[M+H]+	110779265.8	64854419.03	150883361.1	66137296.3	67397087.36	42253845.61
M173T101	4-Quinolinecarboxylic acid	172.956	100.6	173.0477	6.64936	C10H7NO2	[M]+	47386119.94	26892222.27	27014469.93	42501376.1	35528158.72	31499554.83
M157T32	Uracil 5-carboxylate	156.965	31.8	156.0171	8.2605	C5H4N2O4	[M+H]+	11401427.64	7927580.573	11444100.78	3983180.93	8146518.878	10930812.6
M159T763	4,5-Dihydroorotic acid	158.959	763.3	158.0328	13.9165	C5H6N2O4	[M+H]+	13489107.86	14160223.64	24301799.64	5045949.35	11652110.53	10003300.6
M159T145	Ascorbate	159.028	145.1	176.0321	21.5937	C6H8O6	[M+H-H2O]+	8661819.931	8196720.061	8724100.076	9744421.99	8854620.028	10785506.94
M164T289	Acetylcysteine	164.038	289.1	163.0303	2.58477	C5H9NO3S	[M+H]+	11248903.55	14282733.18	8592466.583	11453487	9098858.654	12647268.84
M183T381	Sorbitol	182.985	381.2	182.079	0.00319	C6H14O6	[M+H]+	24985513.32	71829352.06	225299212.7	11709535.6	48782832.83	123054186.2
M166T402	L-Phenylalanine	166.085	402.4	165.079	6.14826	C9H11NO2	[M+H]+	16184512.03	15856008.32	40542624.82	6572006.83	4948602.871	17157638.57

M187T107	Undecanoic acid	186.957	107	186.162	1.29982	C11H22O2	[M+H]+	35640879.52	88072894.06	65360703.94	72931510	75949344.87	72896412
M172T43	3-Dehydroshikimate	171.991	43.2	172.0372	11.7979	C7H8O5	[M]+	1397741.019	1379599.551	6363641.168	1206619.75	4684649.398	1287841.093
M183T341	Mannitol	182.985	341.4	182.079	0.4058	C6H14O6	[M+H]+	35482499.79	96785447.66	19953243.05	94660414.2	108143735.8	58742486.81
M183T577	4-Pyridoxic acid	182.982	577.3	183.0532	18.8999	C8H9NO4	[M]+	9796096.334	17573476.71	22031659.47	8708891.54	22887858.66	27498666.98
M200T647	Dodecanoic acid	200.165	646.7	200.1776	3.98939	C12H24O2	[M]+	10641402.67	9902013.886	8621382.742	9803231.4	9489625.505	10874697.14
M185T420	3,4-Dihydroxymandelic acid	184.983	420.3	184.037	13.8671	C8H8O5	[M+H]+	3958888.112	12278607.43	9692811.194	9595623.8	12680114.99	9175004.452
M204T859	L-Tryptophan	204.139	859.1	204.0899	0.59592	C11H12N2O2	[M]+	6882868.601	6368367.912	10367881.07	11505696.8	10154187.35	9759868.568
M224T306	Hydroxykynurenine	224.129	306.3	224.0797	0.97873	C10H12N2O4	[M]+	10028313.92	9780241.631	17234133.77	3704014.66	3702203.809	7164546.996
M213T112	D-Ribose 5-phosphate	213.015	111.9	230.0192	16.5904	C5H11O8P	[M+H-H2O]+	40127767.66	45809333.31	43437966.2	50137807.3	51824072.79	53628512.12
M224T777	Methyl jasmonate	223.989	777	224.1412	3.17078	C13H20O3	[M]+	119806929.3	245934234.3	284751937.9	214929311	107291049.7	135015836.6
M226T873	Porphobilinogen	226.18	873.2	226.0954	0.80006	C10H14N2O4	[M]+	17561093.23	28044667.84	42083338.02	51673129.1	56473904.84	67441419.06
M228T776	Deoxyuridine	228.196	776.2	228.0746	0.01897	C9H12N2O5	[M]+	150478456.3	140706976.2	143654380.6	145175263	137048755.7	152108856.3
M242T357	Equol	242.1	357	242.0943	19.7517	C15H14O3	[M]+	81161109.19	90522484.07	75185836.86	68178911.6	60164211.35	72759069.59
M248T146	Pyridoxal 5'-phosphate	248.032	145.7	247.0246	0.07747	C8H10NO6P	[M+H]+	9088645.219	9036210.48	8325538.481	8393779.39	8000164.658	9268269.88
M280T939	Linoleic acid	280.263	938.7	280.2402	1.32804	C18H32O2	[M]+	12648636.19	8656113.451	15392828.21	13975761.2	3027810.116	4769759.344
M282T128	1-Methyladenosine	282.119	127.6	281.1124	3.63614	C11H15N5O4	[M+H]+	17816567.2	27576146.72	32029580.4	65690563	42627788.2	63057589.84
M282T811	Oleic acid	282.279	811.5	282.2559	0.099	C18H34O2	[M]+	2811876.489	8321846.407	41055978.65	39297308.5	34078650.45	4286217.873
M284T442	Stearic acid	284.186	442.2	284.2715	4.07591	C18H36O2	[M]+	15944513.64	12303008.59	16558982.27	13023292.3	32413115.65	27557795.53
M288T750	Dehydroepiandrosterone	288.291	750.4	288.2089	0.71888	C19H28O2	[M]+	25672926.41	69444824.65	49145051.18	35568873.7	26483739.21	14652459.54
M282T761	(6Z)-Octadecenoic acid	282.279	761.2	282.2559	2.57582	C18H34O2	[M]+	156425858	217487234.5	245834331.6	226333553	223309837.4	78352729.98
M286T780	Fisetin	286.273	779.9	286.048	4.98714	C15H10O6	[M]+	8915464.59	15568732.34	11897708.74	11183083.6	7210413.815	9048614.166
M308T138	Glutathione	308.091	137.5	307.0838	0.34205	C10H17N3O6S	[M+H]+	14182148478	12223543003	14457954281	9890156361	10652409697	10394674344
M336T937	12-Keto-tetrahydro-leukotriene B4	336.311	936.7	336.2301	2.33274	C20H32O4	[M]+	10664665.36	11251052.41	9083100.53	10247693	7592082.486	9727819.717
M335T101	Nicotinamide ribotide	335.064	101.1	334.0566	1.12218	C11H15N2O8P	[M+H]+	15730680.3	8315397.992	9133505.221	4909514.08	4731633.701	5127518.021
M355T967	Prostaglandin F2a	355.281	967.4	354.2406	4.65043	C20H34O5	[M+H]+	15183648.89	13670635.79	18070107.09	17784639	9112984.045	4480374.255
M338T974_1	Erucic acid	338.341	974	338.3185	1.38999	C22H42O2	[M]+	173781476.8	866580702.6	59842550.51	711267437	1789614733	1766635373
M341T937	Fructose 1,6-bisphosphate	341.265	936.8	339.996	5.18006	C6H14O12P2	[M+H]+	12218476.77	17316775.19	9726210.962	13673487.8	8166475.201	7660658.513
M361T875	Aldosterone	361.22	875.3	360.1937	7.80843	C21H28O5	[M+H]+	9106076.651	14757319.93	11630969.14	4022612.66	16839575.62	11879157.15
M364T151	GMP	364.073	151.4	363.058	22.2483	C10H14N5O8P	[M+H]+	11564564.06	12397309.93	13668127.29	51682717.8	17190128.36	4583206.358
M385T937	Vitamin D3	385.293	936.7	384.3392	2.34767	C27H44O	[M+H]+	7382583.466	4379654.245	9864868.272	12942873.6	2448760.567	2626034.547
M369T972	Cholesterol	369.36	972.3	386.3549	22.4713	C27H46O	[M+H-H2O]+	12560255.8	87699096.69	84356817.15	39665186.2	5001066.612	29385817.48
M391T916_1	S-Hexyl-glutathione	391.288	915.5	391.1777	10.3862	C16H29N3O6S	[M]+	8653168.314	6595422.855	45152446.54	84214977.6	158344791.3	175807978.8
M385T318_2	7-Dehydrocholesterol	385.339	317.6	384.3392	19.9201	C27H44O	[M+H]+	9699243.878	4277042.212	6355468.612	4636068.89	3288747.266	5219756.427
M428T159	ADP	428.03	158.9	427.0294	16.0643	C10H15N5O10P2	[M+H]+	4410335.797	5157823.22	4481385.435	5905787.72	7216645.056	17618162.43
M430T539_2	alpha-Tocopherol	430.241	538.8	430.3811	3.22335	C29H50O2	[M]+	22555872.75	11902214.78	16076697.19	11801326	16690996.4	17431302.53
M415T810	Sodium deoxycholate	415.213	810	414.2746	5.59118	C24H39O4. Na	[M+H]+	5380215.016	5257419.806	5196309.175	4805422.66	3155076.922	2862623.299
M432T799_2	Isovitexin	432.246	799.1	432.1056	16.1697	C21H20O10	[M]+	8994536.564	33687308.81	5442349.96	19696991.4	11218038.31	24006284.93
M610T959	Rutin	610.179	959	610.153	8.15499	C27H30O16	[M-H2O+NH4]+	24600108.23	26827920.36	26510244.05	22361340.4	88072254.72	109110232.5
M123T132	Niacinamide	123.055	132	122.048	0	C6H6N2O	[M+H]+	1076514456	1083095573	1025098516	1620609098	1498682938	1621605367
M130T419	Pyrrolidonecarboxylic acid	130.049	419.3	129.0426	5.96698	C5H7NO3	[M+H]+	19461049.53	28454035.49	5114866.032	28433139.4	4810253.366	18981192.13
M134T100	Iminodiacetic acid	134.045	100.2	133.0375	0.74602	C4H7NO4	[M+H]+	51854561.38	204068014.7	54104934.21	47838587.9	40063485.84	45220730.32
M137T90	1-Methylnicotinamide	137.071	90.1	137.0715	0.72955	C7H9N2O	[M+H]+	78594138.05	78369762.72	75097544.89	77757728.7	81801866.05	83892091.75
M152T277	Guanine	152.057	277.3	151.0494	2.63059	C5H5N5O	[M+H]+	37100942.48	35752577.25	38901995.05	44071950.8	42796708.75	45567758.61
M152T462	Loratadine	152.144	462.4	151.1361	2.62909	C10H17N	[M+H]+	1192914.92	409724.6079	976887.0385	844820.972	250730.1771	201384.0926
M153T318	Xanthine	153.041	318.4	152.0334	1.96026	C5H4N4O2	[M+H]+	26119850.9	10366347.93	11610644.79	17092696.3	22973510.62	22459798.47
M163T142	Nicotine	163.123	141.8	162.1157	0.61303	C10H14N2	[M+H]+	54954885.94	44190505.67	85201589.32	53104842.8	49444390.2	40725515.01
M174T498	N-Acetylleucine	174.113	498.5	173.1052	0	C8H15NO3	[M+H]+	1892103.115	3994005.259	4023358.775	4004273.78	3859843.888	1680978.982
M184T101	Phosphorylcholine	184.073	100.6	184.0739	0.82723	C5H15NO4P	[M+H]+	171304965.7	39428901.74	135348228.3	35296855.9	35817527.84	140798874.7
M204T150	N-Acetyl-D-glucosamine	204.086	149.6	221.0899	18.7862	C8H15NO6	[M+H-H2O]+	94409777.9	128366339.9	119787972.6	71835132.9	44678669.37	88694849.6
M224T668	Cerulenin	224.126	667.9	223.1208	8.01557	C12H17NO3	[M+H]+	32521863.68	20653233.09	23786022.62	7308254.31	7532794.031	33857452.74
M219T111	gamma-Glutamylalanine	219.097	110.7		1.36926	C7H11N2O5R	[M+H]+	3933974.875	11317296.44	10174484.43	13692729.8	7327932.91	7259546.749
M245T171	Uridine	245.077	170.8	244.0695	1.12618	C9H12N2O6	[M+H]+	231505300	208664652.5	232326918.8	260984688	257471793.4	275634810.6
M268T237_2	Adenosine	268.104	236.6	267.0968	0.8355	C10H13N5O4	[M+H]+	211524074.2	278228424.8	201145668.5	198104476	216866225.4	198895600.5
M279T277	Alpha-dimorphecolic acid	279.236	276.8	296.2351	13.9667	C18H32O3	[M+H-H2O]+	10238330.42	8272516.261	7750259.377	4655602.26	15883174.32	4611044.596
M282T955	Oleamide	282.279	955.2	281.2719	0.08502	C18H35NO	[M+H]+	1474382.603	3542317.052	21801006.49	14203562	17045460.33	13575572.83
M348T122_1	AMP	348.069	122.3	347.0631	3.37864	C10H14N5O7P	[M+H]+	3539208285	4030584955	3311344251	4068717116	3430620670	3496000754
M369T643	Lathosterol	369.361	642.8	386.3549	24.9079	C27H46O	[M+H-H2O]+	1567056.825	1916125.748	1984015.339	385395.136	1486620.72	1500263.304
M399T958_2	Calcitriol	399.313	958	416.329	21.2014	C27H44O3	[M+H-H2O]+	20984778.22	24093945.29	27028605.81	28423767.6	11326838.78	9052356.035
M401T932_1	20a,22b-Dihydroxycholesterol	401.341	932.1	418.3447	2.24248	C27H46O3	[M+H-H2O]+	76390.8654	75852.54872	3932865.649	18507818.9	36865587.36	27088253.62
M474T378_2	Folinic acid	474.169	378.2	473.1659	7.9634	C20H23N7O7	[M+H]+	10026967.54	5230714.95	6220513.402	13202252.5	16096917.13	17000185.33

M744T117_2	NADP	744.091	117	744.0833	9.67624	C21H29N7O17P3	[M]+	22207931.19	25606498.48	22226602.51	38629465.2	27826222.57	41714230.8
M786T401_2	FAD	786.159	400.8	785.1571	6.96551	C27H33N9O15P2	[M+H]+	8384669.722	9899783.06	8156162.275	14560198.2	12901822.14	12108970.71
M194T356	3,4-Methylenedioxyamphetamine	194.116	356.3	193.1103	0.20119	C11H15NO2	[M+H]+	39121218.45	9442798.2	13130118.8	8876863.98	17696400.58	9093742.267
M180T689	Rimantadine	180.175	689.1	179.1674	0.41999	C12H21N	[M+H]+	1149793.621	1990813.968	1429922.536	1285751.18	1214877.649	2011031.922
M192T110	Isocitric acid	192.033	110.3	192.027	0.36392	C6H8O7	[M]+	84553854.18	100100914.5	87249353.42	172171086	114341645.4	128496709.1
M122T93	L-Cysteine	122.027	92.6	121.0197	0.62281	C3H7NO2S	[M+H]+	7120379.6	7469051.055	7595524.537	11244468.8	7031930.299	10478743.77
M126T72	Taurine	125.986	72.5	125.0147	4.01027	C2H7NO3S	[M+H]+	354267834.3	209534796.2	294502678.9	275348074	259542400.1	273054141.1
M132T561	Leucine	132.104	560.5	131.0946	15.3213	C6H13NO2	[M+H]+	37784235.5	4606186.62	4377976.234	1717541.47	4060571.112	2055713.528
M137T277_1	Hypoxanthine	137.046	277	136.0385	0.65664	C5H4N4O	[M+H]+	2625014214	2109937689	2385282096	1839188185	2710832967	2486261805
M176T135	N-Acetyl-L-aspartic acid	176.056	134.6	175.0481	0.19483	C6H9NO5	[M+H]+	193628583.2	196849306.7	218406585.5	249265699	214766545	265116497.2
M220T218	5-Hydroxy-L-tryptophan	220.082	218.1	220.0848	13.1769	C11H12N2O3	[M]+	7938486.245	7756045.774	7273303.642	9093374.7	8280838.466	9066507.516
M222T98	N-Acetylmannosamine	222.098	97.7	221.0899	1.90907	C8H15NO6	[M+H]+	15653315.64	12673752.95	12752489	13069768.6	15667714.37	16581589.1
M284T924	Octadecanamide	284.294	924.5	283.2875	2.72957	C18H37NO	[M+H]+	25790255.67	41570587.8	58411434.39	47871930.7	41422476.33	24623895.51
M104T788	Choline	104.108	787.8	104.1075	0	C5H14NO	[M]+	3364005.986	18914481.05	2753013.021	10731497.8	2939797.263	12034209.46
M123T190	4-Hydroxybenzaldehyde	123.044	190.1	122.0368	0.61767	C7H6O2	[M+H]+	68719655.31	52207928.94	51936670.77	61778554.2	59575289.56	64769047.7
M124T760	L-Histidinol	124.086	760.3	141.0902	26.0626	C6H11N3O	[M+H-H2O]+	13315825.7	17214716.08	14026817.44	15723305.1	21575517.99	4374484.23
M129T236	Dihydrothymine	129.066	235.8	128.0586	1.73554	C5H8N2O2	[M+H]+	23640957.4	14166217.72	13103847.31	18259954.6	17496909.57	18881685.19
M149T340	3,4-Dihydro-2H-1-benzopyran-2-one	149.06	339.8	148.0524	1.97574	C9H8O2	[M+H]+	56752576.17	42939687.95	43414337.46	48619530.3	50116594.95	52512604.25
M159T399	Serotonin	159.092	398.6	176.095	27.2421	C10H12N2O	[M+H-H2O]+	34305253.53	26718664.51	24642302.85	30518045	29267241.48	32503268.53
M182T190_2	L-Tyrosine	182.081	190.1	181.0739	0.13181	C9H11NO3	[M+H]+	4746228303	3572255534	3556548142	4221575798	4197100868	4428990652
M189T300_2	Glycylleucine	189.124	299.7	188.1161	1.18441	C8H16N2O3	[M+H]+	20924873.56	16050735.01	20091434.61	7771744.9	18716764.86	15428021.94
M218T276	Propionylcarnitine	218.139	275.9	217.1314	1.44821	C10H19NO4	[M+H]+	745282092.8	1175206322	1212966241	818317126	1086334217	1052358126
M318T786	Phytosphingosine	318.299	786.1	317.293	2.73361	C18H39NO3	[M+H]+	6617119.619	6392112.835	4759922.8	1460750.21	1026976.4	1494042.173
M144T398	2-Naphthylamine	144.081	398.3	143.0735	0.16657	C10H9N	[M+H]+	3603869.123	2992559.062	3126354.202	3891180.69	3242425.662	3445536.194
M166T134	L-Methionine S-oxide	166.053	133.7	165.046	0.74675	C5H11NO3S	[M+H]+	16385674.84	31654318.87	40535536.76	76387390.6	91422576.89	106630137.5
M165T190	3,4-Dihydroxyphenylpropanoate	165.054	190.1	182.0579	23.2287	C9H10O4	[M+H-H2O]+	807085105.2	618045109.4	606171816.6	732997038	717807113.5	773898829.8
M250T743	Alprenolol	250.177	743.2	249.172879	13.0107	C15H23NO2	[M+H]+	142036765.9	152805924	130473526.2	122718148	130208364.7	139300915.8
M259T106	(5-L-Glutamyl)-L-glutamate	259.092	106.1	276.0958	12.4821	C10H16N2O7	[M+H-H2O]+	4290773.194	3910212.474	4170827.205	4572193.71	3615985.7	3321787.598
M298T926	Tridemorph	298.31	925.6	297.3032	1.93088	C19H39NO	[M+H]+	715118.4591	1131870.179	6719559.263	8825811.16	15323431.32	14064145.09
M101T93	Methyl isobutyl ketone	101.096	92.6	100.0888	0.08501	C6H12O	[M+H]+	28536293.96	7261247.095	7206913.461	15928242.3	6776477.189	28548606.18
M114T199	1-Pyrroline-5-carboxylic acid	114.055	199.1	113.0477	0.66635	C5H7NO2	[M+H]+	8979769.386	9999652.998	8001730.011	8191997.97	9399949.714	9665264.511
M114T623	1-Pyrroline-2-carboxylic acid	114.067	623.1	113.0477	8.16496	C5H7NO2	[M+H]+	55117722.97	55541223.03	38335500.87	35561441.5	22200403.03	37444548.61
M114T192	Beta-Guanidinopropionic acid	114.065	191.7	131.0695	29.2288	C4H9N3O2	[M+H-H2O]+	75787919.86	75894506.18	54584050.39	63097117.8	42748690.23	64716789.77
M114T418	Epsilon-caprolactam	114.092	418.2	113.0841	1.36806	C6H11NO	[M+H]+	27262991.27	26102561.24	24455877.28	15872726.6	24747631.66	33178568.08
M119T106	Aminomalonic acid	119.016	105.6	119.0219	0.86491	C3H5NO4	[M]+	4625542.94	3605218.975	4168013.2	5060055.4	4600631	6411554.311
M119T190	2-Methylserine	119.049	190.2	119.0582	1.48285	C4H9NO3	[M]+	40413010.08	30777659.35	30148943.51	35369178.3	35737049.5	36462267.14
M127T230	Triacetate lactone	127.039	229.8	126.0317	3.7469	C6H6O3	[M+H]+	15821022.41	4078113.086	13618633.8	16533313.5	13627723.11	12629952.75
M130T72_3	Mesaconate	130.017	72.4	130.0266	0.05183	C5H6O4	[M]+	32967582.61	27339471.46	32573510.36	28499584.4	35086317.39	35547237.53
M132T744	N-Carbamoylputrescine	132.1	744	131.1059	17.5531	C5H13N3O	[M+H]+	34098952.52	28380290.7	14339679	13984128.7	37575932.77	49021088.01
M132T494	N,N-Diethylglycine	132.1	493.7	131.0946	12.6873	C6H13NO2	[M+H]+	35876901.82	19541351.29	17702640.55	38041313.5	7190629.373	13923331.13
M132T476	D-allo-Isoleucine	132.1	476.2	131.0946	12.5281	C6H13NO2	[M+H]+	24076699.27	43750237.43	21314806.27	17401980.1	20332671.01	29934342.37
M135T738	Chavicol	135.081	738.1	134.0732	0.06617	C9H10O	[M+H]+	3231713.751	2382856.613	3242565.13	3109210.59	3286694.805	3102675.664
M136T237	4-Hydroxyphenylacetaldehyde	136.062	237	136.0524	2.43729	C8H8O2	[M]+	8590970.181	9660831.274	7801381.7	8788329.88	8191349.533	8162547.383
M137T145	p-Aminobenzoic acid	137.046	144.6	137.0477	0.25609	C7H7NO2	[M]+	4132587283	4252685397	4031731264	5817366232	5143385198	5635644289
M140T212	Acetylphosphate	139.981	211.7	139.9875	7.57922	C2H5O5P	[M]+	63225052.67	30720645.04	68557373.6	26366476.8	33036156.56	35004876.14
M146T260	4-Guanidinobutanoic acid	146.082	260.4	145.0851	1.79494	C5H11N3O2	[M+H]+	5027085.645	4955941.436	4619743.108	5642854.94	5323521.46	5785360.58
M147T190	Coumarin	147.044	190.1	146.0368	0.47719	C9H6O2	[M+H]+	79512153.21	60765635.55	60823173.86	72245101.5	70746789.9	74265027.31
M151T72	(S)-4-Hydroxymandelate	151.035	72.4	168.0423	2.8735	C8H8O4	[M+H-H2O]+	491190166.1	407476017.9	491657722	427739892	525313145.2	532644567
M157T765	Phosphoglycolic acid	156.993	765.1	155.9824	17.9881	C2H5O6P	[M+H]+	125500216.9	92229370.53	15572817.08	105665213	92004946.44	47545315.86
M162T282	L-Rhamnono-1,4-lactone	162.059	281.6	162.0528	2.24619	C6H10O5	[M]+	225731482.2	135546425.3	113394966.5	136313153	111071944.8	140263360.8
M162T122	N-methyl-L-glutamic Acid	162.076	121.7	161.0688	0.0151	C6H11NO4	[M+H]+	44448652.67	36253853.34	37768249.02	47127670.8	48677185.04	48234272.53
M163T717	2-Deoxystreptamine	163.112	717.1	162.1004	0.00703	C6H14N2O3	[M+H]+	3684557.365	3218627.922	2981895.724	3064635.86	3109008.857	4197453.699
M164T191	Pterin	164.057	191.5	163.0494	0.73075	C6H5N5O	[M+H]+	12905306.86	10998586.05	11941340.14	7938977.9	8346136.404	9877415.999
M166T324	7-Methylguanine	166.073	324	165.0651	0.00555	C6H7N5O	[M+H]+	614533.2777	645349.7647	1524516.289	3428514.09	934112.1441	3330659.225
M167T819	3-(2-Hydroxyphenyl)propanoic acid	167.07	819.5	166.063	0.00408	C9H10O3	[M+H]+	5458402.944	4637070.351	5654676.222	5496949.4	4676388.668	4776586.758
M168T128	3-Methoxyanthranilate	168.066	127.6	167.0582	0.46863	C8H9NO3	[M+H]+	44481434.1	32379136.39	37952432.19	35629552.1	36869039.51	44314981.01
M170T485	8-Amino-7-oxononanoate	170.118	484.8	187.1208	26.0643	C9H17NO3	[M+H-H2O]+	13738723.06	13156134	5771079.665	5077842.99	7694722.646	9619209.038
M172T516	Gabapentin	172.133	515.7	171.1259	0.04454	C9H17NO2	[M+H]+	63638578.24	42494187.4	34422925.11	19938409.6	37149608.27	43950644.28
M173T108	Iminoarginine	173.092	108.4	172.096	0.01457	C6H12N4O2	[M+H]+	14727526.2	13112684.11	14237689.87	12142275.7	4004639.324	14576301.48
M176T383	D-Alanyl-D-serine	176.074	382.9	176.0797	1.62413	C6H12N2O4	[M]+	167215240.7	118709676.3	96663185.48	115489042	94857972.78	130272529.7

M183T420	Se-Methylselenocysteine	182.983	420.2	182.9799	14.5585	C4H9NO2Se	[M]+	11673206.23	39862698.42	19468465.39	56670197.9	19238179.19	11939272.35
M193T168	5,6-Dihydroxy-3-methyl-2-oxo-1,2,5,6-tetrahydroquinoline	193.068	168.3	193.0739	1.15882	C10H11NO3	[M]+	647419848.6	612279560.8	615396032.1	628228679	738457588.5	839640895.2
M200T312	gamma-Glutamyl-beta-aminopropiononitrile	200.092	311.6	199.0957	0.54943	C8H13N3O3	[M+H]+	10563516.75	9869084.6	9147440.409	9112802.02	8657319.769	9099522.938
M219T114	D-Lysopine	219.134	114	218.1267	0.05738	C9H18N2O4	[M+H]+	6087281.092	4812465.012	5071451.628	7802139.93	8016184.756	8222637.952
M202T400	Thiabendazole	202.045	400.3	201.0361	0.24572	C10H7N3S	[M+H]+	2015015580	1943269464	1850490693	1776605181	1817038429	1870682844
M217T354	N-a-Acetylcitrulline	217.106	354.2	217.1063	3.22424	C8H15N3O4	[M]+	6887484.887	5463763.382	4822870.45	4408737.73	7321403.501	5085817.936
M219T692	Capsidiol	219.174	692.1	236.1776	4.2E-05	C15H24O2	[M+H-H2O]+	2485535.026	2301596.891	2101364.695	533579.326	4519419.458	4120841.883
M220T357_2	1D-1-Guanidino-3-amino-1,3-dideoxy-scyllo-inositol	220.117	357	220.1172	0.4543	C7H16N4O4	[M]+	1653684679	1549138947	1384898515	1263438727	1285199181	1338393314
M239T282	4a-Carbinolamine tetrahydrobiopterin	239.103	281.6	239.1018	0.12804	C9H13N5O3	[M]+	9245391.689	5532103.841	7771857.991	2347936.19	6595600.834	4897019.497
M232T380_2	Butyryl-L-carnitine	232.155	379.5	231.1470582	0.00883	C11H21NO4	[M+H]+	811015193	986678658.6	919738345.6	1148525137	1231519190	1336708673
M241T479	N(alpha)-gamma-L-Glutamylhistamine	241.109	478.5	240.1222	1.66732	C10H16N4O3	[M+H]+	1253406.418	1216448.063	1197161.503	1596040.95	1963543.006	1982678.737
M291T574	Aurin	291.101	574.3	290.0943	2.66574	C19H14O3	[M+H]+	4212005.425	3596757.134	3036915.269	7942088.78	7294628.584	9194775.26
M296T381	Sumatriptan	296.135	381.3	295.1354	13.0747	C14H21N3O2S	[M+H]+	1395674.449	2408775.823	1778245.36	3786572.23	3608876.629	3834721.188
M295T738	13(S)-HOT	295.226	738.1	294.2195	4.32212	C18H30O3	[M+H]+	11732120.04	9684298.739	16159619.49	13508977.4	14516522.3	13801679.94
M302T93	N-Acetyl-alpha-D-glucosamine 1-phosphate	302.063	92.6	301.0563	0.52229	C8H16NO9P	[M+H]+	14543192.47	11035134.36	10555535.63	17587404.9	17181087.88	16920347.01
M322T185_2	gamma-L-Glutamyl-L-cysteinyl-beta-alanine	322.077	185.4	321.0995	0.02697	C11H19N3O6S	[M+H]+	916803661.3	882109595.4	884328315.3	1047519016	1073040180	1137098476
M405T132_1	UDP	405.007	131.9	404.0022	3.82479	C9H14N2O12P2	[M+H]+	48871236.21	69338255.55	56548526.17	72426135.4	311034650.4	321120448.6
M482T805	Antibiotic JI-20A	482.318	804.8	481.2748	13.0897	C19H39N5O9	[M+H]+	1142504.651	1992772.469	4577832.622	14154483.8	10431174.39	14190910.06
M608T261_2	UDP-N-acetyl-D-mannosamine	608.096	260.7	607.0816	12.0848	C17H27N3O17P2	[M+H]+	13879032.86	45113455.21	173457624.8	136866743	150921244.6	52459180.21
M161T303	D-Galactose	161.044	303.4	180.0634	4.40871	C6H12O6	[M-H2O-H]-	2343418.248	14725890.03	14098998.47	10621927.5	3896037.66	10593069.22
M668T129_2	Dephospho-CoA	668.119	129	687.1489	17.6762	C21H35N7O13P2S	[M-H2O-H]-	264766.7541	1030827.567	231045.9746	1529763.4	311208.524	247935.6379
M611T78_2	Oxidized glutathione	611.152	77.7	612.152	12.2328	C20H32N6O12S2	[M-H]-	35248882.42	78427249.24	41405916.06	55406282.6	33048777.49	49154347.64
M161T674	D-Aldose	161.047	673.8	180.0634	14.2196	C6H12O6	[M-H2O-H]-	715699.9815	1873831.426	551882.0253	1145214.69	1173494.939	715699.5577
M161T530	D-Glucopyranoside	161.044	529.7	180.0634	6.27154	C6H12O6	[M-H2O-H]-	1054962.726	5801859.561	1875348.711	6942123.82	1780243.518	7553398.595
M108T87	Hypotaurine	108.013	86.7	109.0197	1.62944	C2H7NO2S	[M-H]-	13726119.49	22073299.58	22207779.16	12607818.1	12478026.47	12311619.29
M128T87	Pyroglutamic acid	128.035	87.3	129.0426	0.59359	C5H7NO3	[M-H]-	31606980.62	35447420.31	47163958.89	42211698.2	45939835.68	36047117.64
M112T129_1	Uracil	112.024	129.1	112.0273	10.7737	C4H4N2O2	[M]-	294658.2801	5095940.582	612786.8284	7745063.97	338812.6041	7144582.926
M113T403	2-Heptanone	112.984	402.9	114.1045	6.01405	C7H14O	[M-H]-	2964177.254	1591300.87	1400308.916	13719600.7	4757586.364	5846643.914
M115T870	Caproic acid	114.934	869.7	116.0837	7.44399	C6H12O2	[M-H]-	43581881.06	123854469	128392840.1	131152947	145604185.8	71859962.99
M115T79	Fumaric acid	115.004	78.6	116.011	1.53038	C4H4O4	[M-H]-	35137217.44	71648670.69	60120369.13	56994481.3	62775768.24	64393577.73
M115T133	Maleic acid	115.004	133.1	116.011	0.66085	C4H4O4	[M-H]-	3315393.138	1141184.115	4046425.142	3403231.77	5523790.682	5247289.828
M116T947	trans-1,2-Cyclohexanediol	115.922	947.2	116.0837	15.3907	C6H12O2	[M]-	34592169.78	9968516.137	18873247.53	17400890	7279027.193	13743646.29
M116T167	5-Aminopentanoic acid	116.071	167.2	117.079	5.65487	C5H11NO2	[M-H]-	16356124.04	4886079.094	6386038.538	7241772.12	26355186.42	1762910.744
M116T250	L-Norvaline	116.071	250.1	117.079	1.90965	C5H11NO2	[M-H]-	4049964.32	2875542.789	318013.206	6894766.26	3900348.797	3621734.369
M116T109	L-Valine	116.072	109.4	117.079	0.20677	C5H11NO2	[M-H]-	28493483.28	36448182.95	39196025.33	43355704.5	45409793.05	44768910.75
M116T198	Betaine	116.071	198.1	117.079	6.89234	C5H11NO2	[M-H]-	2456015.416	7052923.776	9570527.414	4572868.38	427838.7782	5463497.048
M117T755	Guanidoacetic acid	116.93	754.7	117.0538	23.1821	C3H7N3O2	[M]-	10473303.16	18042399.16	18435749.68	128609238	16527548.51	136926966.9
M117T113	Succinic acid	117.019	112.7	118.0266	0.20509	C4H6O4	[M-H]-	3084130.592	5098069.97	3006437.67	3311703.43	3081833.022	3433277.693
M124T62	1-Naphthylamine	123.902	61.8	143.0735	7.87828	C10H9N	[M-H2O-H]-	2915835.204	3864918.922	4897723.17	4833378.24	4647008.955	4324132.659
M130T304	L-Isoleucine	130.086	304.2	131.0946	0.14582	C6H13NO2	[M-H]-	9479199.721	33331500.71	19777398.79	17076952.3	14248024.78	7451317.194
M130T96	Creatine	130.062	96.4	131.0695	0.18453	C4H9N3O2	[M-H]-	24799517.92	49412926.32	41121386.74	40556736.1	32849384.77	41314457.84
M131T302	Glutaric acid	130.993	301.7	132.0423	1.6664	C5H8O4	[M-H]-	634774.9083	2925614.486	1129792.32	6971001.11	1062759.481	5085905.885
M131T85	D-Asparagine	131.046	85.1	132.0535	0.57995	C4H8N2O3	[M-H]-	13410080.55	20425339.44	22532350.4	22229968.6	23838417.47	23142132.25
M135T60	Phenylacetic acid	134.865	60	136.0524	12.429	C8H8O2	[M-H]-	5730204.625	7532058.039	9641399.357	8541524.88	8432225.709	8431941.641
M136T887_2	Phenyl acetate	135.971	887.5	136.0524	10.6621	C8H8O2	[M]-	11989498.36	50145771.11	27320808.05	43930499.5	21035110.32	6306736.354
M159T231	Oxoadipic acid	158.978	231.4	160.0372	4.67519	C6H8O5	[M-H]-	52503109.29	23468403.16	113166157	24952646.9	108527028.5	30180232.78
M144T119	4-Acetamidobutanoic acid	144.067	118.9	145.0739	0.52753	C6H11NO3	[M-H]-	1254006.928	1963663.32	4317912.723	4437087.9	2453488.148	2204331.022
M146T78	L-Glutamic acid	146.046	77.6	147.0532	1.88981	C5H9NO4	[M-H]-	1082625089	1711702517	1724486814	1674955524	1757971612	1778445935
M154T98	L-Histidine	154.063	97.6	155.0695	1.79148	C6H9N3O2	[M-H]-	23455935.25	36822932.64	31520159.35	47404253.2	36057492.41	49312074.91
M180T799	D-Mannose	179.989	798.9	180.0634	8.32597	C6H12O6	[M]-	2070901.939	12117633.41	6331539.239	21296044.4	1184814.246	10040029.76
M179T85_2	myo-Inositol	179.056	85.1	180.0634	0.98293	C6H12O6	[M-H]-	16887781.24	21559005.57	29060540.6	25546667.2	17535890.24	16971185.51
M180T826	Allose	179.989	825.5	180.0634	8.15641	C6H12O6	[M]-	11363500.16	3728968.662	21973259.13	17724180	10395548.92	15164587.98
M180T953	Paraxanthine	179.989	952.8	180.0647	5.73575	C7H8N4O2	[M]-	1024143572	664791592.4	582192652.2	783252499	738043805.8	931324478.5
M165T302_1	D-Phenylalanine	165.02	302.2	165.079	12.3861	C9H11NO2	[M]-	5374257.44	8851708.992	32565547.75	15848215.5	8415092.815	8353582.188
M165T963	Phthalic acid	165.041	963.5	166.0266	5.95711	C8H6O4	[M-H]-	8833844.885	17982530.62	24365838.31	6719100.85	26447209.93	7385849.051
M188T80	N-Acetylglutamic acid	188.056	79.5	189.0637	1.19113	C7H11NO5	[M-H]-	126320237.2	287828890.5	222299235	327662200	219497027.4	319365524.3
M191T79	Citric acid	191.02	79.2	192.027	1.44487	C6H8O7	[M-H]-	75655732.78	155136326.2	156673663	87368471.9	75051642.96	128573920.1
M173T101	L-Arginine	173.105	101	174.1117	0.43904	C6H14N4O2	[M-H]-	59512560.14	85796518.57	94095728.97	112727789	117122966.5	125368196.9
M175T35	Guanidinosuccinic acid	174.958	34.9	175.0593	19.8588	C5H9N3O4	[M]-	10380631.64	4230849.87	10030493.95	7084782.39	9220974.147	14061433.7
M181T92	Acetylcholine chloride	180.973	91.9	181.087	7.51997	C7H16NO2. Cl	[M]-	259121687.5	479440473.1	472245471.2	462754998	491539432.4	500675915.8

M187T574	10-Hydroxydecanoic acid	187.134	573.9	188.1412	0.66263	C10H20O3	[M-H]-	733848.1195	1708679.113	1531159.467	3970126.68	3741850.622	2558162.314
M187T305	Azelaic acid	187.098	304.6	188.1049	1.47516	C9H16O4	[M-H]-	1629209.058	12521102.05	20000915.92	25544974.9	9777589.329	10926842.61
M206T372	N-Acetyl-L-phenylalanine	206.082	371.9	207.0895	0.11646	C11H13NO3	[M-H]-	1276068.252	1895598.83	2047753.517	1897161.5	2310776.71	2206946.538
M191T80	Quinate	191.057	79.9	192.0634	1.968	C7H12O6	[M-H]-	3898064.457	39640090.3	5430871.794	3762707.27	4169135.304	3919029.597
M218T157	Pantothenic acid	218.104	157.1	219.1107	1.72395	C9H17NO5	[M-H]-	594470963	467030334.6	976280712.3	432319372	848761814.8	412130372.4
M223T842	Thymidine	223.028	842	242.0903	1.82765	C10H14N2O5	[M-H2O-H]-	13292113.17	24267701.95	23502274.07	18240843	27305294.3	11601511.61
M249T91	gamma-Glutamylcysteine	248.96	91.3	250.0623	0.03486	C8H14N2O5S	[M-H]-	675122217.6	1232282742	1096885346	1228939870	1289676640	1326085197
M255T864	Palmitic acid	255.233	864.2	256.2402	1.86496	C16H32O2	[M-H]-	717060820.9	284717097.7	446021734.9	552464239	460778334.9	367442369
M282T269	Guanosine	282.085	269.3	283.0917	0.97843	C10H13N5O5	[M-H]-	8384415.062	12984019.25	12436571.88	17665931	16323749.46	18650887.78
M277T923	Alpha-Linolenic acid	277.217	922.8	278.2246	2.61168	C18H30O2	[M-H]-	6483154.403	6843274.433	3781364.336	6594844.15	1164014.714	7421695.65
M277T930	Gamma-Linolenic acid	277.217	930	278.2246	0.93609	C18H30O2	[M-H]-	1448793.549	5625103.645	5545978.12	5971435.39	4044574.359	1611187.262
M306T93	(-)-Epigallocatechin	306.075	92.7	306.074	4.24732	C15H14O7	[M]-	238331075.7	335656435.4	384754637.6	274596875	294651538.9	269773020.6
M297T895	5'-Methylthioadenosine	297.246	894.8	297.0896	7.13994	C11H15N5O3S	[M]-	10501833.49	14355761.88	15668155.11	30017357.3	14596410.19	12734562.62
M297T854	Nonadecanoic acid	297.238	854.2	298.2872	18.9305	C19H38O2	[M-H]-	15151770.76	21208255.88	32495190.95	11071109	10656435.92	30521867.78
M323T87_2	UMP	323.029	86.8	324.0359	0.0743	C9H13N2O9P	[M-H]-	116607368.3	161868349.1	219886508.2	150865297	249552683.4	153268402.9
M311T768	Arachidic acid	311.169	768.2	312.3028	0.33424	C20H40O2	[M-H]-	15098240.03	12704640.59	17225871.82	14408521.8	17079957.15	12919360.08
M328T313	Cyclic AMP	328.045	312.7	329.0525	1.04467	C10H12N5O6P	[M-H]-	339074.5574	588646.9815	579101.8954	655225.25	692344.1176	640253.1732
M347T99	IMP	347.04	99	348.0471	0.50715	C10H13N4O8P	[M-H]-	1876582.89	5673901.938	621486.8734	4954647.93	1046500.255	6236835.637
M383T305_2	S-Adenosylhomocysteine	383.113	305.2	384.1216	2.35849	C14H20N6O5S	[M-H]-	30377604.28	51866331.59	43772779.24	48241786.1	74963933.18	55604899.81
M449T839	Glycochenodeoxycholic acid	449.314	839.2	449.314	0.89025	C26H43NO5	[M]-	7265154.103	3054142.762	2201372.968	8878074.23	9173490.307	10250737.55
M465T865_1	Glycocholic acid	465.303	865.1	465.309	3.7593	C26H43NO6	[M]-	59856404.99	75188345.37	72061636.89	87841431.8	55289181.98	49111011.99
M101T101	2-Ketobutyric acid	101.024	100.8	102.0317	4.94932	C4H6O3	[M-H]-	4696758.285	8826102.925	9632366.561	8528240.15	9005027.783	9751619.677
M129T118	Itaconic acid	129.019	117.8	130.0266	0	C5H6O4	[M-H]-	4768694.829	7213339.631	8727696.674	13440739.1	7969154.131	7150976.316
M130T335	Beta-Leucine	130.086	335	131.0946	8.64042	C6H13NO2	[M-H]-	3774696.054	2100561.774	6262875.881	6518433.29	6885114.07	2209603.195
M133T157	L-Malic acid	133.015	157.4	134.0215	2.07496	C4H6O5	[M-H]-	1458966.585	4241578.555	7022209.622	4592553.84	2607875.756	6295994.141
M134T247	Adenine	134.047	246.6	135.0545	0.56696	C5H5N5	[M-H]-	4008152.183	5066759.989	6352159.678	3977448.02	8200210.713	3699600.109
M138T600	4-Nitrophenol	138.021	599.7	139.0269	7.24529	C6H5NO3	[M-H]-	3876392.319	5391858.574	6154694.698	5465170.43	6424125.208	6229807.82
M148T129	L-Methionine	148.044	128.8	149.051	0	C5H11NO2S	[M-H]-	1473822.562	46269550.28	6500868.288	53970787.1	2325728.183	58771748.43
M177T76	Pyrophosphate	176.936	75.8	177.9432	1.55988	H4P2O7	[M-H]-	337668560.2	632873938.1	660324263	677704324	737493116.9	782380535.9
M179T328	4-Hydroxyphenylpyruvic acid	179.035	328.1	180.0423	0	C9H8O4	[M-H]-	274113.4858	413291.2192	395840.5058	3736321.13	428075.7725	1324338.706
M161T591	L-Gulose	161.044	591	180.0634	5.02968	C6H12O6	[M-H2O-H]-	965261.5426	5933760.552	5003034.148	7405563.36	1622183.895	4651666.415
M161T684	Alpha-D-Glucose	161.044	684.3	180.0634	5.02968	C6H12O6	[M-H2O-H]-	920537.1289	4402644.43	5355257.585	5461761.14	1504339.748	1515422.265
M161T168	Fructose-1P	161.044	167.6	180.0634	5.65063	C6H12O6	[M-H2O-H]-	1689219.207	12081680.26	18612836.29	7015162.02	2884761.768	7952140.61
M181T152	Hydroxyphenyllactic acid	181.051	152.1	182.0579	1.52443	C9H10O4	[M-H]-	3953896.115	8564597.081	6281106.121	11718317.6	8638876.449	13169885.3
M165T854	1-Methylxanthine	165.041	854	166.0491	3.63546	C6H6N4O2	[M-H]-	23731153	13399228.63	13696005.4	9230840.05	16717108.58	14211488.09
M165T332	L-3-Phenyllactic acid	165.056	331.9	166.0629942	0	C9H10O3	[M-H]-	1579420.758	3880334.154	3386700.238	6530006.8	5838383.238	6101858.96
M165T155	Terephthalate	165.019	155.3	166.0266	0.46055	C8H6O4	[M-H]-	3754288.096	4916099.858	7836255.966	16685694.6	7821844.591	9174618.923
M173T372	Shikimic acid	173.046	372.5	174.0528	2.17283	C7H10O5	[M-H]-	10480383.81	20670043.89	21953097.36	21506635.9	21669410.75	21831164.41
M176T163	N-Formyl-L-methionine	176.039	163.1	177.046	2.13589	C6H11NO3S	[M-H]-	12470484.4	11037475.17	23888152.75	17856783.1	19469499.35	17180819.61
M177T104	Gluconolactone	177.041	103.6	178.0477	1.69452	C6H10O6	[M-H]-	4911435.908	7176283.968	8991409.9	5182476.03	8145536.089	7083915.056
M221T83	L-Cystathionine	221.06	83.1	222.0674	0.56093	C7H14N2O4S	[M-H]-	132407395.8	224682413.4	209755759.4	216499289	216901043.5	210735723.7
M229T74	Ribose 1-phosphate	229.012	74.5	230.0192	0	C5H11O8P	[M-H]-	68185469.64	120598986.5	131440031.9	73267808.4	109233513.6	91416045.05
M275T71	6-Phosphogluconic acid	275.021	70.8	276.0246	13.4535	C6H13O10P	[M-H]-	47402712.23	44527472.92	64857257.04	72385381	159458202.4	149490783.8
M259T73	Galactose 1-phosphate	259.022	73	260.0297	1.1582	C6H13O9P	[M-H]-	54908757.8	47800190.67	91957404.37	28688300.7	100259474.4	70699499.92
M267T250_1	Inosine	267.074	250.1	268.0808	1.40785	C10H12N4O5	[M-H]-	592924572.8	969493775.4	1063010185	655110226	1101207796	1018812240
M267T781	Pentostatin	267.109	780.9	268.1172	2.99503	C11H16N4O4	[M-H]-	1496476.257	1560260.198	2480968.876	1672684.51	1156080.806	1346178.775
M271T965	16-Hydroxy hexadecanoic acid	271.23	965.1	272.2351	8.84857	C16H32O3	[M-H]-	1635336.247	2900288.114	3454582.982	15602462.9	12666335.01	6837567.952
M280T807	Bovinic acid	280.232	806.6	280.2402	27.834	C18H32O2	[M]-	263595.9698	4218311.986	524649.4825	6734355.36	7026025.939	1323708.952
M308T82	N-Acetyl-a-neuraminic acid	308.099	81.8	309.106	0.24667	C11H19NO9	[M-H]-	436027452	644785995.1	51267702.61	729277054	958780806	585759231.7
M295T829	9,10-Epoxyoctadecenoic acid	295.228	828.9	296.2351	0.75874	C18H32O3	[M-H]-	3877733.355	8811241.603	7967474.497	11438054.1	6372153.577	7502281.774
M301T954	Isopimaric acid	301.217	953.7	302.2246	0.25231	C20H30O2	[M-H]-	1078025.374	3179205.203	985908.6104	783654.295	331336.1054	808080.6892
M303T975	Arachidonic acid	303.233	975.4	304.2402	1.39827	C20H32O2	[M-H]-	13754501.6	27881606.11	3698592.175	7178238.61	18722893.74	17350588.9
M330T150	dAMP	330.062	149.9	331.0682	2.0481	C10H14N5O6P	[M-H]-	24367.7142	1478897.158	152541.5658	1931379.85	56127.22528	981469.3036
M319T847	8-HETE	319.227	846.5	320.2351	3.20775	C20H32O3	[M-H]-	1505542.068	6557283.227	4655586.464	4905265.6	4403450.64	3562235.435
M325T785	11-Dehydrocorticosterone	325.184	785.2	344.1988	10.7324	C21H28O4	[M-H2O-H]-	18146466.39	19656880.31	22500712.38	25756855.3	21216920.97	16402259.53
M329T956	Docosapentaenoic acid (22n-3)	329.25	956	330.2559	3.26804	C22H34O2	[M-H]-	28054838.99	35115506.06	33473922.33	40715399.9	21236825.34	36548024.96
M331T858_1	Adrenic acid	331.264	858.2	332.2715	0.97807	C22H36O2	[M-H]-	259476.739	30698438.43	74785804.66	103527255	87823213.12	41086854.82
M346T123_2	3'-AMP	346.059	123.2	347.0631	9.17763	C10H14N5O7P	[M-H]-	61140429.04	207536300.4	502588680.7	360206323	16955654.83	192991949.9
M397T788_1	Mitragynine	397.225	788	398.2206	29.8974	C23H30N2O4	[M-H]-	3897398.86	11879003.26	11051076.16	10199827.8	6508174.18	9383361.767
M458T307_1	5-Methyltetrahydrofolic acid	458.178	306.8	459.1866	2.67145	C20H25N7O6	[M-H]-	4109952.948	6882119.277	6373671.625	14065764.7	8259514.247	15080397

M540T148_2	Adenosine diphosphate ribose	540.058	147.7	559.0717	8.12876	C15H23N5O14P2	[M-H2O-H]-	1209665.489	2240760.169	1914658.175	1339065.02	1046712.767	2126653.663
M565T79_2	Uridine diphosphate glucose	565.045	78.8	566.055	5.13234	C15H24N2O17P2	[M-H]-	57363270.11	140364435.9	130855229	339912563	317840349.5	267517711
M579T72_2	Uridine diphosphate glucuronic acid	579.026	71.8	580.0343	2.41786	C15H22N2O18P2	[M-H]-	62915291.58	161311505.1	114510576.6	164330496	193406866.4	203476369.8
M606T80_3	Uridine diphosphate-N-acetylglucosamine	606.084	79.6	607.0816	15.1398	C17H27N3O17P2	[M-H]-	455399001.2	665032057.1	1331304228	1239366642	1212356204	444467129.9
M662T148_3	NAD	662.109	147.7	664.1169	10.4212	C21H28N7O14P2	[M-H]-	12984394.05	32140137.94	20977618.94	15232241	11726532.85	15995366.99
M161T347	D-Glucose	161.044	346.9	180.0634	5.65063	C6H12O6	[M-H2O-H]-	1293025.602	814980.5652	8559960.49	8280175.64	2184083.665	9565721.351
M183T444	2,4-Dinitrophenol	183.005	444	184.012	0.96172	C6H4N2O5	[M-H]-	13251372	20031632.31	24051918.43	29569494.8	21288591.9	20737852.71
M294T649	Cinchonidine	294.18	648.7	294.1732	22.4353	C19H22N2O	[M]-	2089052.601	3969642.297	4533792.363	3801641.2	2764295.368	3575126.745
M301T910	EPA (d5)	301.217	909.7	302.2246	0.07968	C20H30O2	[M-H]-	6615022.566	10887644.55	8424257.648	9330615.09	3981565.087	7123617.097
M324T80	N-Glycolylneuraminic acid	324.094	80.1	325.1009	0.85161	C11H19NO10	[M-H]-	10140242.93	20900823.44	22992599.98	27595285.6	24918678.86	26207997.84
M161T473	D-(+)-Glucose	161.044	472.6	180.0633881	4.9559	C6H12O6	[M-H2O-H]-	1043175.215	5656416.187	1892487.342	4289401.83	1797961.859	7760010.94
M188T441	N-Acetyl-L-glutamine	188.076	440.8	188.0797069	21.8364	C7H12N2O4	[M]-	1001877.933	1777588.689	1432575.196	1820314.98	1490838.24	1723378.451
M283T165	Xanthosine	283.069	165	284.0757	2.03484	C10H12N4O6	[M-H]-	3936487.556	2868363.978	591902.4366	1157354.49	6027256.037	5366531.794
M279T328	Sulfamethoxypyridazine	279.056	328.4	280.063011	0.842	C11H12N4O3S	[M-H]-	439965.817	663488.7608	729282.1625	482436.36	668869.9541	630060.0045
